# Unsupervised classification of non-Hermitian topological phases under symmetries

**DOI:** 10.1093/nsr/nwaf536

**Published:** 2025-11-27

**Authors:** Yang Long, Haoran Xue, Baile Zhang

**Affiliations:** School of Physics Science and Engineering, Tongji University, Shanghai 200092, China; Division of Physics and Applied Physics, School of Physical and Mathematical Sciences, Nanyang Technological University, Singapore 637371, Singapore; Department of Physics, The Chinese University of Hong Kong, Hong Kong 999077, China; Division of Physics and Applied Physics, School of Physical and Mathematical Sciences, Nanyang Technological University, Singapore 637371, Singapore; Centre for Disruptive Photonic Technologies, Nanyang Technological University, Singapore 637371, Singapore

**Keywords:** unsupervised learning, topological phase, non-Hermitian systems

## Abstract

The integration of machine learning into fundamental science has opened new avenues for addressing long-standing challenges rooted in mathematical limitations. For instance, while topological invariants are essential for characterizing topological phases of matter, no single invariant is universally applicable. This limitation explains why, over decades of classifying topological phases—primarily in Hermitian systems—many phases initially deemed ‘trivial’ were later recognized as topological. Recently, the discovery of non-Hermitian band topology has driven substantial efforts in non-Hermitian topological classification, leading to the development of new topological invariants. However, these invariants still fail to capture all non-Hermitian topological features. Here, without relying on any topological invariant, we develop a machine-learning algorithm for the unsupervised classification of symmetry-protected non-Hermitian topological phases. By utilizing random Hamiltonians, we unsupervisedly construct a topological periodic table without requiring advanced mathematical knowledge. Furthermore, based on the learning results, we derive a formula that reveals the impact of parity transformation on periodicity. Our algorithm can also account for boundary effects, enabling the exploration of open-boundary influences on the topological phase diagram. These findings establish an unsupervised approach for classifying symmetry-protected non-Hermitian topological phases, uncover previously unnoticed topological features in non-Hermitian systems, and provide valuable guidance for both theoretical advancements and experimental realizations.

## Teaser

A new AI system can now identify exotic states of matter by learning the underlying rules of quantum physics on its own, surpassing traditional mathematical methods.

## INTRODUCTION

Artificial intelligence (AI) for science, often referred to as ‘AI for Science’, leverages human-like intelligence to handle fundamental scientific problems [1–5]. For example, machine-learning techniques commonly used in computer vision have been employed to distinguish paramagnetic and ferromagnetic phases in the Ising model [6,7]. Restricted Boltzmann machines, traditionally used for dimensionality reduction [8], can efficiently obtain the ground states of many-body systems [9,10]. To date, machine learning has demonstrated its capability to solve problems either at a human level [11] or with significantly higher efficiency in game playing and protein predictions [12–16]. However, most applications are still constrained by human knowledge, addressing challenges within existing mathematical frameworks rather than surpassing the mathematical limitations that underpin many branches of physics.

Topology is a mathematical concept describing properties of objects preserved during continuous deformation. Its use in characterizing topological phases of matter has revolutionized condensed matter physics in the past decades [17–20]. A long-standing challenge in topology, which has carried over to the classification of topological phases of matter, is the lack of a universally applicable topological invariant. This limitation leads to a fundamental risk: even if all existing topological invariants identify a phase (or a specific Hamiltonian) as ‘trivial’, it may later be identified as topological with the development of new invariants. This issue arose in earlier classification of Hermitian topological phases, where phases initially deemed ‘trivial’, such as the topological valley Hall phase [21] and higher-order topological phases [22,23], were subsequently recognized as topological through theoretical advances.

This challenge is particularly pronounced in the emerging field of non-Hermitian topological phases [24–29]. Unlike Hermitian topological phases, which have accumulated numerous topological invariants through decades of extensive studies [30], the framework of non-Hermitian topological classification is relatively new and remains under active exploration. Significant efforts have been made in recent years to develop new topological invariants to characterize the non-Hermitian topology [31–34]. At the same time, there is a considerable interest in exploring non-Hermitian topological phases [35–39], due to their promising applications, including high-precision sensors [40,41], mode switching [42,43] and high-quality lasers [44–46].

Unsupervised learning, a major branch of AI, uncovers hidden patterns in raw data without requiring labeled training sets. It has proven effective in recognizing topological phases without relying on predefined topological invariants [47–54], addressing challenges such as randomness and disorder that extend beyond theoretical limitations [55]. Recently, unsupervised learning has been successfully applied to the topological classification of Hermitian systems under symmetry constraints [56], generating the topological periodic table in a data-driven manner—an achievement previously possible only through abstract group theory. This capability positions unsupervised learning as a promising tool for advancing topological classifications of non-Hermitian systems, e.g. capturing the braiding and knot topology in non-Hermitian bands [57–59]. While certain recent works have discussed the machine learning of non-Hermitian topological phases [60–63], symmetry-protected topological classifications remain unexplored in these studies.

In this work, we demonstrate the use of unsupervised learning in the topological classification of non-Hermitian systems under symmetries. Guided by the fundamental principles of non-Hermitian topology, we introduce a similarity function to identify topological differences based on three distinct gap types: point gap, real line gap and imaginary line gap. Using an unsupervised clustering algorithm [56], we determine the number of phases in Hamiltonian samples and classify each phase unsupervisedly. We validate our algorithm through multiple examples, focusing on topological phases induced by non-Hermiticity. Applying our algorithm to random Hamiltonian samples from non-Hermitian 38-fold symmetry classes, we construct a topological periodic table for non-Hermitian systems. This table aligns with theoretical predictions derived from homotopy groups and Clifford algebra [31–33], which primarily address abstract Hamiltonians but do not directly apply to specific Hamiltonians derived from concrete physical systems. In contrast, our algorithm can handle both concrete non-Hermitian systems and random Hamiltonians under symmetries. Furthermore, we discuss the effect of parity transformation on symmetry classes, leading to a new topological periodic table for symmetry classes that include parity transformation. Finally, we investigate the boundary effects on the non-Hermitian topological phase diagram under open boundary conditions. Compared to our previous work on topological classifications for Hermitian systems [56], here we discuss non-Hermitian systems, extend our algorithms to work on new types of band topology on the complex-energy plane that are absent in Hermitian systems and investigate the effect of non-Hermiticity-enriched symmetry classes on topological classifications.

## RESULTS

**Figure 1.**
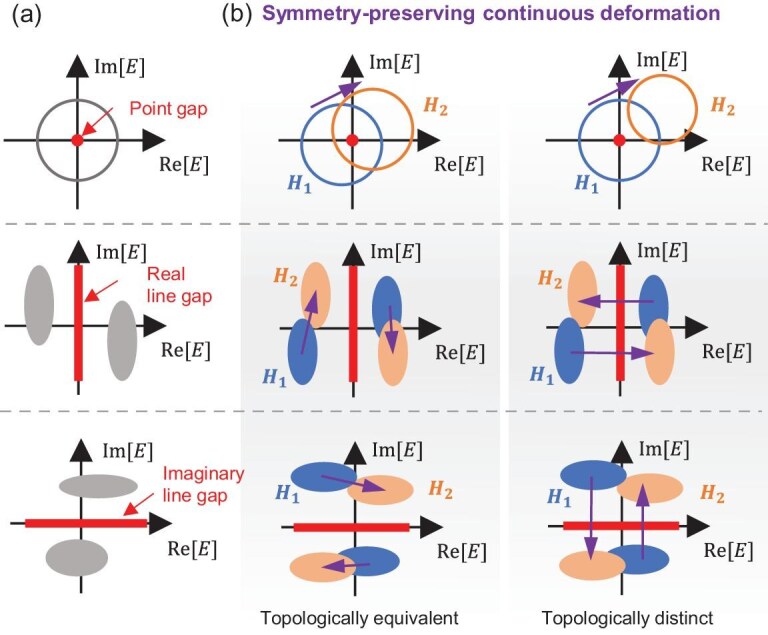
Gap types in non-Hermitian systems and symmetry-preserving continuous deformation. (a) Three typical gap types: point gap, real line gap and imaginary line gap. The gray regions denote the regions covered by the eigenenergies of the Hamiltonian on the complex-energy plane. (b) Symmetry-preserving continuous deformation between two Hamiltonians $H_1$ and $H_2$. When $H_1$ and $H_2$ are topologically equivalent, one can find a path to realize the continuous deformation between them without closing the gap. While $H_1$ and $H_2$ are topologically distinct, any continuous deformation between them will close the gap. The purple arrows denote the continuous deformation.

Hermiticity, expressed as $H=H^\dagger$, acts as a symmetry condition that constrains the allowed terms in the Hamiltonian and protects the real nature of eigenenergies, similar to the continuous time-translation symmetry in Noether’s theorem [64]. Breaking Hermiticity (i.e. $H\ne H^\dagger$) primarily leads to the emergence of complex-energy spectra, but also introduce new topological phases. One notable consequence of breaking Hermiticity is the diversification of energy gap types. While Hermitian systems typically exhibit only one type of gap, non-Hermitian systems can manifest three distinct types of gaps [32,65]: point gap, real line gap and imaginary line gap, as illustrated in Fig. 1a. A complex energy $E_f \in \mathbb {C}$, often referred as the ‘Fermi level’, serves as a reference point, with the gaps defined as (i) a point gap at the specific energy $E_f$; (ii) a real line gap along the line defined by ${\rm Re}[E_f]$ and (iii) an imaginary line gap along the line defined by ${\rm Im}[E_f]$. Furthermore, breaking Hermiticity modifies the symmetry conditions. For example, in Hermitian systems, the chiral symmetry $\Gamma$ ($U_{\Gamma } H^\dagger (\boldsymbol {k}) U_{\Gamma }^{-1} = -H(\boldsymbol {k})$) and the sublattice symmetry $\mathcal {S}$ ($U_{\mathcal {S}} H(\boldsymbol {k}) U_{\mathcal {S}}^{-1} = -H(\boldsymbol {k})$) are identical. In contrast, for non-Hermitian systems, the chiral symmetry and sublattice symmetry are different, leading to a richer variety of symmetries and topological phases.

Intuitively, similar to Hermitian systems, the topological phases of non-Hermitian systems are defined by whether there exists a continuous deformation path between two Hamiltonians without closing the gap. Specifically, as illustrated in Fig. 1b, if two non-Hermitian Hamiltonians $H_1$ and $H_2$ are topologically equivalent, there exists a continuous deformation path connecting them without closing the gap. Conversely, if $H_1$ and $H_2$ are topologically distinct, any continuous deformation between them will inevitably result in gap closing. Rather than searching for such a continuous path [50], we define an initial continuous path and subsequently assess its robustness by introducing symmetry-preserving perturbations [56]. In our approach, the continuous deformation between two Hamiltonians $H_1$ and $H_2$ is realized using linear interpolation: $H_\alpha = (1-\alpha ) H_1 + \alpha H_2$ with $a\in [0,1]$. This method preserves the representation basis and ensures that the symmetry conditions are maintained throughout the deformation process. To identify the topological difference between $H_1$ and $H_2$, we detect the presence of a topologically protected crossing point at the Fermi level $E_f$ [56] within the interpolated Hamiltonian $H_\alpha$. Since the eigenenergies of $H_{1(2)}$ can be arbitrary, we utilize the flattened Hamiltonian $Q_{1(2)}$ of $H_{1(2)}$. This allows us to detect the topologically protected crossing point in $Q_\alpha = (1-\alpha ) Q_1 + \alpha Q_2$ rather than in $H_\alpha$ (see the online supplementary material).

We define the similarity function based on the flattened Hamiltonian $Q$. Although the similarity function depends on the gap type, it can be expressed in a compact form (see the online supplementary material). For a non-Hermitian Hamiltonian $H$, we consider the eigenequations $H |\psi _{n,\boldsymbol {k}}\rangle = E_n |\psi _{n,\boldsymbol {k}}\rangle$ and $H^\dagger |\varphi _{n,\boldsymbol {k}}\rangle = E_n^{*} |\varphi _{n,\boldsymbol {k}}\rangle$. For a line gap, the projection operator of the $n$th band can be defined as $P(\boldsymbol {k}) = \sum _{n \in cocc} |\psi _{n, \boldsymbol {k}}\rangle \langle \varphi _{n,\boldsymbol {k}}|$, where $cocc$ denotes the complex occupied bands. The flattened Hamiltonian $Q$ is then given by $Q(\boldsymbol {k}) = 1-2P(\boldsymbol {k})$. Here, $cocc$ depends on the type of line gap: (i) for a real line gap, $cocc = \lbrace n\mid {\rm Re}[E_n] < {\rm Re}[E_f] \rbrace$; (ii) for an imaginary line gap, $cocc = \lbrace n\mid {\rm Im}[E_n] < {\rm Im}[E_f] \rbrace$. We define $v_{\rm line} = \Pi _n {\rm Re}[\lambda _n]$, where $\lbrace \lambda _n\rbrace$ are the eigenvalues of $Q_i(\boldsymbol {k})+Q_j(\boldsymbol {k})$. For a point gap, we define


\begin{eqnarray*}
\widetilde{H} = \Big({\begin{array}{cc}0 &\quad H-E_f \\
H^\dagger - E_f^{*} &\quad 0 \end{array}}\Big),
\end{eqnarray*}

which maps $H$ to a Hermitian Hamiltonian $\widetilde{H}$ with the emergent chiral symmetry, $\sigma _z \widetilde{H}(\boldsymbol {k}) \sigma _z = - \widetilde{H} (\boldsymbol {k})$ (see the online supplementary material). The projection operator for $\widetilde{H}$ is $\widetilde{P}(\boldsymbol {k}) = \sum _{n \in occ} |\widetilde{\varphi }_{n, \boldsymbol {k}}\rangle \langle \widetilde{\varphi }_{n,\boldsymbol {k}}|$, where $\widetilde{H} |\widetilde{\varphi }_{n, \boldsymbol {k}}\rangle = \widetilde{E}_n |\widetilde{\varphi }_{n, \boldsymbol {k}}\rangle$ and $occ= \lbrace n|\widetilde{E}_n<0\rbrace$. The flattened Hamiltonian $\widetilde{Q}$ is given by $\widetilde{Q}(\boldsymbol {k}) = 1-2\widetilde{P}(\boldsymbol {k})$. We define $v_{\rm point} = \Pi _n \widetilde{\lambda }_n$, where $\lbrace \widetilde{\lambda }_n\rbrace$ are the eigenvalues of $\widetilde{Q}_i(\boldsymbol {k})+\widetilde{Q}_j(\boldsymbol {k})$. The similarity function $\mathcal {K}_{ij}$ between the Hamiltonian sample $H_i$ and $H_j$ is then defined as


(1) \begin{equation*}
\mathcal {K}^{\rm point/line}_{ij} = \prod _{k\in {\rm BZ}} \bigg (1-\exp \bigg [-\frac{|v_{\rm point/line} |^2}{\varepsilon ^2} \bigg ] \bigg ),
\end{equation*}

where $\varepsilon \in \mathbb {R}$ and the subscript ${\rm point/line}$ denotes the gap type. Based on Equation (1), the corresponding distance function is $d_{ij} = 1 - \mathcal {K}_{ij}$. During the calculation, symmetry-preserving perturbations are introduced to test the robustness of the crossing point (see the online supplementary material). In practice, we set $\varepsilon \rightarrow 0$, so that the similarity function becomes a binary function: $\mathcal {K}_{ij} = 1$ for topologically equivalent Hamiltonians, and $\mathcal {K}_{ij} = 0$ for topologically distinct ones [56].

We apply our previously proposed clustering algorithm in [56] to detect the number of phases and identify the phases in the Hamiltonian samples $\lbrace H_i \rbrace $. Below, we provide a brief overview of the algorithm. The algorithm operates on a set $\mathcal {G}$ and a list $\lbrace M_c\rbrace$, where $\mathcal {G} = \lbrace H_{p_c}\rbrace$ is a set of samples that are mutually different (i.e., $\mathcal {K}_{p_c p_{c^{\prime }}}<1/2$ for all $H_{p_c}, H_{p_{c^{\prime }}}\in \mathcal {G}$) and $M_c$ denotes the number of samples that are topologically equivalent to $H_{p_c}$, $\lbrace M_c\mid c=1,2,\dots ,N_c\rbrace$. The algorithm proceeds in two steps.

(1) The first sample $H_1$ is added into $\mathcal {G}$ since the initial $\mathcal {G} = \emptyset$. Then, $\mathcal {G}=\lbrace H_1\rbrace$, $p_1=1$, $M_1 = 1$ and $N_c=1$.

(2) For each subsequent sample $H_j$, the algorithm compares it with the samples in $\mathcal {G}$. If $H_j$ is topologically equivalent to $H_{p_c}$, i.e. $H_{p_c}\in \mathcal {S}$ and $\mathcal {K}_{j,p_c}> \kappa _c$, then $M_c := M_c + 1$. Otherwise, if none of the samples in $\mathcal {G}$ is topologically equivalent to $H_j$, $H_j$ is added into $\mathcal {G}$, $M_{N_c+1} = 1$, $p_{N_c+1}=j$ and $N_c := N_c + 1$.

After processing all the samples in $\lbrace H_i \rbrace$, we obtain the following: $N_c$ denotes the number of topologically distinct phases, and $\lbrace M_c\rbrace$ denotes the number of samples that have the same phase as $H_{p_c}$ (see the online supplementary material). The index $c$ is used to label the topologically distinct phases.

**Figure 2.**
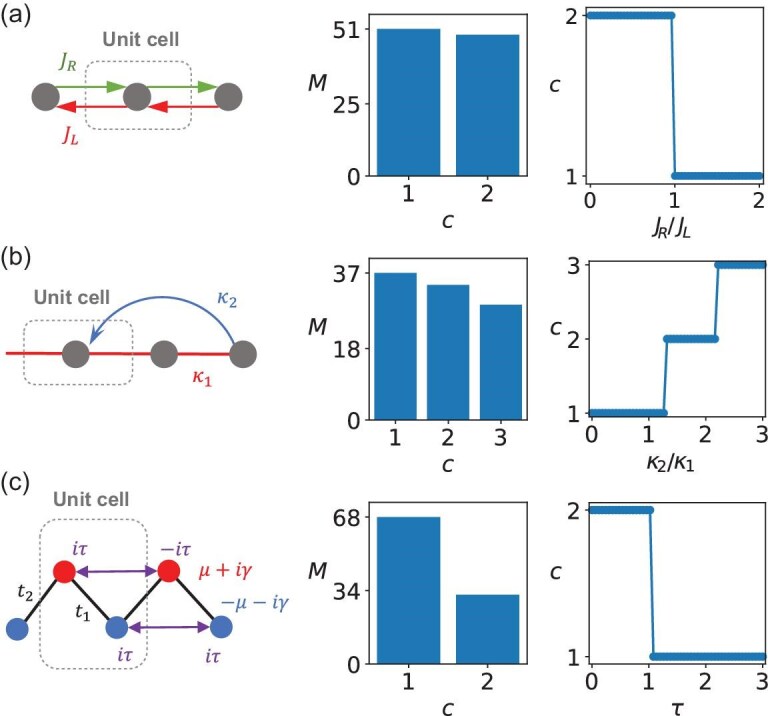
Unsupervised learning of non-Hermitian point-gap topological phases. The left plots represent the system settings. The central plots represent the number of samples $M$ for the topologically distinct phases. Index $c$ denotes the custom label of the phases. The right plots show the topological phase diagrams obtained from the similarity between the Hamiltonian with the given parameters and Hamiltonians in $\mathcal {G}$. The different colors and labels denote the topologically distinct phases, but not the topological invariants. (a) One-dimensional Hatano–Nelson system. We set $J_L=1$, $J_R \in [0,2]$, $E_f=0$. (b) One-dimensional non-Hermitian system with twisted winding in the complex-energy plane. We set $\kappa =1$, $\kappa _2 \in [0,3]$, $E_f=i$. (c) One-dimensional non-Hermitian topological point-gap phase induced by on-site losses and gains. We set $t_1=\gamma =2$, $t_2=\mu =1$, $\tau \in [0,3]$ and $E_f=0$. Here, we generate 100 samples for each case.

In the following, we demonstrate the validity of our algorithm. Firstly, we apply the algorithm to identify the non-Hermiticity-induced point-gap topological phases. Typical cases include the one-dimensional (1D) Hatano–Nelson model in Fig. 2a [29,66], a 1D non-Hermitian system with twisted loop in Fig. 2b [67] and a loss-and-gain-induced point-gap topological system in Fig. 2c [68]. For each mode, we generate samples with random parameters, calculate their similarities and apply the clustering algorithm to determine the number of topologically distinct phases. We can see that the number of topologically distinct phases, denoted $N_c$, is found to be $N_c=2$ in Fig. 2a, $N_c=3$ in Fig. 2b and $N_c = 2$ in Fig. 2c. After labeling all the phases (i.e. assigning different values of $c$), we calculate their similarities with the samples and classify samples based on the label of the sample $H_{p_c}$ that has the maximum similarity. Consequently, we can obtain the topological phase diagrams in an unsupervised manner, as shown in Fig. 2.

**Figure 3.**
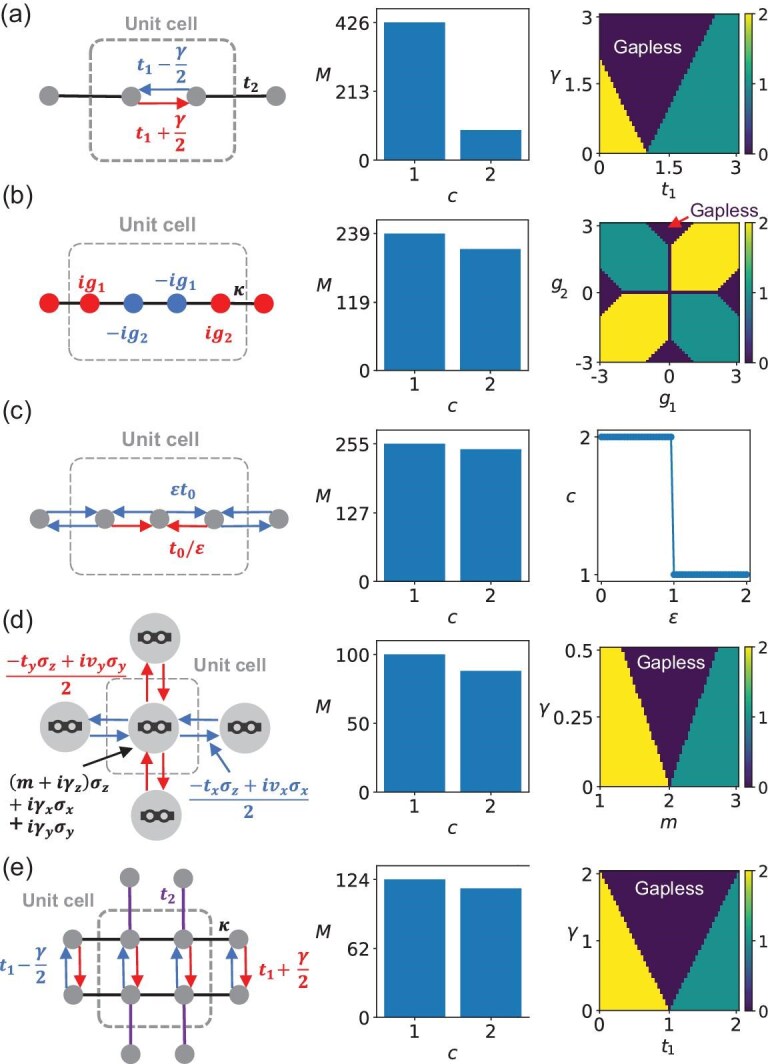
Unsupervised learning of non-Hermitian real line-gap topological phases. The left plots represent the system settings. The central plots represent the number of samples $M$ for the topologically distinct phases. Index $c$ denotes the label of the phases. The right plots show the topological phase diagrams obtained from the similarity between the Hamiltonian with the given parameters and Hamiltonians in $\mathcal {G}$. The different colors and labels denote the topologically distinct phases, but not the topological invariants. Note that $c=0$ denotes the gapless system. (a) One-dimensional non-Hermitian SSH system. We set $t_2=1$, $t_1\in [0,3]$, $\gamma \in [0,3]$. (b) One-dimensional topological insulator phase solely induced by on-site gains and losses. We set $\kappa =1$, $g_1,g_2\in [-3,3]$. (c) One-dimensional topological insulator phase solely induced by non-reciprocal couplings. We set $t_0=1$, $\varepsilon \in [0,2]$. (d) Two-dimensional non-Hermitian Chern insulator. We set $t_x=t_y=v_x=v_y=1$, $m\in [1,3]$, $\gamma \in [0,0.5]$. (e) Two-dimensional non-Hermitian topological Möbius insulator. We set $\kappa =0.25$, $t_2=1$, $t_1 \in [0,2]$, $\gamma \in [0,2]$. Here, to obtain the central plots, we generate 500 samples for each case and filter out the gapless systems. For all cases, $E_f=0$.

Secondly, non-Hermiticity can also induce new topological phases in systems with a line gap [69–73]. Below we take the real line gap as an example. We apply our algorithm to systems with the real line-gap topology, including the 1D non-Hermitian Su–Schrieffer–Heeger (SSH) system in Fig. 3a [74], the 1D topological system with on-site gain and loss in Fig. 3b [69], the 1D topological system with non-reciprocal couplings in Fig. 3c [75], the 2D non-Hermitian Chern insulator in Fig. 3d [76] and the 2D non-Hermitian topological Möbius insulator in Fig. 3e (see the online supplementary material). After generating random parameter samples for each model, we calculate their similarities and perform the clustering algorithm to determine the number of topologically distinct phases. In the same manner as before, we can obtain the topological phase diagrams unsupervisedly, as shown in Fig. 3. These results align well with theoretical predictions based on topological invariants (see the online supplementary material).

**Figure 4.**
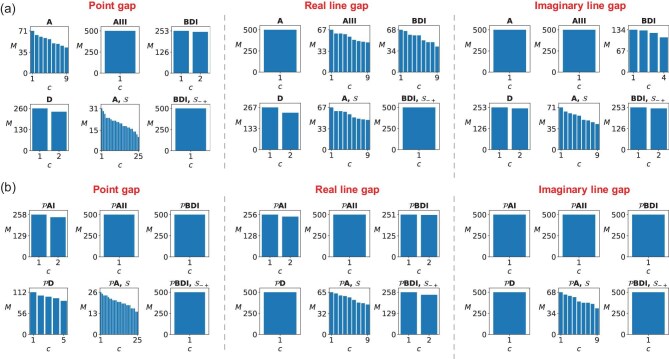
Unsupervised learning of topological classifications of 1D non-Hermitian Hamiltonians in different symmetry classes. (a) Classification results for non-Hermitian symmetry classes. Here, we demonstrate six symmetry classes for each type of gap. (b) Classification results for non-Hermitian symmetry classes with consideration of the parity transformation. Here, we randomly generate 500 Hamiltonian samples for each symmetry class according to the 0D $n\times n$ Hamiltonians (see the online supplementary material). The number of phases $N_c$ can reflect the topological classification, e.g. $N_c=2$ corresponds to $\mathbb {Z}_2$ and $N_c =n+1$ corresponds to $\mathbb {Z}$. Here, we set $n=8$ and $E_f=0$.

Here, we apply our algorithm to achieve topological classification of non-Hermitian systems within the non-Hermitian symmetry classes. Non-Hermiticity enriches the symmetry classes into 38 distinct classes [31,32,77], defined by combinations of not only time-reversal ($\mathcal {T}_{\pm }$), particle-hole ($\mathcal {C}_{\pm }$) and chiral ($\Gamma$) symmetries, but also sublattice ($\mathcal {S}$) and pseudo-Hermiticity ($\eta$) symmetries. By randomly generating Hamiltonian samples for each symmetry class, we employ our algorithm to determine the number of topologically distinct phases (see the online supplementary material). The resulting number of distinct phases from these randomly generated Hamiltonian samples under symmetries reveals the topological classification and reconstructs the topological periodic table [56]. As shown in Fig. 4a, we demonstrate unsupervised classification of 1D non-Hermitian systems for three types of gaps across selected symmetry classes.

From our calculations, we summarize the topological classification results for non-Hermitian systems under symmetries in Table 1. Higher-dimensional non-Hermitian Hamiltonians are generated based on 0D $n$-banded Hamiltonians [30,56,78] (see the online supplementary material). Table 1 provides key information on the symmetry conditions and dimensions under which a system can exhibit topological phases, as well as the maximum number of phases. From Table 1, we observe the following key features. (i) The classification exhibits an eight-fold periodicity with respect to the dimension $d$, similar to the Bott periodicity in the topological periodic table for Hermitian systems. (ii) The classification results for a given symmetry class can vary depending on the type of gap considered. When $n\rightarrow \infty$, the topological classification can be deduced from the number of phases $N_c$ [56] as follows:

(1)
$N_c=1$ corresponds to a trivial group,
(2)
$N_c=n+1$ corresponds to group $\mathbb {Z}$,
(3)
$N_c=n/2 + 1$ corresponds to $2\mathbb {Z}$,
(4)
$N_c=2$ corresponds to $\mathbb {Z}_2$,
(5)
$N_c=({n}/{2}+1)^2$ corresponds to $\mathbb {Z}\oplus \mathbb {Z}$,
(6)
$N_c=({n}/{4} + 1)^2$ corresponds to $2\mathbb {Z}\oplus 2\mathbb {Z}$,
(7)
$N_c=2\times 2$ corresponds to $\mathbb {Z}_2 \oplus \mathbb {Z}_2$.

Although certain theoretical approaches based on the homotopy groups of the classifying space for abstract Hamiltonians [31,32,34], Clifford algebra [33] or topological field theory [79] can achieve topological classification, they are not directly applicable for distinguishing the topological phase differences between specific Hamiltonians and usually require some assumptions like $n\rightarrow \infty$. Further details can be found in the online supplementary material.

**Table 1.** The number of topologically different phases $N_c$ for the $d$-dimensional non-Hermitian Hamiltonians in different symmetry classes. Non-Hermitian topological phases are classified according to the symmetry classes, the dimension $d$ and the definition of complex-energy point (P) or line (L) gaps. The subscript of L denotes the line gap for the real or imaginary part of the complex spectrum. By $\mathcal {S}$ we denote the sublattice symmetry. The subscript of $\mathcal {S}_{\pm }$ denotes the commutation/anti-commutation relation to time-reversal $\mathcal {T}$ or particle-hole $\mathcal {C}$ symmetry. When $\mathcal {T}$ and $\mathcal {C}$ symmetries coexist, the first sign specifies the relation to $\mathcal {T}$ symmetry and the second sign to $\mathcal {C}$ symmetry. Here, we generate the higher-dimensional Hamiltonians based on the 0D $n \times n$ random Hamiltonians (see the online supplementary material).

Symmetry class	Gap	$d=0$	$d=1$	$d=2$	$d=3$	$d=4$	$d=5$	$d=6$	$d=7$	$d=8$	$d=9$
A	P	1	$n+1$	1	$n+1$	1	$n+1$	1	$n+1$	1	$n+1$
	${\rm L}_{\rm r}$	$n+1$	1	$n+1$	1	$n+1$	1	$n+1$	1	$n+1$	1
	${\rm L}_{\rm i}$	$n+1$	1	$n+1$	1	$n+1$	1	$n+1$	1	$n+1$	1
AIII	P	$n+1$	1	$n+1$	1	$n+1$	1	$n+1$	1	$n+1$	1
	${\rm L}_{\rm r}$	1	$n+1$	1	$n+1$	1	$n+1$	1	$n+1$	1	$n+1$
	${\rm L}_{\rm i}$	$\left(\frac{n}{2}+1\right)^2$	1	$\left(\frac{n}{2}+1\right)^2$	1	$\left(\frac{n}{2}+1\right)^2$	1	$\left(\frac{n}{2}+1\right)^2$	1	$\left(\frac{n}{2}+1\right)^2$	1
AI	P	2	$n+1$	1	1	1	$\frac{n}{2}+1$	1	2	2	$n+1$
	${\rm L}_{\rm r}$	$n+1$	1	1	1	$\frac{n}{2}+1$	1	2	2	$n+1$	1
	${\rm L}_{\rm i}$	2	2	$n+1$	1	1	1	$\frac{n}{2}+1$	1	2	2
BDI	P	2	2	$n+1$	1	1	1	$\frac{n}{2}+1$	1	2	2
	${\rm L}_{\rm r}$	2	$n+1$	1	1	1	$\frac{n}{2}+1$	1	2	2	$n+1$
	${\rm L}_{\rm i}$	$2\times 2$	$2\times 2$	$\left(\frac{n}{2}+1\right)^2$	1	1	1	$\left(\frac{n}{4}+1\right)^2$	1	$2\times 2$	$2\times 2$
D	P	1	2	2	$n+1$	1	1	1	$\frac{n}{2}+1$	1	2
	${\rm L}_{\rm r}$	2	2	$n+1$	1	1	1	$\frac{n}{2}+1$	1	2	2
	${\rm L}_{\rm i}$	2	2	$n+1$	1	1	1	$\frac{n}{2}+1$	1	2	2
DIII	P	$\frac{n}{2}+1$	1	2	2	$n+1$	1	1	1	$\frac{n}{2}+1$	1
	${\rm L}_{\rm r}$	1	2	2	$n+1$	1	1	1	$\frac{n}{2}+1$	1	2
	${\rm L}_{\rm i}$	$n+1$	1	$n+1$	1	$n+1$	1	$n+1$	1	$n+1$	1
AII	P	1	$\frac{n}{2}+1$	1	2	2	$n+1$	1	1	1	$\frac{n}{2}+1$
	${\rm L}_{\rm r}$	$\frac{n}{2}+1$	1	2	2	$n+1$	1	1	1	$\frac{n}{2}+1$	1
	${\rm L}_{\rm i}$	1	1	$\frac{n}{2}+1$	1	2	2	$n+1$	1	1	1
CII	P	1	1	$\frac{n}{2}+1$	1	2	2	$n+1$	1	1	1
	${\rm L}_{\rm r}$	1	$\frac{n}{2}+1$	1	2	2	$n+1$	1	1	1	$\frac{n}{2}+1$
	${\rm L}_{\rm i}$	1	1	$\left(\frac{n}{4}+1\right)^2$	1	$2\times 2$	$2\times 2$	$\left(\frac{n}{2}+1\right)^2$	1	1	1
C	P	1	1	1	$\frac{n}{2}+1$	1	2	2	$n+1$	1	1
	${\rm L}_{\rm r}$	1	1	$\frac{n}{2}+1$	1	2	2	$n+1$	1	1	1
	${\rm L}_{\rm i}$	1	1	$\frac{n}{2}+1$	1	2	2	$n+1$	1	1	1
CI	P	$n+1$	1	1	1	$\frac{n}{2}+1$	1	2	2	$n+1$	1
	${\rm L}_{\rm r}$	1	1	1	$\frac{n}{2}+1$	1	2	2	$n+1$	1	1
	${\rm L}_{\rm i}$	$n+1$	1	$n+1$	1	$n+1$	1	$n+1$	1	$n+1$	1
AI$^\dagger$	P	1	1	1	$\frac{n}{2}+1$	1	2	2	$n+1$	1	1
	${\rm L}_{\rm r}$	$n+1$	1	1	1	$\frac{n}{2}+1$	1	2	2	$n+1$	1
	${\rm L}_{\rm i}$	$n+1$	1	1	1	$\frac{n}{2}+1$	1	2	2	$n+1$	1
BDI$^\dagger$	P	$n+1$	1	1	1	$\frac{n}{2}+1$	1	2	2	$n+1$	1
	${\rm L}_{\rm r}$	2	$n+1$	1	1	1	$\frac{n}{2}+1$	1	2	2	$n+1$
	${\rm L}_{\rm i}$	$\left(\frac{n}{2}+1\right)^2$	1	1	1	$\left(\frac{n}{4}+1\right)^2$	1	$2\times 2$	$2\times 2$	$\left(\frac{n}{2}+1\right)^2$	1
DIII$^\dagger$	P	2	2	$n+1$	1	1	1	$\frac{n}{2}+1$	1	2	2
	${\rm L}_{\rm r}$	1	2	2	$n+1$	1	1	1	$\frac{n}{2}+1$	1	2
	${\rm L}_{\rm i}$	$n+1$	1	$n+1$	1	$n+1$	1	$n+1$	1	$n+1$	1
AII$^\dagger$	P	1	2	2	$n+1$	1	1	1	$\frac{n}{2}+1$	1	2
	${\rm L}_{\rm r}$	$\frac{n}{2}+1$	1	2	2	$n+1$	1	1	1	$\frac{n}{2}+1$	1
	${\rm L}_{\rm i}$	$\frac{n}{2}+1$	1	2	2	$n+1$	1	1	1	$\frac{n}{2}+1$	1
CII$^\dagger$	P	$\frac{n}{2}+1$	1	2	2	$n+1$	1	1	1	$\frac{n}{2}+1$	1
	${\rm L}_{\rm r}$	1	$\frac{n}{2}+1$	1	2	2	$n+1$	1	1	1	$\frac{n}{2}+1$
	${\rm L}_{\rm i}$	$\left(\frac{n}{4}+1\right)^2$	1	$2\times 2$	$2\times 2$	$\left(\frac{n}{2}+1\right)^2$	1	1	1	$\left(\frac{n}{4}+1\right)^2$	1
CI$^\dagger$	P	1	1	$\frac{n}{2}+1$	1	2	2	$n+1$	1	1	1
	${\rm L}_{\rm r}$	1	1	1	$\frac{n}{2}+1$	1	2	2	$n+1$	1	1
	${\rm L}_{\rm i}$	$n+1$	1	$n+1$	1	$n+1$	1	$n+1$	1	$n+1$	1
AIII, $\mathcal {S}_{+}$	P	1	$n+1$	1	$n+1$	1	$n+1$	1	$n+1$	1	$n+1$
	${\rm L}_{\rm r}$	1	$\left(\frac{n}{2}+1\right)^2$	1	$\left(\frac{n}{2}+1\right)^2$	1	$\left(\frac{n}{2}+1\right)^2$	1	$\left(\frac{n}{2}+1\right)^2$	1	$\left(\frac{n}{2}+1\right)^2$
	${\rm L}_{\rm i}$	1	$\left(\frac{n}{2}+1\right)^2$	1	$\left(\frac{n}{2}+1\right)^2$	1	$\left(\frac{n}{2}+1\right)^2$	1	$\left(\frac{n}{2}+1\right)^2$	1	$\left(\frac{n}{2}+1\right)^2$
A, $\mathcal {S}$	P	1	$\left(\frac{n}{2}+1\right)^2$	1	$\left(\frac{n}{2}+1\right)^2$	1	$\left(\frac{n}{2}+1\right)^2$	1	$\left(\frac{n}{2}+1\right)^2$	1	$\left(\frac{n}{2}+1\right)^2$
	${\rm L}_{\rm r}$	1	$n+1$	1	$n+1$	1	$n+1$	1	$n+1$	1	$n+1$
	${\rm L}_{\rm i}$	1	$n+1$	1	$n+1$	1	$n+1$	1	$n+1$	1	$n+1$
AIII, $\mathcal {S}_{-}$	P	$\left(\frac{n}{2}+1\right)^2$	1	$\left(\frac{n}{2}+1\right)^2$	1	$\left(\frac{n}{2}+1\right)^2$	1	$\left(\frac{n}{2}+1\right)^2$	1	$\left(\frac{n}{2}+1\right)^2$	1
	${\rm L}_{\rm r}$	$n+1$	1	$n+1$	1	$n+1$	1	$n+1$	1	$n+1$	1
	${\rm L}_{\rm i}$	$n+1$	1	$n+1$	1	$n+1$	1	$n+1$	1	$n+1$	1
BDI, $\mathcal {S}_{++}$	P	2	$n+1$	1	1	1	$\frac{n}{2}+1$	1	2	2	$n+1$
	${\rm L}_{\rm r}$	$2\times 2$	$\left(\frac{n}{2}+1\right)^2$	1	1	1	$\frac{n}{2}+1$	1	$2\times 2$	$2\times 2$	$\left(\frac{n}{2}+1\right)^2$
	${\rm L}_{\rm i}$	$2\times 2$	$\left(\frac{n}{2}+1\right)^2$	1	1	1	$\frac{n}{2}+1$	1	$2\times 2$	$2\times 2$	$\left(\frac{n}{2}+1\right)^2$
DIII, $\mathcal {S}_{--}$	P	1	2	2	$n+1$	1	1	1	$\frac{n}{2}+1$	1	2
	${\rm L}_{\rm r}$	1	$2\times 2$	$2\times 2$	$\left(\frac{n}{2}+1\right)^2$	1	1	1	$\left(\frac{n}{4}+1\right)^2$	1	$2\times 2$
	${\rm L}_{\rm i}$	1	$n+1$	1	$n+1$	1	$n+1$	1	$n+1$	1	$n+1$
CII, $\mathcal {S}_{++}$	P	1	$\frac{n}{2}+1$	1	2	2	$n+1$	1	1	1	$\frac{n}{2}+1$
	${\rm L}_{\rm r}$	1	$\left(\frac{n}{4}+1\right)^2$	1	$2\times 2$	$2\times 2$	$\left(\frac{n}{2}+1\right)^2$	1	1	1	$\left(\frac{n}{4}+1\right)^2$
	${\rm L}_{\rm i}$	1	$\left(\frac{n}{4}+1\right)^2$	1	$2\times 2$	$2\times 2$	$\left(\frac{n}{2}+1\right)^2$	1	1	1	$\left(\frac{n}{4}+1\right)^2$
CI, $\mathcal {S}_{--}$	P	1	1	1	$\frac{n}{2}+1$	1	2	2	$n+1$	1	1
	${\rm L}_{\rm r}$	1	1	1	$\left(\frac{n}{4}+1\right)^2$	1	$2\times 2$	$2\times 2$	$\left(\frac{n}{2}+1\right)^2$	1	1
	${\rm L}_{\rm i}$	1	$n+1$	1	$n+1$	1	$n+1$	1	$n+1$	1	$n+1$
AI, $\mathcal {S}_{-}$	P	1	$n+1$	1	$n+1$	1	$n+1$	1	$n+1$	1	$n+1$
	${\rm L}_{\rm r}$	1	1	1	$\frac{n}{2}+1$	1	2	2	$n+1$	1	1
	${\rm L}_{\rm i}$	1	2	2	$n+1$	1	1	1	$\frac{n}{2}+1$	1	2
BDI, $\mathcal {S}_{-+}$	P	$n+1$	1	$n+1$	1	$n+1$	1	$n+1$	1	$n+1$	1
	${\rm L}_{\rm r}$	$n+1$	1	1	1	$\frac{n}{2}+1$	1	2	2	$n+1$	1
	${\rm L}_{\rm i}$	2	2	$n+1$	1	1	1	$\frac{n}{2}+1$	1	2	2
D, $\mathcal {S}_{+}$	P	1	$n+1$	1	$n+1$	1	$n+1$	1	$n+1$	1	$n+1$
	${\rm L}_{\rm r}$	2	$n+1$	1	1	1	$\frac{n}{2}+1$	1	2	2	$n+1$
	${\rm L}_{\rm i}$	2	$n+1$	1	1	1	$\frac{n}{2}+1$	1	2	2	$n+1$
CII, $\mathcal {S}_{-+}$	P	$n+1$	1	$n+1$	1	$n+1$	1	$n+1$	1	$n+1$	1
	${\rm L}_{\rm r}$	$\frac{n}{2}+1$	1	2	2	$n+1$	1	1	1	$\frac{n}{2}+1$	1
	${\rm L}_{\rm i}$	1	1	$\frac{n}{2}+1$	1	2	2	$n+1$	1	1	1
C, $\mathcal {S}_{+}$	P	1	$n+1$	1	$n+1$	1	$n+1$	1	$n+1$	1	$n+1$
	${\rm L}_{\rm r}$	1	$\frac{n}{2}+1$	1	2	2	$n+1$	1	1	1	$\frac{n}{2}+1$
	${\rm L}_{\rm i}$	1	$\frac{n}{2}+1$	1	2	2	$n+1$	1	1	1	$\frac{n}{2}+1$
DIII, $\mathcal {S}_{++}$	P	1	$\frac{n}{2}+1$	1	2	2	$n+1$	1	1	1	$\frac{n}{2}+1$
	${\rm L}_{\rm r}$	1	$n+1$	1	$n+1$	1	$n+1$	1	$n+1$	1	$n+1$
	${\rm L}_{\rm i}$	1	$n+1$	1	$n+1$	1	$n+1$	1	$n+1$	1	$n+1$
CI, $\mathcal {S}_{++}$	P	2	$n+1$	1	1	1	$\frac{n}{2}+1$	1	2	2	$n+1$
	${\rm L}_{\rm r}$	1	$n+1$	1	$n+1$	1	$n+1$	1	$n+1$	1	$n+1$
	${\rm L}_{\rm i}$	1	$n+1$	1	$n+1$	1	$n+1$	1	$n+1$	1	$n+1$
AI, $\mathcal {S}_{+}$	P	2	$n+1$	1	1	1	$\frac{n}{2}+1$	1	2	2	$n+1$
	${\rm L}_{\rm r}$	2	$n+1$	1	1	1	$\frac{n}{2}+1$	1	2	2	$n+1$
	${\rm L}_{\rm i}$	2	$n+1$	1	1	1	$\frac{n}{2}+1$	1	2	2	$n+1$
BDI, $\mathcal {S}_{+-}$	P	$2\times 2$	$2\times 2$	$\left(\frac{n}{2}+1\right)^2$	1	1	1	$\left(\frac{n}{4}+1\right)^2$	1	$2\times 2$	$2\times 2$
	${\rm L}_{\rm r}$	2	2	$n+1$	1	1	1	$\frac{n}{2}+1$	1	2	2
	${\rm L}_{\rm i}$	2	2	$n+1$	1	1	1	$\frac{n}{2}+1$	1	2	2
D, $\mathcal {S}_{-}$	P	1	$2\times 2$	$2\times 2$	$\left(\frac{n}{2}+1\right)^2$	1	1	1	$\left(\frac{n}{4}+1\right)^2$	1	$2\times 2$
	${\rm L}_{\rm r}$	1	2	2	$n+1$	1	1	1	$\frac{n}{2}+1$	1	2
	${\rm L}_{\rm i}$	1	2	2	$n+1$	1	1	1	$\frac{n}{2}+1$	1	2
DIII, $\mathcal {S}_{+-}$	P	$\left(\frac{n}{4}+1\right)^2$	1	$2\times 2$	$2\times 2$	$\left(\frac{n}{2}+1\right)^2$	1	1	1	$\left(\frac{n}{4}+1\right)^2$	1
	${\rm L}_{\rm r}$	$\frac{n}{2}+1$	1	2	2	$n+1$	1	1	1	$\frac{n}{2}+1$	1
	${\rm L}_{\rm i}$	$\frac{n}{2}+1$	1	2	2	$n+1$	1	1	1	$\frac{n}{2}+1$	1
AII, $\mathcal {S}_{+}$	P	1	$\left(\frac{n}{4}+1\right)^2$	1	$2\times 2$	$2\times 2$	$\left(\frac{n}{2}+1\right)^2$	1	1	1	$\left(\frac{n}{4}+1\right)^2$
	${\rm L}_{\rm r}$	1	$\frac{n}{2}+1$	1	2	2	$n+1$	1	1	1	$\frac{n}{2}+1$
	${\rm L}_{\rm i}$	1	$\frac{n}{2}+1$	1	2	2	$n+1$	1	1	1	$\frac{n}{2}+1$
CII, $\mathcal {S}_{+-}$	P	1	1	$\left(\frac{n}{4}+1\right)^2$	1	$2\times 2$	$2\times 2$	$\left(\frac{n}{2}+1\right)^2$	1	1	1
	${\rm L}_{\rm r}$	1	1	$\frac{n}{2}+1$	1	2	2	$n+1$	1	1	1
	${\rm L}_{\rm i}$	1	1	$\frac{n}{2}+1$	1	2	2	$n+1$	1	1	1
C, $\mathcal {S}_{-}$	P	1	1	1	$\left(\frac{n}{4}+1\right)^2$	1	$2\times 2$	$2\times 2$	$\left(\frac{n}{2}+1\right)^2$	1	1
	${\rm L}_{\rm r}$	1	1	1	$\frac{n}{2}+1$	1	2	2	$n+1$	1	1
	${\rm L}_{\rm i}$	1	1	1	$\frac{n}{2}+1$	1	2	2	$n+1$	1	1
CI, $\mathcal {S}_{+-}$	P	$\left(\frac{n}{2}+1\right)^2$	1	1	1	$\left(\frac{n}{4}+1\right)^2$	1	$2\times 2$	$2\times 2$	$\left(\frac{n}{2}+1\right)^2$	1
	${\rm L}_{\rm r}$	$n+1$	1	1	1	$\frac{n}{2}+1$	1	2	2	$n+1$	1
	${\rm L}_{\rm i}$	$n+1$	1	1	1	$\frac{n}{2}+1$	1	2	2	$n+1$	1

Parity-time ($\mathcal {PT}$) symmetry, defined as $\mathcal {PT}:\boldsymbol {r}\rightarrow -\boldsymbol {r}$, $t\rightarrow -t$, is a fundamental symmetry in non-Hermitian systems and plays a pivotal role in the development of non-Hermitian devices [26,28,35,80–83]. To investigate how parity transformation affects topological classification, we combine symmetry conditions with the parity transformation $\mathcal {P}$ ($\boldsymbol {r} \rightarrow -\boldsymbol {r}$) (see the online supplementary material). This transformation modifies $\mathcal {T}$ symmetry and $\mathcal {C}$ symmetry into $\mathcal {PT}$ symmetry and $\mathcal {PC}$ symmetry [84], respectively. In Fig. 4b, we present the topological classification results of Hamiltonian samples in symmetry classes after incorporating the parity transformation. For example, class $\mathcal {P}$AII corresponds to Hamiltonians with $\mathcal {PT}$ symmetry. The results, summarized in Table 2, reveal clear differences from Table 1, indicating that $\mathcal {P}$ alters the topological classifications. Notably, there exists a systematic correspondence between classifications before and after applying $\mathcal {P}$, expressed by the relation (see the online supplementary material)


(2) \begin{equation*}
K^{\mathcal {P}}_{d} (s^{\mathcal {P}}) = K_{8-d} (s),
\end{equation*}

where $K_{d}(s)$ represents the topological classification of $d$-dimensional non-Hermitian systems in symmetry classes $s$, and superscript $\mathcal {P}$ denotes the classification after performing $\mathcal {P}$. For example, if $s$ corresponds to class AII then $s^{\mathcal {P}}$ corresponds to $\mathcal {P}$AII. The mapping in Equation (2) highlights that the introduction of $\mathcal {P}$ reverses the periodicity of the classifications (analogous to Bott periodicity) without introducing new topological phases. Crucially, this reversal is nontrivial: the parity transformation $\mathcal {P}$ aligns the topological classification of lower-dimensional systems with that of higher-dimensional systems. For example, 1D Hamiltonians in class $\mathcal {P}$AII ($\mathcal {PT}$ symmetry) with a real line gap exhibit the same topological classification, $\mathbb {Z}_2$ ($N_c=2$), as 7D Hamiltonians in class AII.

**Table 2.** The number of topologically different phases $N_c$ for the $d$-dimensional non-Hermitian Hamiltonians in different parity-equipped symmetry classes. Non-Hermitian topological phases are classified according to the symmetry classes, the dimension $d$ and the definition of complex-energy point (P) or line (L) gaps. The subscript of L denotes the line gap for the real or imaginary part of the complex spectrum. By $\mathcal {S}$ we denote the sublattice symmetry. The subscript of $\mathcal {S}_{\pm }$ denotes the commutation/anti-commutation relation to time-reversal $\mathcal {T}$ or particle-hole $\mathcal {C}$ symmetry. When $\mathcal {T}$ and $\mathcal {C}$ symmetries coexist, the first sign specifies the relation to $\mathcal {T}$ symmetry and the second sign to $\mathcal {C}$ symmetry. Note that dimension $d$ starts from $d=1$, because 0D Hamiltonians do not have the parity transformation. Classes A and AIII are identical to the classes in Table 1. Here, we generate the higher-dimensional Hamiltonians based on the 0D $n \times n$ random Hamiltonians (see the online supplementary material).

Symmetry class	Gap	$d=1$	$d=2$	$d=3$	$d=4$	$d=5$	$d=6$	$d=7$	$d=8$	$d=9$	$d=10$
$\mathcal {P}$ AI	P	2	1	$\frac{n}{2}+1$	1	1	1	$n+1$	2	2	1
	${\rm L}_{\rm r}$	2	2	1	$\frac{n}{2}+1$	1	1	1	$n+1$	2	2
	${\rm L}_{\rm i}$	1	$\frac{n}{2}+1$	1	1	1	$n+1$	2	2	1	$\frac{n}{2}+1$
$\mathcal {P}$ BDI	P	1	$\frac{n}{2}+1$	1	1	1	$n+1$	2	2	1	$\frac{n}{2}+1$
	${\rm L}_{\rm r}$	2	1	$\frac{n}{2}+1$	1	1	1	$n+1$	2	2	1
	${\rm L}_{\rm i}$	1	$\left(\frac{n}{4}+1\right)^2$	1	1	1	$\left(\frac{n}{2}+1\right)^2$	$2\times 2$	$2\times 2$	1	$\left(\frac{n}{4}+1\right)^2$
$\mathcal {P}$ D	P	$\frac{n}{2}+1$	1	1	1	$n+1$	2	2	1	$\frac{n}{2}+1$	1
	${\rm L}_{\rm r}$	1	$\frac{n}{2}+1$	1	1	1	$n+1$	2	2	1	$\frac{n}{2}+1$
	${\rm L}_{\rm i}$	1	$\frac{n}{2}+1$	1	1	1	$n+1$	2	2	1	$\frac{n}{2}+1$
$\mathcal {P}$ DIII	P	1	1	1	$n+1$	2	2	1	$\frac{n}{2}+1$	1	1
	${\rm L}_{\rm r}$	$\frac{n}{2}+1$	1	1	1	$n+1$	2	2	1	$\frac{n}{2}+1$	1
	${\rm L}_{\rm i}$	1	$n+1$	1	$n+1$	1	$n+1$	1	$n+1$	1	$n+1$
$\mathcal {P}$ AII	P	1	1	$n+1$	2	2	1	$\frac{n}{2}+1$	1	1	1
	${\rm L}_{\rm r}$	1	1	1	$n+1$	2	2	1	$\frac{n}{2}+1$	1	1
	${\rm L}_{\rm i}$	1	$n+1$	2	2	1	$\frac{n}{2}+1$	1	1	1	$n+1$
$\mathcal {P}$ CII	P	1	$n+1$	2	2	1	$\frac{n}{2}+1$	1	1	1	$n+1$
	${\rm L}_{\rm r}$	1	1	$n+1$	2	2	1	$\frac{n}{2}+1$	1	1	1
	${\rm L}_{\rm i}$	1	$\left(\frac{n}{2}+1\right)^2$	$2\times 2$	$2\times 2$	1	$\left(\frac{n}{4}+1\right)^2$	1	1	1	$\left(\frac{n}{2}+1\right)^2$
$\mathcal {P}$ C	P	$n+1$	2	2	1	$\frac{n}{2}+1$	1	1	1	$n+1$	2
	${\rm L}_{\rm r}$	1	$n+1$	2	2	1	$\frac{n}{2}+1$	1	1	1	$n+1$
	${\rm L}_{\rm i}$	1	$n+1$	2	2	1	$\frac{n}{2}+1$	1	1	1	$n+1$
$\mathcal {P}$ CI	P	2	2	1	$\frac{n}{2}+1$	1	1	1	$n+1$	2	2
	${\rm L}_{\rm r}$	$n+1$	2	2	1	$\frac{n}{2}+1$	1	1	1	$n+1$	2
	${\rm L}_{\rm i}$	1	$n+1$	1	$n+1$	1	$n+1$	1	$n+1$	1	$n+1$
$\mathcal {P}$ AI$^\dagger$	P	$n+1$	2	2	1	$\frac{n}{2}+1$	1	1	1	$n+1$	2
	${\rm L}_{\rm r}$	2	2	1	$\frac{n}{2}+1$	1	1	1	$n+1$	2	2
	${\rm L}_{\rm i}$	2	2	1	$\frac{n}{2}+1$	1	1	1	$n+1$	2	2
$\mathcal {P}$ BDI$^\dagger$	P	2	2	1	$\frac{n}{2}+1$	1	1	1	$n+1$	2	2
	${\rm L}_{\rm r}$	2	1	$\frac{n}{2}+1$	1	1	1	$n+1$	2	2	1
	${\rm L}_{\rm i}$	$2\times 2$	$2\times 2$	1	$\left(\frac{n}{4}+1\right)^2$	1	1	1	$\left(\frac{n}{2}+1\right)^2$	$2\times 2$	$2\times 2$
$\mathcal {P}$ DIII$^\dagger$	P	1	$\frac{n}{2}+1$	1	1	1	$n+1$	2	2	1	$\frac{n}{2}+1$
	${\rm L}_{\rm r}$	$\frac{n}{2}+1$	1	1	1	$n+1$	2	2	1	$\frac{n}{2}+1$	1
	${\rm L}_{\rm i}$	1	$n+1$	1	$n+1$	1	$n+1$	1	$n+1$	1	$n+1$
$\mathcal {P}$ AII$^\dagger$	P	$\frac{n}{2}+1$	1	1	1	$n+1$	2	2	1	$\frac{n}{2}+1$	1
	${\rm L}_{\rm r}$	1	1	1	$n+1$	2	2	1	$\frac{n}{2}+1$	1	1
	${\rm L}_{\rm i}$	1	1	1	$n+1$	2	2	1	$\frac{n}{2}+1$	1	1
$\mathcal {P}$ CII$^\dagger$	P	1	1	1	$n+1$	2	2	1	$\frac{n}{2}+1$	1	1
	${\rm L}_{\rm r}$	1	1	$n+1$	2	2	1	$\frac{n}{2}+1$	1	1	1
	${\rm L}_{\rm i}$	1	1	1	$\left(\frac{n}{2}+1\right)^2$	$2\times 2$	$2\times 2$	1	$\left(\frac{n}{4}+1\right)^2$	1	1
$\mathcal {P}$ CI$^\dagger$	P	1	$n+1$	2	2	1	$\frac{n}{2}+1$	1	1	1	$n+1$
	${\rm L}_{\rm r}$	$n+1$	2	2	1	$\frac{n}{2}+1$	1	1	1	$n+1$	2
	${\rm L}_{\rm i}$	1	$n+1$	1	$n+1$	1	$n+1$	1	$n+1$	1	$n+1$
$\mathcal {P}$ BDI, $\mathcal {S}_{++}$	P	2	1	$\frac{n}{2}+1$	1	1	1	$n+1$	2	2	1
	${\rm L}_{\rm r}$	$2\times 2$	1	$\left(\frac{n}{4}+1\right)^2$	1	1	1	$\left(\frac{n}{2}+1\right)^2$	$2\times 2$	$2\times 2$	1
	${\rm L}_{\rm i}$	$2\times 2$	1	$\left(\frac{n}{4}+1\right)^2$	1	1	1	$\left(\frac{n}{2}+1\right)^2$	$2\times 2$	$2\times 2$	1
$\mathcal {P}$ DIII, $\mathcal {S}_{--}$	P	$\frac{n}{2}+1$	1	1	1	$n+1$	2	2	1	$\frac{n}{2}+1$	1
	${\rm L}_{\rm r}$	$\left(\frac{n}{4}+1\right)^2$	1	1	1	$\left(\frac{n}{2}+1\right)^2$	$2\times 2$	$2\times 2$	1	$\left(\frac{n}{4}+1\right)^2$	1
	${\rm L}_{\rm i}$	$n+1$	1	$n+1$	1	$n+1$	1	$n+1$	1	$n+1$	1
$\mathcal {P}$ CII, $\mathcal {S}_{++}$	P	1	1	$n+1$	2	2	1	$\frac{n}{2}+1$	1	1	1
	${\rm L}_{\rm r}$	1	1	$\left(\frac{n}{2}+1\right)^2$	$2\times 2$	$2\times 2$	1	$\left(\frac{n}{4}+1\right)^2$	1	1	1
	${\rm L}_{\rm i}$	1	1	$\left(\frac{n}{2}+1\right)^2$	$2\times 2$	$2\times 2$	1	$\left(\frac{n}{4}+1\right)^2$	1	1	1
$\mathcal {P}$ CI, $\mathcal {S}_{--}$	P	$n+1$	2	2	1	$\frac{n}{2}+1$	1	1	1	$n+1$	2
	${\rm L}_{\rm r}$	$\left(\frac{n}{2}+1\right)^2$	$2\times 2$	$2\times 2$	1	$\left(\frac{n}{4}+1\right)^2$	1	1	1	$\left(\frac{n}{2}+1\right)^2$	$2\times 2$
	${\rm L}_{\rm i}$	$n+1$	1	$n+1$	1	$n+1$	1	$n+1$	1	$n+1$	1
$\mathcal {P}$ AI, $\mathcal {S}_{-}$	P	$n+1$	1	$n+1$	1	$n+1$	1	$n+1$	1	$n+1$	1
	${\rm L}_{\rm r}$	$n+1$	2	2	1	$\frac{n}{2}+1$	1	1	1	$n+1$	2
	${\rm L}_{\rm i}$	$\frac{n}{2}+1$	1	1	1	$n+1$	2	2	1	$\frac{n}{2}+1$	1
$\mathcal {P}$ BDI, $\mathcal {S}_{-+}$	P	1	$n+1$	1	$n+1$	1	$n+1$	1	$n+1$	1	$n+1$
	${\rm L}_{\rm r}$	2	2	1	$\frac{n}{2}+1$	1	1	1	$n+1$	2	2
	${\rm L}_{\rm i}$	1	$\frac{n}{2}+1$	1	1	1	$n+1$	2	2	1	$\frac{n}{2}+1$
$\mathcal {P}$ D, $\mathcal {S}_{+}$	P	$n+1$	1	$n+1$	1	$n+1$	1	$n+1$	1	$n+1$	1
	${\rm L}_{\rm r}$	2	1	$\frac{n}{2}+1$	1	1	1	$n+1$	2	2	1
	${\rm L}_{\rm i}$	2	1	$\frac{n}{2}+1$	1	1	1	$n+1$	2	2	1
$\mathcal {P}$ CII, $\mathcal {S}_{-+}$	P	1	$n+1$	1	$n+1$	1	$n+1$	1	$n+1$	1	$n+1$
	${\rm L}_{\rm r}$	1	1	1	$n+1$	2	2	1	$\frac{n}{2}+1$	1	1
	${\rm L}_{\rm i}$	1	$n+1$	2	2	1	$\frac{n}{2}+1$	1	1	1	$n+1$
$\mathcal {P}$ C, $\mathcal {S}_{+}$	P	$n+1$	1	$n+1$	1	$n+1$	1	$n+1$	1	$n+1$	1
	${\rm L}_{\rm r}$	1	1	$n+1$	2	2	1	$\frac{n}{2}+1$	1	1	1
	${\rm L}_{\rm i}$	1	1	$n+1$	2	2	1	$\frac{n}{2}+1$	1	1	1
$\mathcal {P}$ DIII, $\mathcal {S}_{++}$	P	1	1	$n+1$	2	2	1	$\frac{n}{2}+1$	1	1	1
	${\rm L}_{\rm r}$	$n+1$	1	$n+1$	1	$n+1$	1	$n+1$	1	$n+1$	1
	${\rm L}_{\rm i}$	$n+1$	1	$n+1$	1	$n+1$	1	$n+1$	1	$n+1$	1
$\mathcal {P}$ CI, $\mathcal {S}_{++}$	P	2	1	$\frac{n}{2}+1$	1	1	1	$n+1$	2	2	1
	${\rm L}_{\rm r}$	$n+1$	1	$n+1$	1	$n+1$	1	$n+1$	1	$n+1$	1
	${\rm L}_{\rm i}$	$n+1$	1	$n+1$	1	$n+1$	1	$n+1$	1	$n+1$	1
$\mathcal {P}$ AI, $\mathcal {S}_{+}$	P	$2\times 2$	1	$\left(\frac{n}{4}+1\right)^2$	1	1	1	$\left(\frac{n}{2}+1\right)^2$	$2\times 2$	$2\times 2$	1
	${\rm L}_{\rm r}$	2	1	$\frac{n}{2}+1$	1	1	1	$n+1$	2	2	1
	${\rm L}_{\rm i}$	2	1	$\frac{n}{2}+1$	1	1	1	$n+1$	2	2	1
$\mathcal {P}$ BDI, $\mathcal {S}_{+-}$	P	1	$\left(\frac{n}{4}+1\right)^2$	1	1	1	$\left(\frac{n}{2}+1\right)^2$	$2\times 2$	$2\times 2$	1	$\left(\frac{n}{4}+1\right)^2$
	${\rm L}_{\rm r}$	1	$\frac{n}{2}+1$	1	1	1	$n+1$	2	2	1	$\frac{n}{2}+1$
	${\rm L}_{\rm i}$	1	$\frac{n}{2}+1$	1	1	1	$n+1$	2	2	1	$\frac{n}{2}+1$
$\mathcal {P}$ D, $\mathcal {S}_{-}$	P	$\left(\frac{n}{4}+1\right)^2$	1	1	1	$\left(\frac{n}{2}+1\right)^2$	$2\times 2$	$2\times 2$	1	$\left(\frac{n}{4}+1\right)^2$	1
	${\rm L}_{\rm r}$	$\frac{n}{2}+1$	1	1	1	$n+1$	2	2	1	$\frac{n}{2}+1$	1
	${\rm L}_{\rm i}$	$\frac{n}{2}+1$	1	1	1	$n+1$	2	2	1	$\frac{n}{2}+1$	1
$\mathcal {P}$ DIII, $\mathcal {S}_{+-}$	P	1	1	1	$\left(\frac{n}{2}+1\right)^2$	$2\times 2$	$2\times 2$	1	$\left(\frac{n}{4}+1\right)^2$	1	1
	${\rm L}_{\rm r}$	1	1	1	$n+1$	2	2	1	$\frac{n}{2}+1$	1	1
	${\rm L}_{\rm i}$	1	1	1	$n+1$	2	2	1	$\frac{n}{2}+1$	1	1
$\mathcal {P}$ AII, $\mathcal {S}_{+}$	P	1	1	$\left(\frac{n}{2}+1\right)^2$	$2\times 2$	$2\times 2$	1	$\left(\frac{n}{4}+1\right)^2$	1	1	1
	${\rm L}_{\rm r}$	1	1	$n+1$	2	2	1	$\frac{n}{2}+1$	1	1	1
	${\rm L}_{\rm i}$	1	1	$n+1$	2	2	1	$\frac{n}{2}+1$	1	1	1
$\mathcal {P}$ CII, $\mathcal {S}_{+-}$	P	1	$\left(\frac{n}{2}+1\right)^2$	$2\times 2$	$2\times 2$	1	$\left(\frac{n}{4}+1\right)^2$	1	1	1	$\left(\frac{n}{2}+1\right)^2$
	${\rm L}_{\rm r}$	1	$n+1$	2	2	1	$\frac{n}{2}+1$	1	1	1	$n+1$
	${\rm L}_{\rm i}$	1	$n+1$	2	2	1	$\frac{n}{2}+1$	1	1	1	$n+1$
$\mathcal {P}$ C, $\mathcal {S}_{-}$	P	$\left(\frac{n}{2}+1\right)^2$	$2\times 2$	$2\times 2$	1	$\left(\frac{n}{4}+1\right)^2$	1	1	1	$\left(\frac{n}{2}+1\right)^2$	$2\times 2$
	${\rm L}_{\rm r}$	$n+1$	2	2	1	$\frac{n}{2}+1$	1	1	1	$n+1$	2
	${\rm L}_{\rm i}$	$n+1$	2	2	1	$\frac{n}{2}+1$	1	1	1	$n+1$	2
$\mathcal {P}$ CI, $\mathcal {S}_{+-}$	P	$2\times 2$	$2\times 2$	1	$\left(\frac{n}{4}+1\right)^2$	1	1	1	$\left(\frac{n}{2}+1\right)^2$	$2\times 2$	$2\times 2$
	${\rm L}_{\rm r}$	2	2	1	$\frac{n}{2}+1$	1	1	1	$n+1$	2	2
	${\rm L}_{\rm i}$	2	2	1	$\frac{n}{2}+1$	1	1	1	$n+1$	2	2

Finally, we explore the effects of open boundaries on non-Hermitian topological phases. Unlike Hermitian systems, where the conventional bulk-boundary correspondence (BBC) reliably connects bulk properties to boundary states, non-Hermitian systems often deviate from this principle due to unique features of non-Hermitian topology [29,74,85,86]. These deviations arise primarily from the nontrivial point-gap topology and manifest in several ways: (i) the non-Hermitian skin effect [29,31,67,67,87–89], in which the extended Bloch states under periodic boundary conditions (PBCs) become localized under open boundary conditions (OBCs); (ii) a mismatch between bulk and finite-size spectral gaps, such that a system lacking a line gap under PBCs (e.g. being gapless) can exhibit a line gap under OBCs [90]; (iii) shifts in the topological phase-transition boundaries, where the phase diagram of the line-gap topology changes significantly with boundary conditions [74,76]. In contrast, non-Hermitian systems with trivial point-gap topology retain the conventional BBC [75]. These open-boundary effects can be described using the generalized Brillouin zone (GBZ) formalism, where the Bloch phase factor $e^{ik}$ is replaced by a complex number $\beta$ [74,76]. The shape and size of the GBZ depend

 sensitively on the system’s parameters, providing a crucial framework for analyzing boundary effects in non-Hermitian systems.

**Figure 5.**
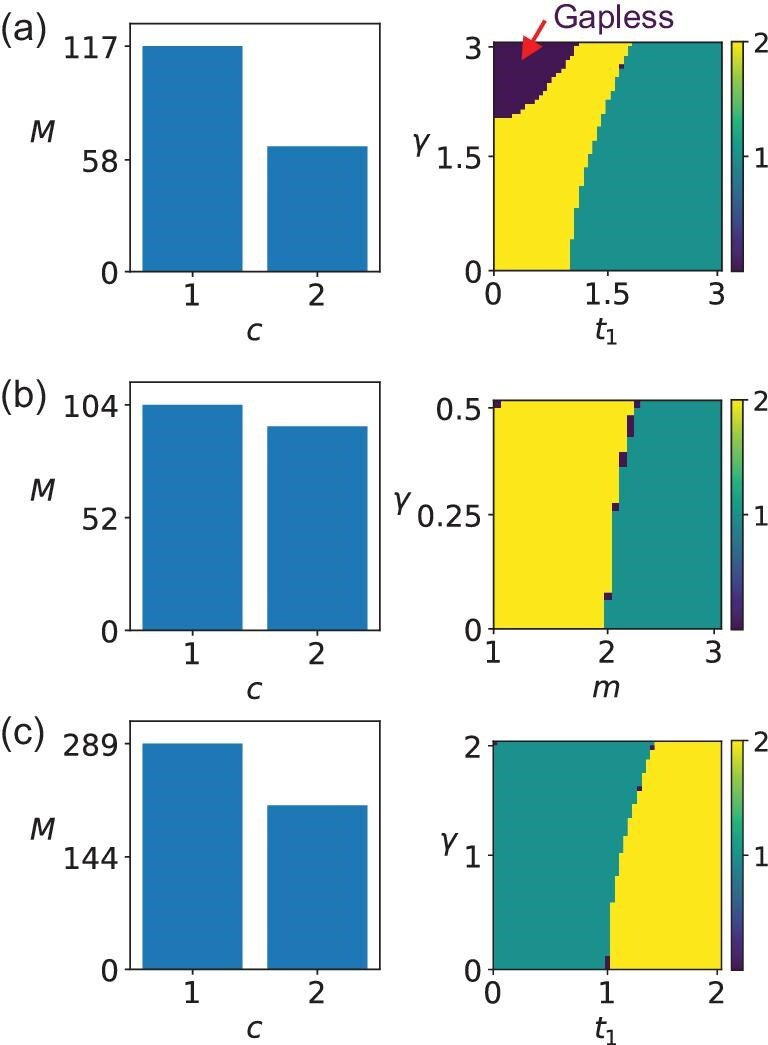
Unsupervised learning of non-Hermitian real line-gap topological phases under GBZ. The left plots represent the number of samples $M$ for the topologically distinct phases. Index $c$ denotes the label of the phases. The right plots show the topological phase diagrams obtained from the similarity between the Hamiltonian with the given parameters and Hamiltonians in $\mathcal {G}$. The different colors and labels denote the topologically distinct phases, but not the topological invariants. Note that $c=0$ denotes the gapless system. (a) One-dimensional non-Hermitian SSH system. We set $t_2=1$, $t_1\in [0,3]$, $\gamma \in [0,3]$. (b) Two-dimensional non-Hermitian Chern insulator. We set $t_x=t_y=v_x=v_y=1$, $m\in [1,3]$, $\gamma \in [0, 0.5]$. (c) Two-dimensional non-Hermitian topological Möbius insulator. We set $\kappa =0.25$, $t_2=1$, $t_1\in [0,2]$, $\gamma \in [0,2]$. Here, for obtaining the left plots, we randomly generate 500 samples for each case and filter out the gapless systems. For all cases, $E_f=0$.

Here, we demonstrate that our algorithm can operate under the GBZ to account for open-boundary effects (see the online supplementary material). We examine several non-Hermitian systems, including the 1D non-Hermitian SSH model in Fig. 5a [74], the 2D non-Hermitian Chern insulator in Fig. 5b [76] and the 2D non-Hermitian topological Möbius insulator in Fig. 5c (see the online supplementary material). By generating random parameter samples for each model, we compute their similarities and apply the clustering algorithm to determine the number of topologically distinct phases. The resulting topological phase diagrams and the number of topologically distinct phases are shown in Fig. 5. Notably, although the open-boundary effects can break the conventional BBC, they do not introduce new topological phases for real line-gap topology. As expected, the number of phases under OBCs matches that under PBCs in Fig. 3, excluding gapless phases. More details can be found in the online supplementary material.

For non-Hermitian systems exhibiting both point-gap and line-gap topology simultaneously, a key feature is the non-zero shift in the topological phase-transition point or the boundary of line-gap topological phases upon changing the boundary conditions [29,74,76,86,91]. However, not all symmetry conditions allow the coexistence of non-trivial point-gap and line-gap topology. Here, we take the line-gap topology in 1D systems as an example. According to Tables 1 and  2, we can conclude that the Hamiltonians in the following symmetry classes can exhibit a non-zero shift of the topological phase-transition point or boundary after changing boundary conditions: AI, BDI, D, DIII$^\dagger$, AIII with $\mathcal {S}_+$, A with $\mathcal {S}$, BDI with $\mathcal {S}_{++}$, DIII with $\mathcal {S}_{--}$, CII with $\mathcal {S}_{++}$, AI with $\mathcal {S}_{-}$, D with $\mathcal {S}_{+}$, C with $\mathcal {S}_{+}$, DIII with $\mathcal {S}_{++}$, CI with $\mathcal {S}_{++}$, AI with $\mathcal {S}_{+}$, BDI with $\mathcal {S}_{+-}$, D with $\mathcal {S}_{-}$, AII with $\mathcal {S}_{+}$, $\mathcal {P}$AI, $\mathcal {P}$CI, $\mathcal {P}$AI$^\dagger$, $\mathcal {P}$BDI$^\dagger$, $\mathcal {P}$BDI+$\mathcal {S}_{++}$, $\mathcal {P}$DIII with $\mathcal {S}_{--}$, $\mathcal {P}$CI with $\mathcal {S}_{--}$, $\mathcal {P}$AI with $\mathcal {S}_{-}$, $\mathcal {P}$D with $\mathcal {S}_{+}$, $\mathcal {P}$BDI with $\mathcal {S}_{+-}$, $\mathcal {P}$CII with $\mathcal {S}_{+-}$, $\mathcal {P}$C with $\mathcal {S}_{-}$, $\mathcal {P}$CI with $\mathcal {S}_{+-}$.

## CONCLUSION

To summarize, we propose an algorithm for the unsupervised topological classification of non-Hermitian topological systems under symmetries. This algorithm distinguishes topological differences among non-Hermitian Hamiltonians with symmetries, without relying on topological invariants, thereby avoiding the limitations associated with topological invariants. A topological periodic table for non-Hermitian systems across different symmetry classes is constructed in an unsupervised manner. Additionally, we incorporate unsupervised learning based on the GBZ to account for boundary effects. Our work paves the unsupervised way to identify the non-Hermitian topological phase, obtain the topological classification and guide new non-Hermitian topological devices [92–96]. Furthermore, this approach can be extended to classify non-Hermitian Hamiltonians with other symmetries, such as dissipative symmetries in the third quantization of open quantum systems [97] and global symmetries in quadratic Lindbladians [98]. Our work can also be extended to identify topological phases of interacting systems, if interacting systems can be described by effective non-interacting Hamiltonians (i.e. by mean-field theories or quasi-particle representations [99,100]).

## Supplementary Material

nwaf536_Supplemental_File

## Data Availability

All the data and code necessary for reproducing our results are publicly available on GitHub: https://github.com/longyangking/ml_topological_classification_non_hermitian.
