# Supplementary material for: Unsupervised classification of non-Hermitian topological phases under symmetries
Source: Natl Sci Rev. 2025 Nov 27;13(1):nwaf536. doi: 10.1093/nsr/nwaf536 (PMC12796827; doi:10.1093/nsr/nwaf536)
Supplement: nwaf536_Supplemental_File [file nwaf536_supplemental_file.pdf]

Supplementary Material - Unsupervised Classification of Non-Hermitian Topological Phases Under Symmetries

Yang Long[1,2,*] Haoran Xue[3], and Baile Zhang[2,4†]
[1]School of Physics Science and Engineering, Tongji University, Shanghai 200092, China
[2]Division of Physics and Applied Physics, School of Physical and Mathematical Sciences, Nanyang Technological University, 21 Nanyang Link, Singapore 637371, Singapore
[3]Department of Physics, The Chinese University of Hong Kong, Shatin, Hong Kong SAR, China
[4]Centre for Disruptive Photonic Technologies, Nanyang Technological University, Singapore 637371, Singapore

CONTENTS

Sec. I. Details about unsupervised clustering of non-Hermitian topological phases 1
A. Similarity function for the point-gap topology 2
B. Similarity function for the line-gap topology 3
C. Compact form of similarity function 4

Sec. II. Unsupervised classifications of non-Hermitian topological phases in well-known systems 4
A. 1D Hatano-Nelson system 5
B. Twisted-winding point-gap topology 5
C. 1D point-gap topological phase induced by gain and loss 6
D. 1D $\mathbb{Z}_2$ point-gap topology 6
E. 1D Non-Hermitian Su-Schrieffer-Heeger system 7
F. 1D line-gap topological phase induced by gain and loss 8
G. 1D line-gap topological phase induced by non-reciprocity 10
H. 2D non-Hermitian Chern insulator 12
I. 2D non-Hermitian topological Möbius insulator 13
J. 3D topological insulator phase induced by gain and loss 13
K. 3D topological insulator phase induced by non-reciprocity 15

Sec. III. Open-boundary effect 15
A. 1D non-Hermitian SSH system 18
B. 2D non-Hermitian Chern insulator 18
C. 2D non-Hermitian topological Möbius insulator 19

Sec. IV. Generation of symmetry-preserving Hamiltonian samples via random matrix technology 19
A. Symmetry conditions for non-Hermitian Hamiltonian 19
B. Symmetry unification 20
C. 38 symmetry classes 22
D. Random generation of non-Hermitian Hamiltonian 22
1. Complexification 23
2. Emergent symmetry 23
3. Dimension increment 23
4. Hermitian flattening 23
5. Effect of long-range hopping 24

Sec. V. Unsupervised topological classifications of non-Hermitian topological systems under symmetries 24
A. The number of phases as a function of the number of bands 24
B. Classifications of non-Hermitian topological systems in different dimensions 29

Sec. VI. Non-Hermitian symmetry classes with parity transformation 29
A. $\mathcal{PT}$ symmetry and $\mathcal{PC}$ symmetry 29
B. Generation of Hamiltonian samples 29
C. Classifications of non-Hermitian topological systems with the parity transformation in different dimensions 30
D. Corresponding relations between the topological classifications of non-Hermitian Hamiltonians with and without parity transformation 30

References 33

Sec. I. DETAILS ABOUT UNSUPERVISED CLUSTERING OF NON-HERMITIAN TOPOLOGICAL PHASES

In this section, we show details about the unsupervised clustering of non-Hermitian systems under symmetries. The process of our unsupervised learning algorithm is similar to that in the Hermitian case [1]. The main differences between non-Hermitian and Hermitian topological classifications are two points: (1) New kinds of gap-related topology: point-gap topology and imaginary line-gap topology. (2) New symmetry conditions due to the non-Hermitian nature $H^T \neq H^*$. In Fig. S1(a), we delineate the process of our algorithm in detail, as described in the main text.

In Fig. S1(b), we give a graphic diagram for demonstrating our algorithm. Our algorithm can also be understood through

* longyangphysics@tongji.edu.cn
† blzhang@ntu.edu.sg

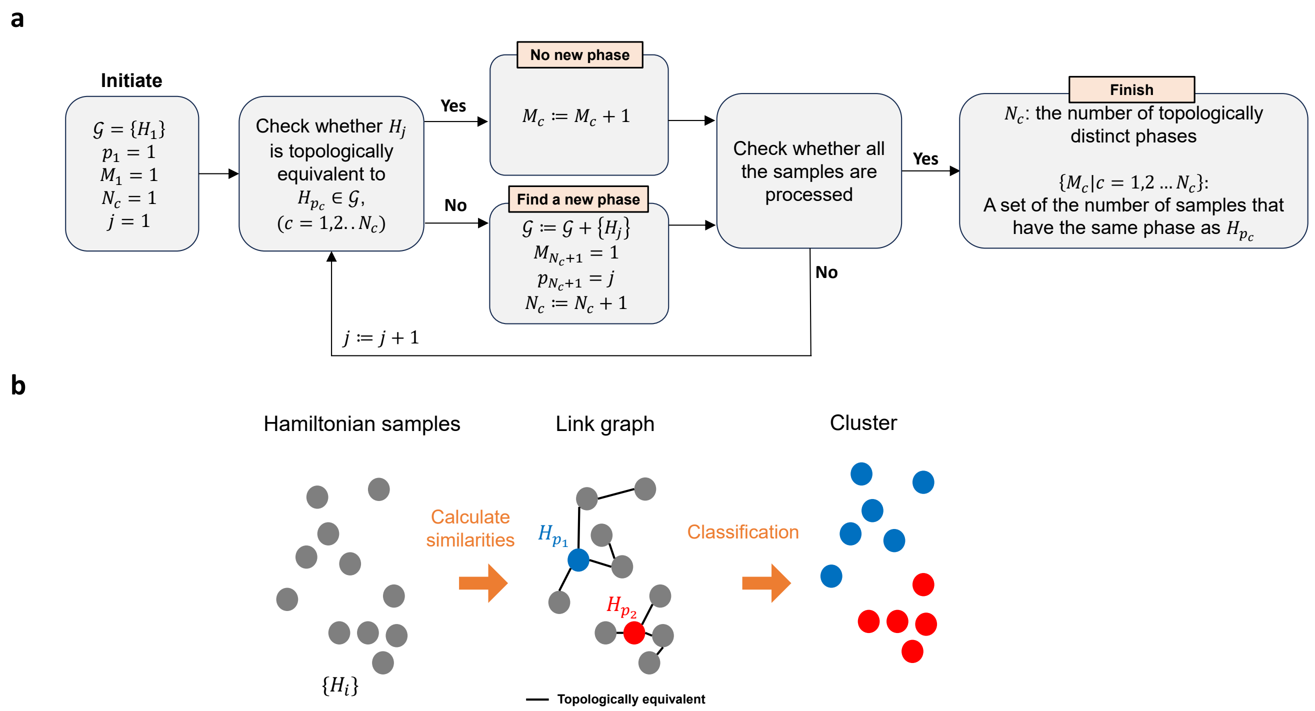

FIG. S1. The procedure of our proposed algorithm. (a) The detailed process of our algorithm. (b) A brief demonstration of our algorithm. Firstly, we calculate the similarities between Hamiltonian samples. The relations about the similarities between Hamiltonian samples can correspond to a link graph, in which the black line means $\mathcal{K}_{ij} > 1/2$, where i, j denotes H_i and H_j, respectively. Finally, we classify the samples by performing our clustering algorithm and identifying them as the phase of the group with the maximum similarity.

the following steps: 1. Generate Hamiltonian samples $\{H_i\}$ with random parameters in the regions of interest; 2. Calculate the similarities $\mathcal{K}_{ij}$ between different Hamiltonian samples H_i and H_j, based on Eq.(1) in the main text. The $\mathcal{K}_{ij}$ describe a link graph that reflects the relations between samples; 3. Perform our proposed clustering algorithm on the samples, and obtain the number of phases N_c and the number of samples for each phase $\{M_c\}$; 4. Classify an arbitrary sample H'_i by calculating its similarity with $\{H_{p_c}\}$ and labelling it as the class c corresponding to the maximum of similarity: $c = \mathrm{argmax}_{j\in\{p_c\}} \mathcal{K}_{i' p_c}$. If none of the samples in $\mathcal{G}$ is topologically equivalent to H'_i , the sample H'_i represents a new phase that is topologically different from the samples in $\mathcal{G}$.

In comparison to diffusion maps [2], our approach, as discussed in Ref. [1], offers several distinct advantages: (1) Efficient calculations. We remove the time-consuming process of calculating eigenvalues and eigenvectors of a big $N \times N$ matrix in the diffusion map when N is large, where N is the number of samples. (2) Our approach requires only a single execution. Subsequent classification of a new Hamiltonian can be promptly accomplished by comparing it with the samples in $\mathcal{G}$. In contrast, the diffusion map approach necessitates the addition of the new Hamiltonian to the original dataset and re-run the diffusion map to determine its phase. (3) The number of phases and topological phase diagram can be obtained directly in our approach without any human intervention. Yet, in diffusion map, it is necessary to initially visualize the eigenvectors and manually discern the clusters. This process becomes challenging when the dimension of the manifold space (after performing dimension reduction) exceeds 3 (i.e., when the number of eigenvalues close to 1 is greater than 3). (4) Our algorithm is hyperparameter-free, eliminating the need for any hyperparameters in machine learning. In contrast, the results obtained with the diffusion map are significantly influenced by its hyperparameters.

A. Similarity function for the point-gap topology

To identify the point-gap topology, we can construct a Hermitian Hamiltonian that has the same topological classification as the non-Hermitian system with point-gap topology. There is a mapping relation between the non-Hermitian Hamiltonian with point-gap topology and the Hermitian Hamiltonian with chiral symmetry [3, 4]. Here, we take the 1D non-Hermitian Hamiltonian as an example. For an arbitrary 1D non-Hermitian Hamiltonian H, we can construct the following 1D Hermitian Hamiltonian $\widetilde{H}$ as

$$\widetilde{H} = \begin{pmatrix} 0 & H \\ H^\dagger & 0 \end{pmatrix} \tag{S1}$$

Obviously, $\widetilde{H} = \widetilde{H}^{\dagger}$ and $\widetilde{H}$ has the chiral symmetry. Its topological invariant is the winding number W,

$$W = \frac{1}{2\pi i}\int_{-\pi}^{\pi} \partial_k \ln\det(H)dk. \tag{S2}$$

Obviously, the winding number W of Eq. S2 can have the same form as the winding number w defined in complex-energy plane [3, 5]:

$$w = \frac{1}{2\pi i}\int_{-\pi}^{\pi} \partial_k \ln\det(H)dk = W. \tag{S3}$$

If we consider the non-zero reference energy $E_f \neq 0$ (i.e., complex Fermi level) for the point-gap topology, $\widetilde{H}$ will be

$$\widetilde{H} = \begin{pmatrix} 0 & H - E_f \\ H^{\dagger} - E_f^* & 0 \end{pmatrix}. \tag{S4}$$

The corresponding winding number W is:

$$W = \frac{1}{2\pi i}\int_{-\pi}^{\pi} \partial_k \ln\det(H - E_f)dk = w, \tag{S5}$$

which is still identical to the winding number w for the point-gap topology with the reference energy E_f.

The mapping relation of Eq. S1 can be extended into the higher dimension [4, 6]. Therefore, the point-gap topology of $H(k)$ can be captured by a Hermitian Hamiltonian $\widetilde{H}$ under chiral symmetry (i.e., $\sigma_z \widetilde{H}(\boldsymbol{k})\sigma_z^{-1} = -\widetilde{H}(\boldsymbol{k})$). The symmetry conditions in H will introduce new constraints to $\widetilde{H}$. For example, if $H(\boldsymbol{k})$ has the time-reversal symmetry $\mathcal{T}_+$,

$$U_{\mathcal{T},+}H^*(\boldsymbol{k})U_{\mathcal{T},+}^{-1} = H(-\boldsymbol{k}) \tag{S6}$$

where $U_{\mathcal{T},+}^2 = \pm 1$ is the time-reversal operator, $\widetilde{H}(\boldsymbol{k})$ will have the following symmetry constraints

$$\widetilde{U}_{\mathcal{T},+}\widetilde{H}^*(\boldsymbol{k})\widetilde{U}_{\mathcal{T},+}^{\dagger} = \widetilde{H}(-\boldsymbol{k}), \quad \widetilde{U}_{\mathcal{T},+} = \begin{pmatrix} U_{\mathcal{T},+} & 0 \\ 0 & U_{\mathcal{T},+} \end{pmatrix} \tag{S7}$$

For point-gap topology, we exploit the following linear interpolation as the continuous deformation

$$\widetilde{H}_\alpha = (1-\alpha)\widetilde{H}_1 + \alpha\widetilde{H}_2 \tag{S8}$$

where $\alpha \in [0, 1]$. Due to the arbitrary eigenvalues of Hamiltonians [1], we exploit the flattened Hamiltonian $\widetilde{Q}$ to replace $\widetilde{H}$:

$$\widetilde{Q} = 1 - 2\sum_{n\in occ} |\widetilde{\psi}_{n,\boldsymbol{k}}\rangle\langle\widetilde{\varphi}_{n,\boldsymbol{k}}| \tag{S9}$$

$\widetilde{Q}$ has the discrete eigenvalues $\lambda_n \in \{-1, 1\}$. Based on $\widetilde{Q}$, the continuous deformation can be written as:

$$\widetilde{Q}_\alpha = (1-a)\widetilde{Q}_1 + a\widetilde{Q}_2. \tag{S10}$$

For detecting whether the continuous deformation will close the gap, we define the similarity function between Hamiltonian H_i and H_j as

$$\mathcal{K}_{ij} = \prod_{k\in \mathrm{BZ}}\left(1 - e^{-\frac{|v|^2}{\varepsilon^2}}\right). \tag{S11}$$

where $v = \prod_n \widetilde{\lambda}_n$, $\{\widetilde{\lambda}_n\}$ are the eigenvalues of $\widetilde{Q}_i + \widetilde{Q}_j$. Because $\widetilde{Q}$ is Hermitian, $\widetilde{\lambda}_n = \mathrm{Re}[\widetilde{\lambda}_n]$, $v = \prod_n \widetilde{\lambda}_n = \prod_n \mathrm{Re}[\widetilde{\lambda}_n]$.

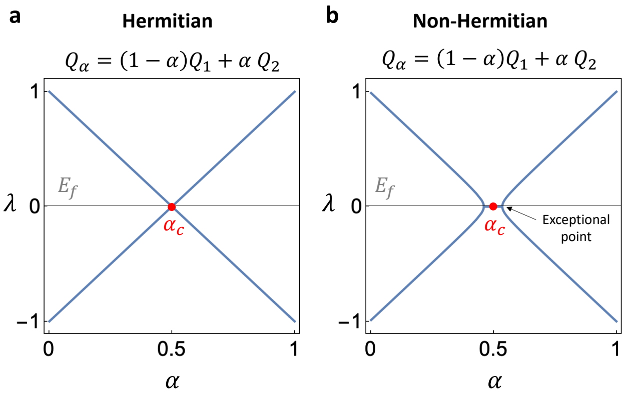

FIG. S2. The continuous deformation between two topologically distinct flattened Hamiltonians Q_1 and Q_2. They are (a) Hermitian, or (b) non-Hermitian. During the continuous deformation, $Q_\alpha = (1-\alpha)Q_1 + \alpha Q_2$ will have a crossing point at α_c with E_f. For non-Hermitian Hamiltonians, there can be a pair of exceptional points during the continuous deformation.

B. Similarity function for the line-gap topology

For the line-gap topology, a non-Hermitian Hamiltonian $H(\boldsymbol{k})$ can be continuously deformed into a Hermitian (anti-Hermitian) Hamiltonian while keeping the real (imaginary) line gap. Namely, a non-Hermitian Hamiltonian under a line gap can have the same topological classification as a Hermitian or anti-Hermitian Hamiltonian (In the following sections, we will show this point according to the concrete Hamiltonians). Although we can transform the problem of identifying non-Hermitian topological phases into a problem of identifying Hermitian topological phases, the continuous deformation between non-Hermitian and Hermitian Hamiltonians is not unique. Besides, it is not easy to realize a continuous deformation for all non-Hermitian Hamiltonians universally. Notably, without introducing a continuous deformation from a non-Hermitian Hamiltonian to a topologically equivalent Hermitian Hamiltonian, our algorithm can be applied directly to the non-Hermitian Hamiltonian.

For the line gap topology, we exploit the similar linear interpolation as the continuous deformation

$$H_\alpha = (1-\alpha)H_1 + \alpha H_2 \tag{S12}$$

where $\alpha \in [0, 1]$. For two kinds of gaps (i.e., real/imaginary line gap), the only difference for the similarity function is that the type of gap that we need to detect is distinct. This difference reflects on the occupied bands. We define the occupied bands $cocc$ as

$$\begin{aligned} L_r &: cocc = \{E_n | \mathrm{Re}[E_n - E_f] < 0\} \\ L_i &: cocc = \{E_n | \mathrm{Im}[E_n - E_f] < 0\} \end{aligned} \tag{S13}$$

where $L_{r/i}$ represents the real/imaginary line gap. The flat-

tened Hamiltonian for the line gap is given by

$$Q = 1 - 2 \sum_{n \in cocc} |\psi_{n,\mathbf{k}}\rangle\langle\varphi_{n,\mathbf{k}}|. \qquad \text{(S14)}$$

Similarly, we exploit the linear interpolation as the continuous deformation

$$Q_\alpha = (1-\alpha)Q_1 + \alpha Q_2 \qquad \text{(S15)}$$

where $\alpha \in [0,1]$. Note that Q_α is non-Hermitian, meaning that its eigenvalue can be complex $\lambda_n \in \mathbb{C}$. The crossing point in the continuous deformation will not be a single point in Hermitian cases, but can become two exceptional points with a flat band connecting them, as shown in Fig. S2. However, if two Hamiltonians are topologically distinct, the gap can still close at $\alpha_c = 1/2$ [1], although it behaves as a flat band rather than a single point. For detecting whether the continuous deformation will close the line gap, we define the similarity function between Hamiltonian H_i and H_j as

$$\mathcal{K}_{ij} = \prod_{k \in \mathrm{BZ}} \left(1 - e^{-\frac{|v|^2}{\varepsilon^2}}\right). \qquad \text{(S16)}$$

where $v = \prod_n \mathrm{Re}[\lambda_n]$, $\{\lambda_n\}$ are the eigenvalues of $Q_i + Q_j$.

Notice that we use a nearly identical similarity function for real/imaginary line-gap topology, but only *occ* is different. For the imaginary line gap, one can construct the flattened Hamiltonian as $Q_{\mathrm{im}} = i - 2i \sum_{n \in cocc} |\psi_{n,\mathbf{k}}\rangle\langle\varphi_{n,\mathbf{k}}|$, with purely imaginary eigenvalues $\lambda_{\mathrm{im},n} \in \{-i, i\}$. v in $\mathcal{K}_{ij}$ will be revised to $v = \prod_n \mathrm{Im}[\lambda_{\mathrm{im},n}]$. However, $Q_{\mathrm{im}} = iQ$ and $\mathrm{Im}[\lambda_{\mathrm{im},n}] = \mathrm{Re}[\lambda_n]$, so that Q_{im} shares the same topology as Q.

C. Compact form of similarity function

Taking the above analysis together, we can obtain the following compact form for the similarity function

$$\mathcal{K}_{ij} = \prod_{k \in \mathrm{BZ}} \left(1 - e^{-\frac{|v|^2}{\varepsilon^2}}\right). \qquad \text{(S17)}$$

The parameters will change according to the type of the gap:

1. **Point-gap topology**: $v = \prod_n \mathrm{Re}[\widetilde{\lambda}_n]$ (or $v = \prod_n \mathrm{Re}[\widetilde{\lambda}_n]$ because of Hermiticity of $\widetilde{H}$), $\{\widetilde{\lambda}_n\}$ are the eigenvalues of $\widetilde{Q}_i + \widetilde{Q}_j$, and $cocc = \{E_n\}$ denotes all bands.

2. **Real line-gap topology**: $v = \prod_n \mathrm{Re}[\lambda_n]$, $\{\lambda_n\}$ are the eigenvalues of $Q_i + Q_j$, $cocc = \{E_n | \mathrm{Re}[E_n - E_f] < 0\}$ and E_f is the complex Fermi level.

3. **Imaginary line-gap topology**: $v = \prod_n \mathrm{Re}[\lambda_n]$, $\{\lambda_n\}$ are the eigenvalues of $Q_i + Q_j$, and $cocc = \{E_n | \mathrm{Im}[E_n - E_f] < 0\}$.

The main difference between real and imaginary line-gap topology is the definition of the occupied bands *cocc*. The similarity function of Eq. S17 can also work for Hermitian Hamiltonians [1], when we only consider the real line-gap topology. In practical computations, we can calculate $v = \min_n\{|\mathrm{Re}[\lambda_n]|\}$ rather than $\prod_n \mathrm{Re}[\lambda_n]$, for numerical stability. We can also set $\varepsilon \to 0$, so that the similarity function of Eq. S17 will become a binary function: $\mathcal{K}_{ij} = 1$ when H_i and H_j are topologically identical, while $\mathcal{K}_{ij} = 0$ when H_i and H_j are topologically distinct.

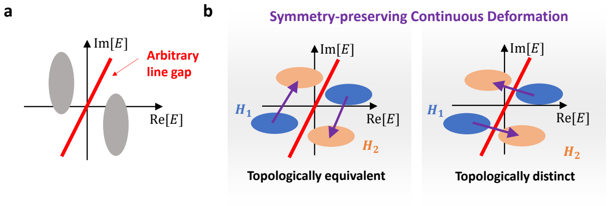

FIG. S3. (a) Arbitrary line gap. The gray regions denote the regions covered by the eigen-energies of the Hamiltonian on the complex-energy plane. (b) Symmetry-preserving continuous deformation between two Hamiltonians H_1 and H_2. When H_1 and H_2 are topologically equivalent, one can find a path to realize the continuous deformation between them without closing the gap. While H_1 and H_2 are topologically distinct, any continuous deformation between them will close the gap. The purple arrows denote the continuous deformation.

During the calculation of Eq. S17, we introduce symmetry-preserving perturbations ΔH to H, namely $H \to H + \Delta H$, for two reasons: (1) Enlarge the band gap, so that it can improve numerical accuracy; (2) Test the robustness of the crossing point at α_c in Fig. S2. For the topologically protected crossing point, any symmetry-preserving perturbation can not gap it out. In contrast, the accidental crossing point can be easily gapped out by perturbations [1].

Note that our algorithm can work for the arbitrary line gap, as shown in Fig. S3(a). Because our algorithm is based on the detection of the existence of the crossing point during the continuous deformation and this is also valid for the arbitrary line-gap topology (see Fig. S3(b)). According to the discussions in the previous parts, the only part needed to revise is *cocc*. However, the arbitrary line gap is not compatible with many symmetry conditions that can possess topological phases, e.g., the sublattice symmetry. Thus, for simplicity, we focus on the point gap and real/imaginary line gap in our work.

Sec. II. UNSUPERVISED CLASSIFICATIONS OF NON-HERMITIAN TOPOLOGICAL PHASES IN WELL-KNOWN SYSTEMS

In this section, we perform our unsupervised learning algorithm to some non-Hermitian topological systems. We focus on the non-Hermiticity-induced topological phases.

A. 1D Hatano-Nelson system

The most well-known topological system about point-gap topology induced by non-reciprocity is 1D Hatano-Nelson system [7], which has the real-space Hamiltonian

$$\mathcal{H} = \sum_n J_L c_n^\dagger c_{n+1} + J_R c_{n+1}^\dagger c_n \tag{S18}$$

where $c_n(c_n^\dagger)$ is the annihilation (creation) operator, $J_L, J_R \in \mathbb{R}$. The non-reciprocal coupling $J_L \neq J_R$ reflects the non-Hermitian nature of Hatano-Nelson system, $\mathcal{H} \neq \mathcal{H}^\dagger$. After Fourier transformation, we can obtain the Hamiltonian in the momentum space as

$$H = J_L e^{ik} + J_R e^{-ik}. \tag{S19}$$

The point-gap topology of 1D Hatano-Nelson system is represented by the closed loop of its eigen-energies on the complex-energy plane [3], $(\mathrm{Re}[E], \mathrm{Im}[E])$, as shown in Fig. S4(a). The topological property of the 1D Hatano-Nelson system is described by the winding number w on the complex-energy plane:

$$w = \frac{1}{2\pi i}\int_{-\pi}^{\pi} \partial_k \ln(E_k - E_f)dk. \tag{S20}$$

where $H|\psi_k\rangle = E_k|\psi_k\rangle$. Here, we set $E_f = 0$. Clearly, when $|J_L| > |J_R|$ ($|J_L| < |J_R|$), $w = 1$ ($w = -1$), denoting the right(left)-hand circle on the complex-energy plane, as shown in Fig. S4(b). As an important consequence of non-trivial point-gap topology in 1D system, the non-zero winding number ($w \neq 0$) of Eq. S20 is related to the appearance of non-Hermitian skin effect (NHSE) [3, 8], in which the extended modes will localize at one of ends of 1D finite chain after changing boundary condition from periodic boundary condition (PBC) to open boundary condition (OBC). The appearance of NHSE makes that the non-Hermitian topology are significantly different from the Hermitian topology, because we can not find the counterpart of NHSE in Hermitian systems. The NHSE reflects that non-Hermitian topological systems are sensitive to boundary conditions or system size [9, 10]. In the following section, we will discuss how to consider the open-boundary effect in our algorithm.

Here, we perform our unsupervised learning algorithm to detect the point-gap topology in 1D Hatano-Nelson system. After randomly generating 100 Hamiltonian samples with $J_L = 1$, $J_R \in [0, 2]$, we exploit the similarity function of Eq. S17 to calculate the similarities between samples for the point-gap topology and perform our algorithm to obtain the number of samples in different phases, as shown in Fig.2(a) of the main text. Clearly, we can see that there are $N_c = 2$ phases in Hamiltonian samples. By calculating the similarity between Hamiltonians with different J_2/J_1 and the samples in $\mathcal{G}$, we can obtain the topological phase diagram in Fig.2(a) of the main text, which is consistent with the theoretical predictions in Fig. S4(b).

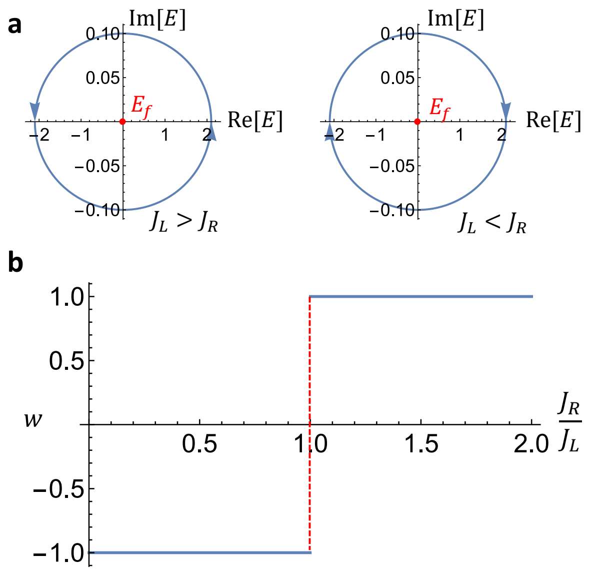

FIG. S4. Point-gap topology of 1D Hatano-Nelson system. (a) The complex-energy loops on the complex-energy plane when $J_L > J_R$ ($J_L = 1.1$, $J_R = 1$), $J_L < J_R$ ($J_L = 1$, $J_R = 1.1$), respectively. The arrows on each loop indicate the direction of increasing k which corresponds to the sign of the winding number w. (b) The winding number as a function of J_R/J_L. Here, $E_f = 0$.

B. Twisted-winding point-gap topology

Here, we discuss a single-band non-Hermitian system possessing multiple winding numbers [11, 12]. We consider a Hamiltonian having non-reciprocal next-nearest coupling [11], which reads

$$H = \kappa_1 e^{ik} + \kappa_1 e^{-ik} + \kappa_2 e^{-2ik} \tag{S21}$$

where $\kappa_1, \kappa_2 \in \mathbb{R}$. As shown in Fig. S5(a), its complex-energy spectra can form different patterns when changing κ_1 and κ_2. Importantly, it can support two closed loops with two opposite winding number [11]. The finite chain of this system can thus support two kinds of skin modes that localize at opposite ends, which are termed as "bipolar NHSE" [12]. The winding number w of Eq. S20 is strongly related to the reference energy E_f (i.e., complex "Fermi level"). As shown in Fig. S5(b), we can see that the winding number has different phase diagrams as the function of κ_2/κ_1 after setting E_f as different values.

Here, we perform our unsupervised learning algorithm to identify the point-gap topological phases in 1D system of Eq. S21. After randomly generating 100 Hamiltonian samples with $\kappa_2/\kappa_1 \in (0, 1)$, we exploit the similarity function to calculate the similarities between samples for the point-gap topology and perform our algorithm to obtain the number of samples in different phases. For $E_f = i$, its results are shown in Fig.2(b) of the main text. For $E_f = 0, -1$, we can see that there

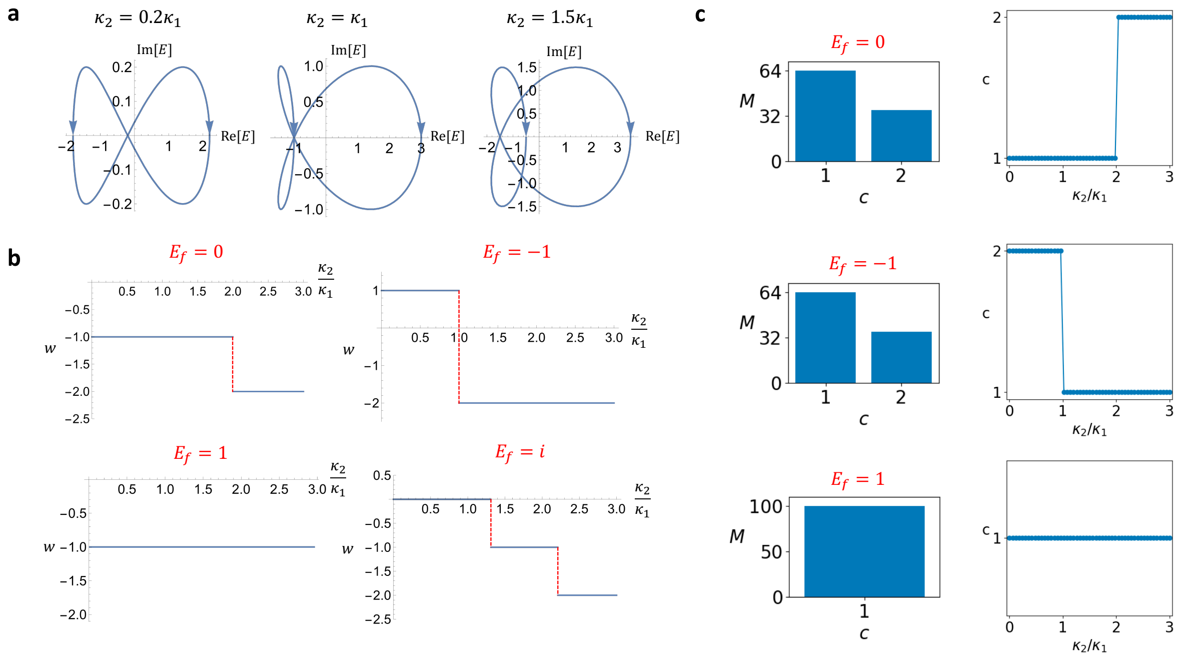

FIG. S5. Twisted-winding point-gap topology and its unsupervised learning. (a) The complex-energy loops for different parameters. Here, we demonstrate the cases with $\kappa_2 = 0.2\kappa_1$, $\kappa_2 = \kappa_1$, and $\kappa_2 = 1.5\kappa_1$, respectively. $\kappa_1 = 1.0$. The arrows on each loop indicate the direction of increasing k which corresponds to the sign of the winding number w. (b) The winding number as the function of κ_2/κ_1 with the different E_f. (c) Unsupervised learning results. Left side: The number of samples in distinct phases under different E_f. Right side: The phase digram obtained by our algorithm. The Hamiltonian samples are generated by randomly varying $\kappa_2/\kappa_1 \in (0, 1)$.

are $N_c = 2$ phases in Hamiltonian samples. For $E_f = 1$, there is only $N_c = 1$ phase in Hamiltonian samples. By calculating the similarity between Hamiltonians with different κ_2/κ_1 and the samples in $\mathcal{G}$, we can obtain the topological phase diagram in Fig. S5(c), which is consistent with the theoretical predictions in Fig. S5(b). This case shows that our algorithm can identify point-gap topological phases with a changing E_f.

C. 1D point-gap topological phase induced by gain and loss

In previous cases, the point-gap topology is mainly induced by non-reciprocal couplings. However, nonreciprocal coupling is not a necessary condition to have non-trivial point-gap topology. It has been shown that gain and loss can also induce non-trivial point-gap topology without non-reciprocal couplings [13]. Here, we discuss an 1D non-Hermitian system with the following Hamiltonian

$$H(k) = (t_1 + t_2\cos(k))\sigma_x + t_2\sin(k)\sigma_y + \lambda\sin(k)\sigma_z + (\mu + i\gamma)\sigma_z \tag{S22}$$

which preserves parity-particle-hole ($\mathcal{PC}_-$) symmetry [13], namely, $U_{\mathcal{PC},-}H(k)^T U^{-1}_{\mathcal{PC},-} = -H(k)$, $U^*_{\mathcal{PC},-}U_{\mathcal{PC},-} = -1$. The $\mathcal{PC}_-$ symmetry can introduce the non-trivial point-gap topology [13]. We will discuss $\mathcal{PC}$ symmetry in the following sections.

Here, we perform our unsupervised learning algorithm to identify the point-gap topology in 1D system of Eq. S22. After randomly generating 100 Hamiltonian samples with $\tau \in (0, 1)$ (for simplicity, we fix $t_1 = 2$, $t_2 = 1$, $\mu = 1$, $\gamma = 2$), we exploit the similarity function to calculate the similarities between samples for the point-gap topology and perform our algorithm to obtain the number of samples in different phases. For $E_f = 0$, its results are shown in Fig.2(c) of the main text. For $E_f = 1, 1.5i$, we can see that there are $N_c = 2$ phases in Hamiltonian samples. For $E_f = 2 + i$, there is only $N_c = 1$ phase in Hamiltonian samples. By calculating the similarity between Hamiltonians with different τ and the samples in $\mathcal{G}$, we can obtain the topological phase diagram in Fig. S6(c), which is consistent with the theoretical predictions in Fig. S6(b).

D. 1D $\mathbb{Z}_2$ point-gap topology

Here, we consider a 1D non-Hermitian in the symmetry class AII†. According to our numerical results (Table I of the main text), the point-gap topological classifications of 1D Hamiltonians of AII† belong to the group $\mathbb{Z}_2$. For the symmetry class AII†, the Hamiltonian H has time-reversal symmetry $\mathcal{T}_-$, $U_{\mathcal{T}_-}H^T(k)U^{(}_{\mathcal{T}_-} - 1) = H(-k)$, $U_{\mathcal{T}_-}U^*_{\mathcal{T}_-} = -1$, where $U_{\mathcal{T}_-}$ is a unitary operator. We consider the following prototypical Hamiltonian that is composed of Hatanano-Nelson model and its time-reversed partner [14]:

$$H(k) = 2t\cos(k) + 2\Delta\sin(k)\sigma_x + 2ig\sin(k)\sigma_z \tag{S23}$$

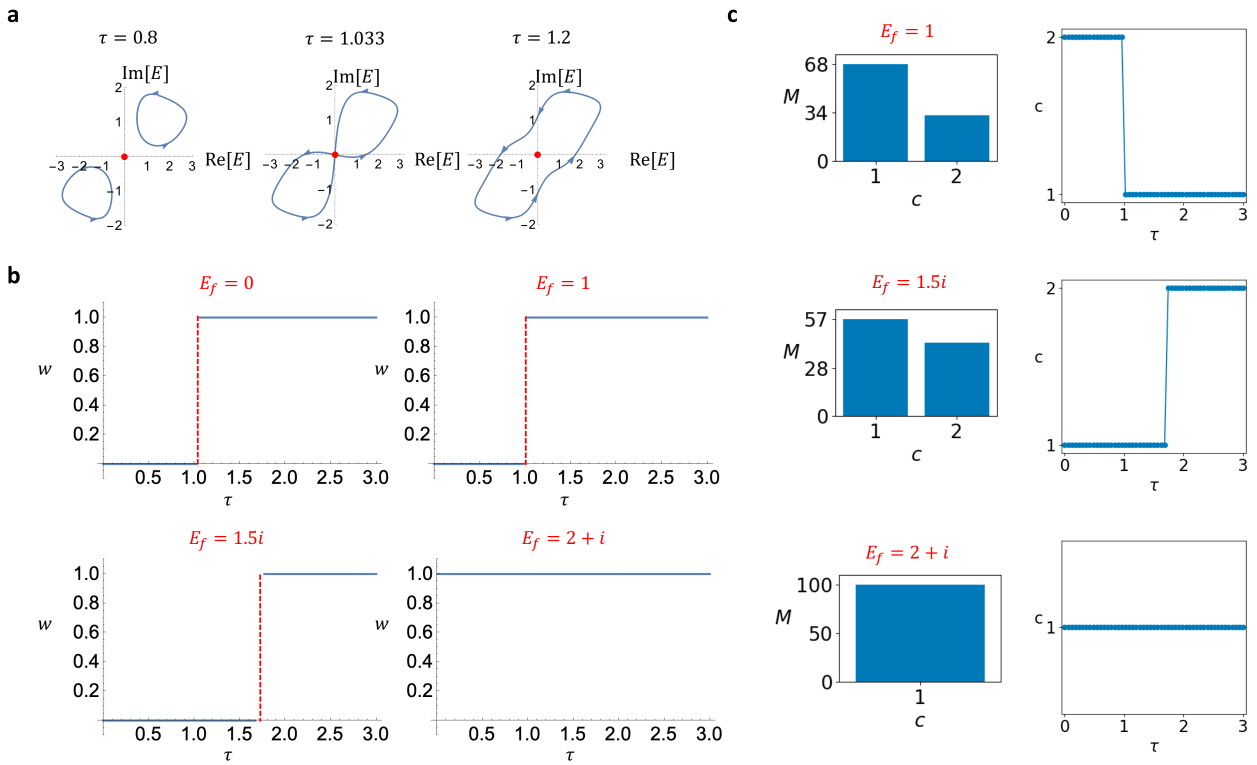

FIG. S6. Point-gap topology induced by the gain and loss and its unsupervised learning. (a) The complex-energy loops when $\tau = 0.8$, $\tau = 1.033$, and $\tau = 1.2$, respectively. Here, we set $t_1 = 2$, $t_2 = 1$, $\mu = 1$, $\gamma = 2$. (b) The winding number w as a function of τ with $E_f = 0$, 1, $1.5i$ and $2 + i$. (c) Unsupervised learning results. Left side: The number of samples in distinct phases when $E_f = 1$, $1.5i$ and $2 + i$. Right side: The phase diagram obtained by our algorithm. The Hamiltonian samples are generated by randomly varying $\tau \in (0, 3)$.

Where t, Δ and $g \in \mathbb{R}$. Clearly, it respects the time-reversal symmetry with $U_{\mathcal{T}_-} = i\sigma_y$. As shown in Fig. S7(a), the time-reversal symmetry makes the winding number keep zero, but instead, it can have a $\mathbb{Z}_2$ invariant to describe its point-gap topology. This is similar to the quantum spin Hall insulator composed of a pair of time-reversed quantum Hall insulators, in which the integer Chern number vanishes but a $\mathbb{Z}_2$ topological invariant becomes nontrivial.

For the point-gap topology, the $\mathbb{Z}_2$ topological invariant $\nu(E_f) \in 0, 1$ for a complex Fermi level $E_f \in \mathbb{C}$ is given by [14]:

$$(-1)^{\nu(E_f)} = \text{sign}\left[\frac{\text{Pf}[(H(\pi) - E_f)U_{\mathcal{T}_-}]}{\text{Pf}[(H(0) - E_f)U_{\mathcal{T}_-}]} \times \exp\left[-\frac{1}{2}\int_{k=0}^{k=\pi} \partial_k \log\det\left[(H(k) - E_f)U_{\mathcal{T}_-}\right] dk\right]\right] \tag{S24}$$

where Pf[·] is the Pfaffian and sign[·] is the sign function. As a result of non-zero $\nu(E_f)$, finite systems with open boundaries host localized modes at both ends, unlike the $\mathbb{Z}$-type skin effect where modes localize at only one end. By varying g and Δ, we can obtain the topological phase diagram as shown in Fig. S7(b).

Here, we perform our unsupervised learning algorithm to detect the point-gap topology in 1D non-Hermitian of AII†. Here, we take $E_f = 0.2i$. After randomly generating 100 Hamiltonian samples with $t = 1$, $\Delta, g \in [-0.3, 0.3]$, we exploit the similarity function of Eq.1 of the main text (or, Eq. S17) to calculate the similarities between samples for the point-gap topology and perform our algorithm to obtain the number of samples in different phases, as shown in Fig. S7(c). Clearly, we can see that there are $N_c = 2$ phases in Hamiltonian samples. By calculating the similarity between Hamiltonians with different Δ, g, and the samples in $\mathcal{G}$, we can obtain the topological phase diagram in Fig. S7(d), which is consistent with the theoretical prediction based on the $\mathbb{Z}_2$ invariant.

E. 1D Non-Hermitian Su-Schrieffer-Heeger system

We now discuss the real line-gap topology of 1D non-Hermitian systems. We first take 1D non-Hermitian Su-Schrieffer-Heeger (SSH) system as an example. The 1D non-Hermitian SSH system has the Hamiltonian [15, 16]

$$H(\boldsymbol{k}) = d_x\sigma_x + \left(d_y - i\frac{\gamma}{2}\right)\sigma_y \tag{S25}$$

where $d_x = t_1 + t_2\cos(k)$, $d_y = t_2\sin(k)$, and σ_i is the Pauli matrix. The non-Hermitian SSH system has the sublattice sym-

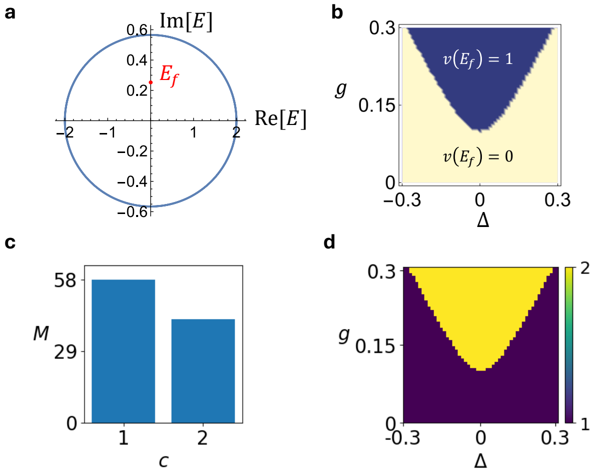

FIG. S7. $\mathbb{Z}_2$ point-gap topology and its unsupervised learning. (a) The complex-energy loops on the complex-energy plane when $t = 1$, $g = 0.3$, and $\Delta = 0.1$. Here, we set $E_f = 0.2i$, as denoted in a red point. (b) The theoretical prediction of the topological phase diagram as a function of g and Δ. Here, $t = 1$. (c) Unsupervised learning results. (d) The phase diagram obtained by our algorithm. The Hamiltonian samples are generated by randomly varying $g \in [0, 0.3]$ and $\Delta \in [-0.3, 0.3]$. Clearly, the obtained phase diagram is consistent with the theoretical prediction based on the $\mathbb{Z}_2$ invariant.

metry, $\sigma_z H(k)\sigma_z^{-1} = -H(k)$. As shown in Fig. S8(a), the non-Hermitian SSH system can not only have two gapped phases, but also have an extra gapless phase for certain regions of parameters. For the real line-gap topology, the symmetry conditions of Eq. S25 belong to the class A with sublattice symmetry, meaning that topological property can be described by an integer topological invariant [4]: the winding number W

$$W = \oint \frac{dk}{4\pi i} \mathrm{Tr}\left[\sigma_z H^{-1}(k) \frac{dH(k)}{dk}\right]. \tag{S26}$$

The winding number W of Eq. S26 can be directly associated with the number of the topological zero-energy edge mode. After filtering the gapless phases out, we can obtain the phase diagram in Fig. S25(b) for the real line-gap topology. Note that the phase diagram in Fig. S25(b) is different from the previous works [4, 15]. Because we only focus on the real line-gap topology here, we ignore the regions of parameters corresponding to the real line gap closing.

Here, we perform our unsupervised learning algorithm to detect the real line-gap topology in 1D non-Hermitian SSH system of Eq. S25. After randomly generating 500 Hamiltonian samples with $\gamma \in (0, 3)$ and $t_1 \in (-3, 3)$ (we fix $t_2 = 1$), we exploit the similarity function to calculate the similarities between samples for the point-gap topology and perform our algorithm to obtain the number of samples in different phases. The unsupervised learning results are shown in Fig.3(a) of the main text. By calculating the similarity between Hamiltonians with different γ and t_1 and the samples in $\mathcal{G}$, we can obtain the topological phase diagram in Fig.3(a) of the main text, which is consistent with the theoretical predictions in Fig. S8(b).

Besides the real line-gap topology, we can notice that the non-Hermitian SSH system can also possess the point-gap topology. As shown in Fig. S8(c), the complex-energy spectra can form the closed loops with non-trivial windings. Clearly, the non-Hermitian SSH system of Eq. S25 has two types of gaps simultaneously. From Fig. S8(c), we can see that when the real line-gap topological phase transition happens, the point-gap topological phase transition can also happens. The coexistence of point-gap topology and line-gap topology can lead to a difference in the phase digram under different boundary conditions [15]. We will discuss this point in the following sections and show that our algorithm can also work after considering the open-boundary effect.

Here, we perform our unsupervised learning algorithm to identify the point-gap topology in 1D non-Hermitian SSH system of Eq. S25. After randomly generating 500 Hamiltonian samples with $\gamma \in (0, 3)$ (for simplicity, we fix $t_2 = 1$ and $t_1 = 2$), we exploit the similarity function to calculate the similarities between samples for the point-gap topology and perform our algorithm to obtain the number of samples in different phases. The unsupervised learning results are shown in Fig. S8(d). By calculating the similarity between Hamiltonians with different γ and the samples in $\mathcal{G}$, we can obtain the topological phase diagrams, which are consistent with the theoretical predictions.

F. 1D line-gap topological phase induced by gain and loss

Here, we consider 1D topological system with real line-gap topology solely induced by on-site gain and loss. The gain-and-loss-induced topological phases have been widely discussed and shown a great potential with applications, e.g., topological laser. It has been shown that the onsite gain and loss can open a real line gap and induce a topological phase transition [17, 18]. Here, we consider the following Hamiltonian, which reads [17]

$$H = \begin{pmatrix} ig_1 & \kappa & 0 & \kappa e^{-ik} \\ \kappa & -ig_2 & \kappa & 0 \\ 0 & \kappa & -ig_1 & \kappa \\ \kappa e^{ik} & 0 & \kappa & ig_2 \end{pmatrix} \tag{S27}$$

where $\kappa, g_1, g_2 \in \mathbb{R}$. Clearly, when $g_1 = g_2 = 0$, the Hamiltonian of Eq. S27 represents a gapless Hermitian system. When $g_1 \neq 0$ and $g_2 \neq 0$, it can open a gap, as shown in Fig. S9(a). The Hamiltonian of Eq. S27 satisfies the particle-hole symmetry $U_{C,-}H^*(k)U_{C,-}^{-1} = -H(-k)$, where $U_{C,-} = \sigma_0 \otimes \sigma_z$ $U_{C,-}^2 = 1$ [17]. The line-gap topology of Eq. S27 is captured by a biorthogonal polarization [19]

$$p^{ab} = -\frac{1}{2\pi}\int_{-\pi}^{\pi} \mathrm{Tr}[\mathcal{A}^{ab}(k)]dk \tag{S28}$$

where $[\mathcal{A}^{ab}(k)]_{mn} = -i\langle u_m^a(k)|\partial_k|u_n^b(k)\rangle$ is the biorthogonal non-Abelian Berry connection, $a, b = L, R$ denote left and right eigenvectors, $m, n = 1, 2$ denotes the two occupied bands. Because of the complex nature of p^{ab}, a real-value polarization $p = (p^{LR} + p^{RL})/2$ is exploited to represent the real line-gap topological phase. When $g_1 = g_2$, $p = 1/2$ refers

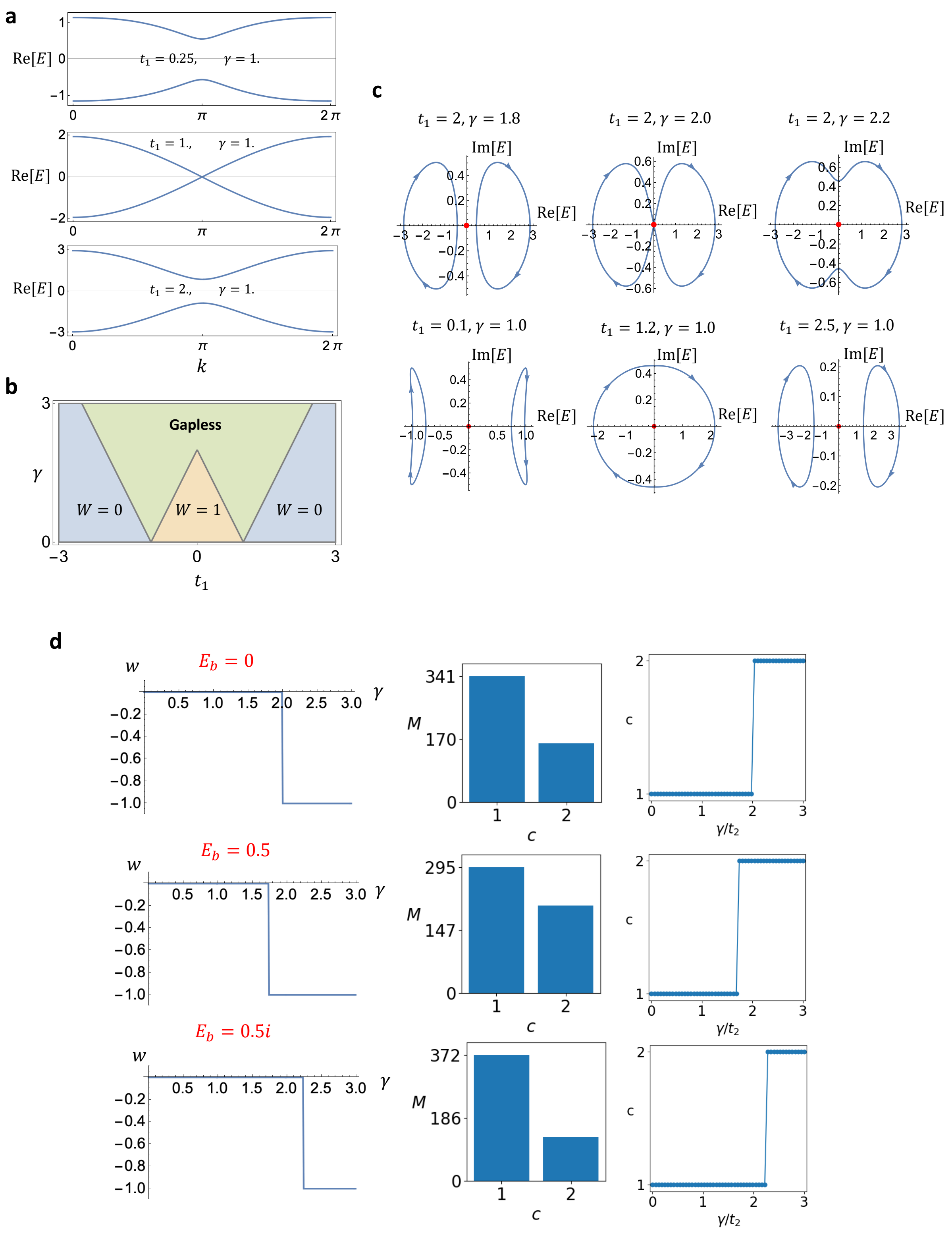

FIG. S8. Topological properties and unsupervised learning of 1D non-Hermitian SSH system. (a) The bandstructures with different parameters. Here, $t_2 = 1$. (b) The phase diagram of 1D non-Hermitian SSH system. (c) The complex-energy loops with different parameters. (d) Theoretical analysis and unsupervised learning results for point-gap topology. Left side: the winding number as a function of γ when $E_f = 0$, 0.5, and $0.5i$. Center: the number of samples in distinct phases. Right side: the phase digram obtained by our algorithm. The Hamiltonian samples are generated by randomly varying $\gamma \in (0, 3)$. Here, we set $t_1 = 2$ and $t_2 = 1$

to the topological phase, while when $g_1 = -g_2$, $p = 0$ refers to the trivial phase. Note that for certain parameters, the Hamiltonian of Eq. S27 is gapless.

As we mentioned in the previous sections, for the line-gap topology, a non-Hermitian Hamiltonian $H(\boldsymbol{k})$ can be continuously deformed into a Hermitian (anti-Hermitian) Hamiltonian while keeping the real (imaginary) line gap. Here, we take the Hamiltonian of Eq. S27 as an example. Firstly, we consider the following Hermitian Hamiltonian $\widetilde{H}$

$$\widetilde{H} = \begin{pmatrix} 0 & \kappa & 0 & (\kappa+\Delta\kappa)e^{-ik} \\ \kappa & 0 & \kappa+\Delta\kappa & 0 \\ 0 & \kappa+\Delta\kappa & 0 & \kappa \\ (\kappa+\Delta\kappa)e^{ik} & 0 & \kappa & 0 \end{pmatrix} \tag{S29}$$

which describes a Hermitian SSH system in a superlattice (doubled unit cells). Clearly, $\widetilde{H}$ also satisfies the particle-hole-like symmetry $U_C\widetilde{H}^*(k)U_C^{-1} = -\widetilde{H}(-k)$. When $\Delta\kappa \neq 0$, $\widetilde{H}$ will have a gap, as shown in Fig. S9. Importantly, according to the topological properties of Hermitian SSH system, it has distinct topological phases when $\Delta\kappa > 0$ or $\Delta\kappa < 0$. Here, we denote H with different parameters as H_1 and H_2, as shown in Fig. S9(a), and $\widetilde{H}$ with different parameters as $\widetilde{H}_1$ and $\widetilde{H}_2$, as shown in Fig. S9(b). We exploit the following linear interpolation as the continuous deformation

$$H_\alpha = (1-\alpha)H + \alpha\widetilde{H} \tag{S30}$$

where $\alpha \in [0,1]$. As shown in Fig. S9, the continuous deformation between H_1 and $\widetilde{H}_1$ does not close the real line gap, meaning that they are in the identical phase, while the continuous deformation between H_1 and $\widetilde{H}_2$ closes the real line gap (Here, for simplicity, we don't show the robustness of the crossing point after introducing perturbations), meaning that they are in distinct phases. Similarly, the continuous deformation between H_2 and $\widetilde{H}_2$ does not close the real line gap, meaning that they are in the identical phase, while the continuous deformation between H_2 and $\widetilde{H}_1$ closes the real line gap, meaning that they are in the distinct phases. Considering the fact that $\widetilde{H}_1$ and $\widetilde{H}_2$ are topologically distinct, we can thus conclude that H_1 and H_2 are topologically distinct. Based on this continuous deformation or the topological properties of Eq. S27, we can obtain the phase diagram for the real line-gap topology, as shown in Fig. S9(d). Note that the system of Eq. S27 has trivial point-gap topology.

Although finding a topologically equivalent Hermitian Hamiltonian is an approach for identifying topological phases, there is no universal way to construct such a Hamiltonian. Fortunately, our algorithm does not rely on this deformation an can apply the non-Hermitian Hamiltonian directly. We perform our unsupervised learning algorithm to detect the real line-gap topology in 1D system of Eq. S27. After randomly generating 500 Hamiltonian samples with $g_1 \in (-3,3)$ and $g_2 \in (-3,3)$ (we fix $\kappa = 1$), we exploit the similarity function to calculate the similarities between samples for the real line-gap topology and perform our algorithm to obtain the number of samples in different phases. The unsupervised learning results are shown in Fig.3(b) of the main text. By calculating the similarity between Hamiltonians with different g_1 and g_2 and the samples in $\mathcal{G}$, we can obtain the topological phase diagrams, which are consistent with the theoretical predictions in Fig. S9(d).

G. 1D line-gap topological phase induced by non-reciprocity

Here, we consider 1D topological system with real line-gap topology solely induced by non-reciprocal couplings [20]. The non-Hermitian Hamiltonian reads

$$H = M^{-1}H_0 = t_0\begin{pmatrix} 0 & \varepsilon & \varepsilon e^{-ik} \\ \frac{1}{\varepsilon} & 0 & \frac{1}{\varepsilon} \\ \varepsilon e^{ik} & \varepsilon & 0 \end{pmatrix} \tag{S31}$$

where

$$H_0 = t_0\begin{pmatrix} 0 & 1 & e^{-ik} \\ 1 & 0 & 1 \\ e^{ik} & 1 & 0 \end{pmatrix}, \quad M = \mathrm{diag}[\varepsilon^{-1}, \varepsilon, \varepsilon^{-1}] \tag{S32}$$

The Hamiltonian of Eq. S31 satisfies the mirror symmetry $\mathcal{M}_xH(k)\mathcal{M}_x^{-1} = H(-k)$ ($\mathcal{M}_x^2 = 1$) and time-reversal symmetry. When $\varepsilon = 1$, $H = H_0$, corresponding to a gapless Hermitian system. When $\varepsilon \neq 1$, H opens two gaps. Importantly, $\varepsilon > 1$ and $\varepsilon < 1$ correspond to topologically distinct phases [20], which can be described by the Zak phase θ_{Zak}:

$$\theta_{\mathrm{Zak}} = -i\int_{-\pi}^{\pi}\langle u_n^L(k)|\partial_k|u_n^R(k)\rangle dk, \tag{S33}$$

where $|u_n^{L/R}(k)\rangle$ is the left/right eigenvector of the n-th band. When $\varepsilon < 1$, the lowest band has zero Zak phase $\theta_{\mathrm{Zak}} = 0$, referring to the trivial phase, while when $\varepsilon > 1$, the lowest band has non-zero Zak phase $\theta_{\mathrm{Zak}} = \pi$, referring to the topological phase.

Firstly, we discuss how to continuously deform H of Eq. S31 to a Hermitian Hamiltonian. We consider the following Hermitian Hamiltonian $\widetilde{H}$

$$\widetilde{H} = \begin{pmatrix} 0 & t_0+\Delta t & t_0e^{-ik} \\ t_0+\Delta t & 0 & t_0+\Delta t \\ t_0e^{ik} & t_0+\Delta t & 0 \end{pmatrix}. \tag{S34}$$

Clearly, $\widetilde{H}$ satisfies the mirror symmetry $\mathcal{M}_x\widetilde{H}(k)\mathcal{M}_x^{-1} = \widetilde{H}(-k)$ ($\mathcal{M}_x^2 = 1$) and time-reversal symmetry. When $\Delta t \neq 0$, $\widetilde{H}$ can open gaps, as shown in Fig. S10(b). Importantly, according to the topological properties protected by the mirror and time-reversal symmetry, it has distinct topological phases when $\Delta t > 0$ or $\Delta t < 0$. Here, we denote H with different parameters as H_1 and H_2, as shown in Fig. S10(a), and $\widetilde{H}$ with different parameters as $\widetilde{H}_1$ and $\widetilde{H}_2$, as shown in Fig. S10(b). We exploit the following linear interpolation as the continuous deformation

$$H_\alpha = (1-\alpha)H + \alpha\widetilde{H} \tag{S35}$$

where $\alpha \in [0,1]$. As shown in Fig. S10, the continuous deformation between H_1 and $\widetilde{H}_1$ does not close the real line gap,

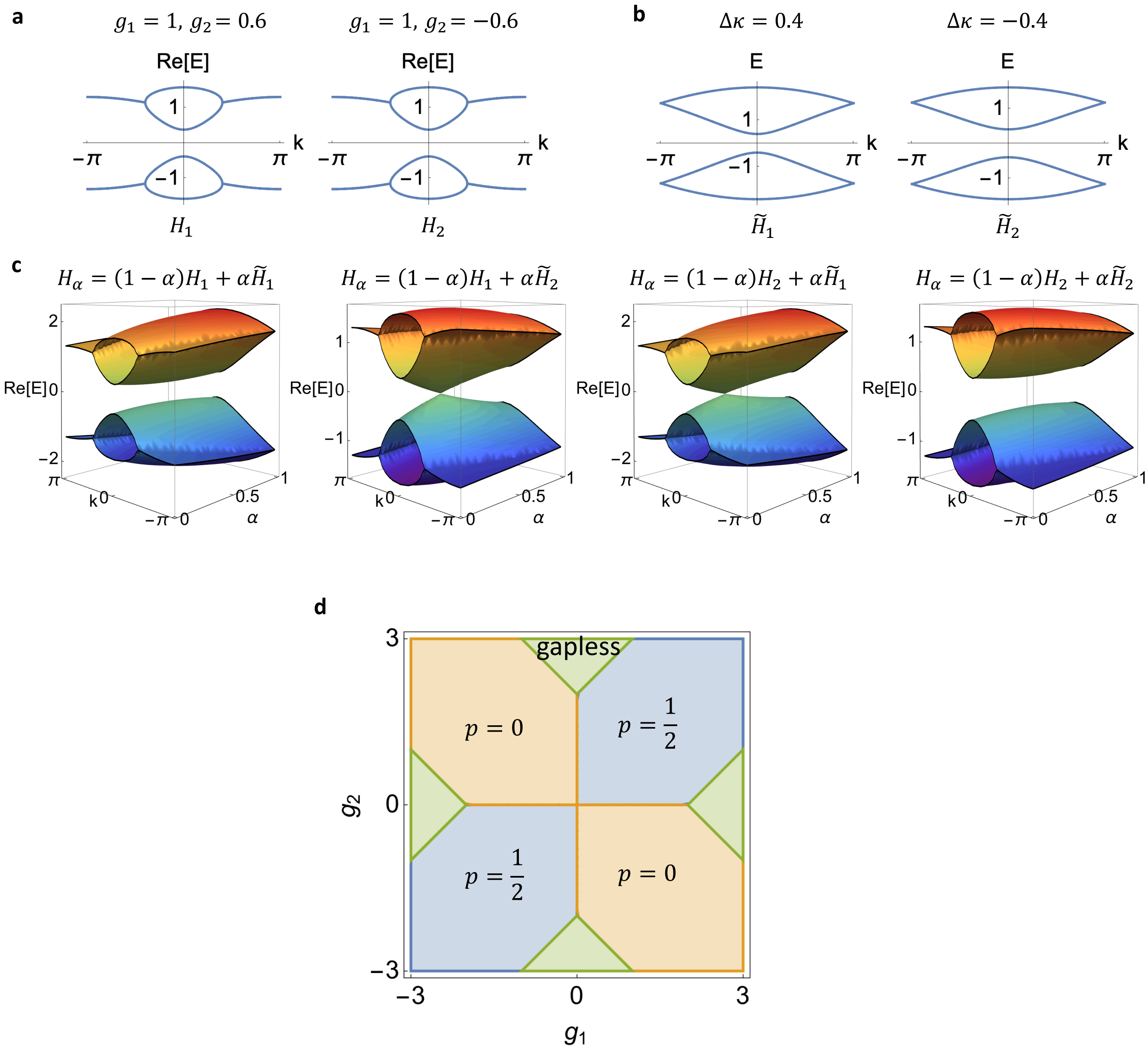

FIG. S9. Topological properties of 1D line-gap topological systems induced by gain and loss. (a) The bandstructures of H of Eq. S27 with different parameters. Here, $\kappa = 1$. (b) The bandstructures of $\mathcal{H}$ of Eq. S29 with different parameters. $\kappa = 1$. (c) The continuous deformations between $H_{1/2}$ and $\mathcal{H}_{1/2}$. (d) The phase diagram of 1D line-gap topological systems of Eq. S27. Here, $\kappa = 1$. The green regions denote the gapless phases.

meaning that they are in the identical phase, while the continuous deformation between H_1 and $\widetilde{H}_2$ closes the real line gap (Here, for simplicity, we don't show the robustness of the crossing point after introducing perturbations), meaning that they are in the distinct phases. Similarly, the continuous deformation between H_2 and $\widetilde{H}_2$ does not close the real line gap, meaning that they are in the identical phase, while the continuous deformation between H_2 and $\widetilde{H}_1$ closes the real line gap, meaning that they are in the distinct phases. Considering the fact that $\widetilde{H}_1$ and $\widetilde{H}_2$ are topologically distinct, we can thus conclude that H_1 and H_2 are topologically distinct. Based on this continuous deformation or the topological properties of Eq. S31, we can obtain the phase diagram for the real line-gap topology, as shown in Fig. S10(d). Note that the system of

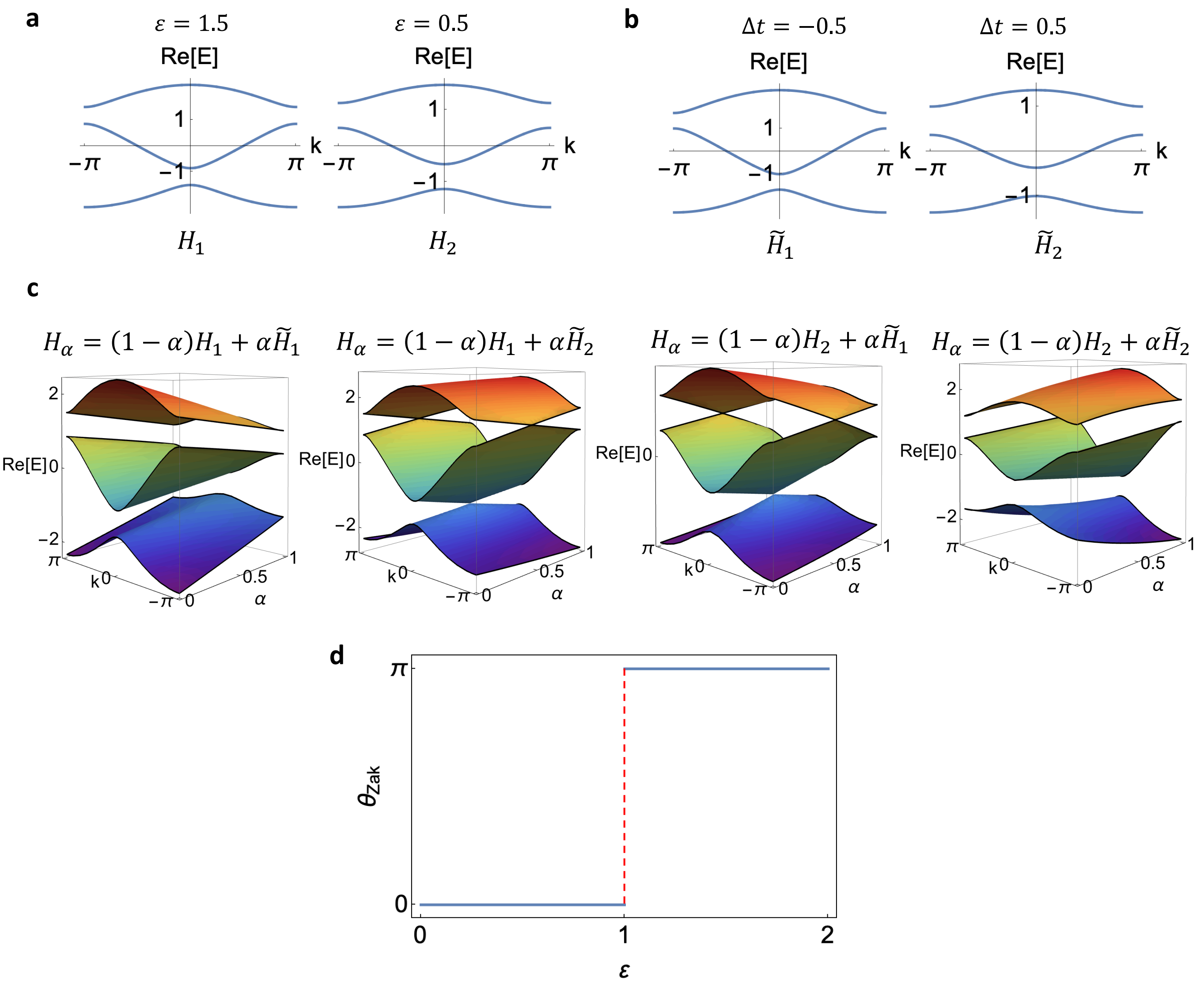

FIG. S10. Topological properties of 1D line-gap topological systems induced by non-reciprocal couplings. (a) The bandstructures of H of Eq. S31 with different parameters. Here, $t_0 = 1$. (b) The bandstructures of $\mathcal{H}$ of Eq. S34 with different parameters. $t_0 = 1$. (c) The continuous deformations between $H_{1/2}$ and $\mathcal{H}_{1/2}$. (d) The phase diagram of 1D line-gap topological systems of Eq. S31. Here, $t_0 = 1$.

Eq. S31 has trivial point-gap topology, because eigenenergies are always real [20].

Here, we perform our unsupervised learning algorithm to detect the real line-gap topology in 1D system of Eq. S31. After randomly generating 500 Hamiltonian samples with $\varepsilon \in (0, 2)$ (we fix $t_0 = 1$), we exploit the similarity function to calculate the similarities between samples for the real line-gap topology and perform our algorithm to obtain the number of samples in different phases. The unsupervised learning results are shown in Fig.3(c) of the main text. By calculating the similarity between Hamiltonians with different ε and the samples in $\mathcal{G}$, we can obtain the topological phase diagram, which are consistent with the theoretical prediction in Fig. S10(d).

H. 2D non-Hermitian Chern insulator

Here, we discuss the real line-gap topology in the non-Hermitian Chern insulator. The 2D non-Hermitian Chern insulator has the Hamiltonian [21]:

$$H = (v_x \sin(k_x) + i\gamma_x)\sigma_x + (v_y \sin(k_y) + i\gamma_y)\sigma_y + (m - t_x \cos(k_x) - t_y \cos(k_y) + i\gamma_z)\sigma_z \quad \text{(S36)}$$

where $m, t_{x,y}, \gamma_{x,y,z} \in \mathbb{R}$. When $t_{x,y,z} \neq 0$, the eigenvalues of Hamiltonian of Eq. S36 are $E_\pm(\boldsymbol{k}) = \pm\sqrt{\sum_{j=x,y,z}(h_j^2 - \gamma_j^2 + 2i\gamma_j h_j)}$ where $(h_x, h_y, h_z) = (v_x \sin(k_x), v_y \sin(k_y), m - \sum_j t_j \cos(k_j))$. As shown in Fig. S11(a), when $m > m_+$ or $m < m_-$, where

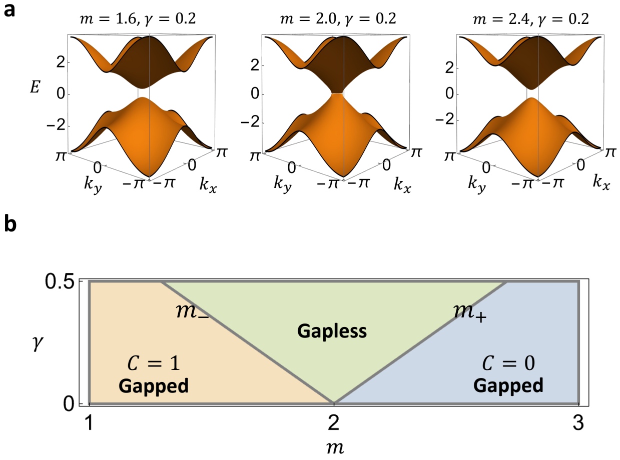

FIG. S11. Topological properties of 2D non-Hermitian Chern insulators. (a) The bandstructures of H of Eq. S36 with different parameters. Here, $v_x = v_y = t_x = t_y = 1$, $\gamma_z = 0$, $\gamma_x = \gamma_y = \gamma$. (b) The phase diagram of 2D non-Hermitian Chern insulators of Eq. S36. The green region denotes the gapless phase.

$m_\pm = t_x + t_y \pm \sqrt{\gamma_x^2 + \gamma_y^2}$, the system has a real line gap. While when $m \in [m_-, m_+]$, the system is in a gapless phase. Clearly, the Hermitian part of Eq. S36 is the Qi-Wu-Zhang model [22], meaning that the Hamiltonian of Eq. S36 has the same real line-gap topology as Qi-Wu-Zhang model. For simplicity, we set $v_x = v_y = t_x = t_y = 1$, $\gamma_z = 0$, $\gamma_x = \gamma_y = \gamma$. The real line-gap topology of Eq. S36 is described by the Chern number C [21]. The phase diagram of Eq. S36 is calculated and plotted in Fig. S11(b).

Here, we perform our unsupervised learning algorithm to detect the real line-gap topology in 2D non-Hermitian Chern insulator of Eq. S36. After randomly generating 500 Hamiltonian samples with $m \in [1, 3]$ and $\gamma \in [0, 0.5]$ (we fix $v_x = v_y = t_x = t_y = 1$, $\gamma_z = 0$ and $\gamma_x = \gamma_y = \gamma$), we exploit the similarity function to calculate the similarities between samples for the real line-gap topology and perform our algorithm to obtain the number of samples in different phases. The unsupervised learning results are shown in Fig.3(d) of the main text. By calculating the similarity between Hamiltonians with different m, γ and the samples in $\mathcal{G}$, we can obtain the topological phase diagram, which are consistent with the theoretical prediction in Fig. S11(b).

I. 2D non-Hermitian topological Möbius insulator

Here, we discuss the 2D non-Hermitian topological Möbius insulator, which has the Hamiltonian as

$$H = (t_1 + t_2\cos(k_y))\Gamma_1 + \left(t_2\sin(k_y) + i\frac{\gamma}{2}\right)\Gamma_2 + (\kappa + \kappa\cos(k_x))\Gamma_3 + \kappa\sin(k_x)\Gamma_4 \tag{S37}$$

where $\Gamma_1 = \sigma_z \otimes \sigma_x$, $\Gamma_2 = \sigma_z \otimes \sigma_y$, $\Gamma_3 = \sigma_x \otimes \sigma_0$ and $\Gamma_4 = \sigma_y \otimes \sigma_0$, $t_1, t_2, \kappa, \gamma \in \mathbb{R}$. The Hamiltonian possess: (1) Time-reversal symmetry: $H^*(k_x, k_y) = H(-k_x, -k_y)$; (2) Sublattice symmetry $\Gamma_5 = \sigma_z \otimes \sigma_z$: $\Gamma_5 H(k_x, k_y)\Gamma_5^{-1} = -H(k_x, k_y)$, $\Gamma_5^2 = 1$; (3) Projective translation symmetry $\mathcal{L}_x H(k_x, k_y)\mathcal{L}_x^{-1} = H(k_x, k_y)$, $\mathcal{L}_x^2 = -1$, where $\mathcal{L}_x = \mathcal{G}L_x$ is the translator L_x up to a gauge transformation $\mathcal{G} = \sigma_0 \otimes \sigma_z$, and $L_x = \begin{pmatrix} 0 & e^{-ik_x} \\ 1 & 0 \end{pmatrix} \otimes \sigma_0$. The non-Hermiticity $\gamma \neq 0$ introduces a gapless phase for certain parameters, as shown in Fig. S12(a). When the system of Eq. S37 has a real line gap, it can continuously deform to a Hermitian topological Möbius insulator [23–25]. In Fig. S12(a), we can see that the 2D non-Hermitian topological Möbius insulator possesses the nontrivial point-gap topology. We will discuss its open-boundary effect in the following sections. In the eigenspace of $\mathcal{L}_x$, the Hamiltonian H of Eq. S37 can be diagonalized into two blocks $H(\boldsymbol{k}) \to \mathrm{diag}[h_1^L(\boldsymbol{k}), h_2^L(\boldsymbol{k})]$. Because h_1^L and h_2^L are connected by the sublattice symmetry Γ_5, we can represent the topology based on h_1^L solely [23]. The real line-gap topological properties of 2D non-Hermitian topological Möbius insulator can be described by the following $\mathbb{Z}_2$ invariant

$$\nu = \frac{1}{2\pi}\int_{[0,2\pi)\times S^1} \mathcal{F} d^2k + \frac{1}{\pi}\theta(0) \bmod 2 \tag{S38}$$

where $\mathcal{F}$ is the Berry curvature, $\theta(0)$ is the Berry phase on the $k_x = 0$ path in the BZ of h_1^L. The phase diagram of 2D non-Hermitian topological Möbius insulator is calculated and plotted in Fig. S12(b).

Here, we perform our unsupervised learning algorithm to detect the real line-gap topology in 2D non-Hermitian topological Möbius insulator of Eq. S37. After randomly generating 500 Hamiltonian samples with $t_1 \in [0, 2]$ and $\gamma \in [0, 2]$ (we fix $t_2 = 1$ and $\kappa = 0.25$), we exploit the similarity function to calculate the similarities between samples for the real line-gap topology and perform our algorithm to obtain the number of samples in different phases. The unsupervised learning results are shown in Fig.3(e) of the main text. By calculating the similarity between Hamiltonians with different t_1, γ and the samples in $\mathcal{G}$, we can obtain the topological phase diagram, which are consistent with the theoretical prediction in Fig. S12(b).

J. 3D topological insulator phase induced by gain and loss

Here, we first introduce a lattice with the unit cell in Fig. S13(a), which is a gapless Hermitian system. The coupling strength is t_0, where the yellow and green denote positive and negative coupling, respectively. In Fig. S13(a), we then introduce on-site gain and loss, which preserve the three mirror-time-reversal symmetries (i.e., $\mathcal{M}_x\mathcal{T}$, $\mathcal{M}_y\mathcal{T}$ and $\mathcal{M}_z\mathcal{T}$) and $\mathcal{PT}$ symmetry. Two parameters g_1 and g_2 control the setting. Introducing symmetric on-site loss and gain (i.e., $g_1 \neq 0$, $g_2 \neq 0$) opens a real-line gap in the original lattice. Importantly, $g_1 g_2 > 0$ and $g_1 g_2 < 0$ correspond to topologically distinct phases, which can be described by the

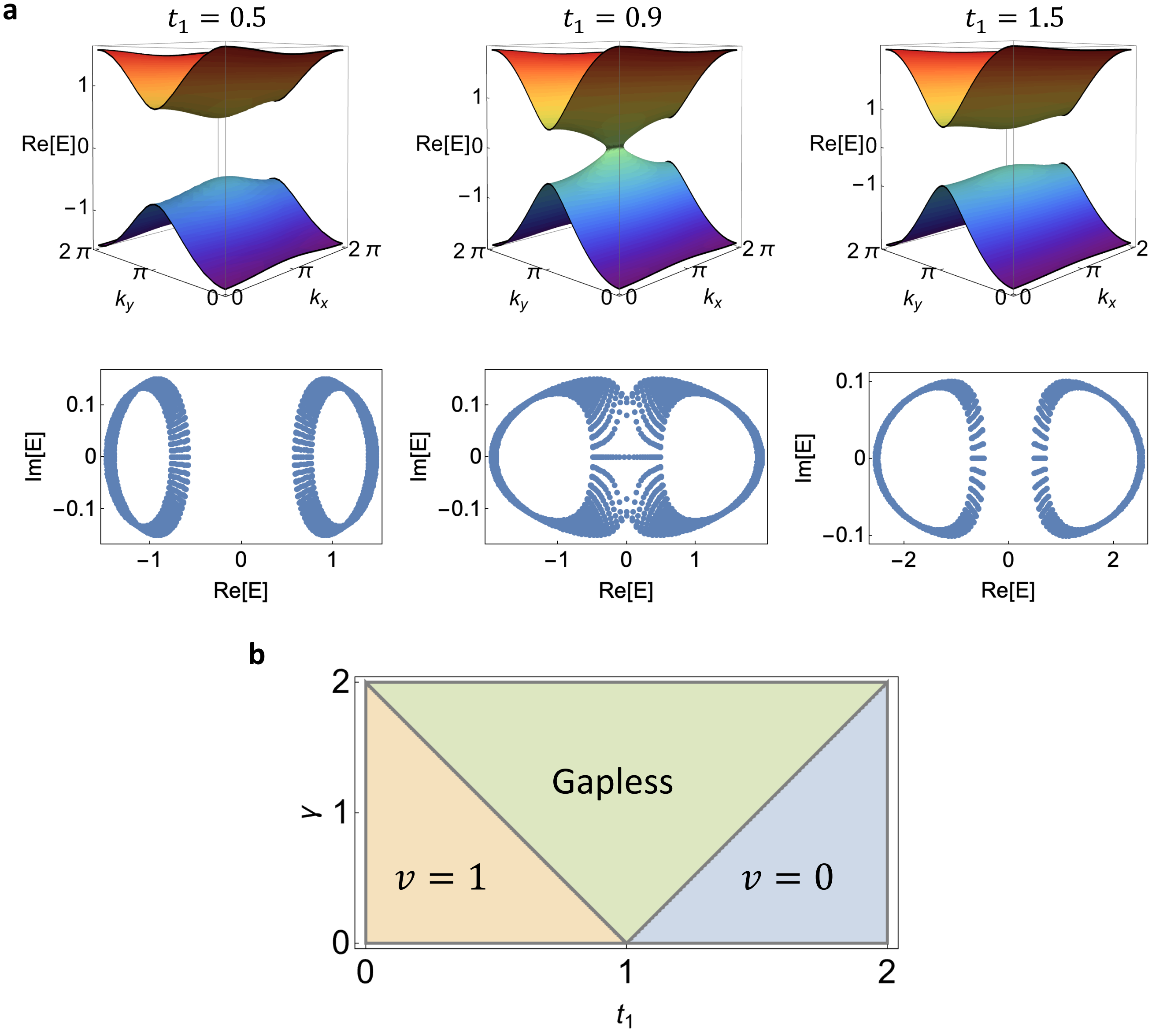

FIG. S12. Topological properties of 2D non-Hermitian topological Möbius insulators. (a) The bandstructures of H of Eq. S37 with different parameters. Here, $t_2 = 1$, $\gamma = 0.3$, $\kappa = 0.25$. (b) The phase diagram of the 2D non-Hermitian topological Möbius insulator of Eq. S37. The green region denotes the gapless phase. v denotes the $\mathbb{Z}_2$ topological invariant.

biorthogonal Wannier sector polarization p_α^v, where v refers to the Wannier sector and $\alpha = x, y, z$ [26, 27]. For calculating p_α^v, we take p_x^v as an example. We first calculate a biorthogonal Wilson loop along z direction, which has 2D Wannier bands. These 2D Wannier bands are gapped and split into two Wannier sectors. Based on one of the 2D Wannier sectors, we calculate a biorthogonal nested Wilson loop along y, which again has separated Wannier bands. One more biorthogonal nested Wilson loop gives the Wannier sector polarization along x as p_x^v [26, 28]. Similar procedures can be performed in other directions. When $g_1 g_2 > 0$, the system has zero p_α^v: $\{p_x^v, p_y^v, p_z^v\} = \{0, 0, 0\}$ corresponding to a trivial insulator, while when $g_1 g_2 < 0$, the system has non-zero p_α^v: $\{p_x^v, p_y^v, p_z^v\} = \{1/2, 1/2, 1/2\}$, corresponding to a topological insulator.

Here, we perform our unsupervised learning algorithm to detect the real line-gap topology in 3D non-Hermitian topological insulator in Fig. S13(a). After randomly generating 500 Hamiltonian samples with g_1,$g_2 \in [-3, 3]$ (we fix $t_0 = 1$), we exploit the similarity function of Eq.1 of the main text (or, Eq. S17) to calculate the similarities between samples for the real line-gap topology and perform our algorithm to obtain the number of samples in different phases. The unsupervised learning results are shown in Fig. S13(b), showing two distinct phases ($N_c = 2$) in the generated samples. By calculating the similarity between Hamiltonians with different g_1, g_2,

and the samples in $\mathcal{G}$, we can obtain the topological phase diagram, which are consistent with the theoretical prediction in Fig. S13(c).

We further discuss the topological phase of the above 3D non-Hermitian insulator and show how to deform the non-Hermitian topological system in Fig. S13(a) to a Hermitian topological system continuously without closing the real-line gap. For the non-Hermitian topological system induced by on-site loss and gain, we consider the Hamiltonian $H_1(H_2)$ for the topological (trivial) phase, respectively, as shown in Fig. S13(e). Building on the symmetries and topological invariants discussed above, we construct a supercell lattice from the topological octupole insulator [29]. This supercell, composed of $2 \times 2 \times 2$ original unit cells (totaling 64 sites), is governed by Hamiltonians $\widetilde{H}_1$ and $\widetilde{H}_2$ for the topological and trivial phases, respectively, as shown in Fig. S13(e). The couplings within the supercell are controlled by two parameters: γ for intra-cell couplings and λ for inter-cell couplings. We exploit the following linear interpolation as the continuous deformation $H_\alpha = (1-\alpha)H + \alpha\widetilde{H}$, where $\alpha \in [0, 1]$.

As shown in Fig. S13(f), the continuous deformation between H_1 and $\widetilde{H}_1$ doesn't close the real line gap, meaning that they are in the identical phase, while the continuous deformation between H_1 and $\widetilde{H}_2$ closes the real line gap (For simplicity, we don't show the robustness of the crossing point after introducing perturbations), meaning that they are in the distinct phases. Similarly, the continuous deformation between H_2 and $\widetilde{H}_2$ doesn't close the real line gap, meaning that they are in the identical phase, while the continuous deformation between H_2 and $\widetilde{H}_1$ closes the real line gap, meaning that they are in the distinct phases. Because $\widetilde{H}_1$ and $\widetilde{H}_2$ are topologically distinct, we can thus conclude that H_1 and H_2 are topologically distinct.

K. 3D topological insulator phase induced by non-reciprocity

Here, we also discuss the 3D topological phase solely induced by the non-reciprocal couplings. Like the previous case, we first introduce a lattice with the unit cell in Fig. S14(a), which is a gapless Hermitian system. The coupling strength is t_0 and the yellow/green color denotes the positive/negative coupling. In Fig. S14(a), we then introduce the non-reciprocal couplings, which preserve the three mirror-time-reversal symmetries (i.e., $\mathcal{M}_x\mathcal{T}$, $\mathcal{M}_y\mathcal{T}$ and $\mathcal{M}_z\mathcal{T}$) and $\mathcal{PT}$ symmetry. In Fig. S14(a), the direction and strength of the non-reciprocal coupling are controlled by the parameter ε, in a manner similar to that described in Sec. II G. Introducing these symmetric non-reciprocal couplings opens a gap in the original lattice's spectrum, as shown in Fig. S14(b). We adjust the coupling strength t_0 according to ε, for two reasons: (1) to maintain the overall energy range of the band structure, and (2) because $t_0 > 0$ does not affect the topological phase.

Here, we perform our unsupervised learning algorithm to detect the real line-gap topology in 3D non-Hermitian topological insulator in Fig. S14(a). After randomly generating 500 Hamiltonian samples with $\varepsilon \in [0, 10]$ (we fix $t_0 = 1$), we exploit the similarity function of Eq.1 of the main text (or, Eq. S17) to calculate the similarities between samples for the real line-gap topology and perform our algorithm to obtain the number of samples in different phases. The unsupervised learning results are shown in Fig. S14(c), showing two distinct phases ($N_c = 2$) in the generated samples. By calculating the similarity between Hamiltonians with different g_1, g_2, and the samples in $\mathcal{G}$, we can obtain the topological phase diagram. Note that while $\varepsilon \neq 1$ can gap out degenerate points in the band structure, inducing a topological phase transition, but a gap only occurs for values of ε that are sufficiently small or large (e.g., $\varepsilon < 0.4$ or $\varepsilon > 1.6$). As a result, (1) the number of gapped systems available for learning is less than 500, and (2) in the region around $\varepsilon = 1$, where no well-defined gap exists, the system is identified as $c = 0$ in the phase diagram.

We further discuss why our algorithm can identify 3D non-Hermitian topological phases. According to the phase diagram obtained by our algorithm, the Hamiltonian H_1 and H_2 in Fig. S14(b) can have distinct phases. Since the lattice preserves the same symmetry conditions as the previous cases, its topological phase can also be described by the biorthogonal Wannier sector polarization p_α^v, where v refers to the Wannier sector and $\alpha = x, y, z$ [26–28]. $\varepsilon < 1$ and $\varepsilon > 1$ correspond to topological distinct phases: $\{p_x^v, p_y^v, p_z^v\} = \{1/2, 1/2, 1/2\}$ for $\varepsilon < 1$, while $\{p_x^v, p_y^v, p_z^v\} = \{0, 0, 0\}$ for $\varepsilon > 1$. Besides the topological invariant, the topological difference of 3D non-Hermitian topological insulators can also be reflected by the absence of the continuous deformation. For the non-Hermitian topological system induced by non-reciprocity, the Hamiltonian H_1 (H_2) in Fig. S14(b) has the topological (trivial) phase, respectively. As shown in Fig. S14(d), the continuous deformation $H_\alpha = (1-\alpha)H_1 + \alpha H_2$, $\alpha \in [0, 1]$, closes the real line gap, meaning that they are in distinct phases. Our algorithm identifies 3D non-Hermitian topological phases by detecting the closure of the band gap during the continuous deformation.

Sec. III. OPEN-BOUNDARY EFFECT

In this section, we discuss the open-boundary effect on the non-Hermitian topological systems. As mentioned in the main text, the non-Hermitian system can break conventional bulk-boundary correspondence(BBC) in the Hermitian system when the non-Hermitian system has non-trivial point-gap topology. However, note that although the open-boundary effect can break BBC, it will not contribute new topological phases, namely there is no new topological phase induced by changing boundary conditions. One approach to describe the open-boundary effect is the usage of generalized Brillouin zone (GBZ), in which the Bloch coefficient e^{ik} is replaced by a complex number $\beta \in \mathbb{C}$, namely $e^{ik} \to \beta$ [15, 21]. In the following, we perform our unsupervised learning algorithm to some non-Hermitian topological systems based on GBZ.

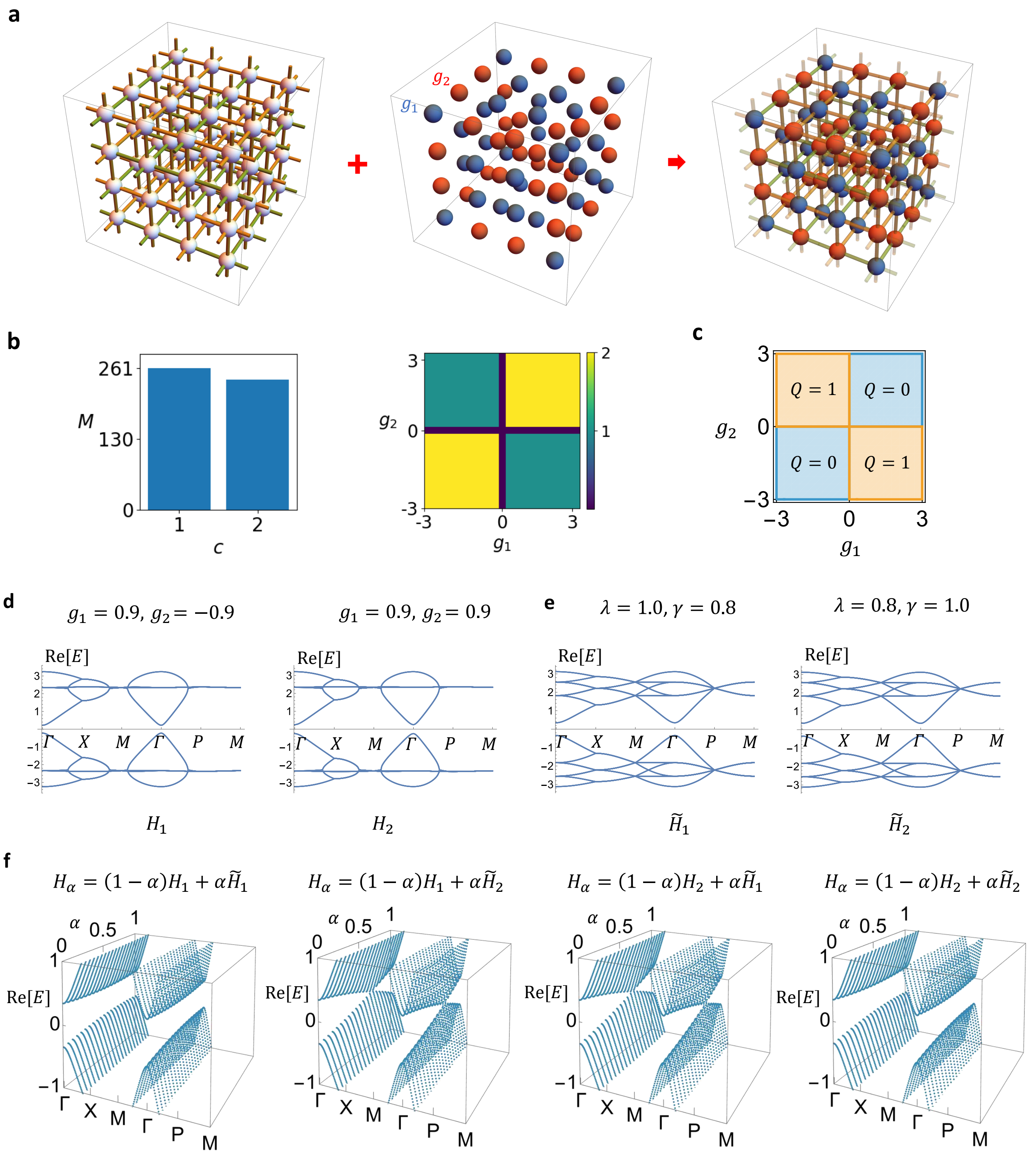

FIG. S13. 3D line-gap topological phases induced by on-site gain and loss and its unsupervised learning. (a) The formation of non-Hermitian lattice. A non-Hermitian lattice is formed by introducing onsite gain and loss to a Hermitian lattice with coupling strength t_0, where yellow and green denote positive and negative couplings, respectively. The on-site gain and loss are controlled by two parameters g_1 and g_2. (b) Unsupervised learning results and the phase diagram obtained by our algorithm. The Hamiltonian samples are generated by randomly varying $g_1, g_2 \in [-3, 3]$ and $t_0 = 1$. (c) The theoretical prediction of the topological phase diagram as a function of g_1 and g_2. Clearly, the phase diagram obtained by our algorithm is consistent with the theoretical prediction. (d) The bandstructures of the system in (a). (e) The bandstructures of 3D topological octupole insulators after taking a super unitcell. (f) The continuous deformationsbetween $H_{1,2}$ and $\widetilde{H}_{1,2}$.

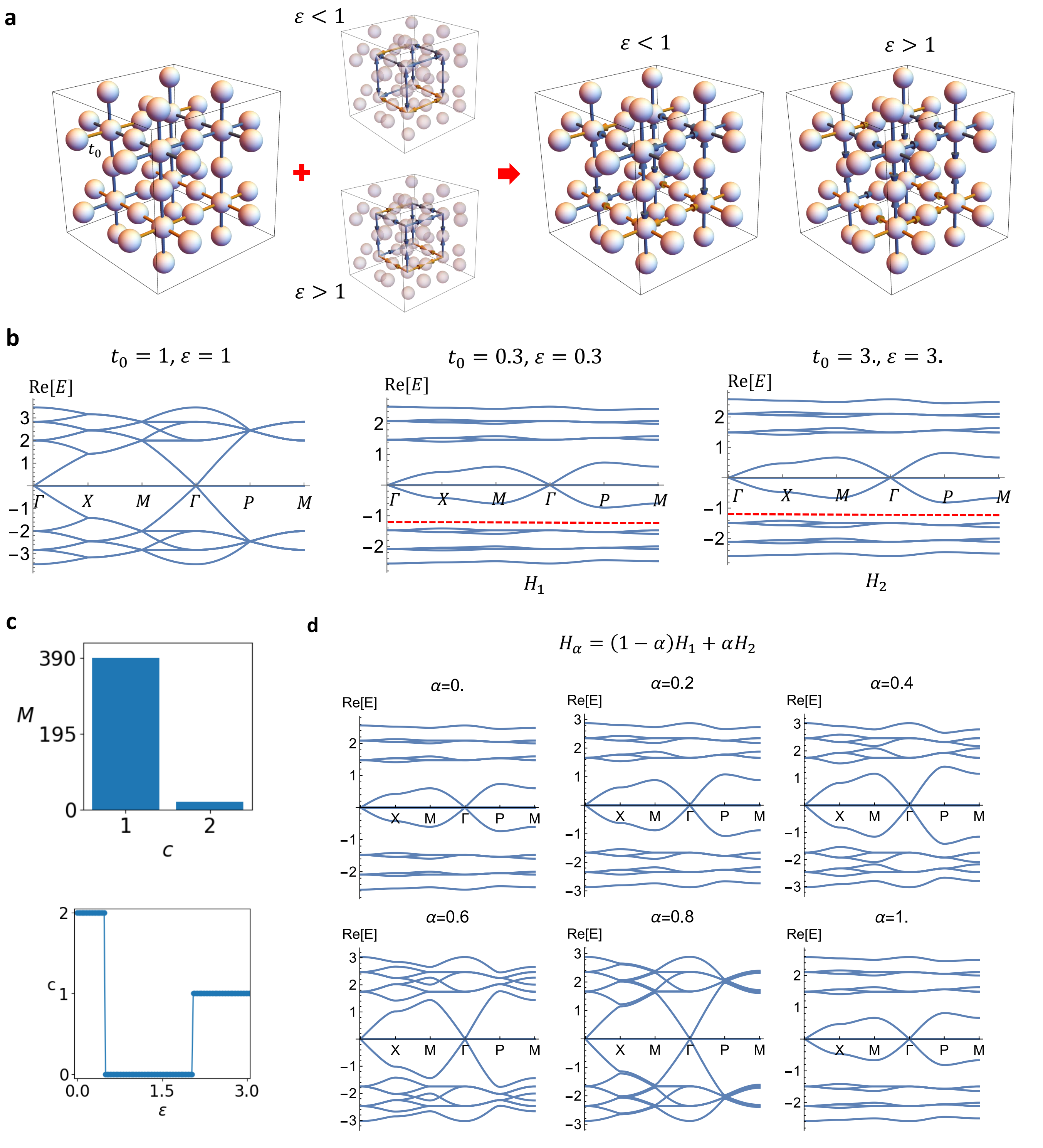

FIG. S14. 3D line-gap topological phases induced by non-reciprocity and its unsupervised learning. (a) The construction of the non-Hermitian lattice. We construct a non-Hermitian lattice by introducing nonreciprocal couplings to a Hermitian lattice with coupling strength t_0, where yellow and green denote positive and negative couplings, respectively. The nonreciprocal coupling is controlled by ε. (b) The degenerate points of the original Hermitian lattice can be gapped out by non-reciprocal coupling. The red dashed line denotes the Fermi level $E_f = -1.2$. (c) Unsupervised learning results and the phase diagram obtained by our algorithm. The Hamiltonian samples are generated by randomly varying $\varepsilon \in [0, 10]$ and $t_0 = 1$. (d) The continuous deformations between H_1 and H_2 in (b)

A. 1D non-Hermitian SSH system

As discussed in the previous sections, we show that the 1D non-Hermitian SSH system can have the non-trivial point-gap topology. To obtain the GBZ of 1D non-Hermitian SSH system, we firstly consider the finite system. For the real-space Hamiltonian $\mathcal{H}$ of 1D non-Hermitian SSH finite system based on Eq. S25, it has been shown that one can exploit the following similarity transformation to obtain an new Hamiltonian $\overline{\mathcal{H}}$ [15]

$$\overline{\mathcal{H}} = S^{-1}\mathcal{H}S \qquad \text{(S39)}$$

where S is a diagonal matrix whose diagonal elements are $\{1, r, r, r^2, r^2, ..., r^{L-1}, r^{L-1}, r^L\}$ [15, 16]. If we take $r = \sqrt{\frac{|t_1-\gamma/2|}{|t_1+\gamma/2|}}$, $\overline{\mathcal{H}}$ will become a standard SSH system [15]. In the momentum space, the standard SSH system reads

$$\overline{H} = (\bar{t}_1 + \bar{t}_2\cos(k))\sigma_x + \bar{t}_2\sin(k)\sigma_y \qquad \text{(S40)}$$

where

$$\bar{t}_1 = \sqrt{(t_1-\gamma/2)(t_1+\gamma/2)}, \quad \bar{t}_1 = t_2. \qquad \text{(S41)}$$

The transformation S implies that the GBZ phase factor β can be written as

$$\beta = e^{ik}\sqrt{\frac{|t_1-\gamma/2|}{|t_1+\gamma/2|}}. \qquad \text{(S42)}$$

From $\bar{t}_1$, we can see that γ can affect the topological phase transition point. However, it should be noted that some symmetry conditions can forbid the transformation [5] of Eq. S39. For example, if the system preserve parity($\mathcal{P}$) symmetry, the eigenstate satisfy $U_{\mathcal{P}}|\psi\rangle = \pm|\psi\rangle$, where $U_{\mathcal{P}}$ is the parity operator. However, for $|\psi'\rangle = S^{-1}|\psi\rangle$, $|\psi'\rangle$ does not preserve $\mathcal{P}$ due to the spatial decay nature of S, namely $U_{\mathcal{P}}|\psi'\rangle \neq \pm|\psi'\rangle$. According to the GBZ, the topological phase transition point after considering the open-boundary effect will be

$$t_1 = \pm\sqrt{t_2^2 + (\gamma/2)^2}. \qquad \text{(S43)}$$

The topological phase transition point for the 1D non-Hermitian SSH finite system is determined by $\overline{H}$ in GBZ [15], not the bulk Hamiltonian of Eq. S25 in the conventional BZ. The phase diagram after considering the open-boundary effect is calculated and shown in Fig. S15.

Here, we perform our algorithm to obtain the phase diagram after considering the open-boundary effect. To investigate the open-boundary effect, we exploit the GBZ to calculate the similarity function in Eq. S17, instead of the conventional BZ. This is realized by replacing e^{ik} with $\beta = re^{ik}$, where $r = \sqrt{\frac{|t_1-\gamma/2|}{|t_1+\gamma/2|}}$. After randomly generating 500 Hamiltonian samples with $t_1 \in [0, 3]$ and $\gamma \in [0, 3]$ (we fix $t_2 = 1$), we exploit the similarity function to calculate the similarities between samples for the real line-gap topology and perform our algorithm to obtain the number of samples in different phases.

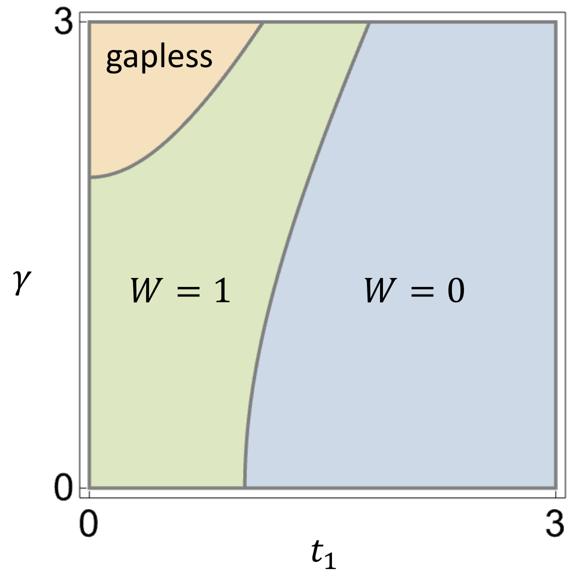

FIG. S15. The phase diagram of the 1D non-Hermitian SSH system under the open-boundary effect. W denotes the winding number of Eq. S26. Here, $t_2 = 1$.

The unsupervised learning results are shown in Fig.5(a) of the main text. By calculating the similarity between Hamiltonians with different t_1, γ and the samples in $\mathcal{S}$, we can obtain the topological phase diagram, which are consistent with the theoretical prediction in Fig. S15. Clearly, the number of gapped phases is still $N_c = 2$, namely that the open-boundary effect does not contribute more phases of the real line-gap topology.

B. 2D non-Hermitian Chern insulator

In the previous section, we discuss the unsupervised learning of 2D non-Hermitian Chern insulator under PBC. Due to the non-trivial point-gap topology, after changing the boundary conditions, the extended bulk modes will become localized modes [21]. When $\gamma_x \neq 0$, $\gamma_y \neq 0$ and $\gamma_z = 0$, the eigenstates of the real-space finite system of $\mathcal{H}$ of Eq. S36 become localized like $e^{(\gamma_x/v_x)x+(\gamma_y/v_y)y}$ [21], corresponding to the 2D NHSE. Similarly, we can exploit the GBZ to describe the open-boundary effect for the 2D non-Hermitian Chern insulator. The topological phase transition of 2D non-Hermitian Chern insulator can be predicted by the non-Bloch Chern number, i.e., Chern number defined based on GBZ [21]. The phase boundary in the phase diagram is described by [21] $m = t_x + t_y + \frac{t_x\gamma_x^2}{2v_x^2} + \frac{t_y\gamma_y^2}{2v_y^2}$. For $t_{x,y} = v_{x,y} = 1$, $\gamma_{x,y} = \gamma$, the phase boundary is

$$m = 2 + \gamma^2 \qquad \text{(S44)}$$

The phase diagram of 2D non-Hermitian Chern insulator with the open-boundary effect is calculated and plotted in Fig. S16.

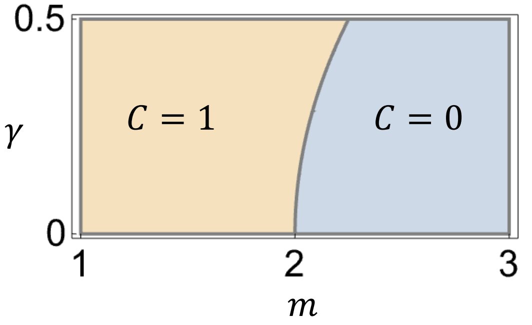

FIG. S16. The phase diagram of the 1D non-Hermitian SSH system under the open-boundary effect. C denotes the non-Bloch Chern number. Here, $t_{x,y} = v_{x,y} = 1, \gamma_{x,y} = \gamma$.

Here, we perform our algorithm to obtain the phase diagram after considering the open-boundary effect. To investigate the open-boundary effect, we exploit the GBZ to calculate the similarity function in Eq. S17, instead of the conventional BZ. This is realized by replacing e^{ik_x} and e^{ik_y} with $\beta_x = e^{i(k_x - \frac{\gamma_x}{v_x})}$ and $\beta_y = e^{i(k_y - \frac{\gamma_y}{v_y})}$ [21], respectively. After randomly generating 500 Hamiltonian samples with $m \in [0, 3]$ and $\gamma \in [0, 3]$ (we fix $t_{x,y} = v_{x,y} = 1$, $\gamma_{x,y} = \gamma$ and $\gamma_z = 0$), we exploit the similarity function to calculate the similarities between samples for the real line-gap topology and perform our algorithm to obtain the number of samples in different phases. The unsupervised learning results are shown in Fig.5(b) of the main text. By calculating the similarity between Hamiltonians with different m, γ and the samples in $\mathcal{G}$, we can obtain the topological phase diagram, which are consistent with the theoretical prediction in Fig. S16. Obviously, the number of gapped phases under OBC is still $N_c = 2$, identical to N_c under PBC.

C. 2D non-Hermitian topological Möbius insulator

Here, we discuss the open-boundary effect on 2D non-Hermitian topological Möbius insulator. In the topological Möbius insulator phase, the finite system of Eq. S37 has two edge bands forming a Möbius twist along x axis (preserves $\mathcal{L}_x$), as shown in Fig. S17(a). Because the 2D non-Hermitian topological Möbius insulator possesses the non-trivial point-gap topology, all the bulk modes are the skin modes. All the bulk modes become localized at one of edges, as shown in Fig. S17(b). The 2D non-Hermitian topological Möbius insulator has the similar GBZ as the 1D non-Hermitian SSH system, which reads

$$\beta_x = e^{ik_x}, \quad \beta_y = e^{ik_y}\sqrt{\frac{|t_1 - \gamma/2|}{|t_1 + \gamma/2|}} \tag{S45}$$

The phase diagram of 2D non-Hermitian topological Möbius insulator is calculated and plotted in Fig. S12(b).

Here, we perform our algorithm to obtain the phase diagram after considering the open-boundary effect. To investigate the open-boundary effect, we exploit the GBZ to calculate the similarity function in Eq. S17, instead of the conventional BZ. This is realized by replacing e^{ik_y} and $\beta_y = e^{ik_y}\sqrt{\frac{|t_1-\gamma/2|}{|t_1+\gamma/2|}}$. After randomly generating 500 Hamiltonian samples with $t_1 \in [0, 3]$ and $\gamma \in [0, 3]$ (we fix $t_2 = 1$ and $\kappa = 0.2$), we exploit the similarity function to calculate the similarities between samples for the real line-gap topology and perform our algorithm to obtain the number of samples in different phases. The unsupervised learning results are shown in Fig.5(c) of the main text. By calculating the similarity between Hamiltonians with different t_1, γ and the samples in $\mathcal{G}$, we can obtain the topological phase diagram, which are consistent with the theoretical prediction in Fig. S17(c). Obviously, the number of gapped phases under OBC is still $N_c = 2$, identical to N_c under PBC.

Sec. IV. GENERATION OF SYMMETRY-PRESERVING HAMILTONIAN SAMPLES VIA RANDOM MATRIX TECHNOLOGY

In this section, we show the details about the construction of non-Hermitian Hamiltonian samples under symmetries based on random matrices. The dataset is a collection of Hamiltonians, i.e., $\{H_m\}$, where the subscript m denotes the m-th sample. Each Hamiltonian sample represents a non-Hermitian system. Here, we focus on the three well-known symmetry conditions: time-reversal symmetry ($\mathcal{T}_\pm$), particle-hole symmetry ($\mathcal{C}_\pm$), and chiral symmetry (Γ), and two additional symmetry conditions: sublattice symmetry ($\mathcal{S}$) and pseudo-Hermiticity symmetry (η).

A. Symmetry conditions for non-Hermitian Hamiltonian

Because of $H^T \neq H^*$ for non-Hermitian Hamiltonian, symmetry conditions become more complicated than Hermitian cases. Some symmetry conditions in Hermitian systems will become two distinct types due to symmetry amplification for non-Hermitian systems [4], for example, the time-reversal symmetry and particle-hole symmetry can have two different forms $\mathcal{T}_\pm$ and $\mathcal{C}_\pm$, respectively. Here, we give a brief review of symmetry conditions in non-Hermitian systems.

Firstly, the non-Hermitian symmetry classes include traditional Altland-Zirnbauer (AZ) classes for Hermitian system [6]. The symmetry conditions in AZ classes are described as

$$\begin{aligned} U_{\mathcal{T},+}H^*(\boldsymbol{k})U_{\mathcal{T},+}^{-1} &= H(-\boldsymbol{k}), \quad U_{\mathcal{T},+}U_{\mathcal{T},+}^* = \pm 1 \\ U_{\mathcal{C},+}H^T(\boldsymbol{k})U_{\mathcal{C},+}^{-1} &= -H(-\boldsymbol{k}), \quad U_{\mathcal{C},+}U_{\mathcal{C},+}^* = \pm 1 \\ U_\Gamma H^\dagger(\boldsymbol{k})U_\Gamma^{-1} &= -H(\boldsymbol{k}), \quad U_\Gamma^2 = 1 \end{aligned} \tag{S46}$$

where $U_{\mathcal{T},+}$ and $U_{\mathcal{C},+}$ are the $\mathcal{T}_+$ and $\mathcal{C}_+$ symmetry operators, U_Γ is the chiral symmetry operator. Because of non-Hermitian nature $H^T \neq H^*$, the above symmetry conditions can be extended to a conjugate form AZ† classes [4], which are de-

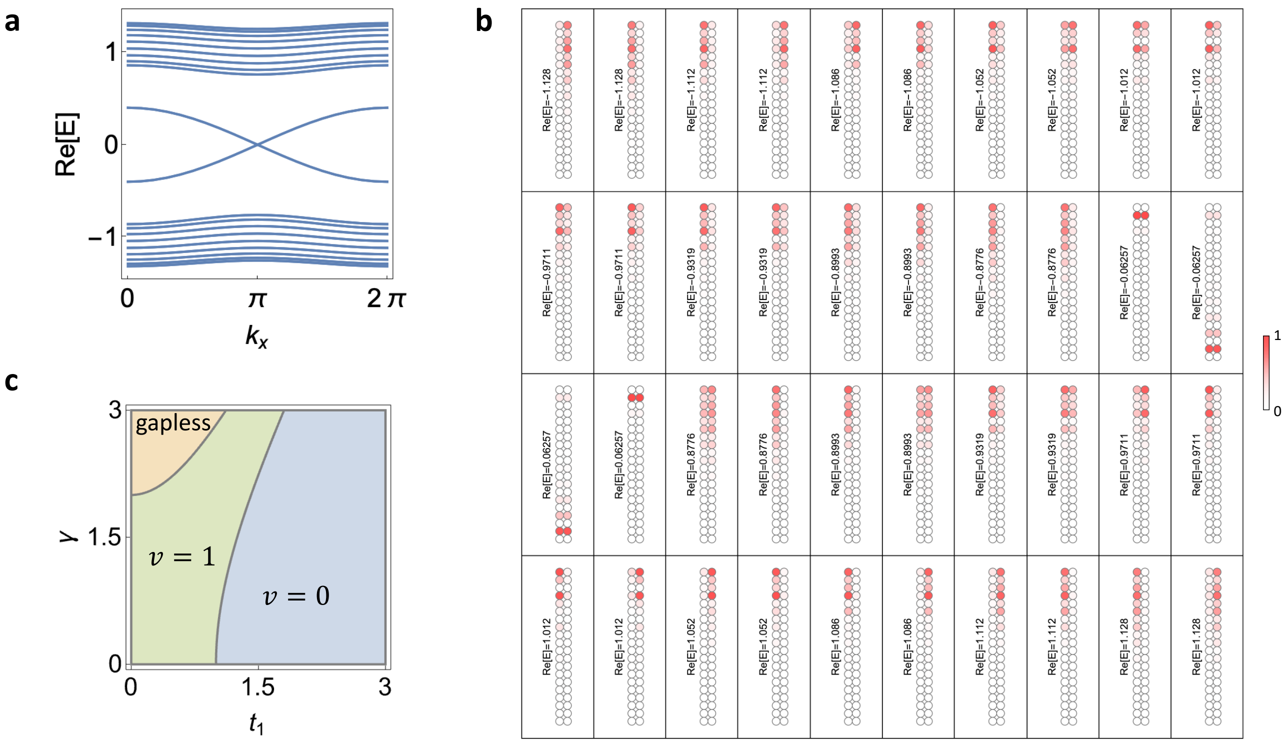

FIG. S17. The topological properties of the 2D non-Hermitian topological Möbius insulator under the open-boundary effect. (a) The energy spectrum of the edge bands. (b) The eigenstates of edge bands when $k_x = 1.1\pi$. (c) The phase diagram. ν denotes the $\mathbb{Z}_2$ topological invariant of Eq. S38. Here, $t_2 = 1$, $\kappa = 0.2$.

scribed by the following symmetry conditions

$$\begin{aligned} U_{\mathcal{T},-}H^T(\boldsymbol{k})U_{\mathcal{T},-}^{-1} &= H(-\boldsymbol{k}), & U_{\mathcal{T},-}U_{\mathcal{T},-}^{*} &= \pm 1 \\ U_{C,-}H^{*}(\boldsymbol{k})U_{C,-}^{-1} &= -H(-\boldsymbol{k}), & U_{C,-}U_{C,-}^{*} &= \pm 1 \end{aligned} \tag{S47}$$

where $U_{\mathcal{T},-}$ and $U_{C,-}$ are the $\mathcal{T}_-$ and C_- symmetry operators. Specially, AZ† classes have the same definition of chiral symmetry Γ as AZ classes, namely $\Gamma = \mathcal{T}C$. The symmetry conditions for AZ and AZ† classes for non-Hermitian systems are listed in Table. S1.

Besides $\mathcal{T}_\pm$, $C_\pm$ and Γ symmetry, the non-Hermitian system can have sublattice symmetry $\mathcal{S}$ and pseudo-Hermiticity symmetry. The $\mathcal{S}$ symmetry is defined by

$$U_{\mathcal{S}}H(\boldsymbol{k})U_{\mathcal{S}}^{-1} = -H(\boldsymbol{k}), \quad U_{\mathcal{S}}^2 = 1. \tag{S48}$$

where $U_{\mathcal{S}}$ is the $\mathcal{S}$ symmetry operator. In Hermitian systems, the $\mathcal{S}$ symmetry is identical to Γ symmetry. The $\mathcal{S}$ symmetry can have the following commutation/anticommutation relations with time-reversal or particle-hole symmetries:

$$\begin{aligned} U_{\mathcal{T},\pm}U_{\mathcal{S}}^{*} &= \epsilon_{\mathcal{T}}U_{\mathcal{S}}U_{\mathcal{T},\pm} \\ U_{C,\pm}U_{\mathcal{S}}^{*} &= \epsilon_{C}U_{\mathcal{S}}U_{C,\pm} \end{aligned} \tag{S49}$$

where $\epsilon_{\mathcal{T}}, \epsilon_C \in \{+,-\}$. When the system has $\mathcal{T}_\pm$ and $C_\pm$ symmetries, we denote the $\mathcal{S}$ symmetry with the subscripts $\mathcal{S}_{\epsilon_{\mathcal{T}}\epsilon_C}$. If there is only one symmetry, the subscript will only have one sign.

Pseudo-Hermiticity symmetry (η symmetry) serves as an key internal symmetry, which is defined by

$$U_\eta H^\dagger(\boldsymbol{k})U_\eta^{-1} = H(\boldsymbol{k}), \quad U_\eta^2 = 1 \tag{S50}$$

where U_η is the η symmetry operator. Similarly, the η symmetry can have the following commutation/anticommutation relations with time-reversal or particle-hole symmetries:

$$\begin{aligned} U_{\mathcal{T},\pm}U_{\eta}^{*} &= \mu_{\mathcal{T}}U_{\eta}U_{\mathcal{T},\pm} \\ U_{C,\pm}U_{\eta}^{*} &= \mu_{C}U_{\eta}U_{C,\pm} \end{aligned} \tag{S51}$$

where $\mu_{\mathcal{T}}, \mu_C \in \{+,-\}$. When the system has $\mathcal{T}_\pm$ and $C_\pm$ symmetries, we denote the η symmetry with the subscripts $\eta_{\mu_{\mathcal{T}}\mu_C}$. If there is only one symmetry, the subscript will only have one sign.

B. Symmetry unification

Here, we discuss the symmetry unification of antiunitary symmetry operators for non-Hermitian Hamiltonians and symmetry equivalence after introducing additional symmetries like $\mathcal{S}$ and η symmetries. Symmetry unification means that some different symmetry classes can be equivalent due to the non-Hermiticity. The main reason is that the non-Hermitian Hamiltonian can have degree of freedom to continuously deform energy spectra in complex energy plane. One

TABLE S1. AZ and AZ† symmetry classes for non-Hermitian Hamiltonians. The sign ±1 corresponds to $U_{\mathcal{T},\pm}U^*_{\mathcal{T},\pm} = \pm1$ or $U_{C,\pm}U^*_{C,\pm} = \pm1$. The value 1 and 0 mean $U^2_\Gamma = 1$ and no corresponding symmetry, respectively.

Symmetry class	$\mathcal{T}_+$	C_+	$\mathcal{T}_-$	C_-	Γ
A	0	0	0	0	0
AIII	0	0	0	0	1
AI	+1	0	0	0	0
BDI	+1	+1	0	0	1
D	0	+1	0	0	0
DIII	-1	+1	0	0	1
AII	-1	0	0	0	0
CII	-1	-1	0	0	1
C	0	-1	0	0	0
CI	+1	-1	0	0	1
AI†	0	0	+1	0	0
BDI†	0	0	+1	+1	1
D†	0	0	0	+1	0
DIII†	0	0	-1	+1	1
AII†	0	0	-1	0	0
CII†	0	0	-1	-1	1
C†	0	0	0	-1	0
CI†	0	0	+1	-1	1

main case is the symmetry unification between antiunitary operators of $\mathcal{T}$ and C-symmetries for non-Hermitian Hamiltonians [30]. Besides, some symmetry classes with additional symmetry are equivalent to the other remaining classes. As a consequence, each AZ symmetry class with $\mathcal{S}$ symmetry is equivalent to an AZ† symmetry class with $\mathcal{S}$ symmetry or am AZ symmetry class with η symmetry [4]. In the following, we will take some examples to show symmetry unification.

class D† and class AI According to the Table. S1, the symmetry condition for a Hamiltonian H_1 in class AI is

$$U_{\mathcal{T},+}H_1^*(\boldsymbol{k})U^{-1}_{\mathcal{T},+} = H_1(-\boldsymbol{k}), \quad U_{\mathcal{T},+}U^*_{\mathcal{T},+} = 1 \tag{S52}$$

The symmetry condition for a Hamiltonian H_2 in class D† is

$$U_{C,-}H_2^*(\boldsymbol{k})U^{-1}_{C,-} = -H_2(-\boldsymbol{k}), \quad U_{C,-}U^*_{C,-} = 1 \tag{S53}$$

Due to the allowance of complex energy spectrum, we can represent the C_- symmetry condition into the following form

$$U_{C,-}(iH_2(\boldsymbol{k}))^*U^{-1}_{C,-} = iH_2(-\boldsymbol{k}), \quad U_{C,-}U^*_{C,-} = 1 \tag{S54}$$

Clearly, iH_2 has an emergent "time-reversal" symmetry with a operator as $U_{C,-}$. When H_1 has $\mathcal{T}_+$ symmetry, there will be a non-Hermitian Hamiltonian $H_2 = -iH_1$ having C_- symmetry. Namely, there is one-to-one mapping between H_1 and H_2. Therefore, the topology of H_2 in class D† can be captured by H_1 in class AI, meaning that they share the identical topological classification. After exchanging the topological classifications of the real/imaginary line-gap topology for class AI, we can obtain the topological classification for class D†. Note that, for Hermitian Hamiltonian, it is not allowed to construct such a Hermitian Hamiltonian iH_2, because if H_2 is Hermitian, iH_2 will be non-Hermitian.

class C† and class AII According to the Table. S1, the symmetry condition for a Hamiltonian H_1 in class AII is

$$U_{\mathcal{T},+}H_1^*(\boldsymbol{k})U^{-1}_{\mathcal{T},+} = H_1(-\boldsymbol{k}), \quad U_{\mathcal{T},+}U^*_{\mathcal{T},+} = -1 \tag{S55}$$

The symmetry condition for a Hamiltonian H_2 in class C† is

$$U_{C,-}H_2^*(\boldsymbol{k})U^{-1}_{C,-} = -H_2(-\boldsymbol{k}), \quad U_{C,-}U^*_{C,-} = -1 \tag{S56}$$

We can represent the above C_- symmetry condition into the following form

$$U_{C,-}(iH_2(\boldsymbol{k}))^*U^{-1}_{C,-} = iH_2(-\boldsymbol{k}), \quad U_{C,-}U^*_{C,-} = -1 \tag{S57}$$

Similar to the previous case, there is one-to-one mapping from H_1 to H_2. Therefore, the topology of H_2 in class C† can be captured by H_1 in class AII, meaning that they share the identical topological classification.

Class DIII with $\mathcal{S}_{-+}$ and class BDI with $\mathcal{S}_{-+}$ According to the Table. S1, a Hamiltonian H_1 in class DIII with $\mathcal{S}_{-+}$ has the following symmetry conditions

$$\begin{aligned} U_{\mathcal{T},+}H^*(\boldsymbol{k})U^{-1}_{\mathcal{T},+} &= H(-\boldsymbol{k}), \quad U_{\mathcal{T},+}U^*_{\mathcal{T},+} = -1 \\ U_{C,+}H^T(\boldsymbol{k})U^{-1}_{C,+} &= -H(-\boldsymbol{k}), \quad U_{C,+}U^*_{C,+} = 1 \\ U_{\mathcal{S}}H(\boldsymbol{k})U^{-1}_{\mathcal{S}} &= -H(\boldsymbol{k}), \quad U^2_{\mathcal{S}} = 1 \\ U_{\mathcal{T},+}U^*_{\mathcal{S}} &= -U_{\mathcal{S}}U_{\mathcal{T},+}, \quad U_{C,+}U^*_{\mathcal{S}} = U_{\mathcal{S}}U_{C,+} \end{aligned} \tag{S58}$$

We can define the following operator of a new "time-reversal" symmetry:

$$U_{\mathcal{T}',+} = U_{\mathcal{S}}U_{\mathcal{T},+} \tag{S59}$$

The symmetry conditions of Eq. S58 are represented into the following equivalent forms:

$$\begin{aligned} U_{\mathcal{T}',+}(iH(\boldsymbol{k}))^*U^{-1}_{\mathcal{T}',+} &= iH(-\boldsymbol{k}), \quad U_{\mathcal{T}',+}U^*_{\mathcal{T}',+} = 1 \\ U_{C,+}(iH(\boldsymbol{k}))^TU^{-1}_{C,+} &= -iH(-\boldsymbol{k}), \quad U_{C,+}U^*_{C,+} = 1 \\ U_{\mathcal{S}}(iH(\boldsymbol{k}))U^{-1}_{\mathcal{S}} &= -iH(\boldsymbol{k}), \quad U^2_{\mathcal{S}} = 1 \\ U_{\mathcal{T}',+}U^*_{\mathcal{S}} &= -U_{\mathcal{S}}U_{\mathcal{T}',+}, \quad U_{C',+}U^*_{\mathcal{S}} = U_{\mathcal{S}}U_{C',+} \end{aligned} \tag{S60}$$

Clearly, through the transformation of Eq. S59, the symmetry conditions of Eq. S58 for H in class DIII with $\mathcal{S}_{-+}$ can map to symmetry conditions of Eq. S60 for iH in class BDI with $\mathcal{S}_{-+}$. Namely, class DIII with $\mathcal{S}_{-+}$ and class BDI with $\mathcal{S}_{-+}$ can have the identical topological classification.

Class AI† with $\mathcal{S}_+$ and class D with $\mathcal{S}_+$ According to the Table. S1, the symmetry conditions for a Hamiltonian H in class AI† with S_+ are

$$\begin{aligned} U_{\mathcal{T},-}H^T(\boldsymbol{k})U^{-1}_{\mathcal{T},-} &= H(-\boldsymbol{k}), \quad U_{\mathcal{T},-}U^*_{\mathcal{T},-} = 1 \\ U_{\mathcal{S}}H(\boldsymbol{k})U^{-1}_{\mathcal{S}} &= -H(\boldsymbol{k}), \quad U^2_{\mathcal{S}} = 1 \\ U_{\mathcal{T},-}U^*_{\mathcal{S}} &= U_{\mathcal{S}}U_{\mathcal{T},-} \end{aligned} \tag{S61}$$

We can define the following operator for a new "particle-hole" symmetry: $U_{C',+} = U_{\mathcal{S}}U_{\mathcal{T},-}$. The symmetry conditions can be represented into the following new forms:

$$\begin{aligned} U_{C',+}H^T(\boldsymbol{k})U^{-1}_{C',+} &= -H(-\boldsymbol{k}), \quad U_{C,+}U^*_{C',+} = 1 \\ U_{C',+}U^*_{\mathcal{S}} &= U_{\mathcal{S}}U_{C',+} \end{aligned} \tag{S62}$$

Similarly, through the transformation of the operator, the symmetry conditions for H in class AI† with $\mathcal{S}_+$ map to symmetry conditions for H in class D with $\mathcal{S}_+$. Namely, class AI† with $\mathcal{S}_+$ and class D with $\mathcal{S}_+$ can have the identical topological classification.

Class AI with η_+ and class BDI† According to the Table. S1, the symmetry conditions of class AI with η_+ are

$$\begin{aligned} U_{\mathcal{T},+}H^*(\boldsymbol{k})U_{\mathcal{T},+}^{-1} &= H(-\boldsymbol{k}), \quad U_{\mathcal{T},+}U_{\mathcal{T},+}^* = 1 \\ U_\eta H^\dagger(\boldsymbol{k})U_\eta^{-1} &= H(\boldsymbol{k}), \quad U_\eta^2 = 1 \\ U_{\mathcal{T},+}U_\eta^* &= U_\eta U_{\mathcal{T},+} \end{aligned} \tag{S63}$$

We can define the following operator for a new "particle-hole" symmetry: $U_{C',-} = U_\eta U_{\mathcal{T},+}$. The symmetry conditions can be represented into the following new forms:

$$\begin{aligned} U_{C',-}(iH(\boldsymbol{k})^T)U_{C',-}^{-1} &= iH(-\boldsymbol{k}), \quad U_{C',-}U_{C',-}^* = 1 \\ U_{\mathcal{T},+}(iH(\boldsymbol{k}))^*U_{\mathcal{T},+}^{-1} &= -iH(-\boldsymbol{k}), \quad U_{\mathcal{T},+}U_{\mathcal{T},+}^* = 1 \end{aligned} \tag{S64}$$

Similarly, through the transformation of the operator, the symmetry conditions for H in class AI with η_+ map to the symmetry conditions for iH in class BDI†. Namely, class AI with η_+ and class BDI† can have the identical topological classification.

Class BDI with η_{+-} and class BDI with $\mathcal{S}_{-+}$ According to the Table. S1, the symmetry conditions for H in class BDI with $\mathcal{S}_{-+}$ are

$$\begin{aligned} U_{\mathcal{T},+}H^*(\boldsymbol{k})U_{\mathcal{T},+}^{-1} &= H(-\boldsymbol{k}), \quad U_{\mathcal{T},+}U_{\mathcal{T},+}^* = 1 \\ U_{C,+}H^T(\boldsymbol{k})U_{C,+}^{-1} &= -H(-\boldsymbol{k}), \quad U_{C,+}U_{C,+}^* = 1 \\ U_{\mathcal{S}}H(\boldsymbol{k})U_{\mathcal{S}}^{-1} &= -H(\boldsymbol{k}), \quad U_{\mathcal{S}}^2 = 1 \\ U_{\mathcal{T},+}U_{\mathcal{S}}^* &= -U_{\mathcal{S}}U_{\mathcal{T},+}, \quad U_{C,+}U_{\mathcal{S}}^* = U_{\mathcal{S}}U_{C,+} \end{aligned} \tag{S65}$$

The additional symmetry $\mathcal{S}_{-+}$ can induce an emergent pseudo-Hermiticity symmetry η. We can have a new operator for pseudo-Hermiticity symmetry: $U_\eta = U_{\mathcal{S}}U_{\mathcal{T},+}U_{C,+}$ if $[\mathcal{T}_+, C_+] = 0$, or $U_\eta = U_{\mathcal{S}}U_{C,+}U_{\mathcal{T},+}$ if $\{\mathcal{T}_+, C_+\} = 0$. The pseudo-Hermiticity symmetry can be represented into the following form:

$$\begin{aligned} U_\eta H^*(\boldsymbol{k})U_\eta^{-1} &= H(\boldsymbol{k}), \quad U_\eta^2 = 1 \\ U_{\mathcal{T},+}\eta^* &= U_\eta U_{\mathcal{T},+}, \quad U_{C,+}\eta^* = -U_\eta U_{C,+} \end{aligned} \tag{S66}$$

Similarly, the Hamiltonian H in class BDI with η_{+-} can also obtain an emergent $\mathcal{S}$ symmetry. The above relations reflect that H in class BDI with $\mathcal{S}_{-+}$ is also in class BDI with η_{+-}. Namely, class BDI with η_{+-} and class BDI with $\mathcal{S}_{-+}$ can have the identical topological classification.

C. 38 symmetry classes

After removing redundant symmetry classes due to symmetry unification, there are totally 38 symmetry classes for non-Hermitian Hamiltonians [4, 31]. There are 10 AZ classes + 6 AZ† classes + 22 AZ classes with $\mathcal{S}$ symmetry. The equivalent symmetry classes in AZ classes, AZ† classes and AZ classes with $\mathcal{S}$ symmetry are summarized in Table. S2.

TABLE S2. Equivalent symmetry classes for non-Hermitian Hamiltonians.

Symmetry class	Equivalent symmetry class
D†	AI
C†	AII
DIII + $\mathcal{S}_{-+}$	BDI + $\mathcal{S}_{-+}$
AII + $\mathcal{S}_-$	AI + $\mathcal{S}_-$
CI + $\mathcal{S}_{-+}$	CII + $\mathcal{S}_{-+}$
BDI + $\mathcal{S}_{--}$	DIII + $\mathcal{S}_{--}$
CII + $\mathcal{S}_{--}$	CI + $\mathcal{S}_{--}$

It was thought to have 42 symmetry classes for non-Hermitian matrices, which is known as Bernard-LeClair classes [32]. The Bernard-LeClair classes describes non-Hermitian random matrices according to the following symmetries:

$$\begin{aligned} \mathfrak{C}H^T\mathfrak{C}^{-1} &= \pm H, \quad \mathfrak{C}^T\mathfrak{C}^{-1} = \pm 1 \\ \mathfrak{P}H\mathfrak{P}^{-1} &= -H, \quad \mathfrak{P}^2 = 1 \\ \mathfrak{Q}H^\dagger\mathfrak{Q}^{-1} &= H, \quad \mathfrak{Q}^\dagger\mathfrak{Q}^{-1} = 1 \\ \mathfrak{K}H^*\mathfrak{K}^{-1} &= H, \quad \mathfrak{K}\mathfrak{K}^* = \pm 1 \end{aligned} \tag{S67}$$

where $\mathfrak{C}, \mathfrak{P}, \mathfrak{Q}, \mathfrak{K}$ are unitary operators. 38 non-Hermitian symmetry classes can have the corresponding counterparts in Bernard-LeClair classes, e.g., K-symmetry in Bernard-LeClair classes can be represented by $\mathcal{T}_+$ and C_-. However, Bernard-LeClair classes overlook and overcount 5 symmetry classes, because they distinguished the pseudo-Hermiticity with positivity from the pseudo-Hermicitiy without positivity [4]. The pseudo-Hermiticity symmetry with positivity is equivalent to Hermiticity condition, corresponding to the trivial pseudo-Hermiticity, namely $H^\dagger = H$ ($U_\eta = 1$).

D. Random generation of non-Hermitian Hamiltonian

Any non-Hermitian Hamiltonian H can be decomposed into two Hermitian parts:

$$H(\boldsymbol{k}) = h_1(\boldsymbol{k}) + ih_2(\boldsymbol{k}) \tag{S68}$$

where

$$h_1 = \frac{1}{2}(H + H^\dagger), \quad h_2 = \frac{1}{2i}(H - H^\dagger). \tag{S69}$$

Clearly, the h_1 and h_2 are two Hermitian Hamiltonians, namely, $h_1^\dagger = h_1$ and $h_2^\dagger = h_2$. Instead of generating a random non-Hermitian Hamiltonian directly, we generate two random Hermitian Hamiltonian h_1 and h_2 according to the symmetry conditions for H. However, the symmetry conditions of h_1 can be different from the symmetry conditions of h_2. The corresponding symmetry conditions for h_1 and h_2 are listed in Table. S3. The symmetry classes for h_1 and h_2 are listed in Table. S4 for AZ and AZ† classes. Combining the sublattice symmetry $\mathcal{S}$, the symmetry classes for h_1 and h_2 are listed in

Table. S5. The generation scheme for h_1 and h_2 in different Hermitian symmetry classes can be found in Ref. [1].

Non-Hermitian symmetry classes allow the existence of the Hamiltonian subspaces. If an unitary operator U can commute with Hamiltonian H, namely,

$$[H, U] = 0, \quad UU^\dagger = U^\dagger U = 1. \tag{S70}$$

We can block diagonalize H based on the eigenvectors of U. Thus, we can construct the Hamiltonian H in a block-diagonal form, i.e., $H = \text{diag}[B_1, B_2]$, and introduce the remaining symmetry constraints to these two independent blocks $\{B_1, B_2\}$, respectively. For example, if a non-Hermitian Hamiltonian has pseudo-Hermiticity symmetry, $U_\eta H^\dagger U_\eta^{-1} = H$, its Hermitian part h_1 will have $U_\eta h_1^\dagger U_\eta^{-1} = h_1$. Because $h_1^\dagger = h_1$, $[h_1, U_\eta] = 0$ means that h_1 can be in a block diagonalized form. If a non-Hermitian Hamiltonian has chiral antisymmetry, $U_\Gamma H^\dagger U_\Gamma^{-1} = H$, its Hermitian part h_1 will also have $U_\Gamma h_1 U_\Gamma^{-1} = h_1$ (i.e., $[h_1, \Gamma] = 0$). h_1 can be in a block diagonalized form, $h_1 = b_1 \oplus b_2$, where b_1 and b_2 are two different and independent Hermitian Hamiltonians.

1. *Complexification*

Some symmetry classes with additional symmetries can change from real AZ classes to complex AZ classes [33]. One known example is the quantum spin Hall insulator preserving the z component of spin. When the quantum spin Hall insulator only has spinful time-reversal symmetry (class AII), it has $\mathbb{Z}_2$ topological insulator phase. However, with keeping the system invariant under the twofold spin rotation along the z direction, the topological properties will be characterized by $\mathbb{Z}$ topological phase, and the corresponding topological number becomes the spin Chern number. For a non-Hermitian Hamiltonian, the symmetry class for its Hermitian parts h_1 and h_2 can change dramatically after combining them with additional symmetry conditions. Here, we take class DIII as an example. The class DIII has $\mathcal{T}_+$ symmetry ($U_{\mathcal{T},+}U_{\mathcal{T},+}^* = -1$) and C_- symmetry ($U_{C,-}U_{C,-}^* = 1$). For h_1, it belongs to class DIII. However, h_2 has the symmetry conditions as:

$$\begin{aligned} U_{\mathcal{T},+}h_2^*(\boldsymbol{k})U_{\mathcal{T},+}^{-1} &= -h_2(-\boldsymbol{k}) \\ U_{C,-}h_2^*(\boldsymbol{k})U_{C,-}^{-1} &= -h_2(-\boldsymbol{k}) \end{aligned} \tag{S71}$$

The above symmetry conditions imply the following relation

$$[h_2, U_{\mathcal{T},+}U_{C,-}^*] = 0 \tag{S72}$$

meaning that the h_2 can be block diagonalized by the eigenvectors of $U_{\mathcal{T},+}U_{C,-}^*$. These blocks are not independent but related to each other through the remaining symmetry conditions. If h_2 is in class AII ($\mathcal{T}_+$ symmetry), the additional symmetry $U_{\mathcal{T},+}U_{C,-}^*$ will change the symmetry class of h_2 from class AII to class A. If h_2 is in class D (C_- symmetry), the additional symmetry $U_{\mathcal{T},+}U_{C,-}^*$ will change the symmetry class of h_2 from class D to class A. Thus, the Hermitian parts of a non-Hermitian Hamiltonian in class DIII are in two different symmetry classes: h_1 in class DIII, but h_2 in class A. The symmetry classes of non-Hermitian systems which have the complexification can be found in Table. S4.

2. *Emergent symmetry*

In Table. S1, we can see that the Hermitian part h_1 shares the same symmetry conditions as the non-Hermitian H, but the symmetry conditions for h_2 can be distinct. Because an antiunitary symmetry operator that applies on ih_2 can lead to a totally different symmetry condition, meaning that an emergent symmetry appears for h_2. Here, we will show some cases to show the emergent symmetry for h_2.

Class D†. The Hamiltonian H in class D† describes a non-Hermitian time-reversal broken superconductor, which has the following symmetry condition

$$U_{C,-}H^*(\boldsymbol{k})U_{C,-}^{-1} = -H(-\boldsymbol{k}), \quad U_{C,-}U_{C,-}^* = 1 \tag{S73}$$

Thus, h_2 will have the following symmetry

$$U_{C,-}h_2^*(\boldsymbol{k})U_{C,-}^{-1} = h_2(-\boldsymbol{k}) \tag{S74}$$

reflecting that h_2 can have an emergent spinless time-reversal symmetry and thus belongs to class AI.

Class BDI. The Hamiltonian H in class BDI has the following symmetry conditions:

$$\begin{aligned} U_{\mathcal{T},+}H^*(\boldsymbol{k})U_{\mathcal{T},+}^{-1} &= H(-\boldsymbol{k}), \quad U_{\mathcal{T},+}U_{\mathcal{T},+}^* = 1 \\ U_{C,+}H^T(\boldsymbol{k})U_{C,+}^{-1} &= -H(-\boldsymbol{k}), \quad U_{C,+}U_{C,+}^* = 1 \end{aligned} \tag{S75}$$

Thus, h_2 will have the following symmetry conditions:

$$\begin{aligned} U_{\mathcal{T},+}h_2^*(\boldsymbol{k})U_{\mathcal{T},+}^{-1} &= -h_2(-\boldsymbol{k}), \quad U_{\mathcal{T},+}U_{\mathcal{T},+}^* = 1 \\ U_{C,+}h_2^*(\boldsymbol{k})U_{C,+}^{-1} &= -h_2(-\boldsymbol{k}), \quad U_{C,+}U_{C,+}^* = 1 \end{aligned} \tag{S76}$$

Clearly, h_2 preserves the particle-hole symmetry and belongs to class D. But importantly, it also has an emergent pseudo-Hermiticity symmetry, i.e., $U_\eta h_2^\dagger U_\eta^{-1} = h_2$, $U_\eta = U_{\mathcal{T},+}U_{C,+}$. Note that because $U_\eta \neq 1$ and $[U_\eta, h_2] = 0$, U_η can block diagonalize h_2.

3. *Dimension increment*

For obtaining any d-dimensional non-Hermitian Hamiltonian H^d, we can firstly obtain two d-dimensional Hermitian parts h_1^d and h_2^d under symmetry conditions according to Ref. [1, 6, 34]. Then we construct the d-dimensional non-Hermitian Hamiltonian based on Eq. S68, namely $H^d = h_1^d + ih_2^d$.

4. *Hermitian flattening*

Hermitian flattening means that we continuously deform the non-Hermitian Hamiltonian to a topologically equivalent Hermitian Hamiltonian. Although the topological classifications of non-Hermitian Hamiltonians don't necessitate the Hermitian flattening, Hermitian flattening can speed up the computations.

According to Section. Sec. I A, for the point-gap topology, the topological classification of H with a point gap and

TABLE S3. Symmetry operations for two Hermitian parts h_1 and h_2 of a non-Hermitian Hamiltonian $H = h_1 + ih_2$.

Symmetry	H	h_1	h_2
$\mathcal{T}_+$	$U_{\mathcal{T},+}H^*(\boldsymbol{k})U_{\mathcal{T},+}^{-1} = H(-\boldsymbol{k})$	$U_{\mathcal{T},+}h_1^*(\boldsymbol{k})U_{\mathcal{T},+}^{-1} = h_1(-\boldsymbol{k})$	$U_{\mathcal{T},+}h_2^*(\boldsymbol{k})U_{\mathcal{T},+}^{-1} = -h_2(-\boldsymbol{k})$
$\mathcal{T}_-$	$U_{\mathcal{T},-}H^T(\boldsymbol{k})U_{\mathcal{T},-}^{-1} = H(-\boldsymbol{k})$	$U_{\mathcal{T},-}h_1^T(\boldsymbol{k})U_{\mathcal{T},-}^{-1} = h_1(-\boldsymbol{k})$	$U_{\mathcal{T},-}h_2^T(\boldsymbol{k})U_{\mathcal{T},-}^{-1} = h_2(-\boldsymbol{k})$
C_+	$U_{C,+}H^T(\boldsymbol{k})U_{C,+}^{-1} = -H(-\boldsymbol{k})$	$U_{C,+}h_1^T(\boldsymbol{k})U_{C,+}^{-1} = -h_1(-\boldsymbol{k})$	$U_{C,+}h_2^T(\boldsymbol{k})U_{C,\pm}^{-1} = -h_2(-\boldsymbol{k})$
C_-	$U_{C,-}H^*(\boldsymbol{k})U_{C,-}^{-1} = -H(-\boldsymbol{k})$	$U_{C,-}h_1^T(\boldsymbol{k})U_{C,-}^{-1} = -h_1(-\boldsymbol{k})$	$U_{C,-}h_2^*(\boldsymbol{k})U_{C,\pm}^{-1} = h_2(-\boldsymbol{k})$
Γ	$U_\Gamma H^\dagger(\boldsymbol{k})U_\Gamma^{-1} = -H(\boldsymbol{k})$	$U_\Gamma h_1(\boldsymbol{k})U_\Gamma^{-1} = -h_1(\boldsymbol{k})$	$U_\Gamma h_2(\boldsymbol{k})U_\Gamma^{-1} = h_2(\boldsymbol{k})$
$\mathcal{S}$	$U_\mathcal{S}H(\boldsymbol{k})U_\mathcal{S}^{-1} = -H(\boldsymbol{k})$	$U_\mathcal{S}h_1(\boldsymbol{k})U_\mathcal{S}^{-1} = -h_1(\boldsymbol{k})$	$U_\mathcal{S}h_2(\boldsymbol{k})U_\mathcal{S}^{-1} = -h_2(\boldsymbol{k})$
η	$U_\eta H^\dagger(\boldsymbol{k})U_\eta^{-1} = H(\boldsymbol{k})$	$U_\eta h_1(\boldsymbol{k})U_\eta^{-1} = h_1(\boldsymbol{k})$	$U_\eta h_2(\boldsymbol{k})U_\eta^{-1} = -h_2(\boldsymbol{k})$

symmetry can reduce to that of a Hermitian Hamiltonian $\widetilde{H} = \begin{pmatrix} 0 & H \\ H^\dagger & 0 \end{pmatrix}$ with the corresponding symmetry constraints. The topological classification of H with a point gap can correspond to that of $\widetilde{H}$ with sublattice symmetry.

According to Section. Sec. I B, for the line-gap topology, if there is a real (imaginary) line gap for H, H can continuously deform into a Hermitian(anti-Hermitian) Hamiltonian while keeping a real (imaginary) gap and its symmetry, meaning that H shares the same topology as a Hermitian(anti-Hermitian) Hamiltonian. Because two Hermitian parts h_1 and h_2 are randomly generated, we can regard that h_1 and h_2 can access all topological phases under the corresponding symmetry conditions. Thereby, the topological classification of H with a real line gap can correspond to that of h_1, while the topological classification of H with an imaginary line gap can correspond to that of h_2.

5. *Effect of long-range hopping*

The long-range hopping can introduce more topological phases without increasing the number of bands. For example, in the Hatano-Nelson model of Eq. S19, the long-range hopping can lead to a large winding number on the complex energy plane. As we mentioned before, the topological classification of H can have one-by-one mapping with a Hermitian Hamiltonian after performing Hermitian flattening. However, adding bands without long-range hopping for Hermitian systems can access all possible topological phases obtained only by long-range hopping [1]. Namely, introducing long-rang hopping has no extra contributions for topological classifications obtained by increasing the number of bands. Moreover, it is invalid for 0D non-Hermitian Hamiltonians to introduce long-range hopping. Thus, for the simplicity, we can exploit to increase the number of bands to access all phases, instead of introducing long-range hopping.

TABLE S4. The corresponding Hermitian symmetry classes for the Hermitian parts h_1 and h_2 in different non-Hermitian symmetry classes. The label η denotes an emergent pseudo-Hermiticity symmetry. The symmetry classes denoted in red color are redundant due to the symmetry unification.

H	h_1	h_2
A	A	A
AIII	AIII	A + η
AI	AI	D
BDI	BDI	D+η
D	D	D
DIII	DIII	A
AII	AII	C
CII	CII	C + η
C	C	C
CI	CI	A
AI†	AI	AI
BDI†	BDI	AI + η
D†	D	AI
DIII†	DIII	A
AII†	AII	AII
CII†	CII	AII + η
C†	C	AII
CI†	CI	A

Sec. V. UNSUPERVISED TOPOLOGICAL CLASSIFICATIONS OF NON-HERMITIAN TOPOLOGICAL SYSTEMS UNDER SYMMETRIES

In this section, we show the unsupervised topological classification results obtain by our algorithms.

A. The number of phases as a function of the number of bands

Here, we discuss the relation between the number of phases and the number of bands. We take 0D Hamiltonians as an example. according to Section Sec. IV, we randomly generate 0D Hamiltonian samples with different number of bands n for the line-gap topology and $\frac{n}{2}$ for the point-gap topology (because the construction of $\widetilde{H}$ will double the number of bands).

TABLE S5. The correspond Hermitian symmetry classes for the Hermitian parts h_1 and h_2 of a non-Hermitian Hamiltonian with sublattice symmetry $\mathcal{S}$. The subscript of $\mathcal{S}_\pm$ specifies the commutation (+) or anticommutation (−) relation to $\mathcal{T}$ symmetry and/or C symmetry. For the symmetry classes that contain both $\mathcal{T}$ symmetry and C symmetry, the first and second subscript denote the relation to $\mathcal{T}$ symmetry and C symmetry, respectively. The label η denotes an emergent pseudo-Hermiticity symmetry. The symmetry classes denoted in red color are redundant due to the symmetry unification.

H	h_1	h_2
A + $\mathcal{S}$	AIII	AIII
AIII + $\mathcal{S}_+$	AIII + η	AIII+η
AIII + $\mathcal{S}_-$	A	A
BDI + $\mathcal{S}_{++}$	BDI + η	BDI + η
DIII + $\mathcal{S}_{--}$	DIII + η	AIII
CII + $\mathcal{S}_{++}$	CII+ η	CII+η
CI + $\mathcal{S}_{--}$	CI + η	AIII
AI + $\mathcal{S}_-$	CI	DIII
BDI + $\mathcal{S}_{-+}$	AI	D
D + $\mathcal{S}_+$	BDI	BDI
CII + $\mathcal{S}_{-+}$	AII	C
C + $\mathcal{S}_+$	CII	CII
DIII + $\mathcal{S}_{++}$	AIII	AIII
CI + $\mathcal{S}_{++}$	AIII	AIII
AI + $\mathcal{S}_+$	BDI	BDI
BDI + $\mathcal{S}_{+-}$	D	D
D + $\mathcal{S}_-$	DIII	DIII
DIII + $\mathcal{S}_{+-}$	AII	AII
AII + $\mathcal{S}_+$	CII	CII
CII + $\mathcal{S}_{+-}$	C	C
C+$\mathcal{S}_-$	CI	CI
CI+$\mathcal{S}_{+-}$	AI	AI
DIII+$\mathcal{S}_{-+}$	D	AI
AII + $\mathcal{S}_-$	DIII	CI
CI + $\mathcal{S}_{-+}$	C	AII
BDI + $\mathcal{S}_{--}$	AIII	DIII+η
CII + $\mathcal{S}_{--}$	AIII	CI+η

Here, we take the complex Fermi level as $E_f = 0$. We perform our algorithm to obtain the number of phases N_c. The learning results for n-banded Hamiltonians with different types of gap topology are calculated and plotted: (1) the point-gap topology in Fig. S18; (2) the real line-gap topology in Fig. S19 ; (3) the imaginary line-gap topology in Fig. S20. Compared with theoretical predictions in Ref. [3, 4, 31, 35], we can conclude the following mapping relations when the number of bands become infinite, namely $n \to \infty$:

$$\begin{aligned} N_c &= 1 \to \text{trivial group "0"} \\ N_c &= n+1 \to \mathbb{Z} \\ N_c &= \frac{n}{2}+1 \to 2\mathbb{Z} \\ N_c &= 2 \to \mathbb{Z}_2 \\ N_c &= \left(\frac{n}{2}+1\right)^2 \to \mathbb{Z}\oplus\mathbb{Z} \\ N_c &= \left(\frac{n}{4}+1\right)^2 \to 2\mathbb{Z}\oplus 2\mathbb{Z} \\ N_c &= 2\times 2 \to \mathbb{Z}_2\oplus\mathbb{Z}_2 \end{aligned} \tag{S77}$$

Some symmetry classes that have topological classifications like $\mathbb{Z}\oplus\mathbb{Z}$ and $2\mathbb{Z}\oplus 2\mathbb{Z}$ requires large number of samples, leading to time-consuming calculations (e.g., it took nearly three weeks to obtain Fig. S18 on a workstation with two AMD EPYC 7742 64-Core processors).

Note that 0D non-Hermitian systems can have the point-gap topology, because the topological phase transition for the point-gap topology is dependent on whether it will pass through the reference energy (i.e., complex Fermi level E_f) during the continuous deformation between Hamiltonians, no matter what the dimension the Hamiltonians have. Here, we take some 0D cases to illustrate the point.

Class A. Because the non-Hermitian system with only one band (e.g., 1D Hatano-Nelson system) can have the point-gap topology, we take the one-band system in class A as an example. According to Table. S1, the 0D Hamiltonian H in class A has no symmetry constraint. Thus, we have a general form of H as:

$$H = x + iy \tag{S78}$$

where $x, y \in \mathbb{R}$. Clearly, all the points on the complex-energy plane can be continuously connected without necessarily passing through $E_f = 0$. Consequently, the 0D Hamiltonians in class A with a point gap belong to a trivial group, which is consistent with the classification results in Fig. S18 obtained by our algorithm.

Class AIII. According to Table. S1, the 0D Hamiltonian H in class AIII has the Γ symmetry, $\Gamma H^\dagger \Gamma^{-1} = -H$. Namely, H has at least two bands. Here, we take a two-band Hamiltonian as an example. The general form for a two-band Hamiltonian H is represented as:

$$H = c_0 i\sigma_0 + c_1\sigma_x + c_2\sigma_y + c_3 i\sigma_z \tag{S79}$$

where $c_i \in \mathbb{R}$ and $\Gamma = \sigma_z$. We consider two Hamiltonians: $H_1 = \sigma_x + \sigma_y + i\sigma_z$ and $H_2 = -\sigma_x - \sigma_y - i\sigma_z$. The continuous deformation $H_\alpha = (1-\alpha)H_1 + \alpha H_2$ ($\alpha \in [0,1]$) has the eigen-energies $E_\pm = \pm(1-2\alpha)\sqrt{(c_1^2 + c_2^2 - c_3^2)}$, which will pass through E_f when $\alpha = 1/2$. If we introduce any Γ-symmetric perturbation ΔH into H_1 and H_2, e.g., $\Delta H = 0.05\sigma_x$ or $\Delta H = 0.1 i\sigma_z$, H_α will always pass through $E_f = 0$. This is consistent with the classification results in Fig. S18.

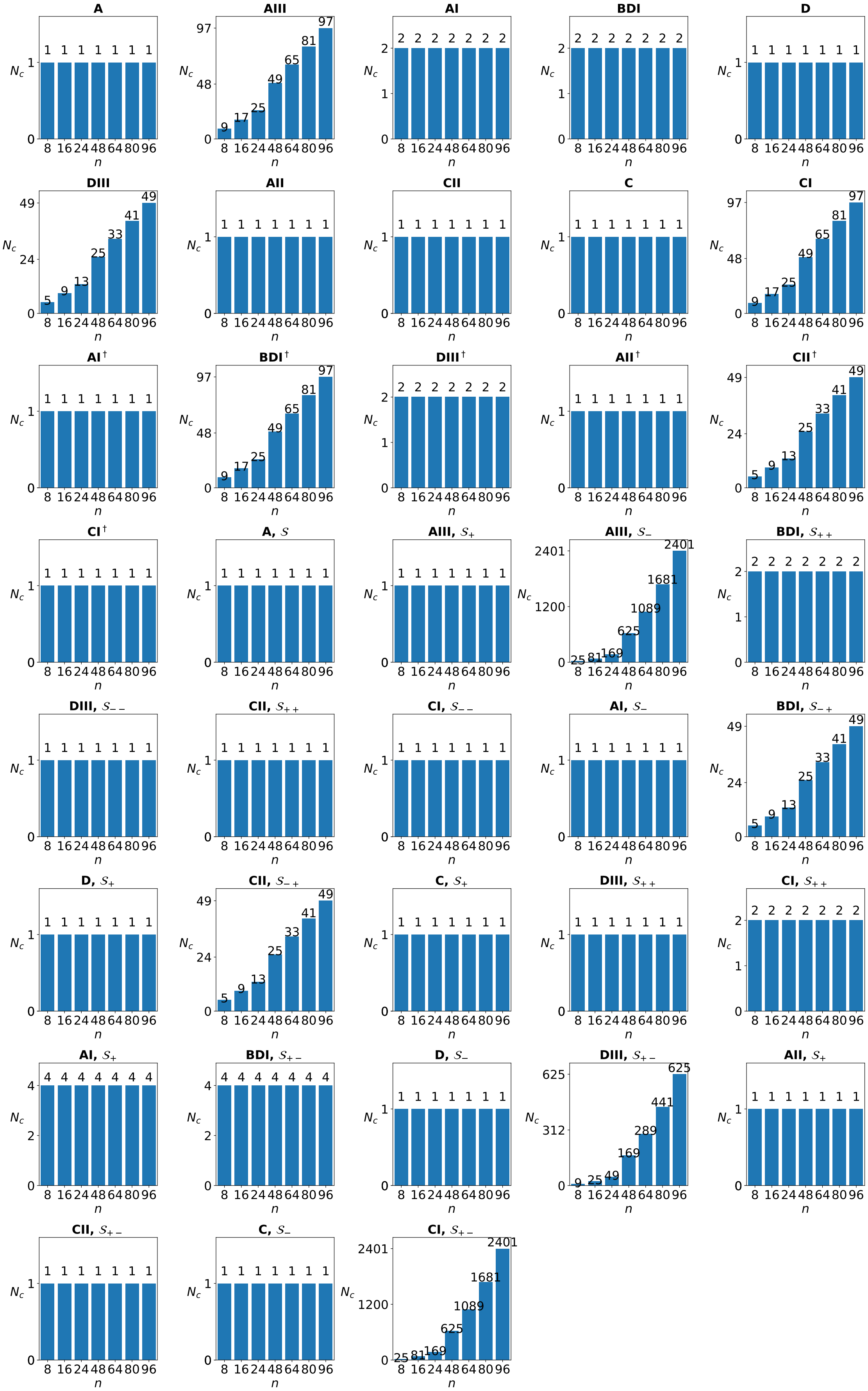

FIG. S18. The number of phases N_c as the function of the number of bands n for non-Hermitian systems with a point gap. The labels (black bold font) on the top are the non-Hermitian symmetry classes. The number on the top of each bar denotes N_c.

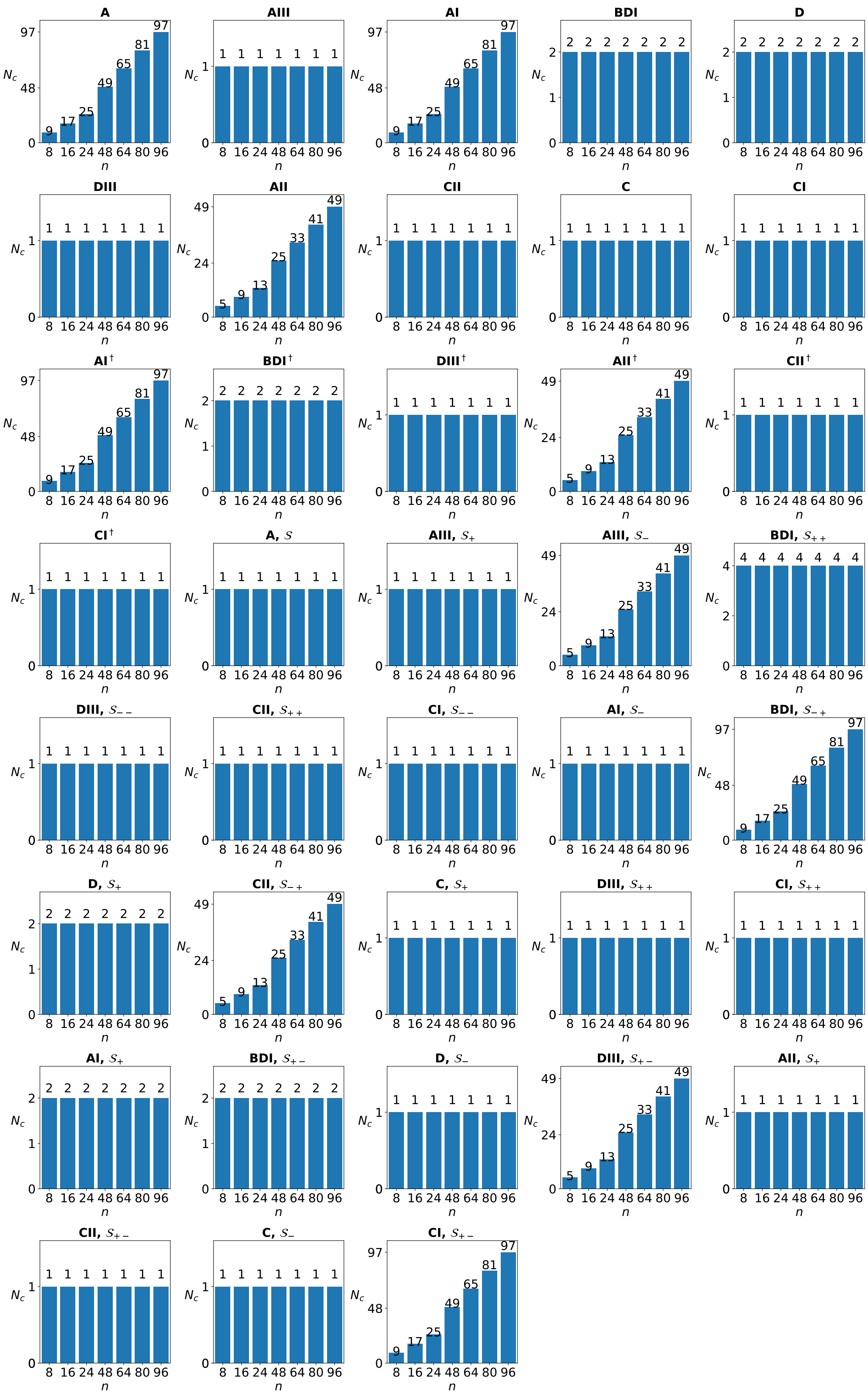

FIG. S19. The number of phases N_c as the function of the number of bands n for non-Hermitian systems with a real line gap. The labels (black bold font) on the top are the non-Hermitian symmetry classes. The number on the top of each bar denotes N_c.

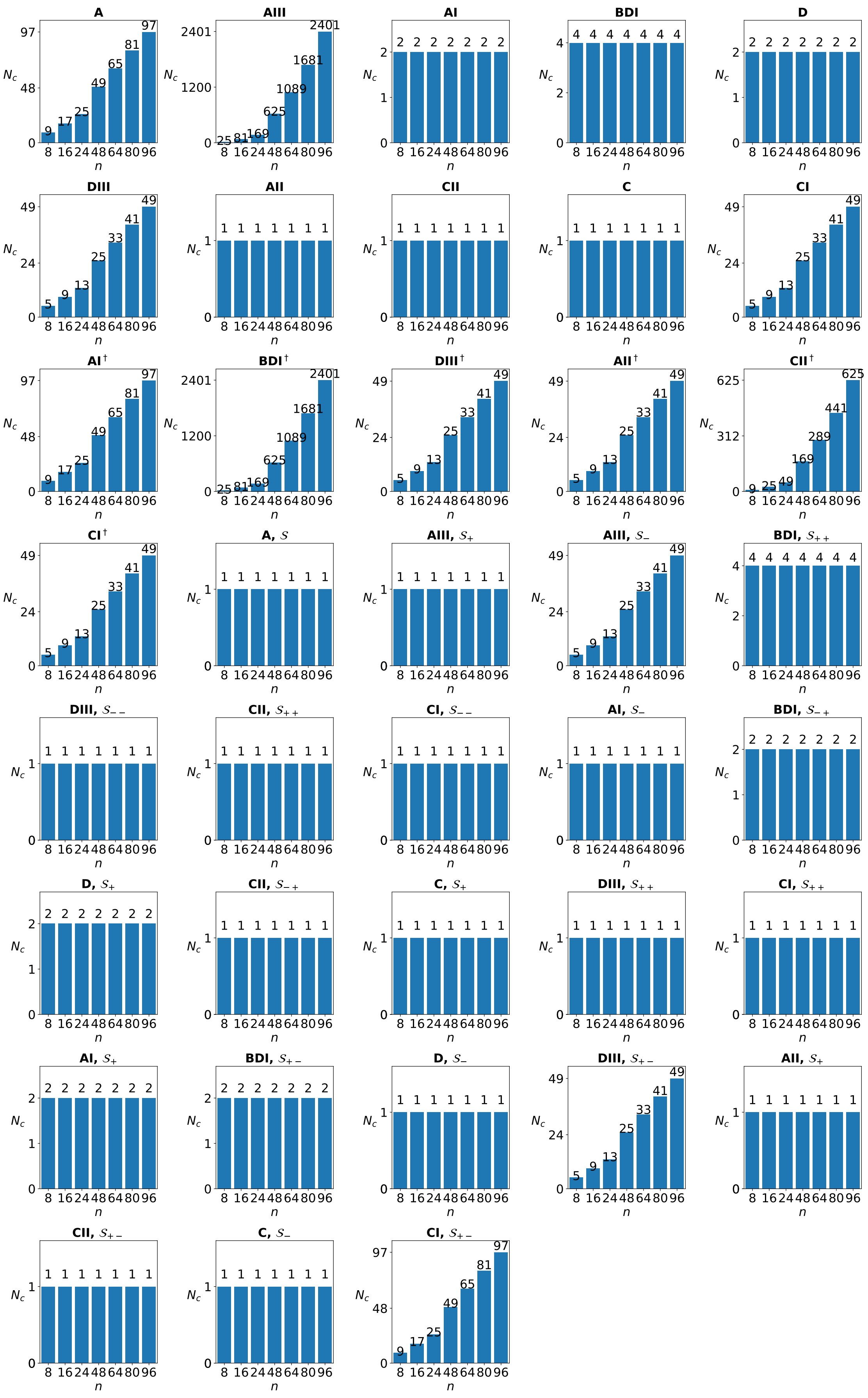

FIG. S20. The number of phases N_c as the function of the number of bands n for non-Hermitian systems with an imaginary line gap. The labels (black bold font) on the top are the non-Hermitian symmetry classes. The number on the top of each bar denotes N_c.

B. Classifications of non-Hermitian topological systems in different dimensions

Here, we show unsupervised classifications of non-Hermitian topological systems in the different dimensions. The dimension d changes from $d = 1$ to $d = 9$. The classification results of non-Hermitian topological systems with different dimensions that are obtained by our algorithm are concluded in Extended Table 1 of the main text. Here, for simplicity, we demonstrate the classification results of Hamiltonians that are generated from 0D Hamiltonians with the number of bands as $n = 8$.

The classifications for the Hamiltonians with a point gap are calculated and plotted: (1) $d = 1$ in Fig. S21; (2) $d = 2$ in Fig. S22; (3) $d = 3$ in Fig. S23; (4) $d = 4$ in Fig. S24; (5) $d = 5$ in Fig. S25; (6) $d = 6$ in Fig. S26; (7) $d = 7$ in Fig. S27; (8) $d = 8$ in Fig. S28; (9) $d = 9$ in Fig. S29.

The classifications for the Hamiltonians with a real line gap are calculated and plotted: (1) $d = 1$ in Fig. S30; (2) $d = 2$ in Fig. S31; (3) $d = 3$ in Fig. S32; (4) $d = 4$ in Fig. S33; (5) $d = 5$ in Fig. S34; (6) $d = 6$ in Fig. S35; (7) $d = 7$ in Fig. S36; (8) $d = 8$ in Fig. S37; (9) $d = 9$ in Fig. S38.

The classifications for the Hamiltonians with an imaginary line gap are calculated and plotted: (1) $d = 1$ in Fig. S39; (2) $d = 2$ in Fig. S40; (3) $d = 3$ in Fig. S41; (4) $d = 4$ in Fig. S42; (5) $d = 5$ in Fig. S43; (6) $d = 6$ in Fig. S44; (7) $d = 7$ in Fig. S45; (8) $d = 8$ in Fig. S46; (9) $d = 9$ in Fig. S47.

Sec. VI. NON-HERMITIAN SYMMETRY CLASSES WITH PARITY TRANSFORMATION

In this section, we discuss the non-Hermitian symmetries combining with the parity transformation ($\mathcal{P} : \boldsymbol{r} \to -\boldsymbol{r}$) and their topological classification. The parity symmetry can serve as an additional symmetry to symmetry classes, like $\mathcal{S}$ symmetry, which can dramatically enrich symmetry classes [33, 36, 37]. However, in our work, instead of considering the parity symmetry($\mathcal{P}$ symmetry) as an additional symmetry, we consider to combine the parity transformation with the well-known symmetry operators, like the famous parity-time ($\mathcal{PT}$) symmetry and parity-particle-hole ($\mathcal{PC}$) symmetry. Based on $\mathcal{PT}$ symmetry and $\mathcal{PC}$ symmetry, we can produce a topological periodic table analogous to the one formed by $\mathcal{T}$ symmetry and C symmetry. In the following, we show the details about $\mathcal{PT}$ symmetry and $\mathcal{PC}$ symmetry, the generation scheme of random Hamiltonian samples, and the classification results.

A. $\mathcal{PT}$ symmetry and $\mathcal{PC}$ symmetry

Because of the non-Hermiticity, $\mathcal{PT}$ symmetry and $\mathcal{PC}$ symmetry for non-Hermitian Hamiltonians can also have two types. For $\mathcal{PT}$ symmetry, it has two symmetry conditions:

$$\begin{aligned} U_{\mathcal{PT},+}H^*(\boldsymbol{k})U_{\mathcal{PT},+}^{-1} &= H(\boldsymbol{k}), \quad U_{\mathcal{PT},+}U_{\mathcal{PT},+}^* = \pm 1 \\ U_{\mathcal{PT},-}H^T(\boldsymbol{k})U_{\mathcal{PT},-}^{-1} &= H(\boldsymbol{k}), \quad U_{\mathcal{PT},-}U_{\mathcal{PT},-}^* = \pm 1 \end{aligned} \tag{S80}$$

TABLE S6. $\mathcal{P}$AZ and $\mathcal{P}$AZ† symmetry classes for non-Hermitian Hamiltonians. The sign ± 1 corresponds to $U_{\mathcal{PT},\pm}U_{\mathcal{PT},\pm}^* = \pm 1$ or $U_{\mathcal{PC},\pm}U_{\mathcal{PC},\pm}^* = \pm 1$. The value 1 and 0 mean $U_\Gamma^2 = 1$ and no corresponding symmetry, respectively.

Symmetry class	$\mathcal{PT}_+$	$\mathcal{PC}_+$	$\mathcal{PT}_-$	$\mathcal{PC}_-$	Γ
A	0	0	0	0	0
AIII	0	0	0	0	1
$\mathcal{P}$AI	+1	0	0	0	0
$\mathcal{P}$BDI	+1	+1	0	0	1
$\mathcal{P}$D	0	+1	0	0	0
$\mathcal{P}$DIII	-1	+1	0	0	1
$\mathcal{P}$AII	-1	0	0	0	0
$\mathcal{P}$CII	-1	-1	0	0	1
$\mathcal{P}$C	0	-1	0	0	0
$\mathcal{P}$CI	+1	-1	0	0	1
$\mathcal{P}$AI†	0	0	+1	0	0
$\mathcal{P}$BDI†	0	0	+1	+1	1
$\mathcal{P}$D†	0	0	0	+1	0
$\mathcal{P}$DIII†	0	0	-1	+1	1
$\mathcal{P}$AII†	0	0	-1	0	0
$\mathcal{P}$CII†	0	0	-1	-1	1
$\mathcal{P}$C†	0	0	0	-1	0
$\mathcal{P}$CI†	0	0	+1	-1	1

where $U_{\mathcal{PT},+}$ is the $\mathcal{PT}_\pm$ symmetry operator. Although a system that preserves $\mathcal{P}$ symmetry and $\mathcal{T}$ symmetry also has $\mathcal{PT}$ symmetry, a system that has $\mathcal{PT}$ symmetry can not have $\mathcal{P}$-symmetry or $\mathcal{T}$ symmetry. Thus, $\mathcal{PT}$-symmetry can be regarded as a new symmetry condition different from $\mathcal{P}$ symmetry and $\mathcal{T}$ symmetry [38, 39]. Similarly, for $\mathcal{PT}$ symmetry, it has two symmetry conditions:

$$\begin{aligned} U_{\mathcal{PC},+}H^T(\boldsymbol{k})U_{\mathcal{PC},+}^{-1} &= -H(\boldsymbol{k}), \quad U_{\mathcal{PC},+}U_{\mathcal{PC},+}^* = \pm 1 \\ U_{\mathcal{PC},-}H^*(\boldsymbol{k})U_{\mathcal{PC},-}^{-1} &= -H(\boldsymbol{k}), \quad U_{\mathcal{PC},-}U_{\mathcal{PC},-}^* = \pm 1 \end{aligned} \tag{S81}$$

where $U_{\mathcal{PC},+}$ is the $\mathcal{PC}_\pm$ symmetry operator.

Based on $\mathcal{PT}$ symmetry and $\mathcal{PC}$ symmetry, we can have the chiral symmetry $\Gamma = (\mathcal{PT})\mathcal{PC}$, which is identical to the chiral symmetry without the parity transformation. Following the similar analysis, we can obtain AZ and AZ† classes with the parity transformation (parity-equipped symmetry classes), as shown in Fig. S6, which we will call as $\mathcal{P}$AZ and $\mathcal{P}$AZ†, respectively. The effect of the parity transformation is to reverse the momentum. Thus, with consider $\mathcal{S}$ symmetry, we can also obtain 38 symmetry classes [40]. The equivalent symmetry classes are listed in Table. S7.

B. Generation of Hamiltonian samples

According to the decomposition of Eq. S68, we can generate two Hermitian parts h_1 and h_2 firstly. For $\mathcal{PT}_+$ symmetry,

TABLE S7. Equivalent symmetry classes for non-Hermitian Hamiltonians. The subscript of $\mathcal{S}_{\pm}$ specifies the commutation (+) or anticommutation (−) relation to $\mathcal{PT}$ symmetry and/or $\mathcal{PC}$ symmetry. For the symmetry classes that contain both $\mathcal{PT}$ symmetry and $\mathcal{PC}$ symmetry, the first and second subscript denote the relation to $\mathcal{PT}$ symmetry and $\mathcal{PC}$ symmetry, respectively.

Symmetry class	Equivalent symmetry class
$\mathcal{P}$D†	$\mathcal{P}$AI
$\mathcal{P}$C†	$\mathcal{P}$AII
$\mathcal{P}$DIII + $\mathcal{S}_{-+}$	BDI + $\mathcal{S}_{-+}$
$\mathcal{P}$AII + $\mathcal{S}_{-}$	$\mathcal{P}$AI + $\mathcal{S}_{-}$
$\mathcal{P}$CI + $\mathcal{S}_{-+}$	$\mathcal{P}$CII + $\mathcal{S}_{-+}$
$\mathcal{P}$BDI + $\mathcal{S}_{--}$	$\mathcal{P}$DIII + $\mathcal{S}_{--}$
$\mathcal{P}$CII + $\mathcal{S}_{--}$	$\mathcal{P}$CI + $\mathcal{S}_{--}$

we can have

$$\begin{aligned} U_{\mathcal{PT},+}h_1^*(\boldsymbol{k})U_{\mathcal{PT},+}^{-1} &= h_1(\boldsymbol{k}) \\ U_{\mathcal{PT},+}h_2^*(\boldsymbol{k})U_{\mathcal{PT},+}^{-1} &= -h_2(\boldsymbol{k}). \end{aligned} \tag{S82}$$

For $\mathcal{PT}_-$ symmetry, we can have

$$\begin{aligned} U_{\mathcal{PT},-}h_1^*(\boldsymbol{k})U_{\mathcal{PT},-}^{-1} &= h_1(\boldsymbol{k}) \\ U_{\mathcal{PT},-}h_2^*(\boldsymbol{k})U_{\mathcal{PT},-}^{-1} &= h_2(\boldsymbol{k}) \end{aligned} \tag{S83}$$

For $\mathcal{PC}_+$ symmetry, we can have

$$\begin{aligned} U_{\mathcal{PC},+}h_1^*(\boldsymbol{k})U_{\mathcal{PC},+}^{-1} &= -h_1(\boldsymbol{k}) \\ U_{\mathcal{PC},+}h_2^*(\boldsymbol{k})U_{\mathcal{PC},+}^{-1} &= -h_2(\boldsymbol{k}) \end{aligned} \tag{S84}$$

For $\mathcal{PC}_-$ symmetry, we can have

$$\begin{aligned} U_{\mathcal{PC},-}h_1^*(\boldsymbol{k})U_{\mathcal{PC},-}^{-1} &= -h_1(\boldsymbol{k}) \\ U_{\mathcal{PC},-}h_2^*(\boldsymbol{k})U_{\mathcal{PC},-}^{-1} &= h_2(\boldsymbol{k}) \end{aligned} \tag{S85}$$

We conclude the above symmetry conditions for h_1 and h_2 in Table. S8.

Because there is no parity transformation (i.e., $\boldsymbol{r} \to -\boldsymbol{r}$) for 0D Hamiltonians, the dimension for $\mathcal{P}$AZ classes, $\mathcal{P}$AZ† classes, and their combination with $\mathcal{S}$ symmetry starts from 1D. To construct the Hamiltonians in higher dimensions $d \geq 1$, we increase dimensions from 0D Hamiltonians based on the scheme in Refs. [1, 6, 34], which is described in the following:

For a d-dimensional Hamiltonian h^d, its dimension is increased from d to $d+1$, according to whether h^d has chiral symmetry: (1) If h^d has the chiral symmetry Γ, the new higher-dimensional Hamiltonian h^{d+1} will be:

$$h^{d+1} = h^d \cos(k_{d+1}) - \Gamma \sin(k_{d+1}) \tag{S86}$$

(2) If h^d does not satisfy the chiral symmetry Γ, h^{d+1} will be:

$$h^{d+1} = h^d \cos(k_{d+1})\tau_x - \sin(k_{d+1})\tau_y \tag{S87}$$

This operation doubles the number of bands of H_d but not change its topological classification. After obtaining the d-dimensional Hermitian parts h_1^d and h_2^d, we can have the d-dimensional non-Hermitian Hamiltonian $H^d = h_1^d + ih_2^d$.

C. Classifications of non-Hermitian topological systems with the parity transformation in different dimensions

Here, we show unsupervised classifications of non-Hermitian topological systems with the parity transformation in different dimensions. The dimension d changes from $d = 1$ to $d = 10$. The classification results that are obtained by our algorithm are concluded in Extended Table 2 of the main text. Here, for simplicity, we demonstrate the classification results of Hamiltonians that are generated from 0D Hamiltonians with the number of bands as $n = 8$.

The classifications for the Hamiltonians with a point gap are calculated and plotted: (1) $d = 1$ in Fig. S48; (2) $d = 2$ in Fig. S49; (3) $d = 3$ in Fig. S50; (4) $d = 4$ in Fig. S51; (5) $d = 5$ in Fig. S52; (6) $d = 6$ in Fig. S53; (7) $d = 7$ in Fig. S54; (8) $d = 8$ in Fig. S55; (9) $d = 9$ in Fig. S56; (10) $d = 10$ in Fig. S57.

The classifications for the Hamiltonians with a real line gap are calculated and plotted: (1) $d = 1$ in Fig. S58; (2) $d = 2$ in Fig. S59; (3) $d = 3$ in Fig. S60; (4) $d = 4$ in Fig. S61; (5) $d = 5$ in Fig. S62; (6) $d = 6$ in Fig. S63; (7) $d = 7$ in Fig. S64; (8) $d = 8$ in Fig. S65; (9) $d = 9$ in Fig. S66; (10) $d = 10$ in Fig. S67.

The classifications for the Hamiltonians with an imaginary line gap are calculated and plotted: (1) $d = 1$ in Fig. S68; (2) $d = 2$ in Fig. S69; (3) $d = 3$ in Fig. S70; (4) $d = 4$ in Fig. S71; (5) $d = 5$ in Fig. S72; (6) $d = 6$ in Fig. S73; (7) $d = 7$ in Fig. S74; (8) $d = 8$ in Fig. S75; (9) $d = 9$ in Fig. S76; (10) $d = 10$ in Fig. S77.

D. Corresponding relations between the topological classifications of non-Hermitian Hamiltonians with and without parity transformation

Clearly, the topological classifications of non-Hermitian Hamiltonians with parity transformation have a corresponding relation to those without parity transformation, namely,

$$K_d^{\mathcal{P}}(s^{\mathcal{P}}) = K_{8-d}(s) \tag{S88}$$

where $K_d(s)$ denotes the topological classification of d-dimensional non-Hermitian systems in the symmetry class s, the subscript $\mathcal{P}$ denotes the classification and the corresponding symmetry class after performing $\mathcal{P}$. For example, if s means class AII, $s^{\mathcal{P}}$ will correspond to $\mathcal{P}$AII. The relation of Eq. S88 shows that the d-dimensional Hamiltonians in the symmetry class $s^{\mathcal{P}}$ have the same topological classifications as the $(8-d)$-dimensional Hamiltonians in the symmetry class s.

Here, we give a brief discussion of Eq. S88 based on Clifford algebra [41]. Since non-Hermitian topology depends on the types of complex-energy gaps, the representation of non-Hermitian Hamiltonian by Dirac matrices depends on the type of complex-energy gaps. Here, without loss of generality, the reference point (i.e., complex Fermi level) is set as $E_f = 0$. For the line-gap topology, a non-Hermitian Hamiltonian can continuously deform to a Hermitian Hamiltonian without closing the gap. For the point-gap topology, we can construct $\widetilde{H}$ of Eq. S1 instead. Namely, we can analyze the

TABLE S8. Symmetry operations on the two Hermitian parts h_1 and h_2 of a non-Hermitian Hamiltonian $H = h_1 + ih_2$.

Symmetry	H	h_1	h_2
$\mathcal{PT}_+$	$U_{\mathcal{PT},+}H^*(\boldsymbol{k})U_{\mathcal{PT},+}^{-1} = H(\boldsymbol{k})$	$U_{\mathcal{PT},+}h_1^*(\boldsymbol{k})U_{\mathcal{PT},+}^{-1} = h_1(\boldsymbol{k})$	$U_{\mathcal{PT},+}h_2^*(\boldsymbol{k})U_{\mathcal{PT},+}^{-1} = -h_2(\boldsymbol{k})$
$\mathcal{PT}_-$	$U_{\mathcal{PT},-}H^T(\boldsymbol{k})U_{\mathcal{PT},-}^{-1} = H(\boldsymbol{k})$	$U_{\mathcal{PT},-}h_1^T(\boldsymbol{k})U_{\mathcal{PT},-}^{-1} = h_1(\boldsymbol{k})$	$U_{\mathcal{PT},-}h_2^T(\boldsymbol{k})U_{\mathcal{PT},-}^{-1} = h_2(\boldsymbol{k})$
$\mathcal{PC}_+$	$U_{\mathcal{PC},+}H^T(\boldsymbol{k})U_{\mathcal{PC},+}^{-1} = -H(\boldsymbol{k})$	$U_{\mathcal{PC},+}h_1^T(\boldsymbol{k})U_{\mathcal{PC},+}^{-1} = -h_1(\boldsymbol{k})$	$U_{\mathcal{PC},+}h_2^T(\boldsymbol{k})U_{\mathcal{PC},\pm}^{-1} = -h_2(\boldsymbol{k})$
$\mathcal{PC}_-$	$U_{\mathcal{PC},-}H^*(\boldsymbol{k})U_{\mathcal{PC},-}^{-1} = -H(\boldsymbol{k})$	$U_{\mathcal{PC},-}h_1^T(\boldsymbol{k})U_{\mathcal{PC},-}^{-1} = -h_1(\boldsymbol{k})$	$U_{\mathcal{PC},-}h_2^*(\boldsymbol{k})U_{\mathcal{PC},\pm}^{-1} = h_2(\boldsymbol{k})$

topological classification based on a Hermitian Hamiltonian H (or $\widetilde{H}$) under the same symmetries as the original non-Hermitian Hamiltonian.

For a Hermitian Hamiltonian H, we can consider its low-energy description near the relevant momentum point $\boldsymbol{k}_0$, which generally takes the following form [6]:

$$H = \sum_i k_i \gamma_i + m\gamma_0 \tag{S89}$$

where $\boldsymbol{k} = (k_1, ..., k_d)$ is the momentum deviation from $\boldsymbol{k}_0$, $m \in \mathbb{R}$ is the mass, and $\{\gamma_i\}$ are Dirac matrices that satisfy the Clifford relation $\{\gamma_i, \gamma_j\} = 2\delta_{ij}$. For Clifford algebras, a complex Clifford algebra Cl_n has n generators e_i, having a relation that $\{e_i, e_j\} = 2\delta_{ij}$, while a real Clifford algebra $Cl_{m,n}$ has $m + n$ generators satisfying

$$\{e_i, e_j\} = 0 \quad (i \neq j)$$
$$e_i^2 = \begin{cases} -1, & 1 \leqslant i \leqslant p \\ 1, & p+1 \leqslant i \leqslant p+q \end{cases}. \tag{S90}$$

We express all kinetic terms $\{\gamma_i | i \neq 0\}$ and symmetry constraints as generators $\{e_i\}$ of a Clifford algebra. Then we extend the algebra by adding a mass term γ_0, which is also a generator for the Clifford algebra. The set of all possible representation of γ_0 forms a classifying space, which can be represented by C_q and R_q for complex and real symmetry classes. Here, we show some useful formulas about the relations between the extensions of Clifford algebra and their classifying spaces:

$$Cl_n \to Cl_{n+1} \Leftrightarrow C_n$$
$$Cl_{m,n} \to Cl_{m,n+1} \Leftrightarrow R_{n-m} \tag{S91}$$
$$Cl_{m,n} \to Cl_{m+1,n} \Leftrightarrow R_{m+2-n}$$

Finally, the topological classification of H of Eq. S89 can be obtained by a zero-th homotopy group of a classifying space: $\pi_0(C_q)$ or $\pi_0(R_q)$, denoting the number of disconnected parts of C_q or R_q [42, 43]. Since the derivations of topological classifications based on Clifford algebra are case-by-case, we focus on some cases to show the validity of Eq. S88 for simplicity.

Class AI and Class $\mathcal{P}$AI. We consider a d_1-dimensional non-Hermitian Hamiltonian H_{AI} under the class AI and a d_2-dimensional non-Hermitian Hamiltonian $H_{\mathcal{P}\mathrm{AI}}$ under the class $\mathcal{P}$AI. The symmetry conditions are listed below:

$$U_{\mathcal{T},+}H_{\mathrm{AI}}^*(\boldsymbol{k})U_{\mathcal{T},+}^{-1} = H_{\mathrm{AI}}(-\boldsymbol{k}), \quad U_{\mathcal{T},+}U_{\mathcal{T},+}^* = 1$$
$$U_{\mathcal{PT},+}H_{\mathcal{P}\mathrm{AI}}^*(\boldsymbol{k})U_{\mathcal{PT},+}^{-1} = H_{\mathcal{P}\mathrm{AI}}(\boldsymbol{k}), \quad U_{\mathcal{PT},+}U_{\mathcal{PT},+}^* = 1 \tag{S92}$$

Since the topological classification is dependent on the types of complex-energy gaps, we discuss the classification case-by-case in the following.

Real line-gap topology. Since both $\mathcal{T}$ and $\mathcal{PT}$ symmetry transformations involve complex conjugation $\mathcal{K}$, an operator J representing the imaginary unit "i" is introduced [43], satisfying $J^2 = -1$. For the class AI, a set of generators

$$\{; \gamma_1, ..., \gamma_{d_1}, U_{\mathcal{T},+}\mathcal{K}, JU_{\mathcal{T},+}\mathcal{K}\} \tag{S93}$$

forms a real Clifford algebra Cl_{0,d_1+2}. Since $\{U_{\mathcal{T},+}\mathcal{K}, J\gamma_0\} = 0$, the mass term can induce an extension $Cl_{0,d_1+2} \to Cl_{1,d_1+2}$, leading to the classifying space $R_{-d_1} \simeq R_{8-d_1}$. The real line-gap topology of the Hamiltonian in class AI is classified by $K_{d_1}(\mathrm{AI}) = \pi_0(R_{8-d_1})$. While, for the class $\mathcal{P}$AI, a set of generators

$$\{J\gamma_1, ..., J\gamma_{d_2}; U_{\mathcal{PT},+}\mathcal{K}, JU_{\mathcal{PT},+}\mathcal{K}\} \tag{S94}$$

forms a real Clifford algebra $Cl_{d_2,2}$. The mass term $J\gamma_0$ can induce an extension $Cl_{d_2,2} \to Cl_{d_2+1,2}$, leading to the classifying space R_{d_2}. The real line-gap topology of the Hamiltonian in class $\mathcal{P}$AI is classified by $K_{d_2}^{\mathcal{P}}(\mathcal{P}\mathrm{AI}) = \pi_0(R_{d_2})$. Since $R_{q+8} \simeq R_q$, for the real line gap, it is clear that $K_{d_2}^{\mathcal{P}}(\mathcal{P}\mathrm{AI}) = K_{d_1}(\mathrm{AI})$ when $d_1 = 8 - d_2$, corresponding to the relation of Eq. S88.

Imaginary line-gap topology. For the class AI, a set of generators

$$\{J\gamma_1, ..., J\gamma_{d_1}; U_{\mathcal{T},+}\mathcal{K}, JU_{\mathcal{T},+}\mathcal{K}\} \tag{S95}$$

forms a real Clifford algebra $Cl_{d_1,2}$. The mass term γ_0 can induce an extension $Cl_{d_1,2} \to Cl_{d_1,3}$, leading to the classifying space R_{2-d_1}. The imaginary line-gap topology of the Hamiltonian in class AI is classified by $K_{d_1}(\mathrm{AI}) = \pi_0(R_{2-d_1})$. While, for the class $\mathcal{P}$AI, a set of generators

$$\{; \gamma_1, ..., \gamma_{d_2}, U_{\mathcal{PT},+}\mathcal{K}, JU_{\mathcal{PT},+}\mathcal{K}\} \tag{S96}$$

forms a real Clifford algebra Cl_{0,d_2+2}. The mass term γ_0 can induce an extension $Cl_{0,d_2+2} \to Cl_{0,d_2+3}$, leading to the classifying space R_{d_2+2}. The imaginary line-gap topology of the Hamiltonian in class $\mathcal{P}$AI is classified by $K_{d_2}^{\mathcal{P}}(\mathcal{P}\mathrm{AI}) = \pi_0(R_{d_2+2})$. For the imaginary line gap, it is clear that $K_{d_2}^{\mathcal{P}}(\mathcal{P}\mathrm{AI}) = K_{d_1}(\mathrm{AI})$ when $d_1 = 8 - d_2$, corresponding to the relation of Eq. S88.

Point-gap topology. For point-gap topology, the topological classification is based on a Hermitian Hamiltonian $\widetilde{H}$ of Eq. S1, which respects an additional chiral symmetry $\Sigma = \sigma_z$ [4]. Both $\mathcal{T}$ and $\mathcal{PT}$ will be represented in new forms

$\widetilde{\mathcal{T}}$ and $\widetilde{\mathcal{PT}}$ (some details can be found in Sec. Sec. I A). For the class AI, a set of generators

$$\{J\Sigma;\gamma_1,...,\gamma_{d_1};\widetilde{U}_{\mathcal{T},+}\mathcal{K},J\widetilde{U}_{\mathcal{T},+}\mathcal{K}\} \tag{S97}$$

forms a real Clifford algebra Cl_{1,d_1+2}. The mass term $J\gamma_0$ can induce an extension $Cl_{1,d_1+2}\to Cl_{2,d_1+2}$, leading to the classifying space R_{1-d_1}. The point-gap topology of the Hamiltonian in class AI is classified by $K_{d_1}(\mathrm{AI})=\pi_0(R_{1-d_1})$. While, for the class $\mathcal{P}$AI, a set of generators

$$\{J\gamma_1,...,J\gamma_{d_2},J\Sigma;\widetilde{U}_{\mathcal{PT},+}\mathcal{K},J\widetilde{U}_{\mathcal{PT},+}\mathcal{K}\} \tag{S98}$$

forms a real Clifford algebra $Cl_{d_2+1,2}$. The mass term $J\gamma_0$ can induce an extension $Cl_{d_2+1,2}\to Cl_{d_2+2,2}$, leading to the classifying space R_{d_2+1}. The imaginary line-gap topology of the Hamiltonian in class $\mathcal{P}$AI is classified by $K^{\mathcal{P}}_{d_2}(\mathcal{P}\mathrm{AI})=\pi_0(R_{d_2+1})$. For the point gap, it is clear that $K^{\mathcal{P}}_{d_2}(\mathcal{P}\mathrm{AI})=K_{d_1}(\mathrm{AI})$ when $d_1=8-d_2$, corresponding to the relation of Eq. S88.

Class AII and Class $\mathcal{P}$AII. We consider a d_1-dimensional non-Hermitian Hamiltonian H_{AII} under the class AII and a d_2-dimensional non-Hermitian Hamiltonian $H_{\mathcal{P}\mathrm{AII}}$ under the class $\mathcal{P}$AII. The symmetry conditions are listed below:

$$\begin{aligned}U_{\mathcal{T},+}H^*_{\mathrm{AII}}(\boldsymbol{k})U^{-1}_{\mathcal{T},+}&=H_{\mathrm{AII}}(-\boldsymbol{k}), & U_{\mathcal{T},+}U^*_{\mathcal{T},+}&=-1\\ U_{\mathcal{PT},+}H^*_{\mathcal{P}\mathrm{AII}}(\boldsymbol{k})U^{-1}_{\mathcal{PT},+}&=H_{\mathcal{P}\mathrm{AII}}(\boldsymbol{k}), & U_{\mathcal{PT},+}U^*_{\mathcal{PT},+}&=-1\end{aligned} \tag{S99}$$

Real line-gap topology. For the class AII, a set of generators

$$\{U_{\mathcal{T},+}\mathcal{K},JU_{\mathcal{T},+}\mathcal{K};\gamma_1,...,\gamma_{d_1}\} \tag{S100}$$

forms a real Clifford algebra Cl_{2,d_1}. The mass term $J\gamma_0$ can induce an extension $Cl_{2,d_1}\to Cl_{3,d_1}$, leading to the classifying space R_{4-d_1}. The real line-gap topology of the Hamiltonian in class AI is classified by $K_{d_1}(\mathrm{AI})=\pi_0(R_{4-d_1})$. While, for the class $\mathcal{P}$AII, a set of generators

$$\{U_{\mathcal{PT},+}\mathcal{K},JU_{\mathcal{PT},+}\mathcal{K},J\gamma_1,...,J\gamma_{d_1};\} \tag{S101}$$

forms a real Clifford algebra $Cl_{d_2+2,0}$. The mass term $J\gamma_0$ can induce an extension $Cl_{d_2+2,0}\to Cl_{d_2+3,0}$, leading to the classifying space R_{4+d_2}. The real line-gap topology of the Hamiltonian in class $\mathcal{P}$AII is classified by $K^{\mathcal{P}}_{d_2}(\mathcal{P}\mathrm{AII})=\pi_0(R_{4+d_2})$. For the real line gap, it is clear that $K^{\mathcal{P}}_{d_2}(\mathcal{P}\mathrm{AII})=K_{d_1}(\mathrm{AII})$ when $d_1=8-d_2$, corresponding to the relation of Eq. S88.

Imaginary line-gap topology. For the class AII, a set of generators

$$\{J\gamma_1,...,J\gamma_{d_1},U_{\mathcal{T},+}\mathcal{K},JU_{\mathcal{T},+}\mathcal{K};\} \tag{S102}$$

forms a real Clifford algebra $Cl_{d_1+2,0}$. The mass term γ_0 can induce an extension $Cl_{d_1+2,0}\to Cl_{d_1+2,1}$, leading to the classifying space $R_{-2-d_1}\simeq R_{6-d_1}$. The imaginary line-gap topology of the Hamiltonian in class AII is classified by $K_{d_1}(\mathrm{AII})=\pi_0(R_{6-d_1})$. While, for the class $\mathcal{P}$AII, a set of generators

$$\{U_{\mathcal{PT},+}\mathcal{K},JU_{\mathcal{PT},+}\mathcal{K};\gamma_1,...,\gamma_{d_2}\} \tag{S103}$$

forms a real Clifford algebra Cl_{2,d_2}. The mass term γ_0 can induce an extension $Cl_{2,d_2}\to Cl_{2,d_2+1}$, leading to the classifying space R_{d_2-2}. The imaginary line-gap topology of the Hamiltonian in class $\mathcal{P}$AII is classified by $K^{\mathcal{P}}_{d_2}(\mathcal{P}\mathrm{AII})=\pi_0(R_{d_2-2})$. For the imaginary line gap, it is clear that $K^{\mathcal{P}}_{d_2}(\mathcal{P}\mathrm{AII})=K_{d_1}(\mathrm{AII})$ when $d_1=8-d_2$, corresponding to the relation of Eq. S88.

Point-gap topology. For the class AII, a set of generators

$$\{J\Sigma,\widetilde{U}_{\mathcal{T},+}\mathcal{K},J\widetilde{U}_{\mathcal{T},+}\mathcal{K};\gamma_1,...,\gamma_{d_1}\} \tag{S104}$$

forms a real Clifford algebra Cl_{3,d_1}. The mass term $J\gamma_0$ can induce an extension $Cl_{3,d_1}\to Cl_{4,d_1}$, leading to the classifying space R_{5-d_1}. The point-gap topology of the Hamiltonian in class AI is classified by $K_{d_1}(\mathrm{AI})=\pi_0(R_{5-d_1})$. While, for the class $\mathcal{P}$AII, a set of generators

$$\{J\gamma_1,...,J\gamma_{d_2},J\Sigma,\widetilde{U}_{\mathcal{PT},+}\mathcal{K},J\widetilde{U}_{\mathcal{PT},+}\mathcal{K};\} \tag{S105}$$

forms a real Clifford algebra $Cl_{d_2+3,0}$. The mass term $J\gamma_0$ can induce an extension $Cl_{d_2+3,0}\to Cl_{d_2+4,0}$, leading to the classifying space R_{d_2+5}. The imaginary line-gap topology of the Hamiltonian in class AI is classified by $K^{\mathcal{P}}_{d_2}(\mathcal{P}\mathrm{AI})=\pi_0(R_{d_2+5})$. For the point gap, it is clear that $K^{\mathcal{P}}_{d_2}(\mathcal{P}\mathrm{AII})=K_{d_1}(\mathrm{AII})$ when $d_1=8-d_2$, corresponding to the relation of Eq. S88.

Class BDI and Class $\mathcal{P}$BDI. We consider a d_1-dimensional non-Hermitian Hamiltonian H_{BDI} under the class BDI and a d_2-dimensional non-Hermitian Hamiltonian $H_{\mathcal{P}\mathrm{BDI}}$ under the class $\mathcal{P}$BDI. The symmetry conditions are listed below:

$$\begin{aligned}U_{\mathcal{T},+}H^*_{\mathrm{BDI}}(\boldsymbol{k})U^{-1}_{\mathcal{T},+}&=H_{\mathrm{BDI}}(-\boldsymbol{k}), & U_{\mathcal{T},+}U^*_{\mathcal{T},+}&=1\\ U_{C,+}H^T_{\mathrm{BDI}}(\boldsymbol{k})U^{-1}_{C,+}&=H_{\mathrm{BDI}}(-\boldsymbol{k}), & U_{C,+}U^*_{C,+}&=1\\ U_{\mathcal{PT},+}H^*_{\mathcal{P}\mathrm{BDI}}(\boldsymbol{k})U^{-1}_{\mathcal{PT},+}&=H_{\mathcal{P}\mathrm{BDI}}(\boldsymbol{k}), & U_{\mathcal{PT},+}U^*_{\mathcal{PT},+}&=1\\ U_{\mathcal{P}C,+}H^T_{\mathcal{P}\mathrm{BDI}}(\boldsymbol{k})U^{-1}_{\mathcal{P}C,+}&=H_{\mathcal{P}\mathrm{BDI}}(\boldsymbol{k}), & U_{\mathcal{P}C,+}U^*_{\mathcal{P}C,+}&=1\end{aligned} \tag{S106}$$

Real line-gap topology. For the class BDI, a set of generators

$$\{JU_{\mathcal{T},+}U_{C,+};\gamma_1,...,\gamma_{d_1},U_{\mathcal{T},+}\mathcal{K},JU_{\mathcal{T},+}\mathcal{K}\} \tag{S107}$$

forms a real Clifford algebra Cl_{1,d_1+2}. The mass term $J\gamma_0$ can induce an extension $Cl_{1,d_1+2}\to Cl_{2,d_1+2}$, leading to the classifying space R_{1-d_1}. The real line-gap topology of the Hamiltonian in class AI is classified by $K_{d_1}(\mathrm{AI})=\pi_0(R_{1-d_1})$. While, for the class $\mathcal{P}$BDI, a set of generators

$$\{J\gamma_1,...,J\gamma_{d_1},JU_{\mathcal{T},+}U_{C,+};U_{\mathcal{PT},+}\mathcal{K},JU_{\mathcal{PT},+}\mathcal{K}\} \tag{S108}$$

forms a real Clifford algebra $Cl_{d_2+1,2}$. The mass term $J\gamma_0$ can induce an extension $Cl_{d_2+1,2}\to Cl_{d_2+2,2}$, leading to the classifying space R_{1+d_2}. The real line-gap topology of the Hamiltonian in class $\mathcal{P}$BDI is classified by $K^{\mathcal{P}}_{d_2}(\mathcal{P}\mathrm{BDI})=\pi_0(R_{1+d_2})$. For the real line gap, it is clear that $K^{\mathcal{P}}_{d_2}(\mathcal{P}\mathrm{BDI})=K_{d_1}(\mathrm{BDI})$ when $d_1=8-d_2$, corresponding to the relation of Eq. S88.

Imaginary line-gap topology. There is an emergent symmetry $U_{\mathcal{T},+}U_{C,+}$ and $U_{\mathcal{T},+}U_{C,+}U^*_{\mathcal{T},+}U^*_{C,+}=1$. For the class BDI, a set of generators

$$\{J\gamma_1,...,J\gamma_{d_1};U_{\mathcal{T},+}\mathcal{K},JU_{\mathcal{T},+}\mathcal{K}\}\otimes\{U_{\mathcal{T},+}U_{C,+}\} \tag{S109}$$

forms a real Clifford algebra $Cl_{d_1,2}\times Cl_{d_1,2}$, since $Cl_{0,1}\simeq\mathbb{R}\oplus\mathbb{R}$. The mass term γ_0 can induce an extension $Cl_{d_1,2}\times Cl_{d_1,2}\rightarrow Cl_{d_1,3}\times Cl_{d_1,3}$, leading to the classifying space $R_{2-d_1}\times R_{2-d_1}$. The imaginary line-gap topology of the Hamiltonian in class BDI is classified by $K_{d_1}(\mathrm{BDI})=\pi_0(R_{2-d_1})\oplus\pi_0(R_{2-d_1})$. While, for the class $\mathcal{P}$BDI, a set of generators

$$\{;U_{\mathcal{PT},+}\mathcal{K},JU_{\mathcal{PT},+}\mathcal{K}\gamma_1,...,\gamma_{d_2}\}\otimes\{U_{\mathcal{PT},+}U_{\mathcal{PC},+}\} \quad (\mathrm{S110})$$

forms a real Clifford algebra $Cl_{0,d_2+2}\times Cl_{0,d_2+2}$. The mass term γ_0 can induce an extension $Cl_{0,d_2+2}\times Cl_{0,d_2+2}\rightarrow Cl_{0,d_2+3}\times Cl_{0,d_2+3}$, leading to the classifying space $R_{d_2+2}\times R_{d_2+2}$. The imaginary line-gap topology of the Hamiltonian in class $\mathcal{P}$BDI is classified by $K^{\mathcal{P}}_{d_2}(\mathcal{P}\mathrm{BDI})=\pi_0(R_{d_2+2})\oplus\pi_0(R_{d_2+2})$. For the imaginary line gap, it is clear that $K^{\mathcal{P}}_{d_2}(\mathcal{P}\mathrm{BDI})=K_{d_1}(\mathrm{BDI})$ when $d_1=8-d_2$, corresponding to the relation of Eq. S88.

Point-gap topology. For the class BDI, a set of generators

$$\{J\Sigma,J\widetilde{U}_{\mathcal{T},+}\widetilde{U}_{C,+};\gamma_1,...,\gamma_{d_1},\widetilde{U}_{\mathcal{T},+}\mathcal{K},J\widetilde{U}_{\mathcal{T},+}\mathcal{K}\} \quad (\mathrm{S111})$$

forms a real Clifford algebra Cl_{2,d_1+2}. The mass term $J\gamma_0$ can induce an extension $Cl_{2,d_1+2}\rightarrow Cl_{3,d_1+2}$, leading to the classifying space R_{2-d_1}. The point-gap topology of the Hamiltonian in class BDI is classified by $K_{d_1}(\mathrm{BDI})=\pi_0(R_{2-d_1})$. While, for the class $\mathcal{P}$BDI, a set of generators

$$\{J\gamma_1,...,J\gamma_{d_2},J\Sigma,\widetilde{U}_{\mathcal{T},+}\widetilde{U}_{C,+};\widetilde{U}_{\mathcal{PT},+}\mathcal{K},J\widetilde{U}_{\mathcal{PT},+}\mathcal{K}\} \quad (\mathrm{S112})$$

forms a real Clifford algebra $Cl_{d_2+2,2}$. The mass term $J\gamma_0$ can induce an extension $Cl_{d_2+2,2}\rightarrow Cl_{d_2+3,2}$, leading to the classifying space R_{d_2+2}. The imaginary line-gap topology of the Hamiltonian in class BDI is classified by $K^{\mathcal{P}}_{d_2}(\mathcal{P}\mathrm{BDI})=\pi_0(R_{d_2+2})$. For the point gap, it is clear that $K^{\mathcal{P}}_{d_2}(\mathcal{P}\mathrm{BDI})=K_{d_1}(\mathrm{BDI})$ when $d_1=8-d_2$, corresponding to the relation of Eq. S88.

Class DIII and Class $\mathcal{P}$DIII. For class DIII and class $\mathcal{P}$DIII, there is the complexification for the Clifford algebra for the imaginary line-gap topology. Here, we show that the relation of Eq. S88 still holds for the case with the complexification. We consider a d_1-dimensional non-Hermitian Hamiltonian H_{DIII} under the class DIII and a d_2-dimensional non-Hermitian Hamiltonian $H_{\mathcal{P}\mathrm{DIII}}$ under the class $\mathcal{P}$DIII. The symmetry conditions are listed below:

$$\begin{aligned}
U_{\mathcal{T},+}H^{*}_{\mathrm{DIII}}(\boldsymbol{k})U^{-1}_{\mathcal{T},+}&=H_{\mathrm{DIII}}(-\boldsymbol{k}), & U_{\mathcal{T},+}U^{*}_{\mathcal{T},+}&=-1\\
U_{C,+}H^{T}_{\mathrm{DIII}}(\boldsymbol{k})U^{-1}_{C,+}&=H_{\mathrm{DIII}}(-\boldsymbol{k}), & U_{C,+}U^{*}_{C,+}&=1\\
U_{\mathcal{PT},+}H^{*}_{\mathcal{P}\mathrm{DIII}}(\boldsymbol{k})U^{-1}_{\mathcal{PT},+}&=H_{\mathcal{P}\mathrm{DIII}}(\boldsymbol{k}), & U_{\mathcal{PT},+}U^{*}_{\mathcal{PT},+}&=-1\\
U_{\mathcal{PC},+}H^{T}_{\mathcal{P}\mathrm{DIII}}(\boldsymbol{k})U^{-1}_{\mathcal{PC},+}&=H_{\mathcal{P}\mathrm{DIII}}(\boldsymbol{k}), & U_{\mathcal{PC},+}U^{*}_{\mathcal{PC},+}&=1
\end{aligned} \quad (\mathrm{S113})$$

Similar to the case of class BDI, there is an emergent symmetry $U_{\mathcal{T},+}U_{C,+}$ and $U_{\mathcal{T},+}U_{C,+}U^{*}_{\mathcal{T},+}U^{*}_{C,+}=-1$ for class DIII. For the class DIII, a set of generators

$$\{U_{\mathcal{T},+}\mathcal{K},JU_{\mathcal{T},+}\mathcal{K},J\gamma_1,...,J\gamma_{d_1};\}\otimes\{U_{\mathcal{T},+}U_{C,+}\} \quad (\mathrm{S114})$$

forms a complex Clifford algebra Cl_{d_1+2}, since $Cl_{1,0}\simeq\mathbb{C}$. The mass term γ_0 can induce an extension $Cl_{d_1+2}\rightarrow Cl_{d_1+3}$, leading to the classifying space $C_{d_1+2}\simeq C_{d_1}$. The imaginary line-gap topology of the Hamiltonian in class DIII is classified by $K_{d_1}(\mathrm{DIII})=\pi_0(C_{d_1})$.

While for the class $\mathcal{P}$BDI, a set of generators

$$\{;\gamma_1,...,\gamma_{d_2},U_{\mathcal{PT},+}\mathcal{K},JU_{\mathcal{PT},+}\mathcal{K}\}\otimes\{U_{\mathcal{PT},+}U_{\mathcal{PC},+}\} \quad (\mathrm{S115})$$

forms a complex Clifford algebra Cl_{d_2+2}. The mass term γ_0 can induce an extension $Cl_{d_2+2}\rightarrow Cl_{d_2+3}$, leading to the classifying space $C_{d_2+2}\simeq C_{d_2}$. The imaginary line-gap topology of the Hamiltonian in class $\mathcal{P}$DIII is classified by $K^{\mathcal{P}}_{d_2}(\mathcal{P}\mathrm{DIII})=\pi_0(C_{d_2})$. Since $C_{q+2}\simeq C_q$, for the imaginary line gap, it is clear that $K^{\mathcal{P}}_{d_2}(\mathcal{P}\mathrm{DIII})=K_{d_1}(\mathrm{DIII})$ when $d_1=8-d_2$, corresponding to the relation of Eq. S88.

From the above cases, we can see that the relation of Eq. S88 obtained by our algorithm is consistent with the results from the Clifford algebra. Note that although the Clifford algebra can obtain the topological classification for non-Hermitian systems, it cannot be directly applied to distinguish the topological phase difference between two concrete Hamiltonians.

[1] Y. Long and B. Zhang, Unsupervised data-driven classification of topological gapped systems with symmetries, Phys. Rev. Lett. **130**, 036601 (2023).

[2] J. F. Rodriguez-Nieva and M. S. Scheurer, Identifying topological order through unsupervised machine learning, Nat. Phys. **15**, 790 (2019).

[3] Z. Gong, Y. Ashida, K. Kawabata, K. Takasan, S. Higashikawa, and M. Ueda, Topological phases of non-hermitian systems, Phys. Rev. X **8**, 031079 (2018).

[4] K. Kawabata, K. Shiozaki, M. Ueda, and M. Sato, Symmetry and topology in non-hermitian physics, Phys. Rev. X **9**, 041015 (2019).

[5] E. J. Bergholtz, J. C. Budich, and F. K. Kunst, Exceptional topology of non-hermitian systems, Rev. Mod. Phys. **93**, 015005 (2021).

[6] C.-K. Chiu, J. C. Teo, A. P. Schnyder, and S. Ryu, Classification of topological quantum matter with symmetries, Rev. Mod. Phys. **88**, 035005 (2016).

[7] N. Hatano and D. R. Nelson, Localization transitions in non-hermitian quantum mechanics, Phys. Rev. Lett. **77**, 570 (1996).

[8] K. Zhang, Z. Yang, and C. Fang, Correspondence between winding numbers and skin modes in non-hermitian systems, Phys. Rev. Lett. **125**, 126402 (2020).

[9] L. Li, C. H. Lee, S. Mu, and J. Gong, Critical non-hermitian skin effect, Nat. Commun. **11**, 5491 (2020).

[10] C.-X. Guo, C.-H. Liu, X.-M. Zhao, Y. Liu, and S. Chen, Exact solution of non-hermitian systems with generalized boundary conditions: Size-dependent boundary effect and fragility of the skin effect, Phys. Rev. Lett. **127**, 116801 (2021).

[11] L. Zhang, Y. Yang, Y. Ge, Y.-J. Guan, Q. Chen, Q. Yan,

F. Chen, R. Xi, Y. Li, D. Jia, S.-Q. Yuan, H.-X. Sun, H. Chen, and B. Zhang, Acoustic non-hermitian skin effect from twisted winding topology, Nat. Commun. **12**, 6297 (2021).

[12] F. Song, S. Yao, and Z. Wang, Non-hermitian topological invariants in real space, Phys. Rev. Lett. **123**, 246801 (2019).

[13] Y. Yi and Z. Yang, Non-hermitian skin modes induced by on-site dissipations and chiral tunneling effect, Phys. Rev. Lett. **125**, 186802 (2020).

[14] N. Okuma, K. Kawabata, K. Shiozaki, and M. Sato, Topological origin of non-hermitian skin effects, Phys. Rev. Lett. **124**, 086801 (2020).

[15] S. Yao and Z. Wang, Edge states and topological invariants of non-hermitian systems, Phys. Rev. Lett. **121**, 086803 (2018).

[16] T. E. Lee, Anomalous edge state in a non-hermitian lattice, Phys. Rev. Lett. **116**, 133903 (2016).

[17] K. Takata and M. Notomi, Photonic topological insulating phase induced solely by gain and loss, Phys. Rev. Lett. **121**, 213902 (2018).

[18] H. Xue, Q. Wang, B. Zhang, and Y. Chong, Non-hermitian dirac cones, Phys. Rev. Lett. **124**, 236403 (2020).

[19] H. Gao, H. Xue, Q. Wang, Z. Gu, T. Liu, J. Zhu, and B. Zhang, Observation of topological edge states induced solely by non-hermiticity in an acoustic crystal, Phys. Rev. B **101**, 180303 (2020).

[20] Y. Long, H. Xue, and B. Zhang, Non-hermitian topological systems with eigenvalues that are always real, Phys. Rev. B **105**, l100102 (2022).

[21] S. Yao, F. Song, and Z. Wang, Non-hermitian chern bands, Phys. Rev. Lett. **121**, 136802 (2018).

[22] X.-L. Qi, Y.-S. Wu, and S.-C. Zhang, Topological quantization of the spin hall effect in two-dimensional paramagnetic semiconductors, Phys. Rev. B **74**, 085308 (2006).

[23] Y. X. Zhao, Y.-X. Huang, and S. A. Yang, $\mathbb{Z}_2$ -projective translational symmetry protected topological phases, Phys. Rev. B **102**, 161117 (2020).

[24] H. Xue, Z. Wang, Y.-X. Huang, Z. Cheng, L. Yu, Y. Foo, Y. Zhao, S. A. Yang, and B. Zhang, Projectively enriched symmetry and topology in acoustic crystals, Phys. Rev. Lett. **128**, 116802 (2022).

[25] T. Li, J. Du, Q. Zhang, Y. Li, X. Fan, F. Zhang, and C. Qiu, Acoustic möbius insulators from projective symmetry, Phys. Rev. Lett. **128**, 116803 (2022).

[26] X.-W. Luo and C. Zhang, Higher-order topological corner states induced by gain and loss, Phys. Rev. Lett. **123**, 073601 (2019).

[27] H. Xue, Y. Ge, H.-X. Sun, Q. Wang, D. Jia, Y.-J. Guan, S.-Q. Yuan, Y. Chong, and B. Zhang, Observation of an acoustic octupole topological insulator, Nat. Commun. **11**, 10.1038/s41467-020-16350-1 (2020).

[28] H. Gao, H. Xue, Z. Gu, T. Liu, J. Zhu, and B. Zhang, Non-hermitian route to higher-order topology in an acoustic crystal, Nat. Commun. **12**, 1888 (2021).

[29] W. A. Benalcazar, B. A. Bernevig, and T. L. Hughes, Quantized electric multipole insulators, Science **357**, 61 (2017).

[30] K. Kawabata, S. Higashikawa, Z. Gong, Y. Ashida, and M. Ueda, Topological unification of time-reversal and particle-hole symmetries in non-hermitian physics, Nat. Commun. **10**, 297 (2019).

[31] H. Zhou and J. Y. Lee, Periodic table for topological bands with non-hermitian symmetries, Phys. Rev. B **99**, 235112 (2019).

[32] D. Bernard and A. LeClair, A classification of non-hermitian random matrices, *Statistical Field Theories*, Statistical Field Theories , 207 (2002).

[33] K. Shiozaki and M. Sato, Topology of crystalline insulators and superconductors, Phys. Rev. B **90**, 165114 (2014).

[34] J. C. Y. Teo and C. L. Kane, Topological defects and gapless modes in insulators and superconductors, Phys. Rev. B **82**, 115120 (2010).

[35] C. C. Wojcik, X.-Q. Sun, T. Bzdušek, and S. Fan, Homotopy characterization of non-hermitian hamiltonians, Phys. Rev. B **101**, 205417 (2020).

[36] C.-K. Chiu, H. Yao, and S. Ryu, Classification of topological insulators and superconductors in the presence of reflection symmetry, Phys. Rev. B **88**, 075142 (2013).

[37] Y.-M. Lu and D.-H. Lee, Inversion symmetry protected topological insulators and superconductors, arXiv , 1403.5558 (2014).

[38] S. K. Ozdemir, S. Rotter, F. Nori, and L. Yang, Parity–time symmetry and exceptional points in photonics, Nat. Mater. **18**, 783 (2019).

[39] L. Feng, R. El-Ganainy, and L. Ge, Non-hermitian photonics based on parity–time symmetry, Nat. Photonics **11**, 752 (2017).

[40] K. Kawabata, T. Bessho, and M. Sato, Classification of exceptional points and non-hermitian topological semimetals, Phys. Rev. Lett. **123**, 066405 (2019).

[41] M. Karoubi, *K-Theory: An Introduction* (Springer Berlin Heidelberg, 1978).

[42] A. Kitaev, V. Lebedev, and M. Feigel'man, Periodic table for topological insulators and superconductors, in *AIP Conference Proceedings* (AIP, 2009).

[43] T. Morimoto and A. Furusaki, Topological classification with additional symmetries from clifford algebras, Phys. Rev. B **88**, 125129 (2013).

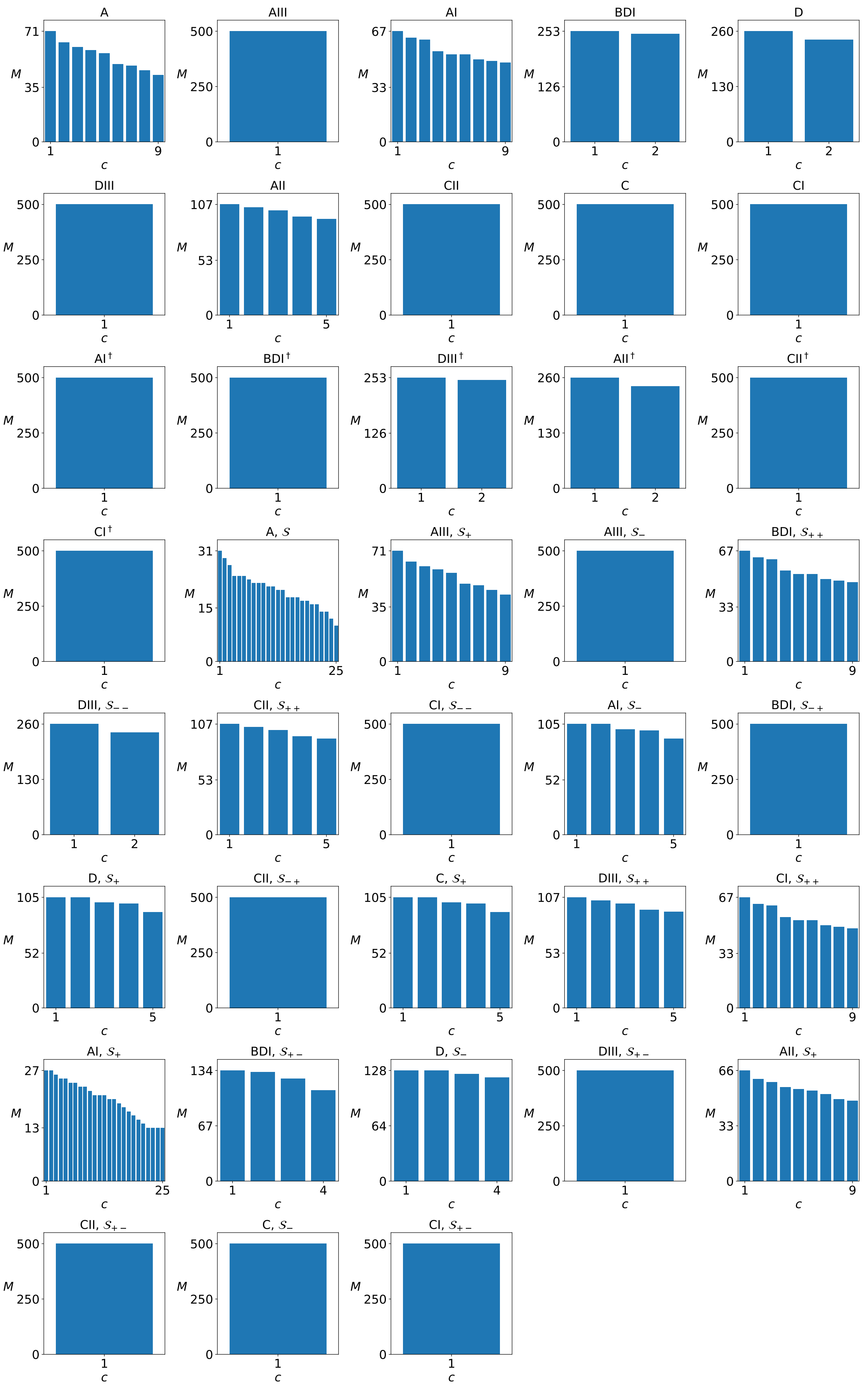

FIG. S21. Topological classifications for non-Hermitian topological systems in $d = 1$ dimension with a point gap.

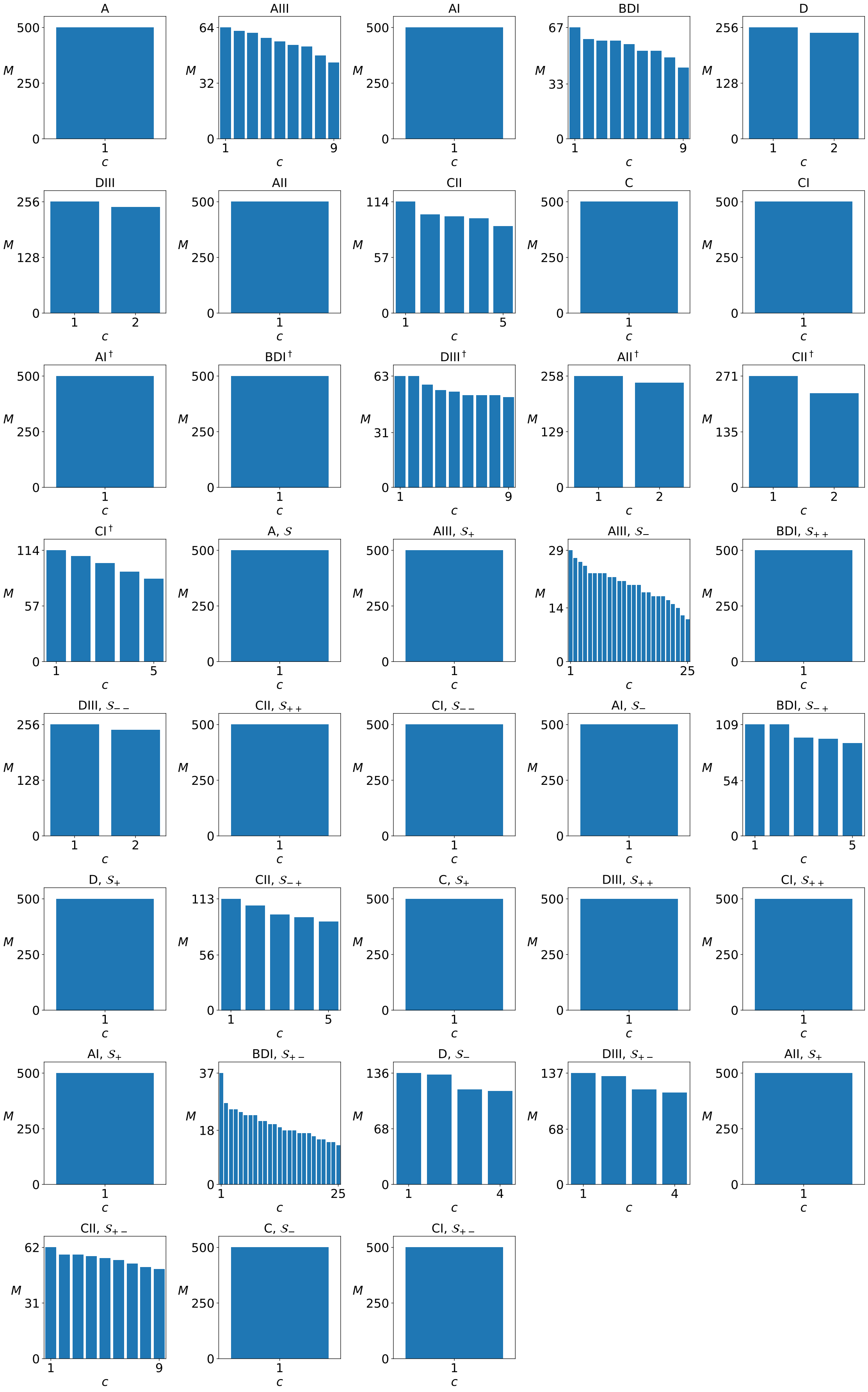

FIG. S22. Topological classifications for non-Hermitian topological systems in $d = 2$ dimension with a point gap.

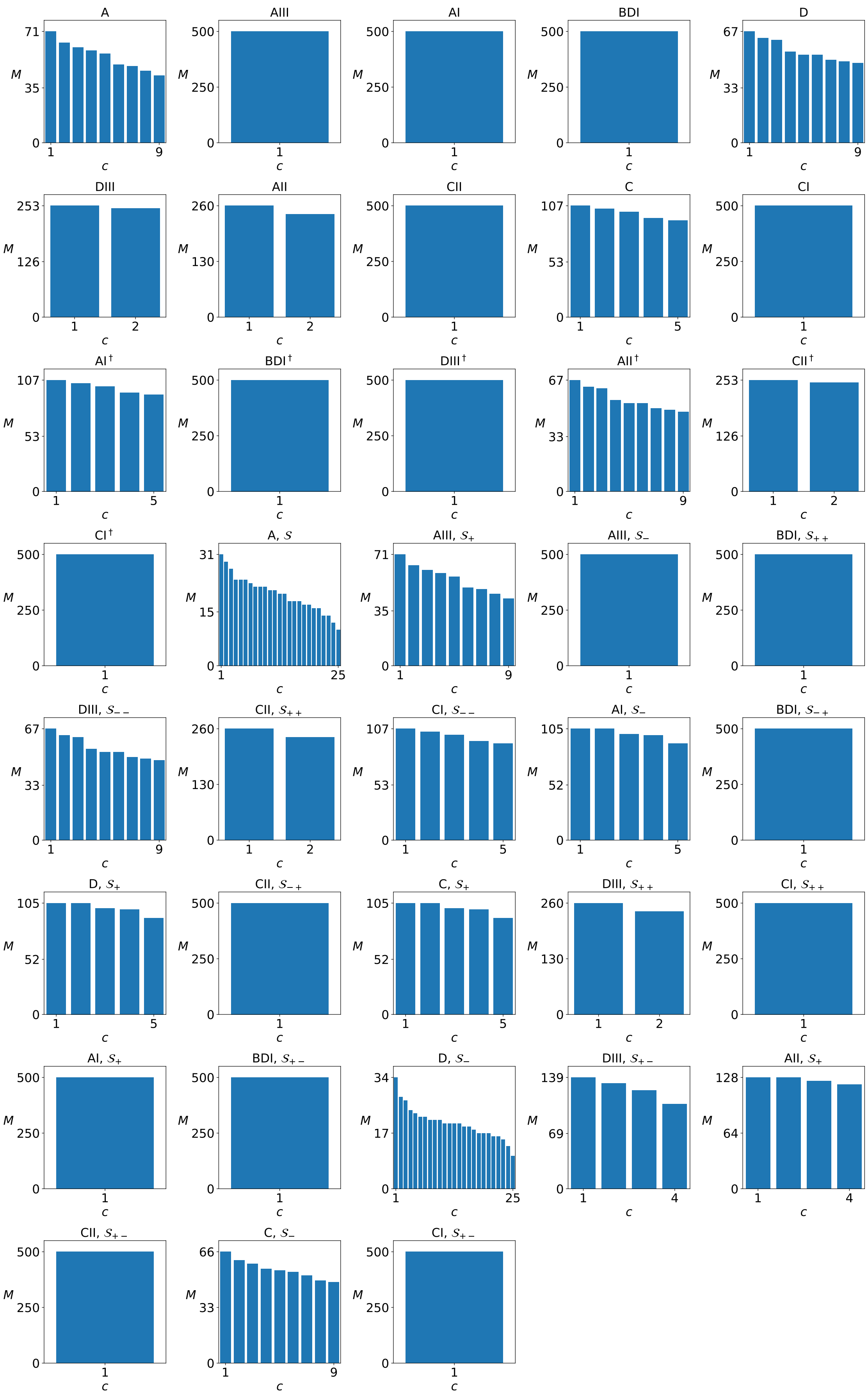

FIG. S23. Topological classifications for non-Hermitian topological systems in $d = 3$ dimension with a point gap.

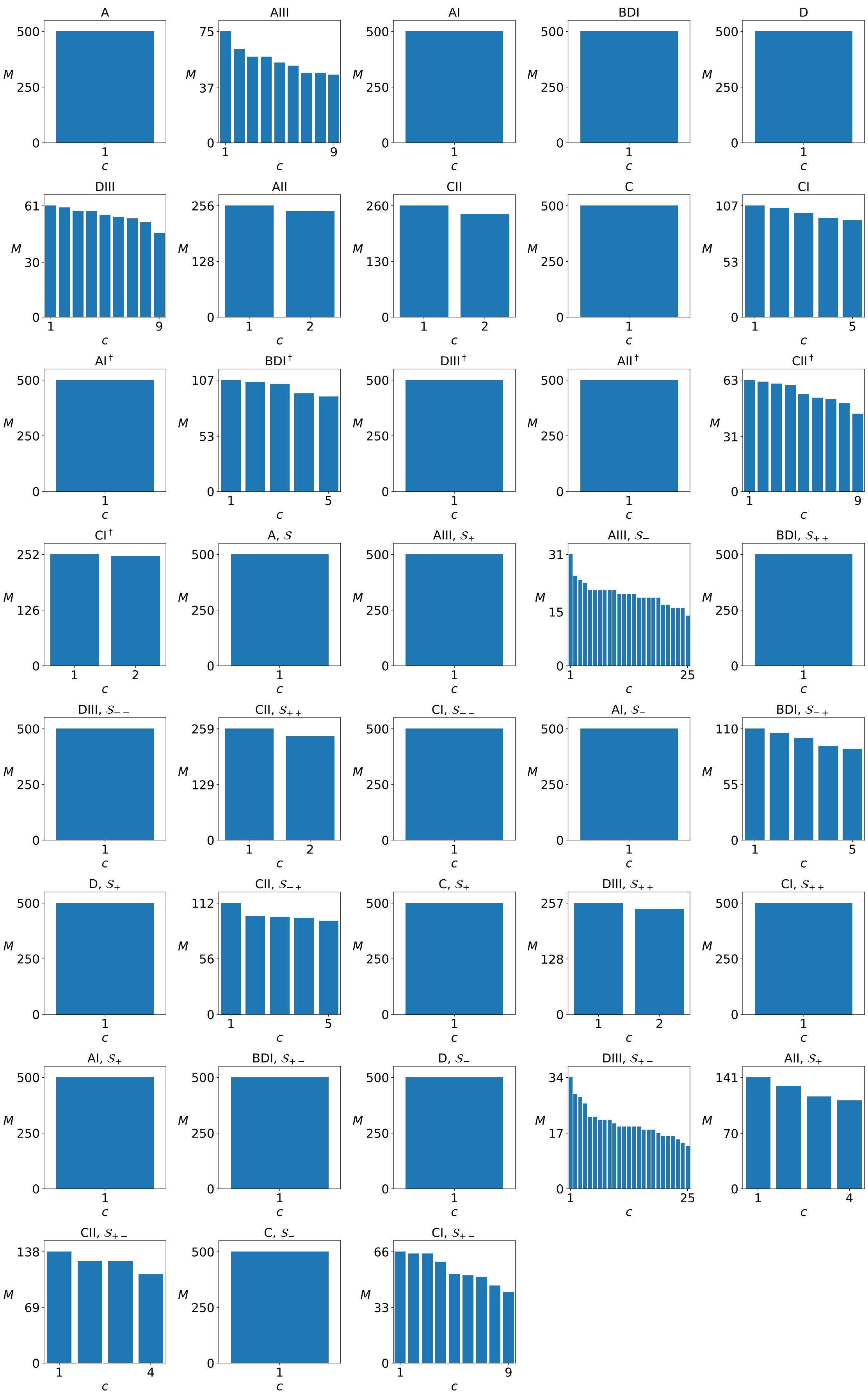

FIG. S24. Topological classifications for non-Hermitian topological systems in $d = 4$ dimension with a point gap.

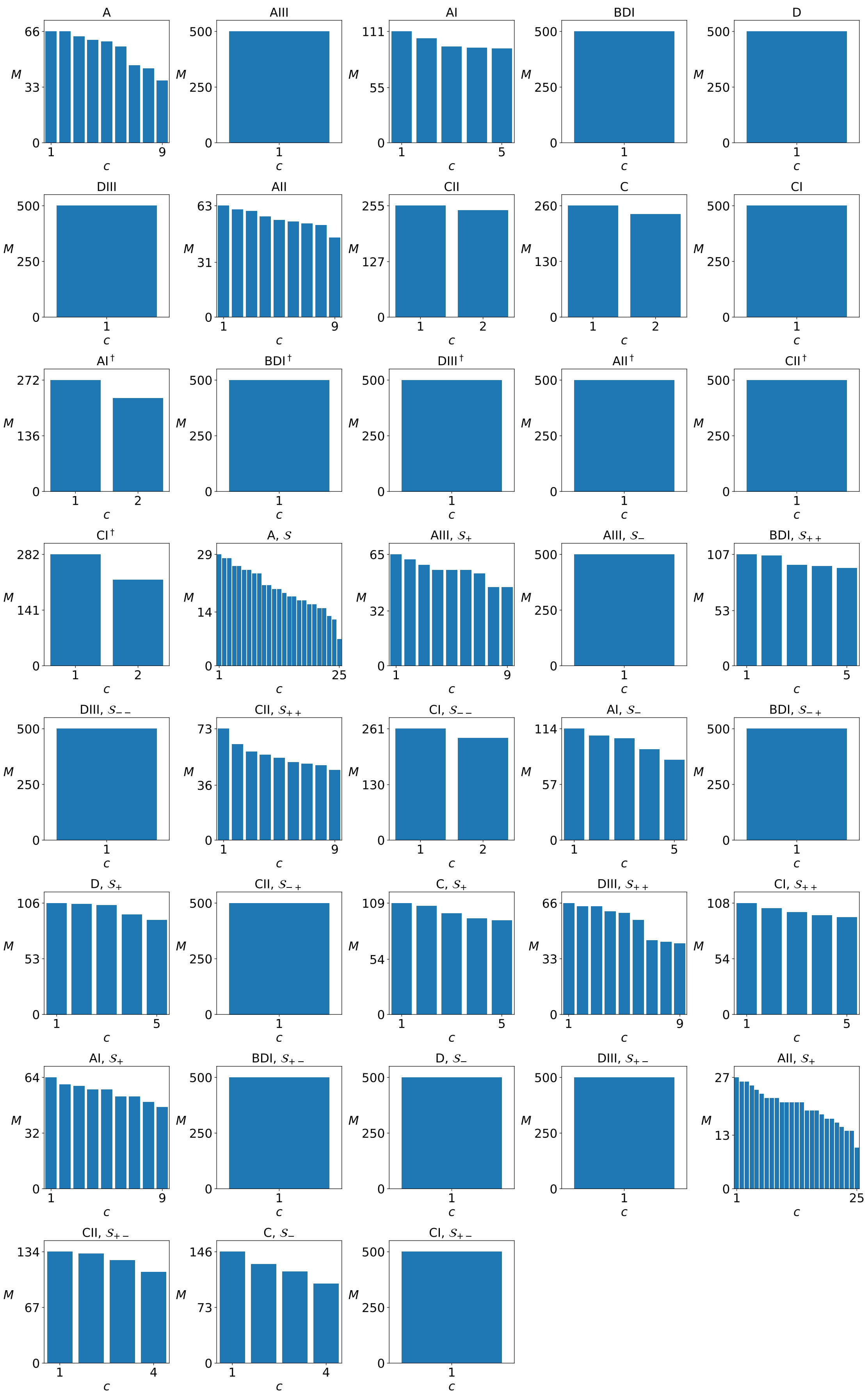

FIG. S25. Topological classifications for non-Hermitian topological systems in $d = 5$ dimension with a point gap.

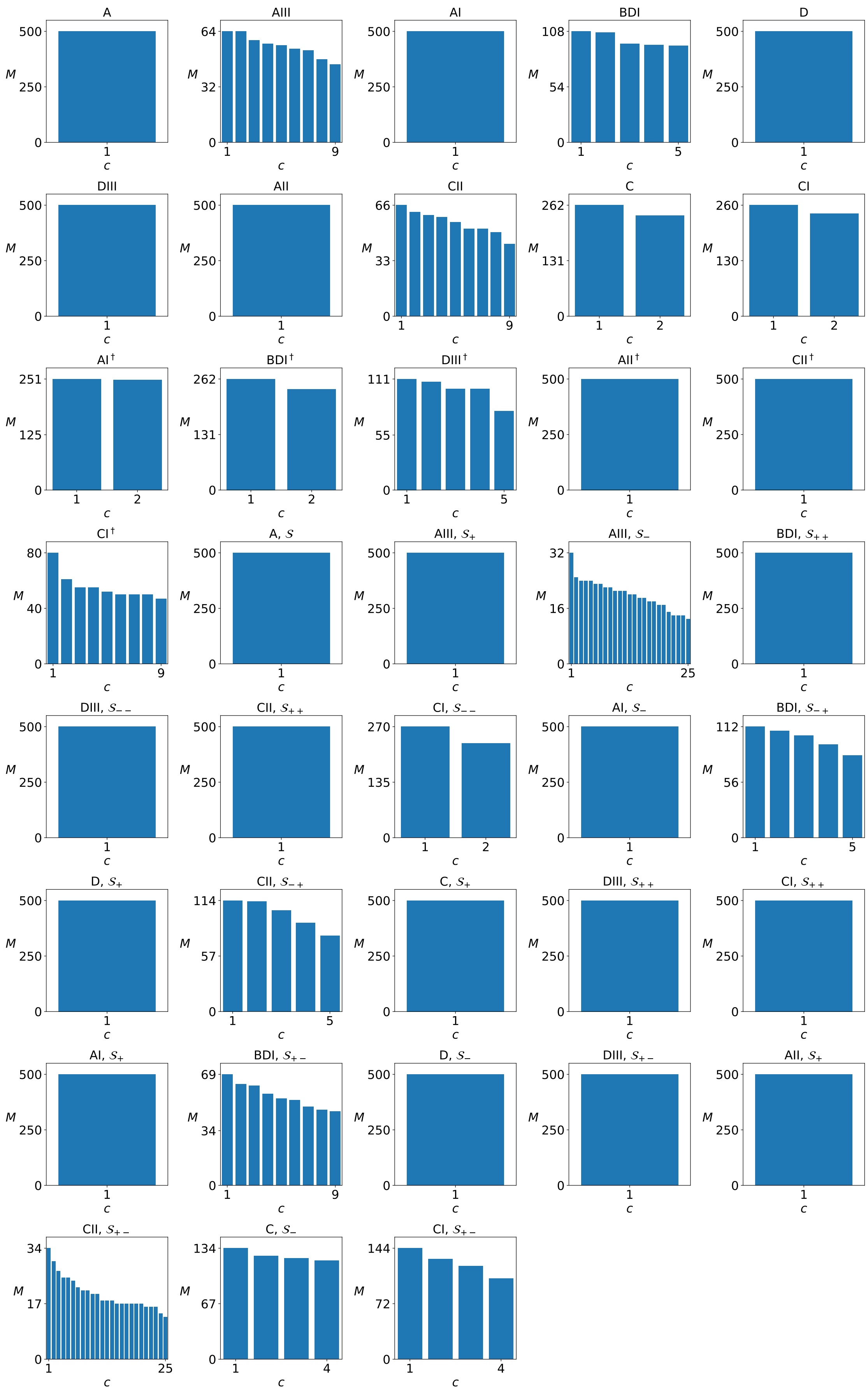

FIG. S26. Topological classifications for non-Hermitian topological systems in $d = 6$ dimension with a point gap.

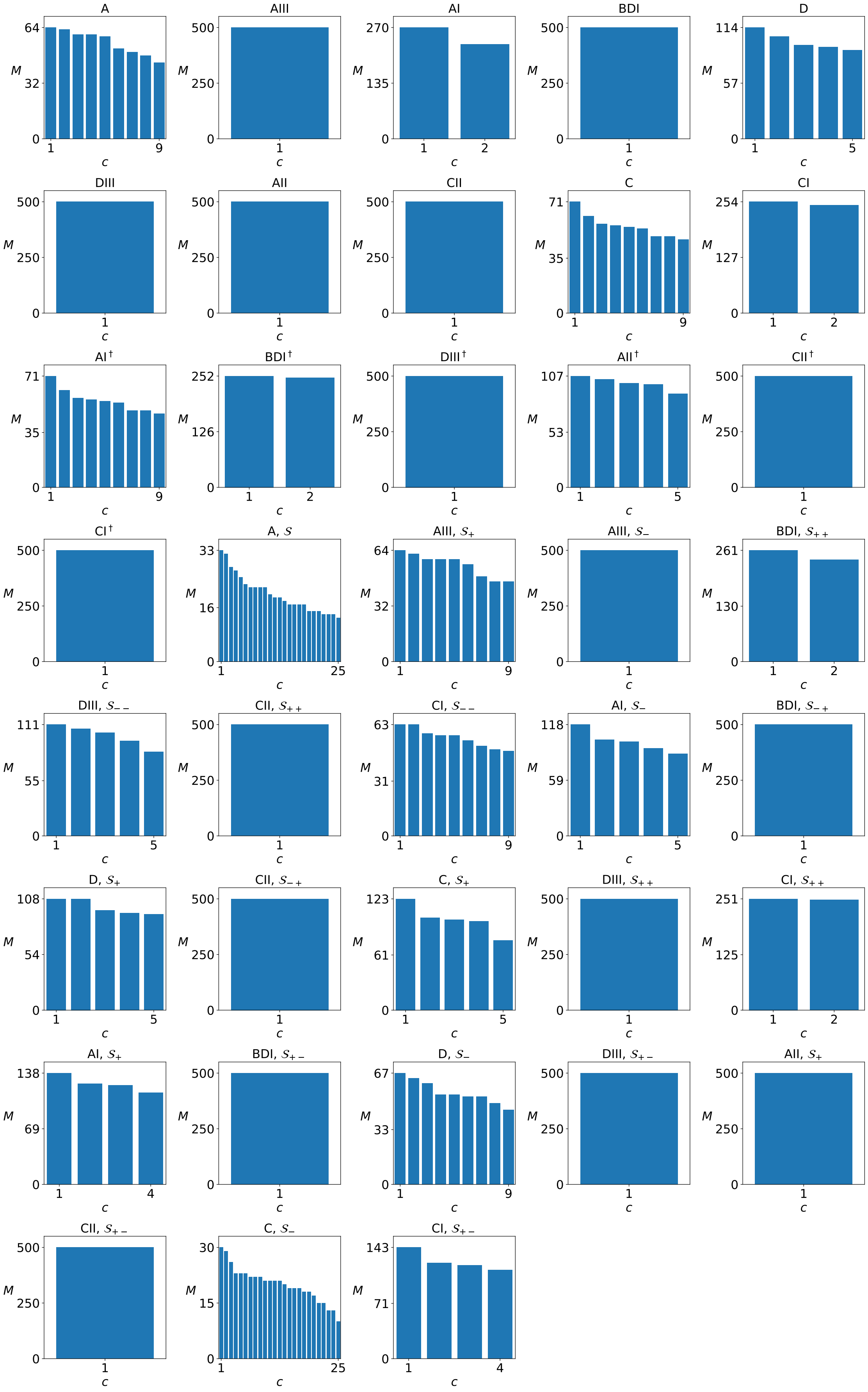

FIG. S27. Topological classifications for non-Hermitian topological systems in $d = 7$ dimension with a point gap.

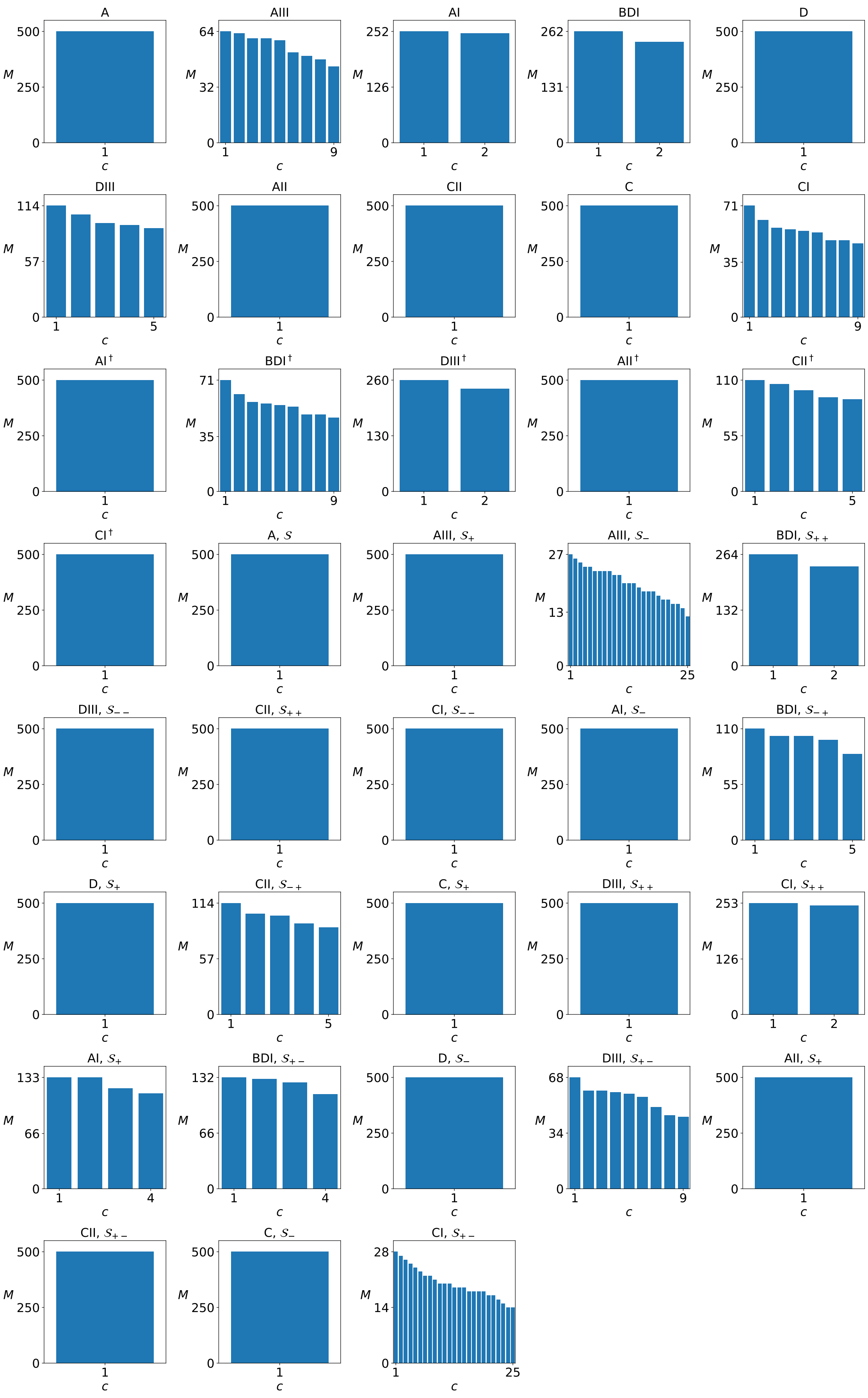

FIG. S28. Topological classifications for non-Hermitian topological systems in $d = 8$ dimension with a point gap.

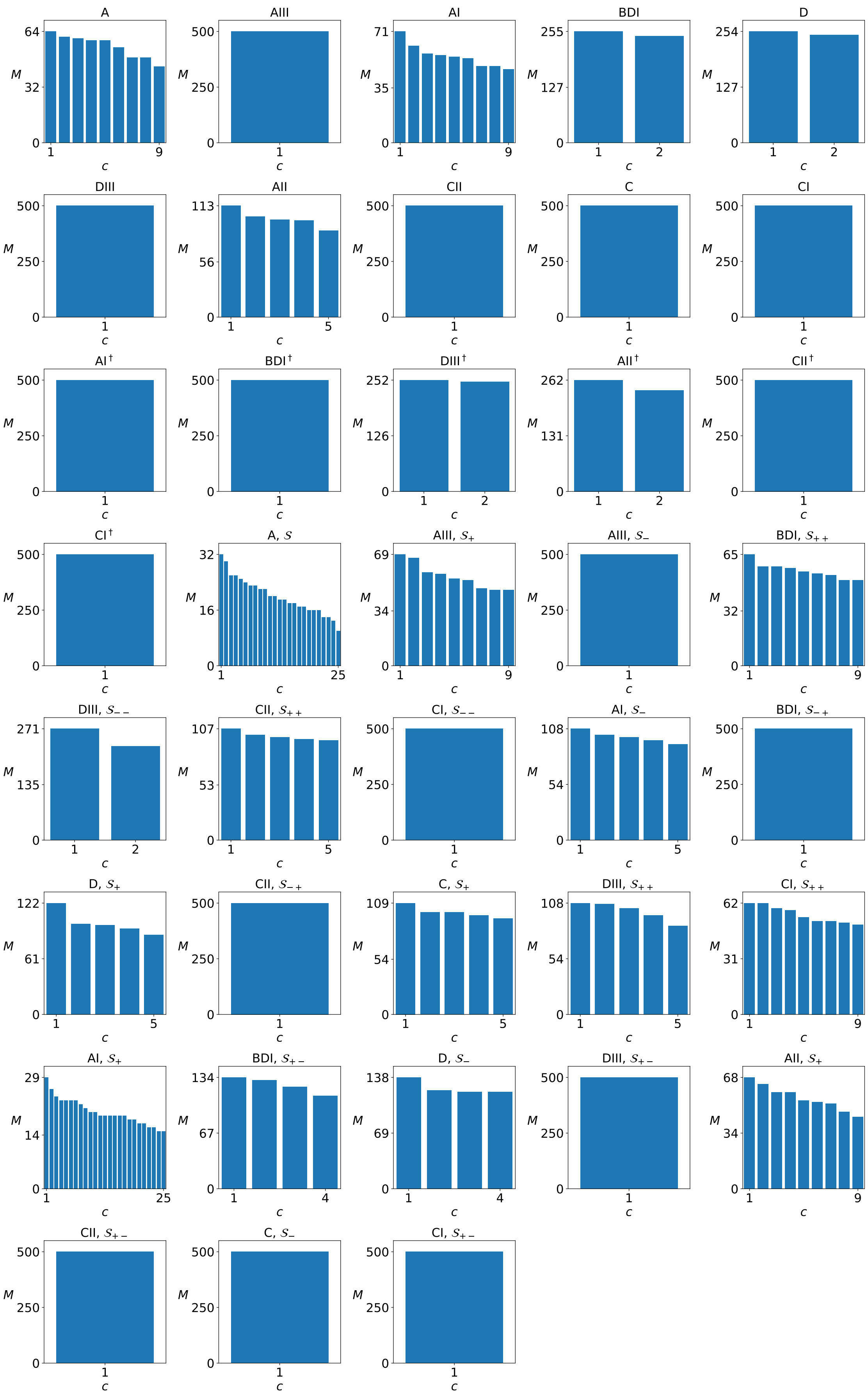

FIG. S29. Topological classifications for non-Hermitian topological systems in $d = 9$ dimension with a point gap.

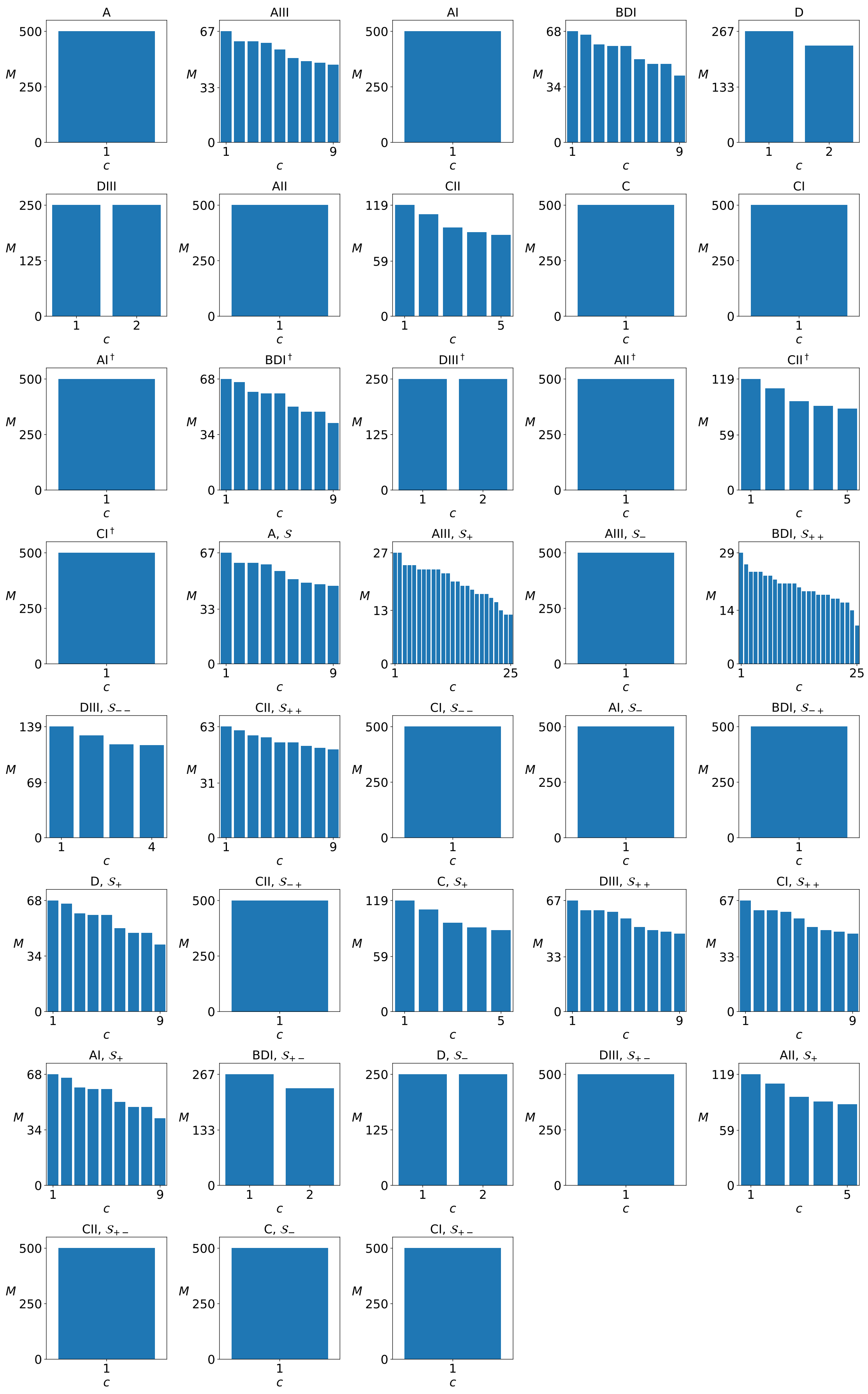

FIG. S30. Topological classifications for non-Hermitian topological systems in $d = 1$ dimension with a real line gap.

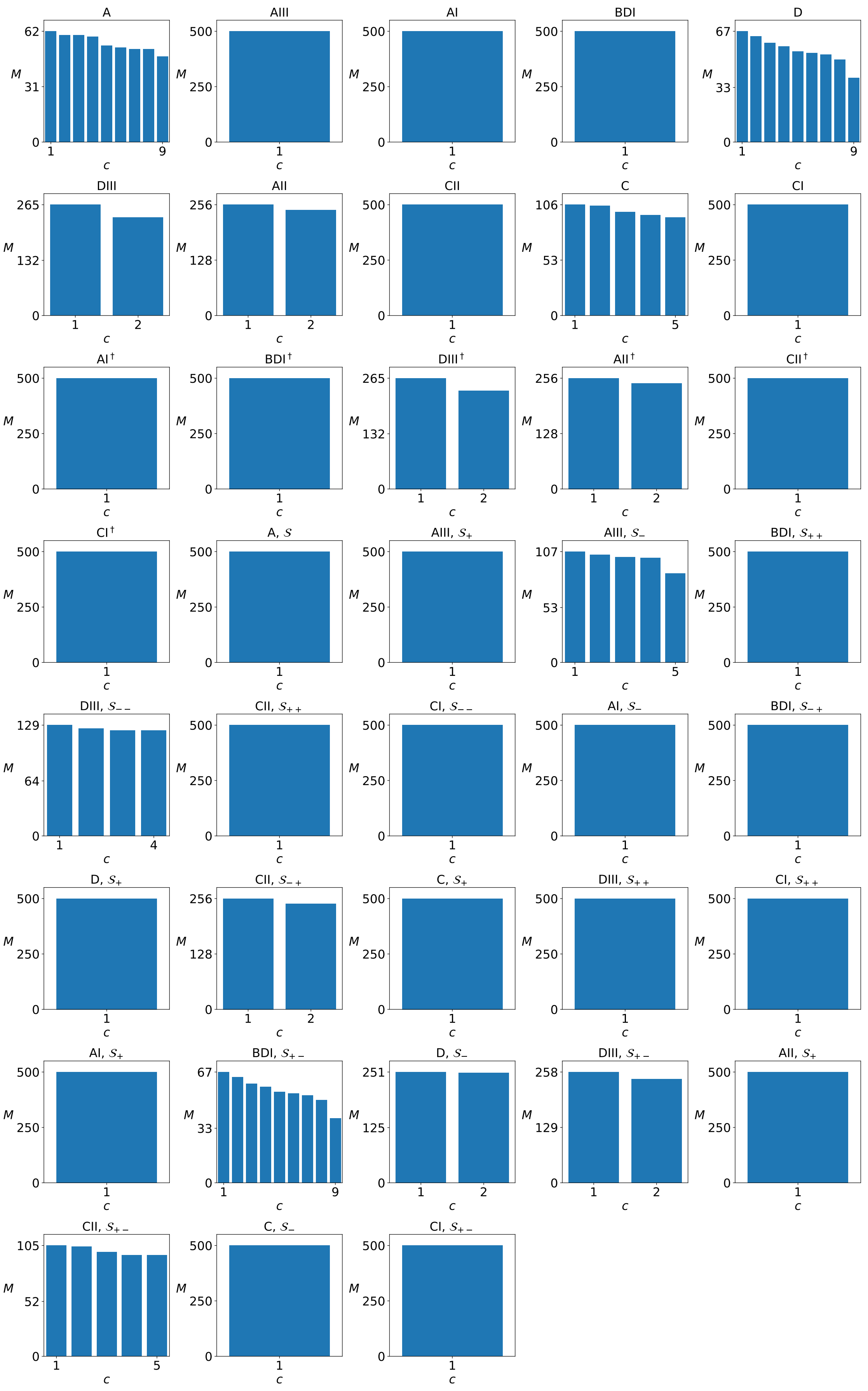

FIG. S31. Topological classifications for non-Hermitian topological systems in $d = 2$ dimension with a real line gap.

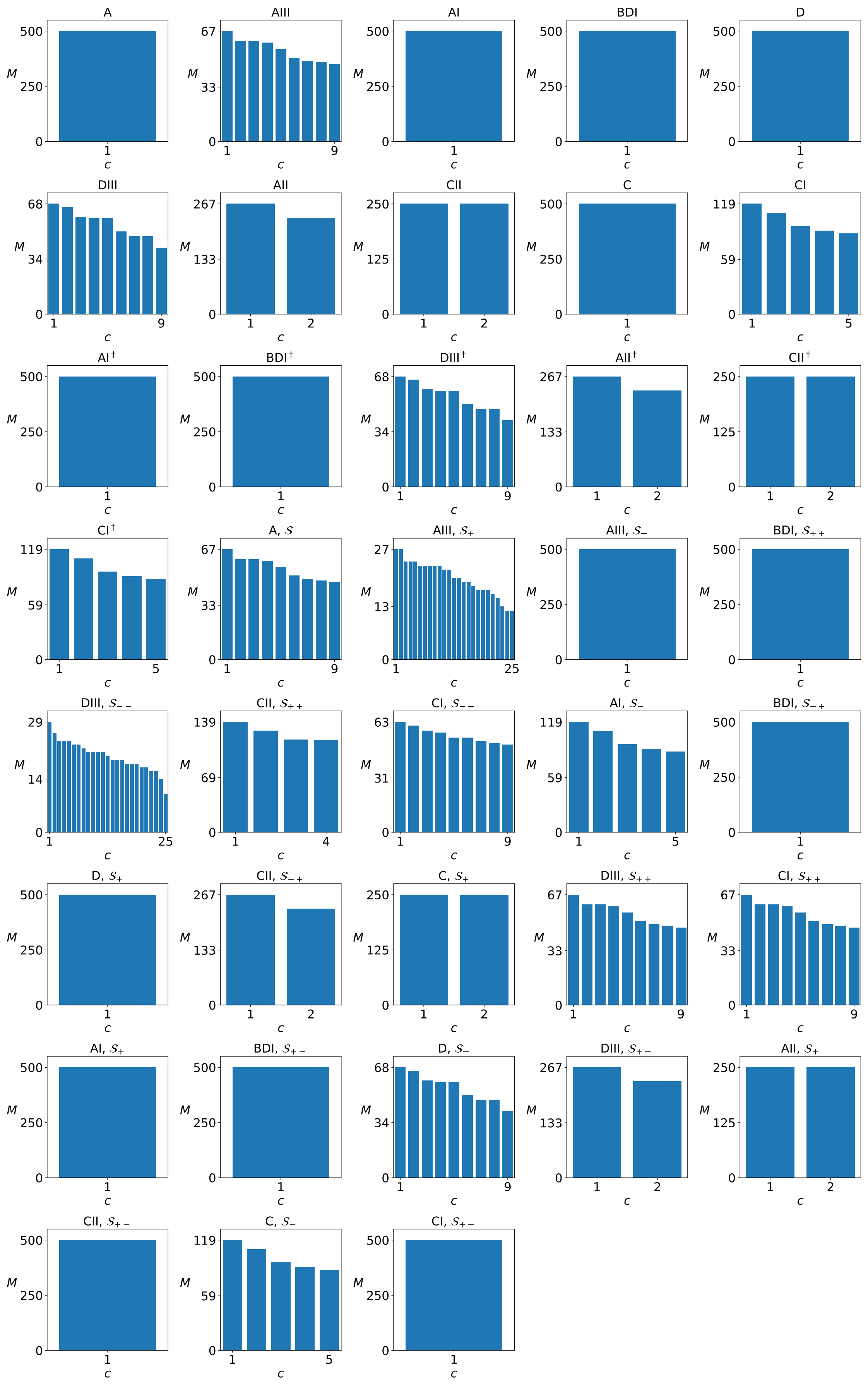

FIG. S32. Topological classifications for non-Hermitian topological systems in $d = 3$ dimension with a real line gap.

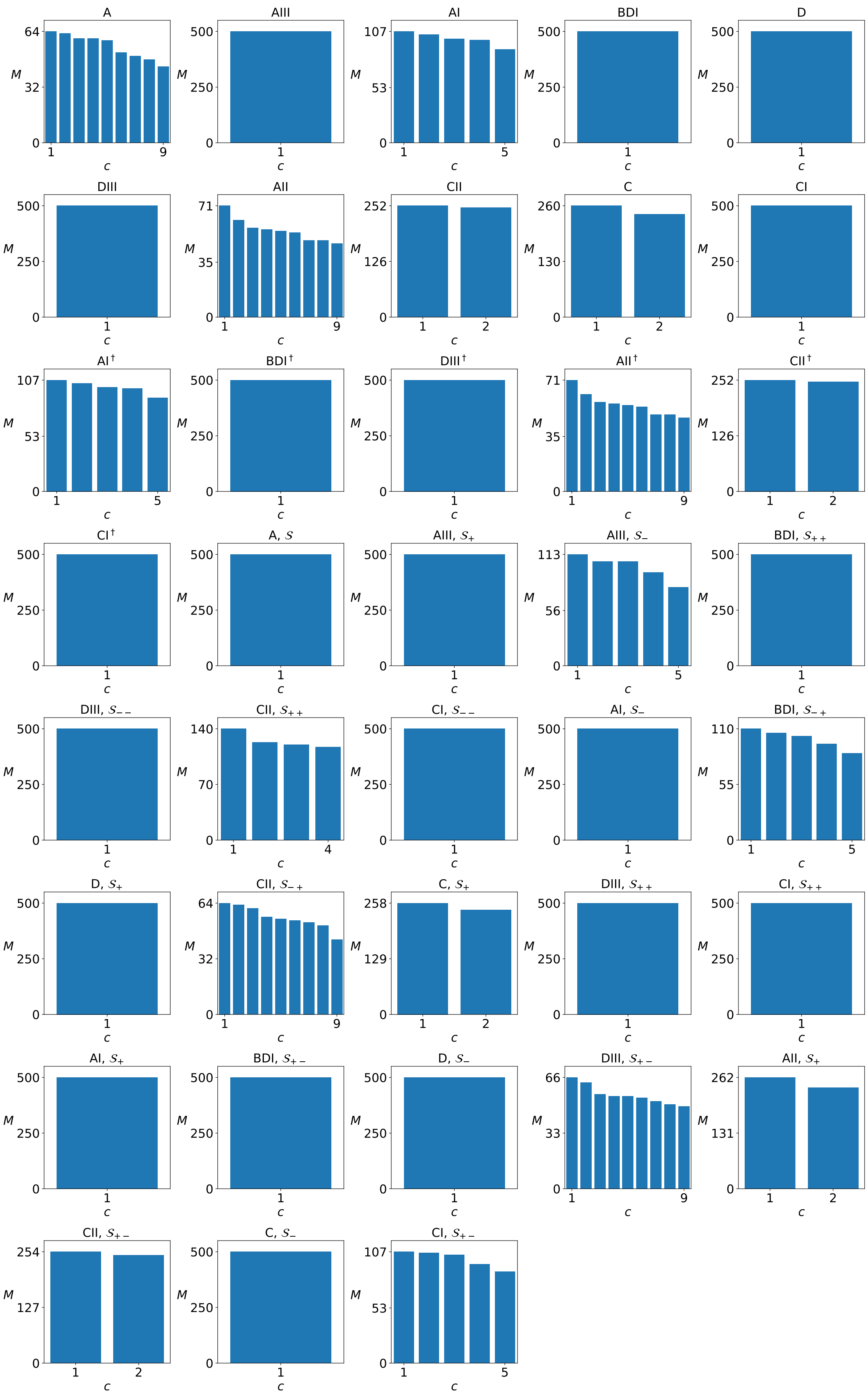

FIG. S33. Topological classifications for non-Hermitian topological systems in $d = 4$ dimension with a real line gap.

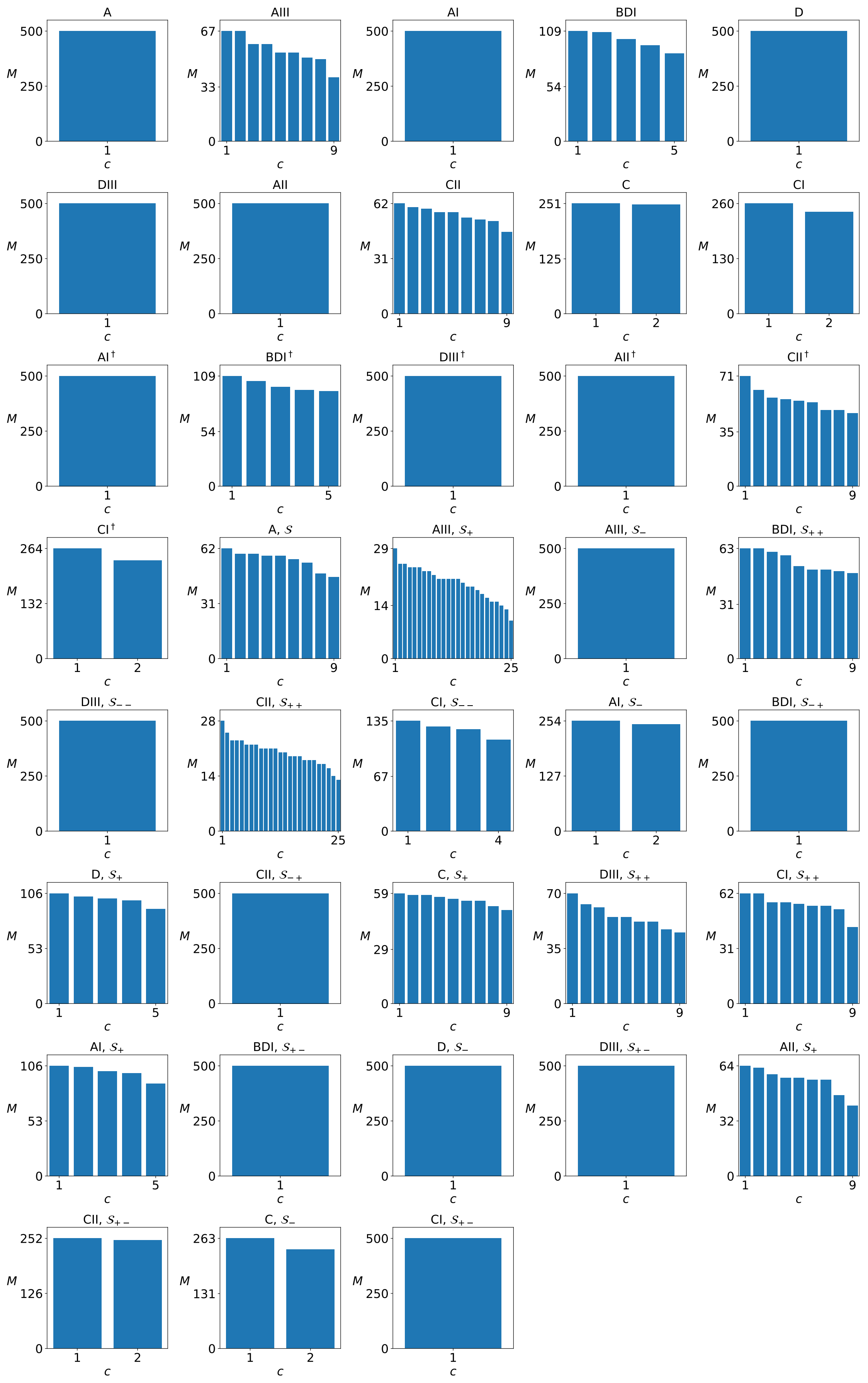

FIG. S34. Topological classifications for non-Hermitian topological systems in $d = 5$ dimension with a real line gap.

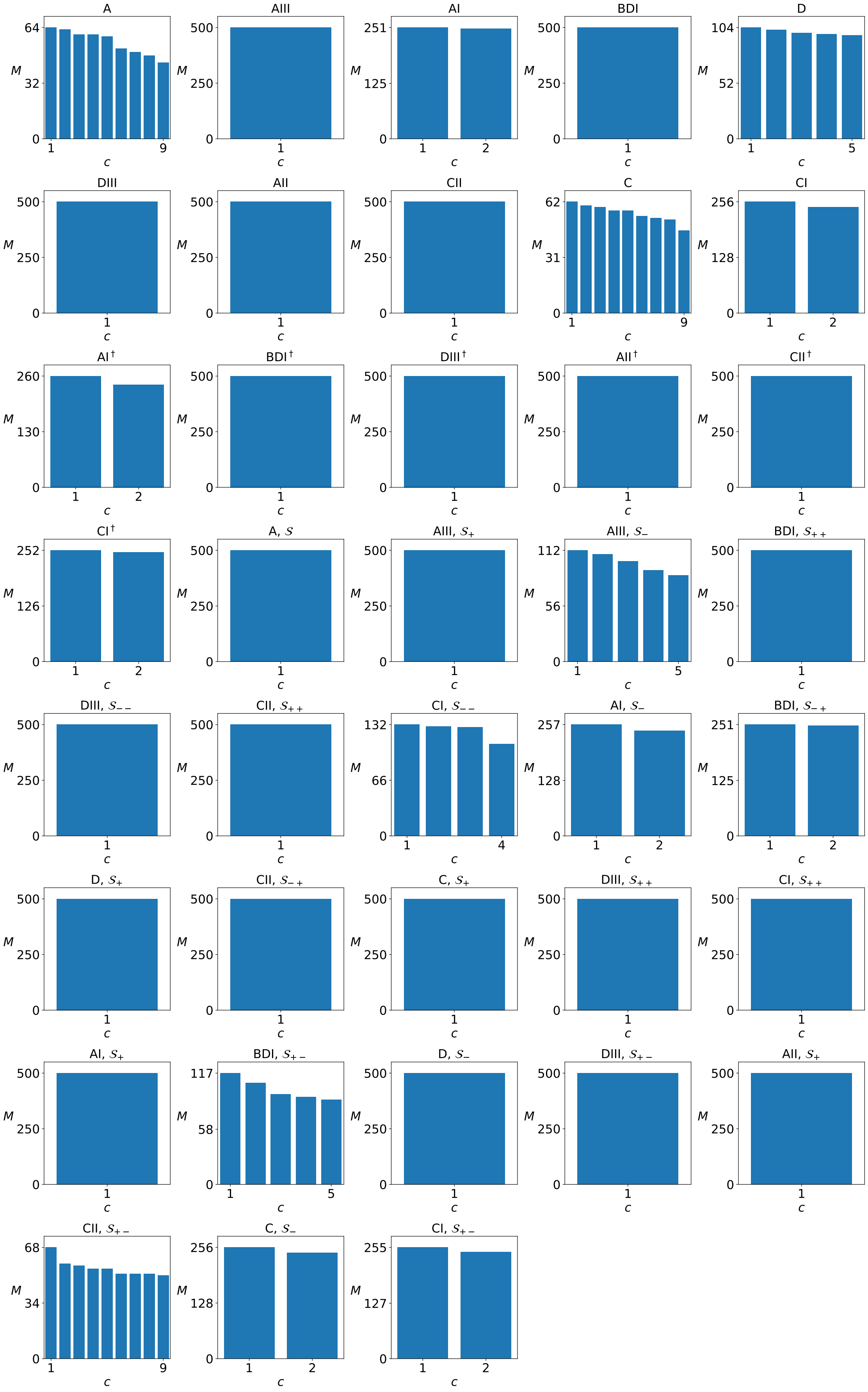

FIG. S35. Topological classifications for non-Hermitian topological systems in $d = 6$ dimension with a real line gap.

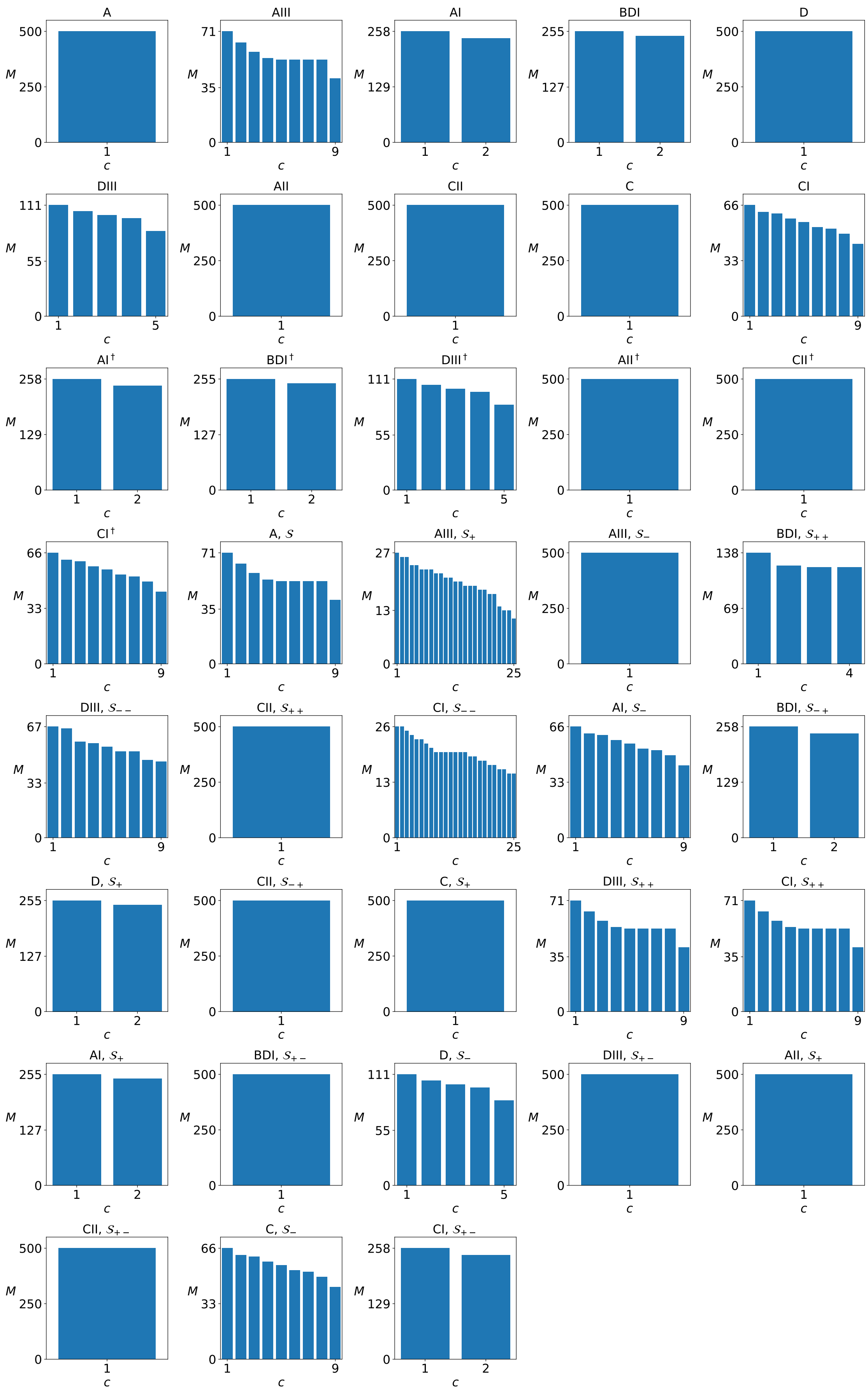

FIG. S36. Topological classifications for non-Hermitian topological systems in $d = 7$ dimension with a real line gap.

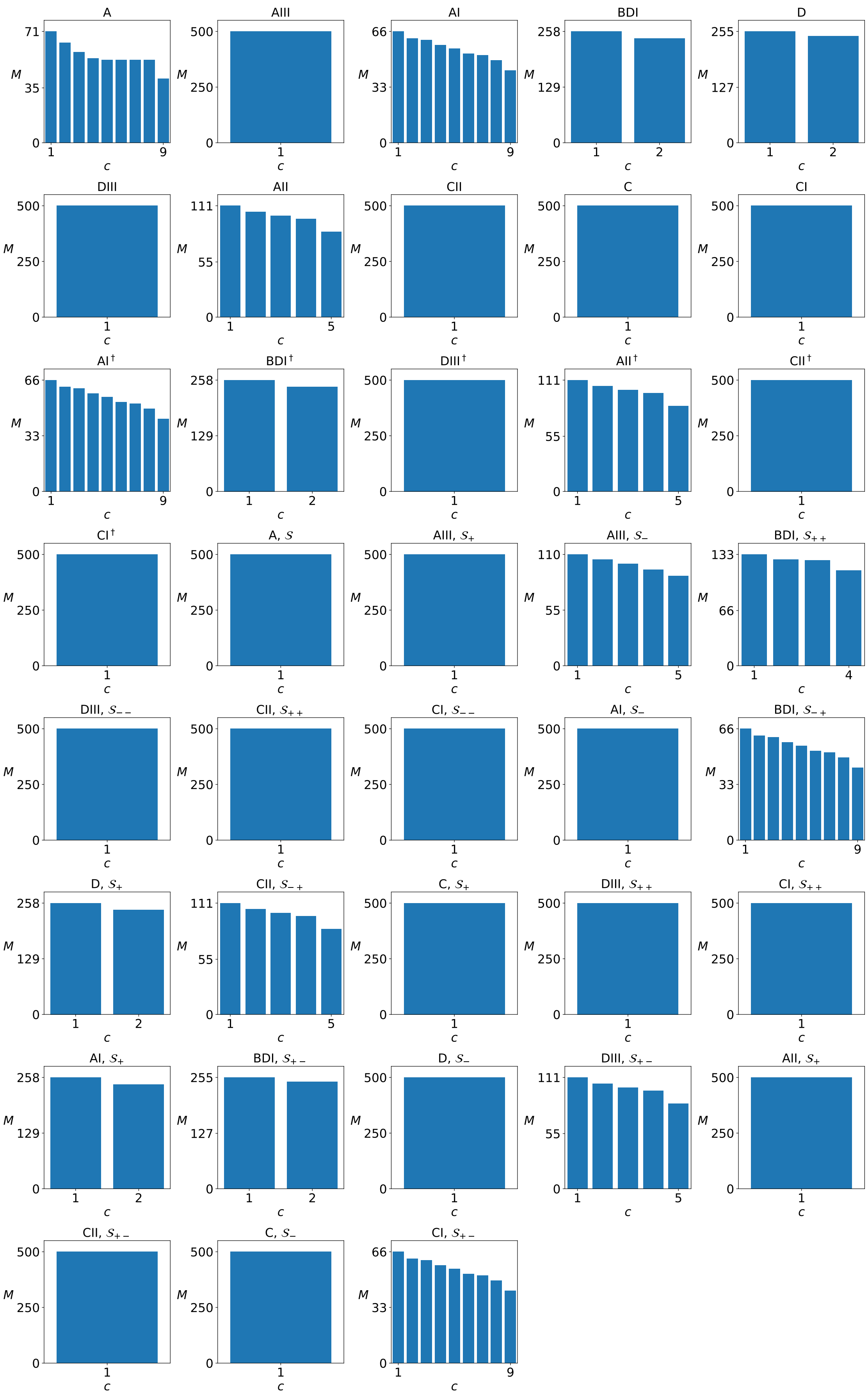

FIG. S37. Topological classifications for non-Hermitian topological systems in $d = 8$ dimension with a real line gap.

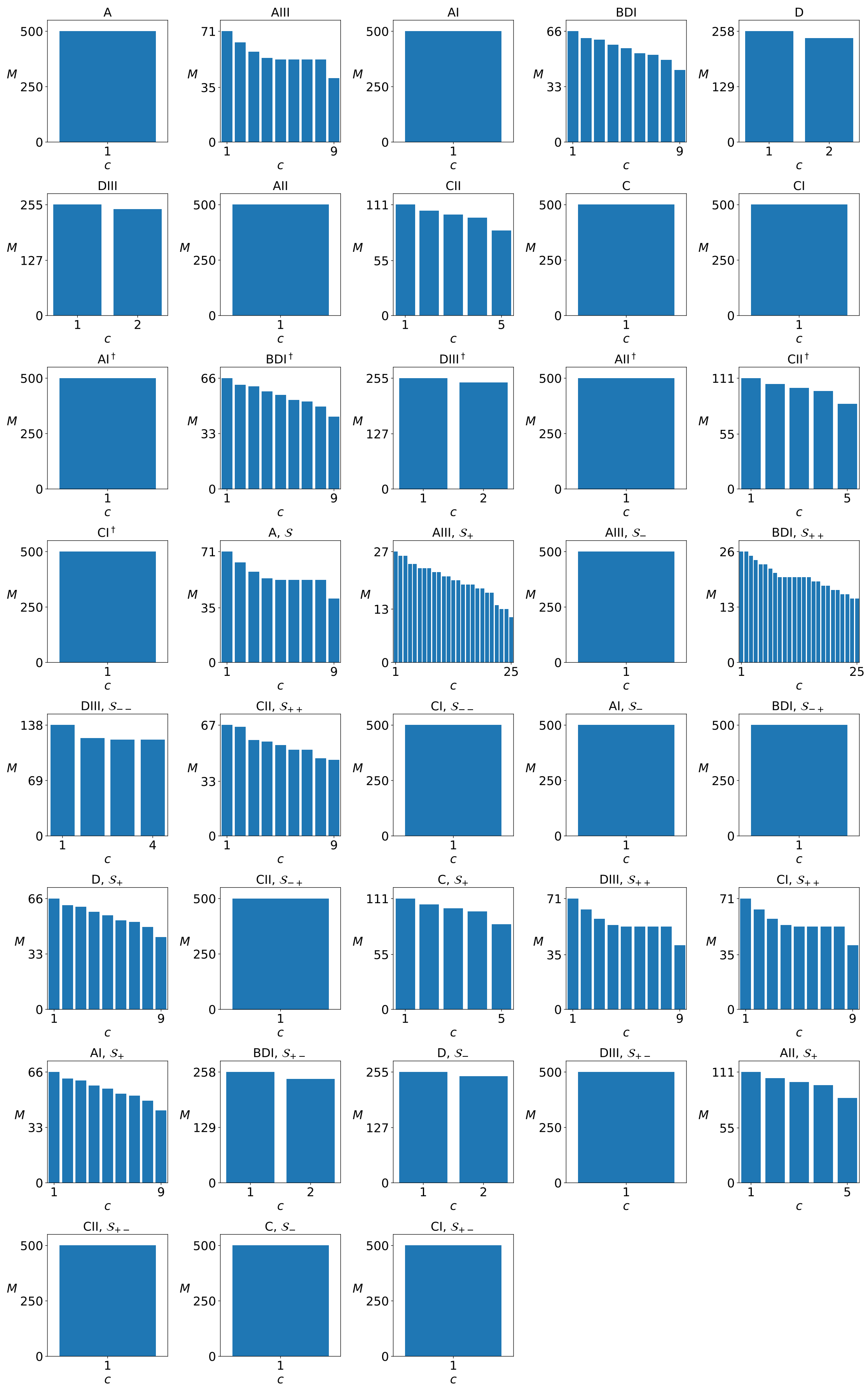

FIG. S38. Topological classifications for non-Hermitian topological systems in $d = 9$ dimension with a real line gap.

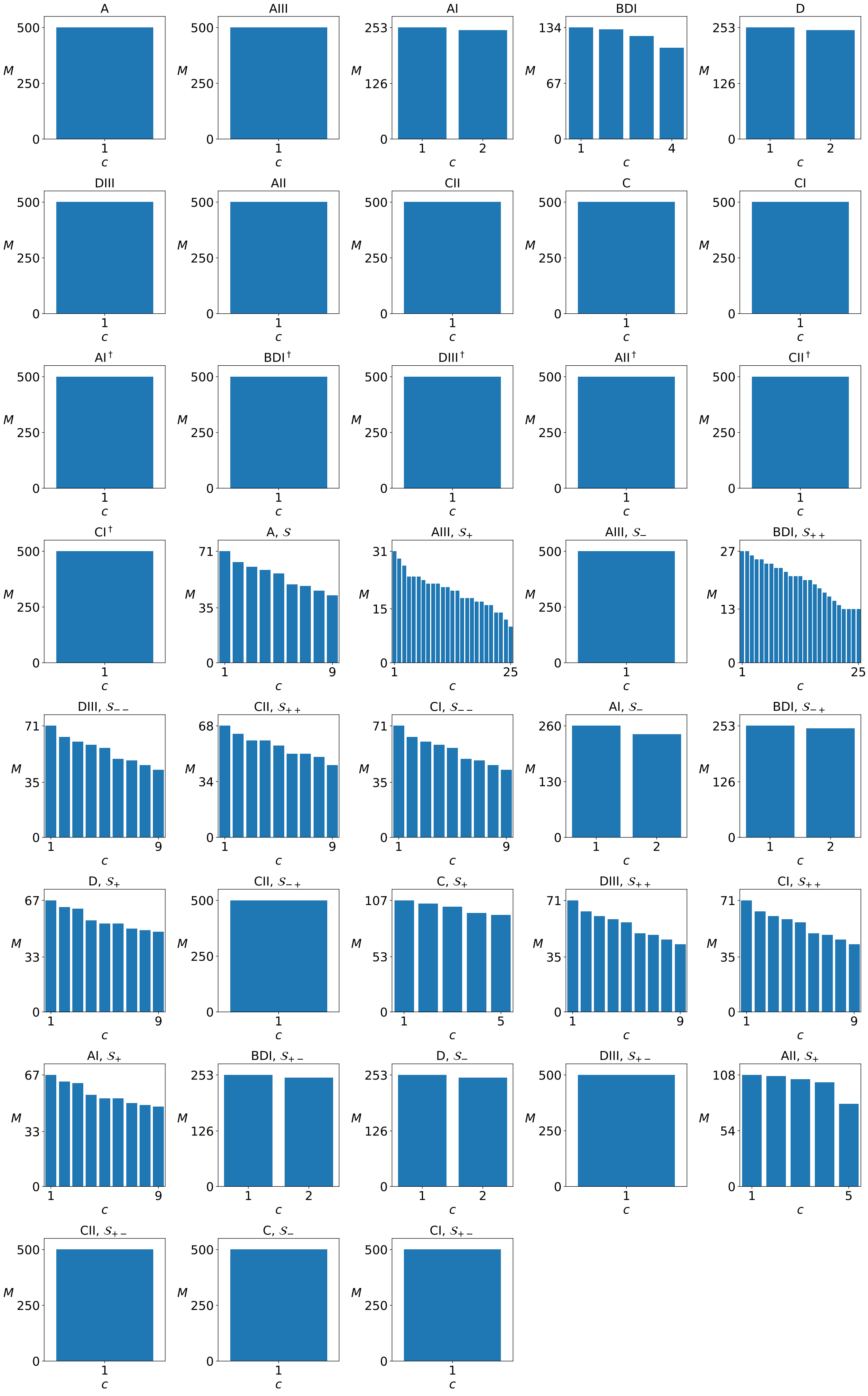

FIG. S39. Topological classifications for non-Hermitian topological systems in $d = 1$ dimension with an imaginary line gap.

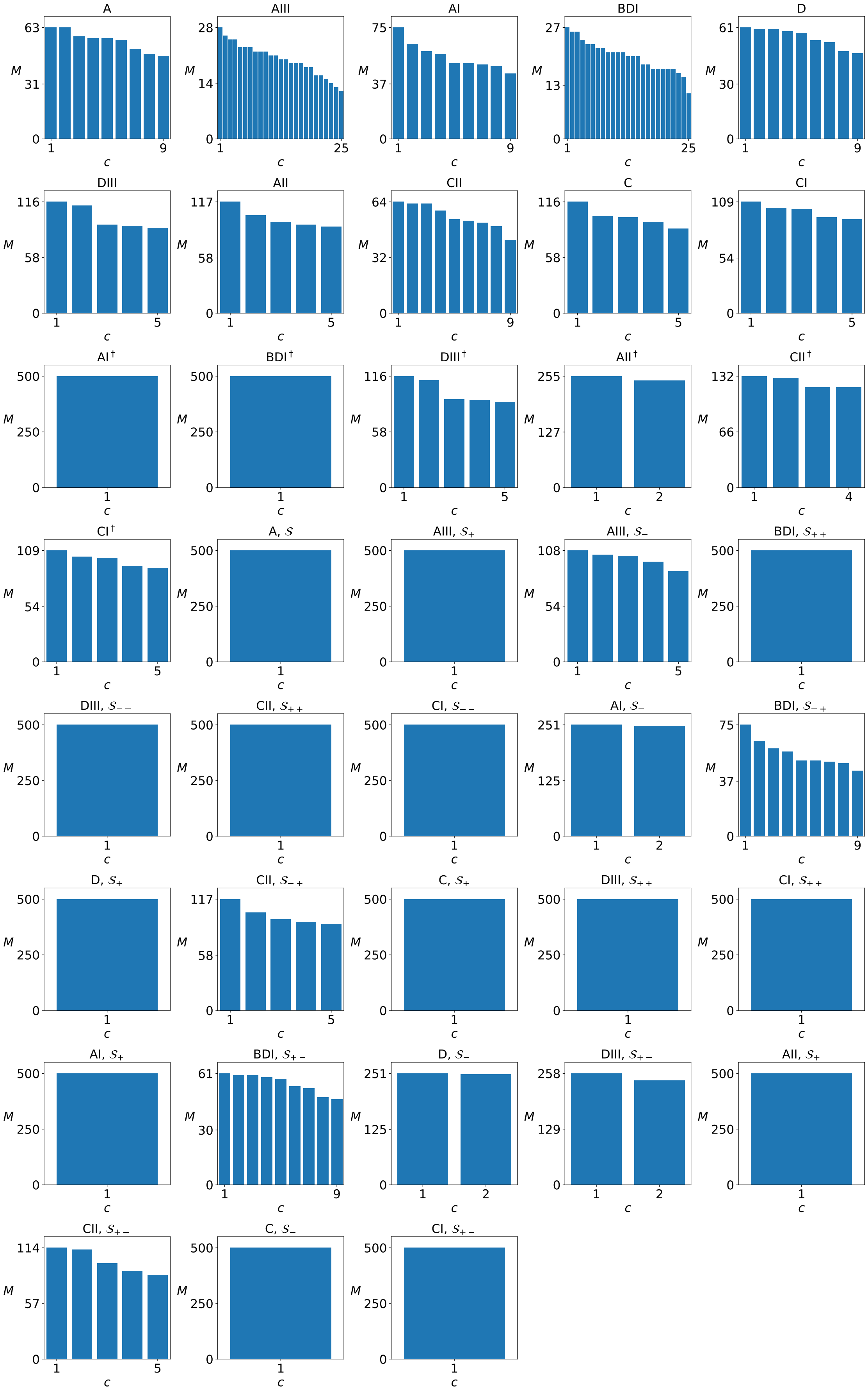

FIG. S40. Topological classifications for non-Hermitian topological systems in $d = 2$ dimension with an imaginary line gap.

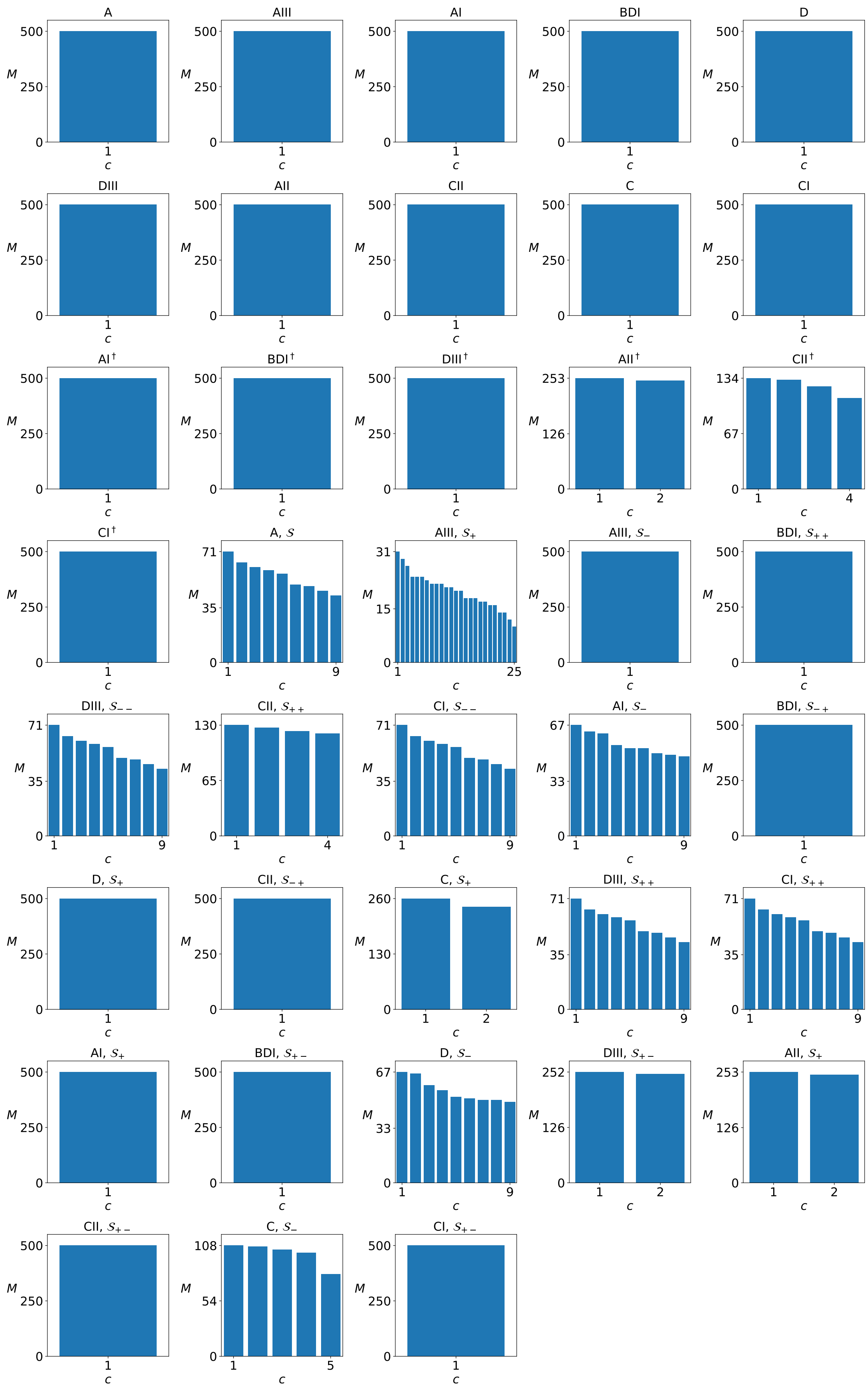

FIG. S41. Topological classifications for non-Hermitian topological systems in $d = 3$ dimension with an imaginary line gap.

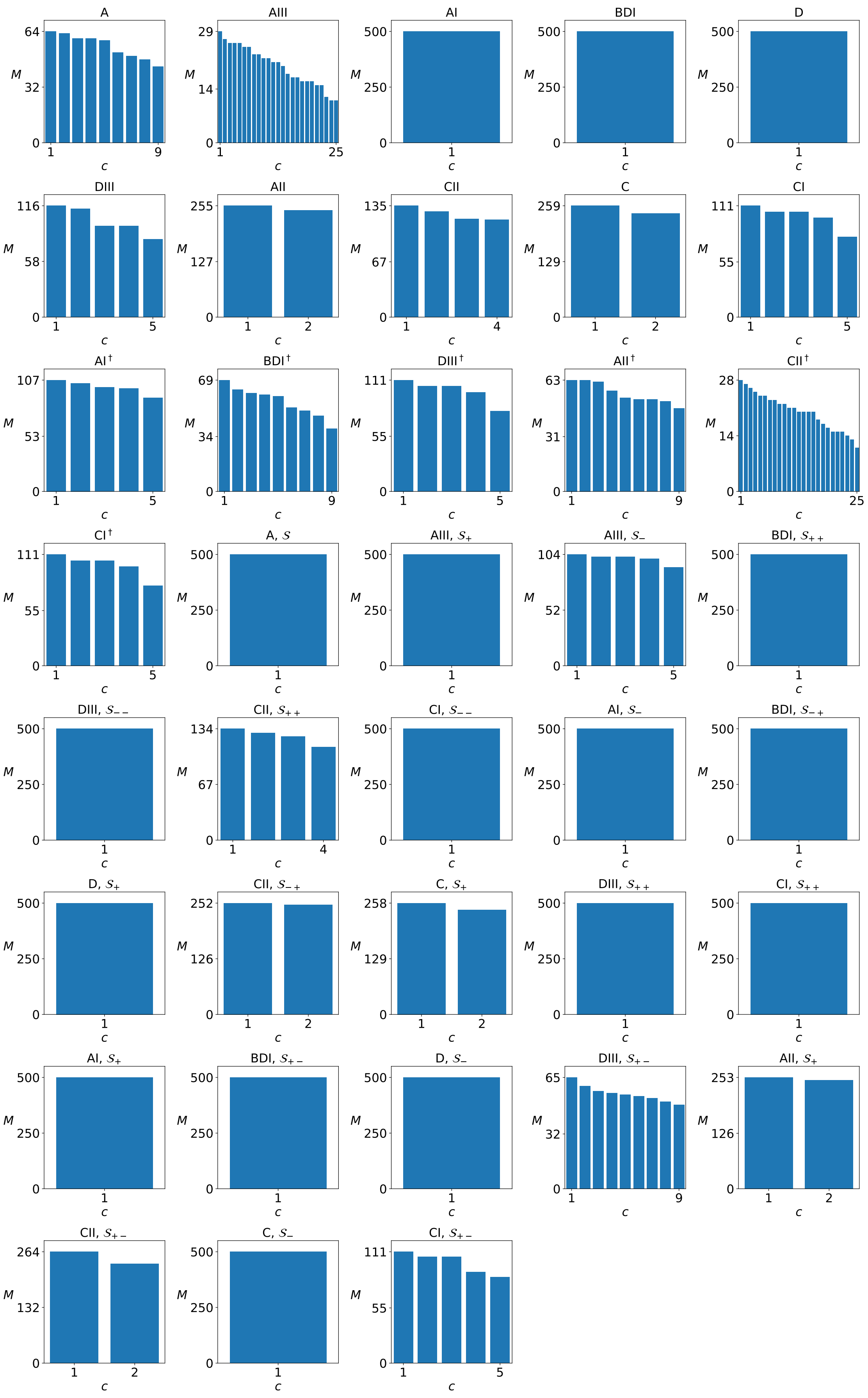

FIG. S42. Topological classifications for non-Hermitian topological systems in $d = 4$ dimension with an imaginary line gap.

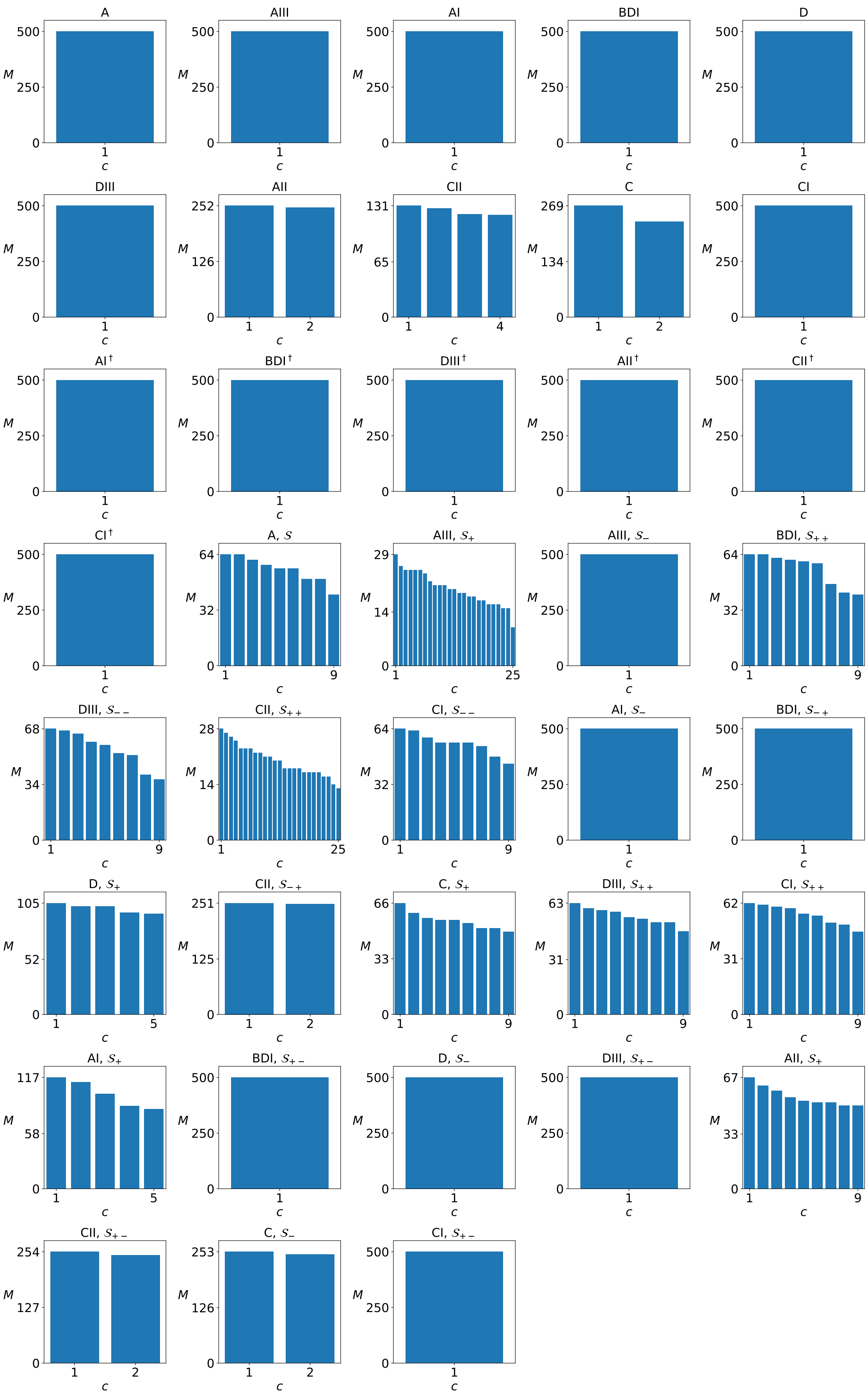

FIG. S43. Topological classifications for non-Hermitian topological systems in $d = 5$ dimension with an imaginary line gap.

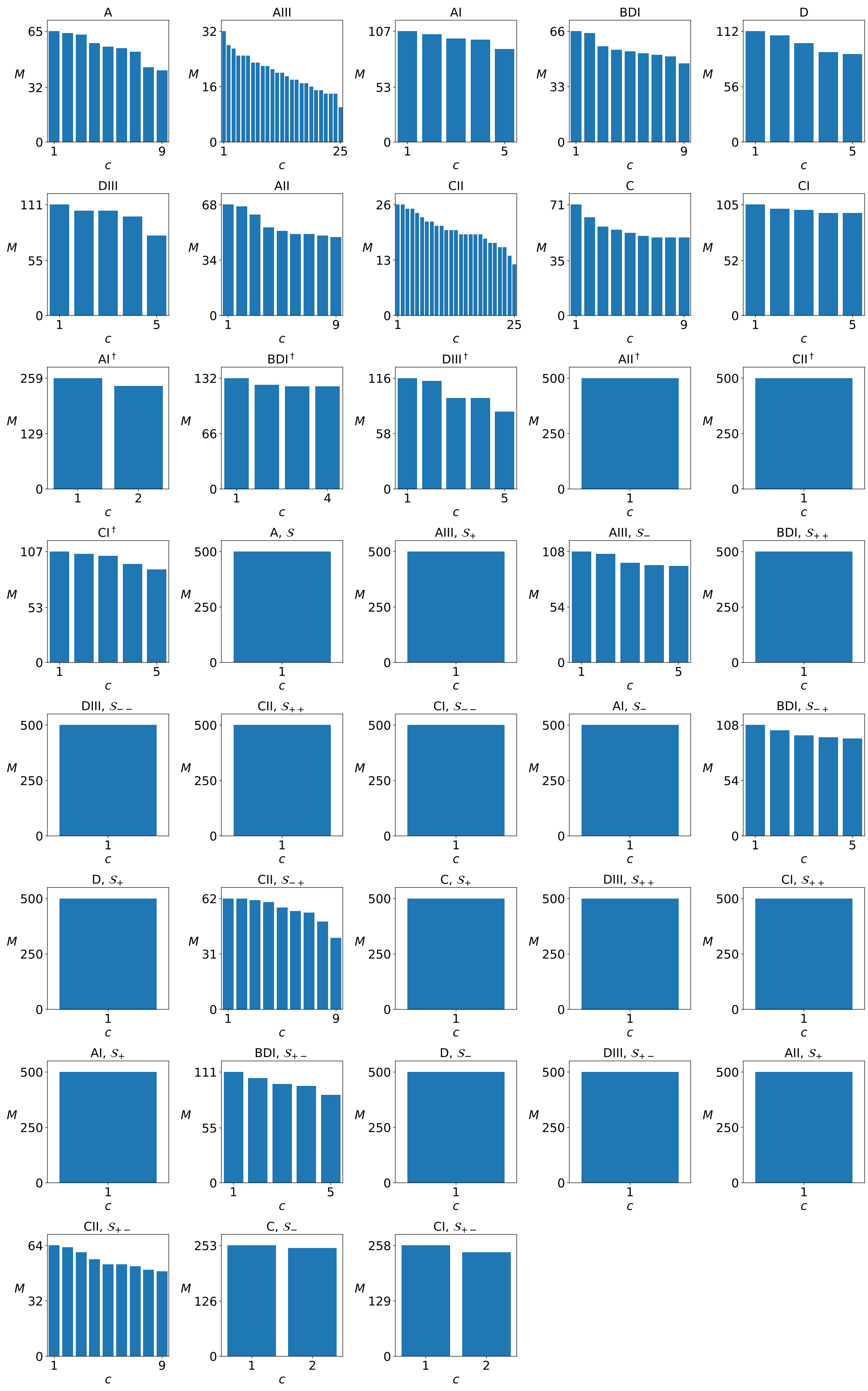

FIG. S44. Topological classifications for non-Hermitian topological systems in $d = 6$ dimension with an imaginary line gap.

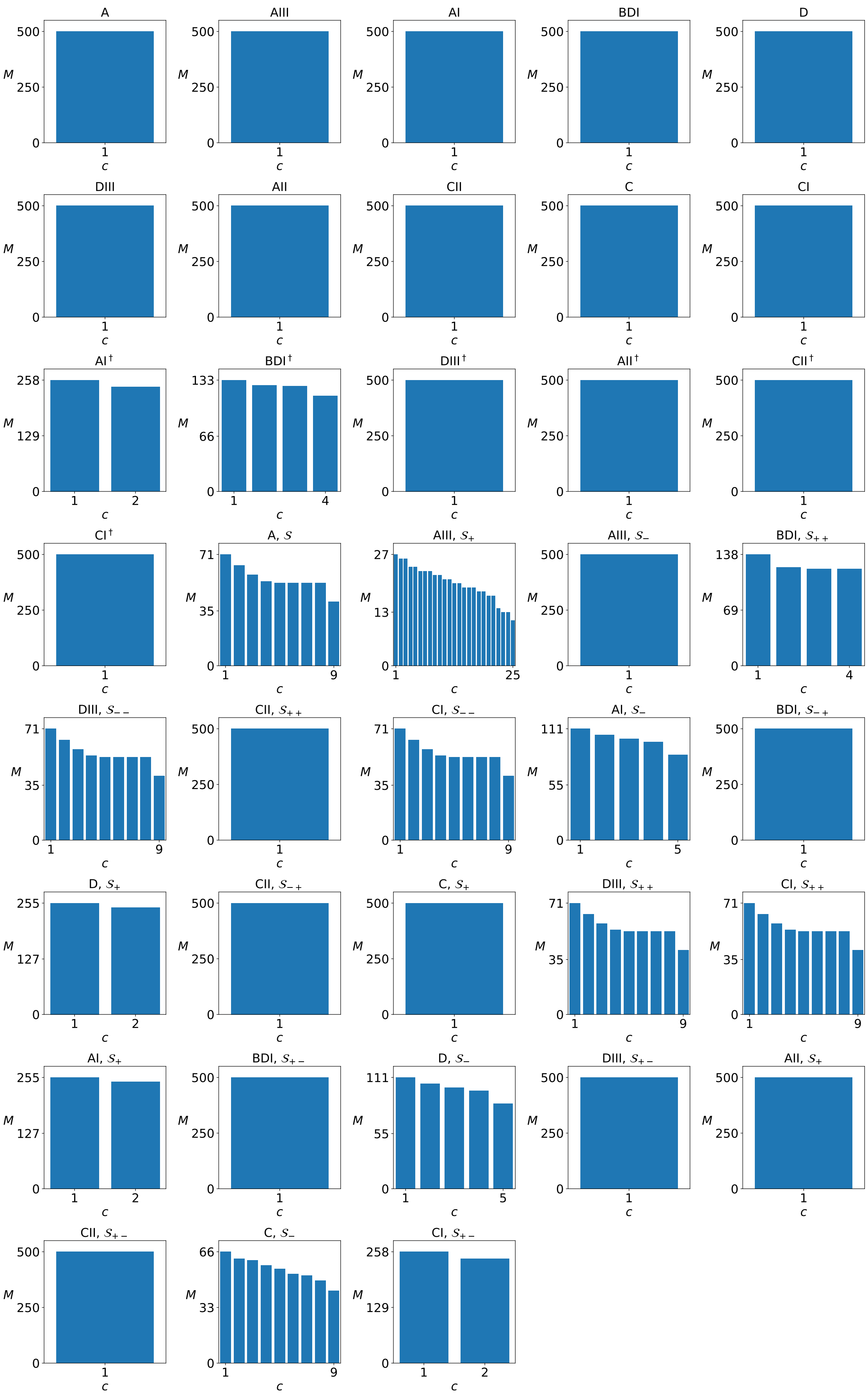

FIG. S45. Topological classifications for non-Hermitian topological systems in $d = 7$ dimension with an imaginary line gap.

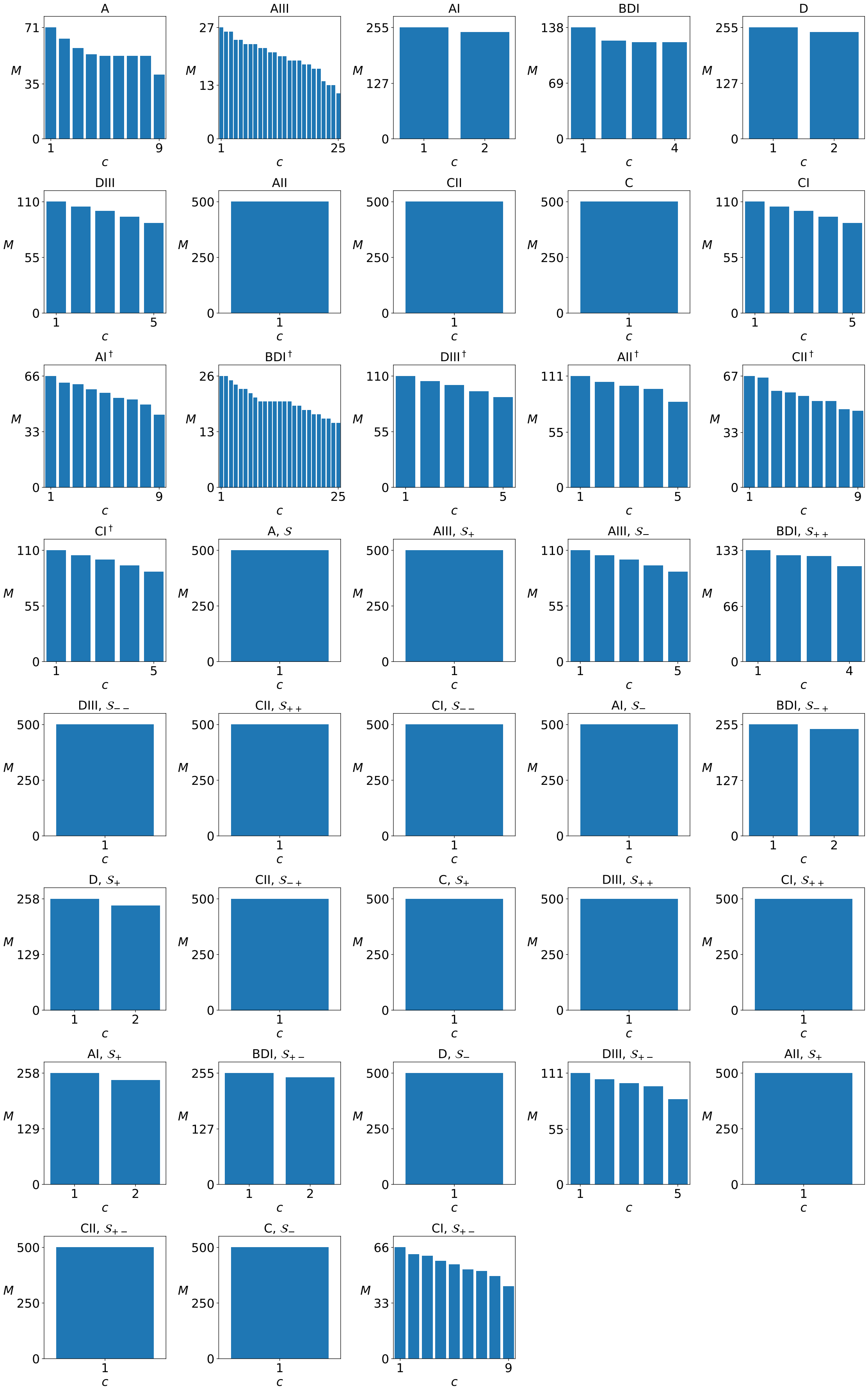

FIG. S46. Topological classifications for non-Hermitian topological systems in $d = 8$ dimension with an imaginary line gap.

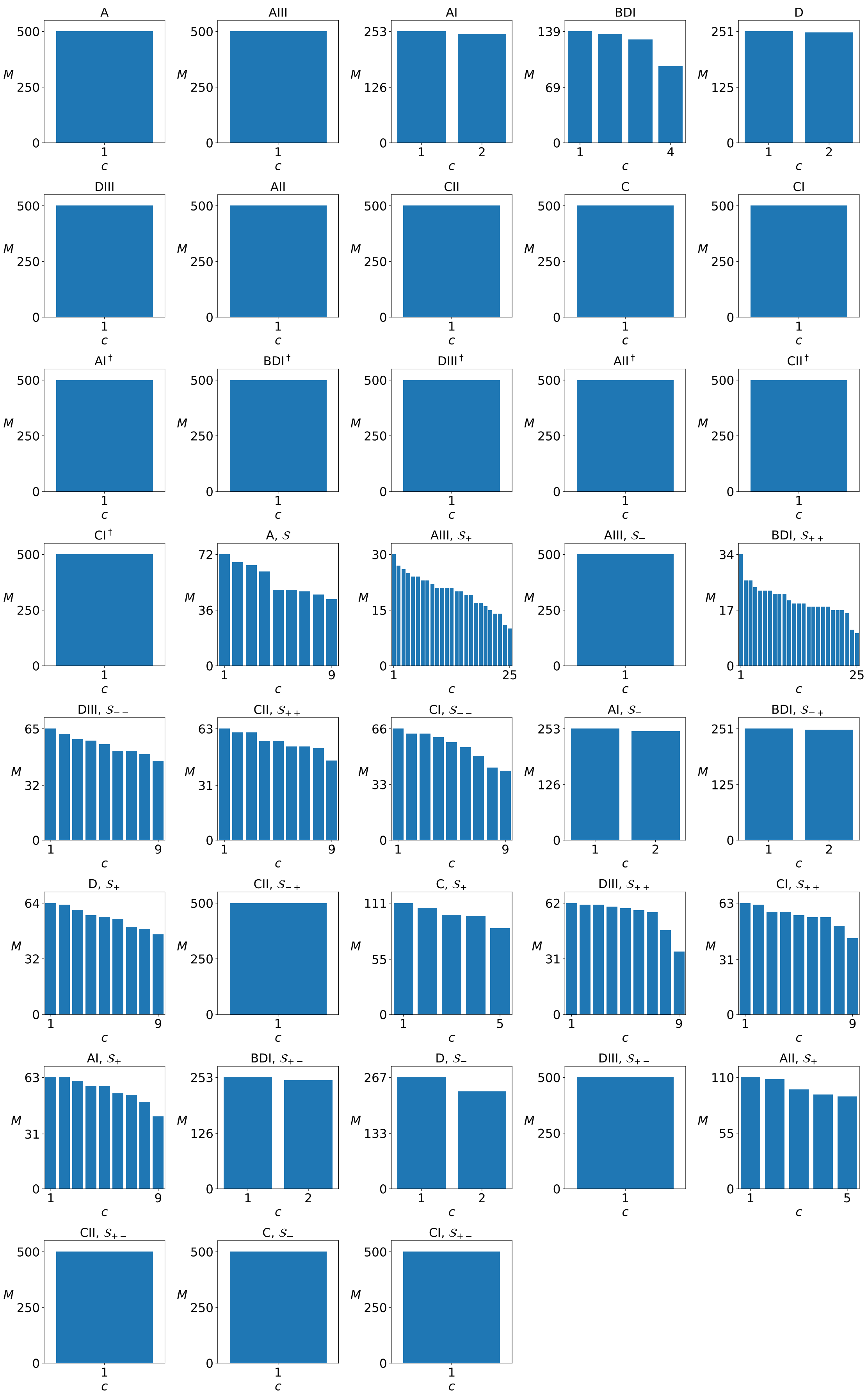

FIG. S47. Topological classifications for non-Hermitian topological systems in $d = 9$ dimension with an imaginary line gap.

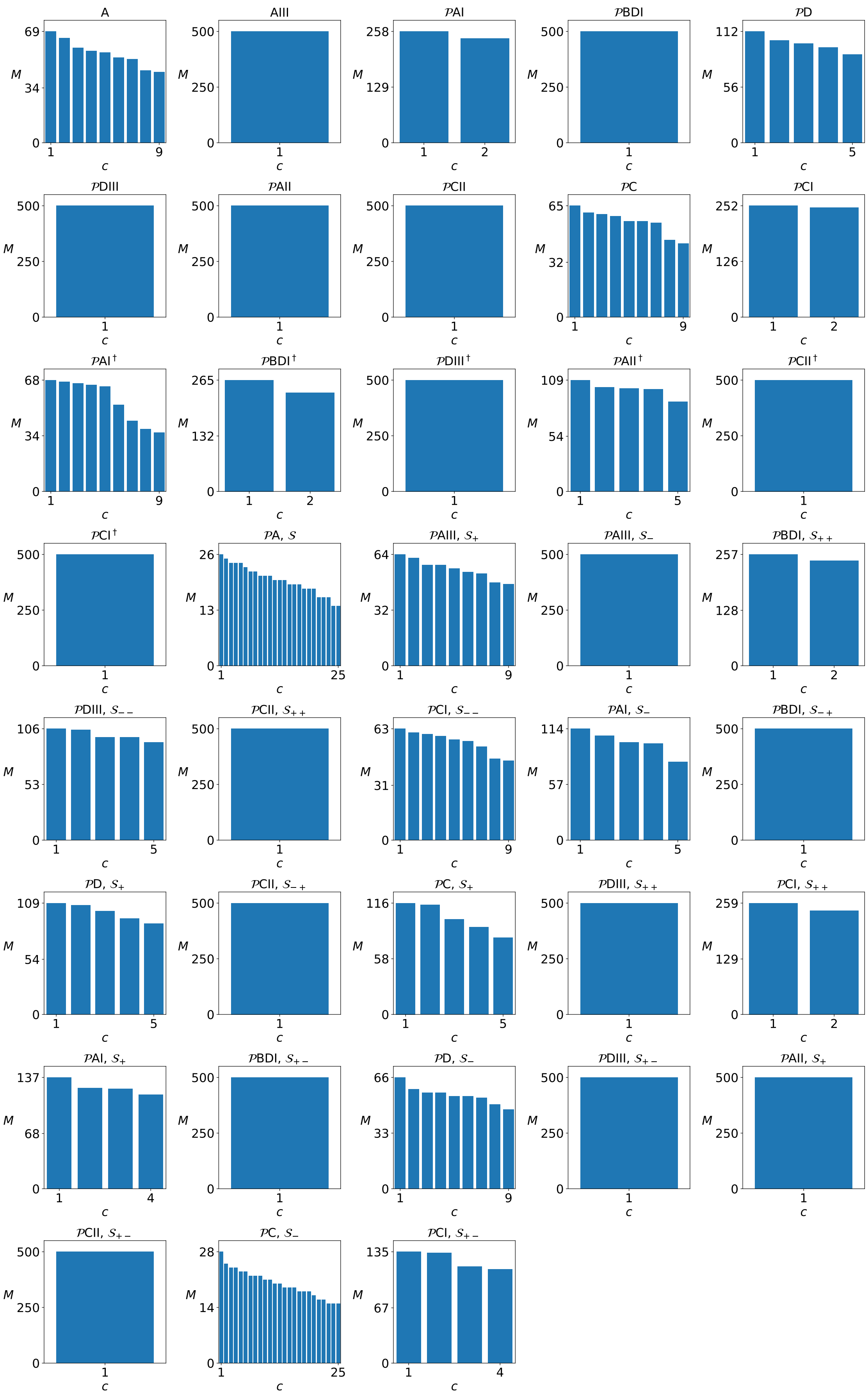

FIG. S48. Topological classifications for non-Hermitian topological systems in $d = 1$ dimension with a point gap after considering the parity transformation.

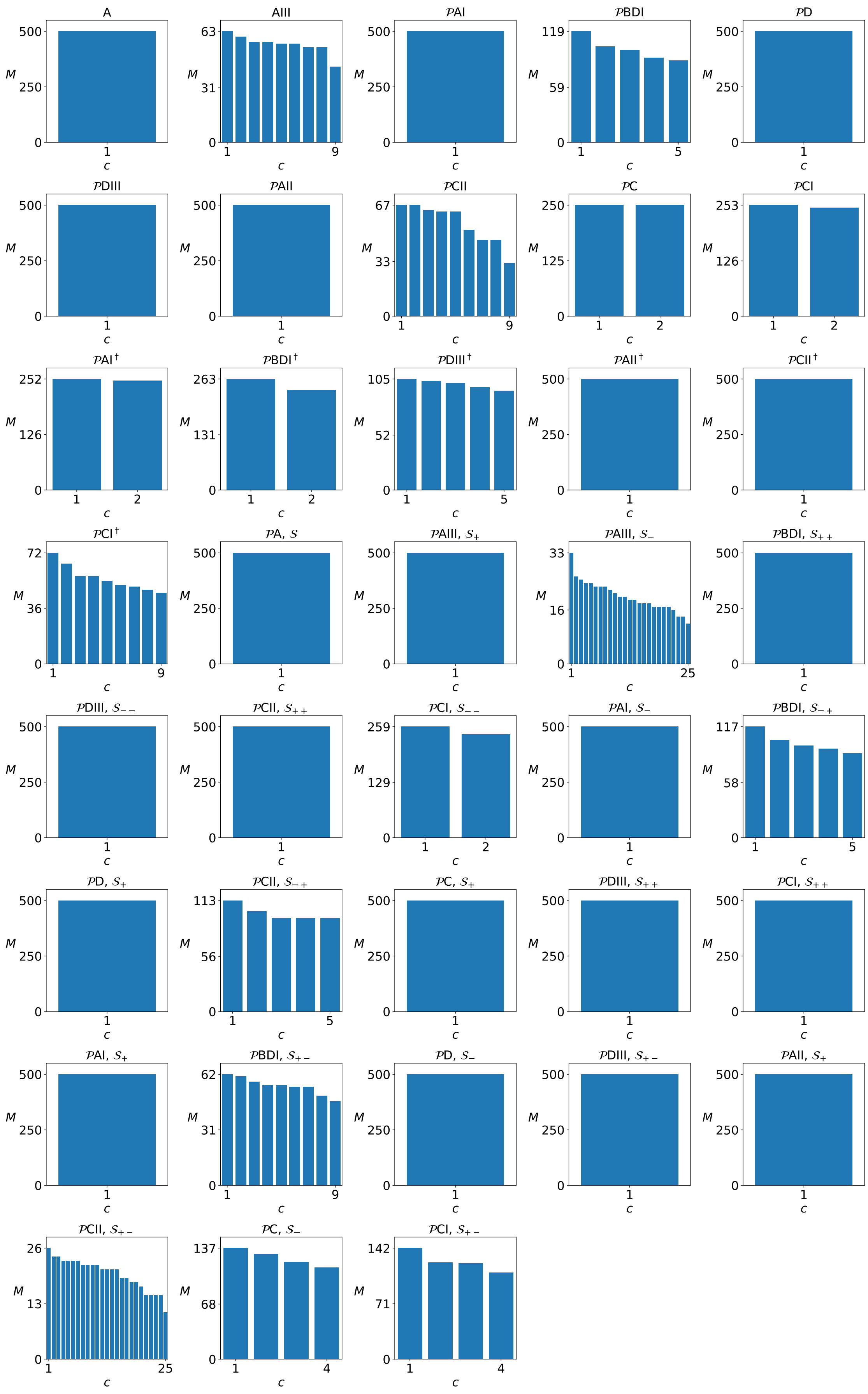

FIG. S49. Topological classifications for non-Hermitian topological systems in $d = 2$ dimension with a point gap after considering the parity transformation.

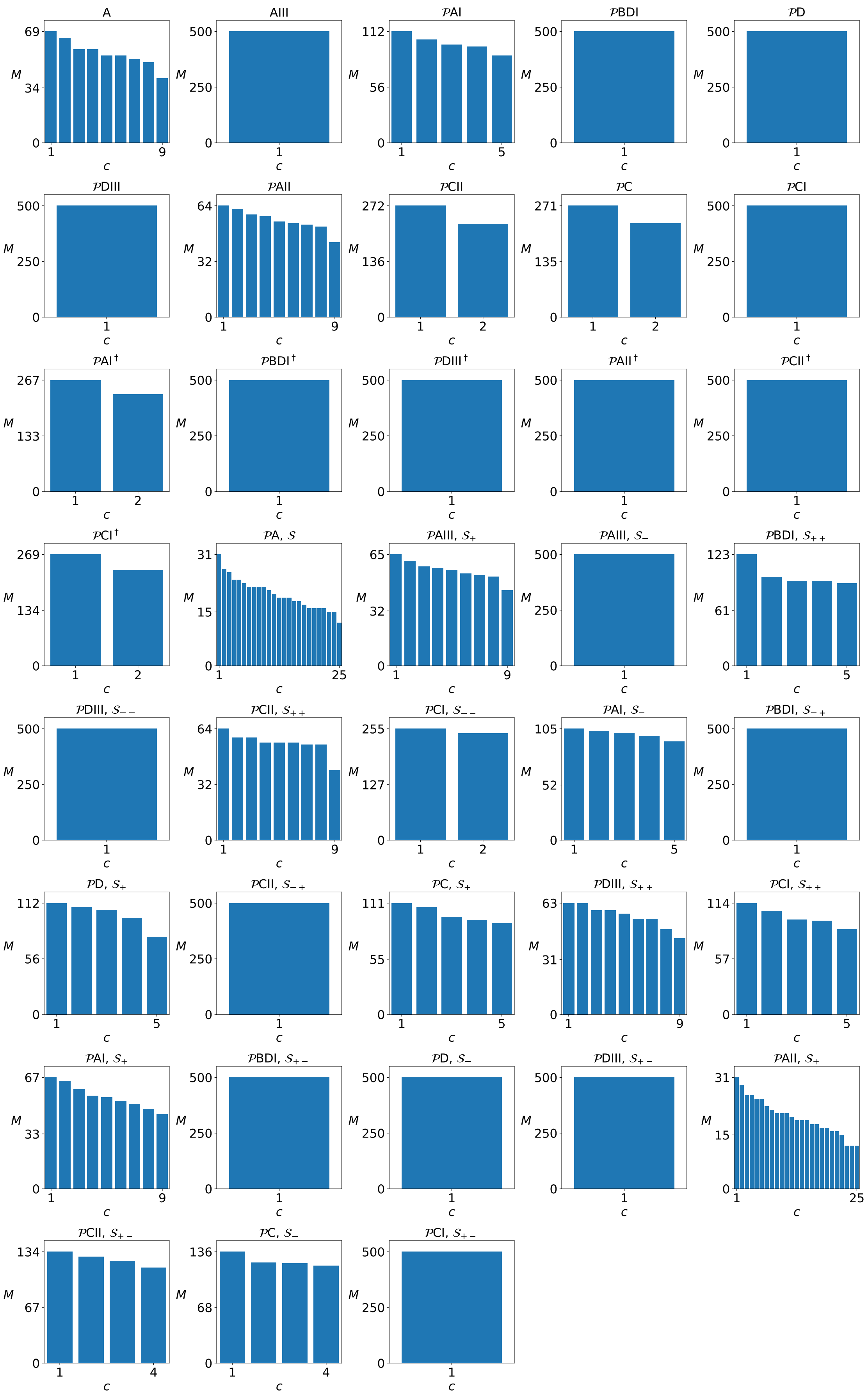

FIG. S50. Topological classifications for non-Hermitian topological systems in $d = 3$ dimension with a point gap after considering the parity transformation.

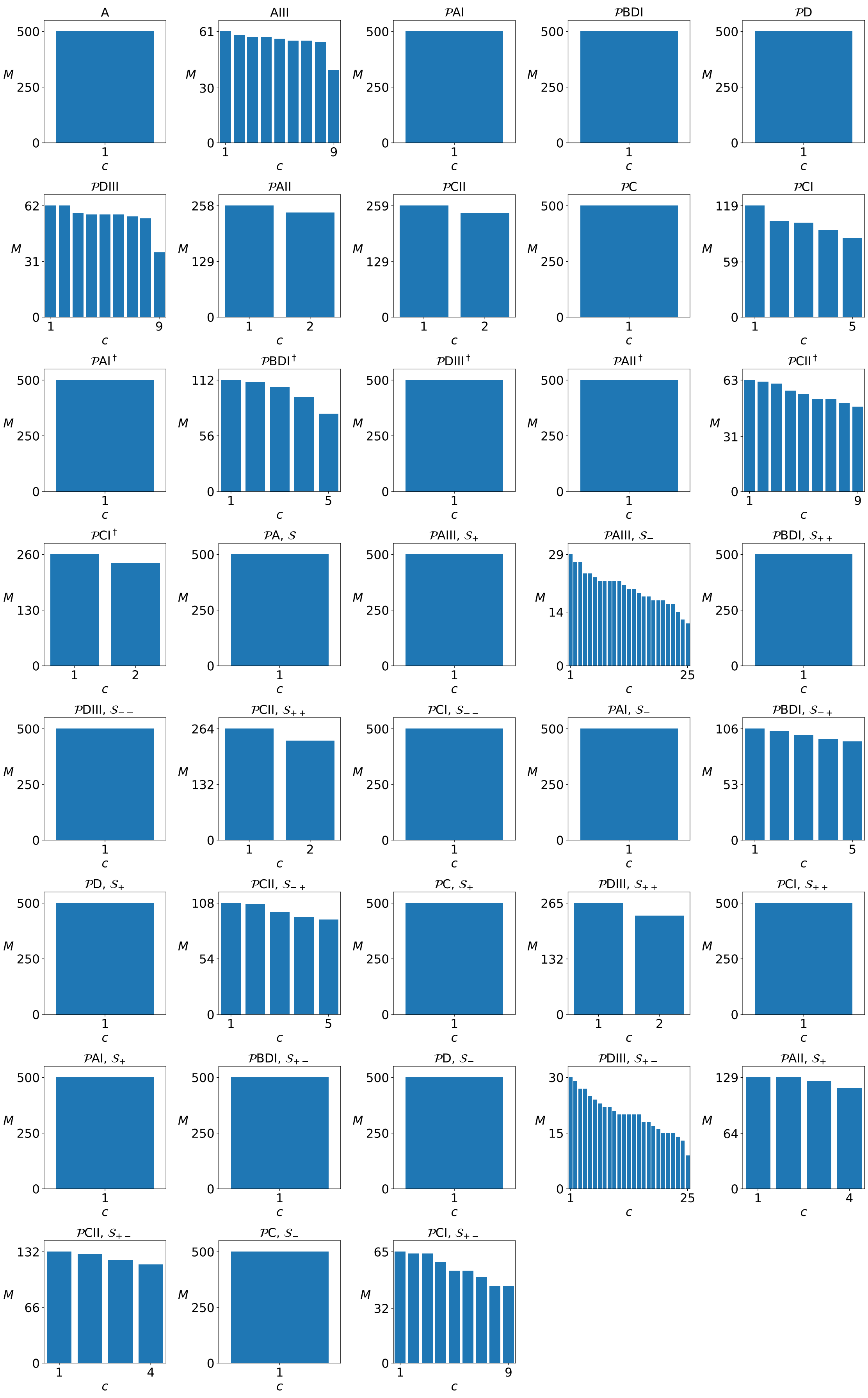

FIG. S51. Topological classifications for non-Hermitian topological systems in $d = 4$ dimension with a point gap after considering the parity transformation.

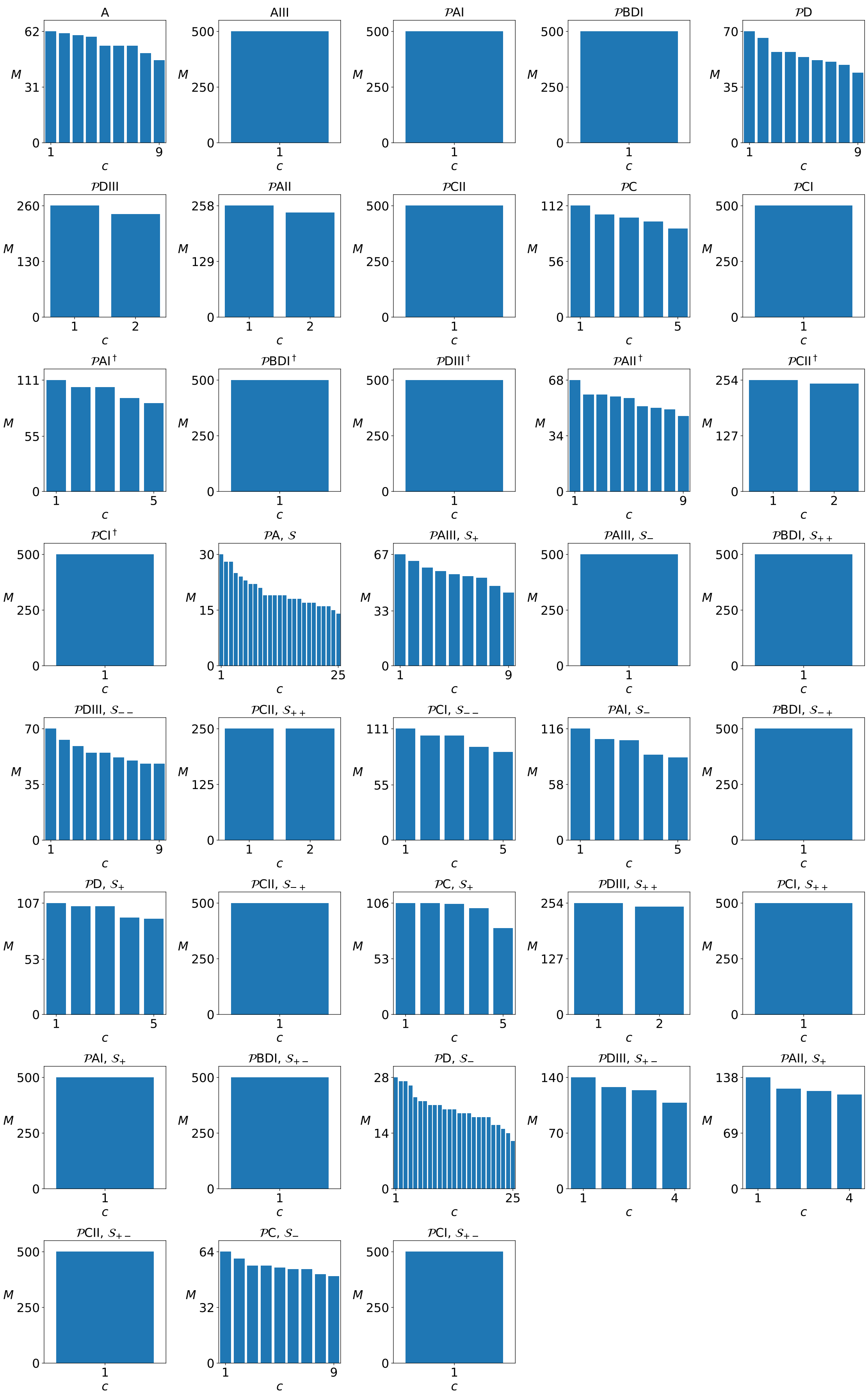

FIG. S52. Topological classifications for non-Hermitian topological systems in $d = 5$ dimension with a point gap after considering the parity transformation.

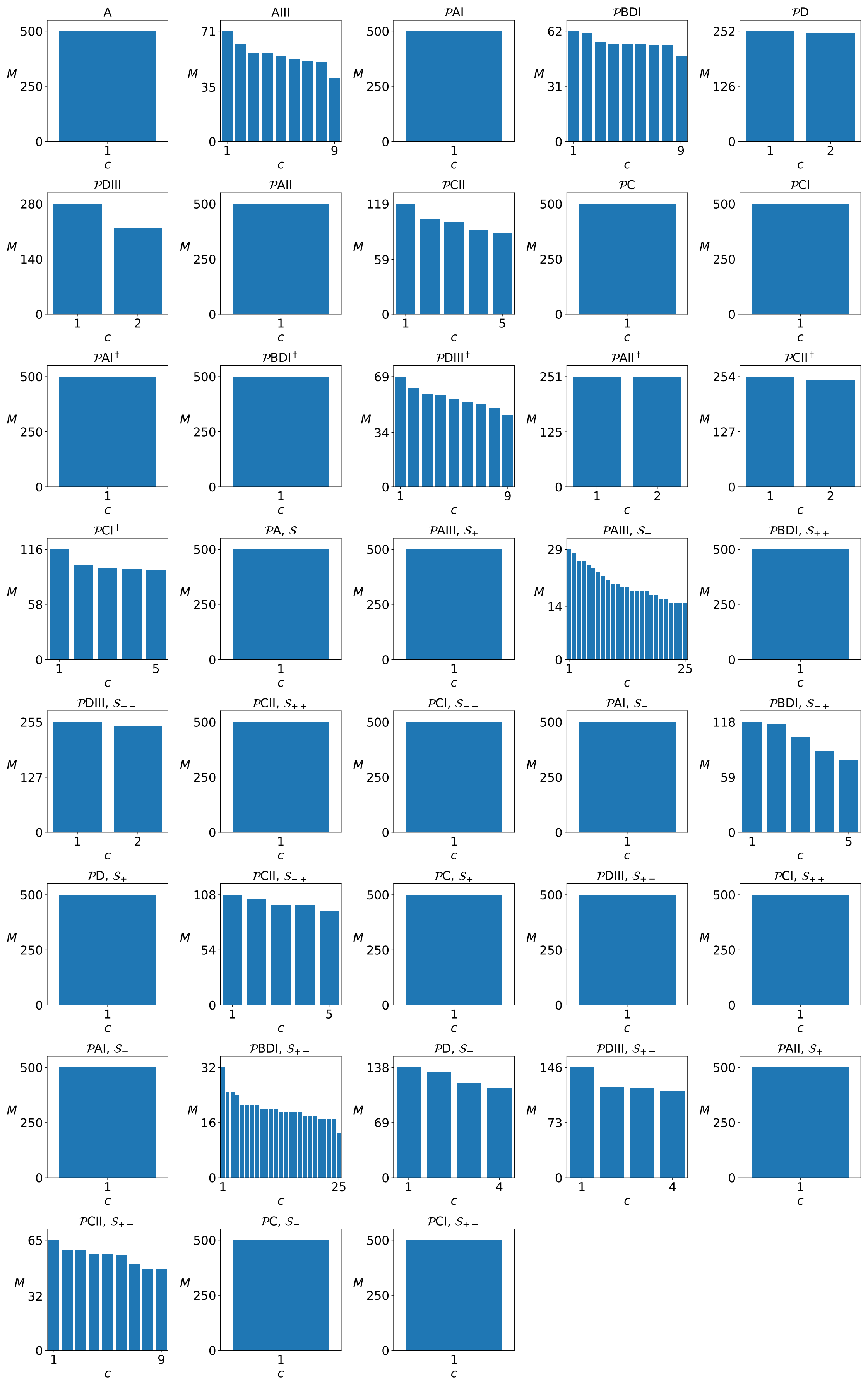

FIG. S53. Topological classifications for non-Hermitian topological systems in $d = 6$ dimension with a point gap after considering the parity transformation.

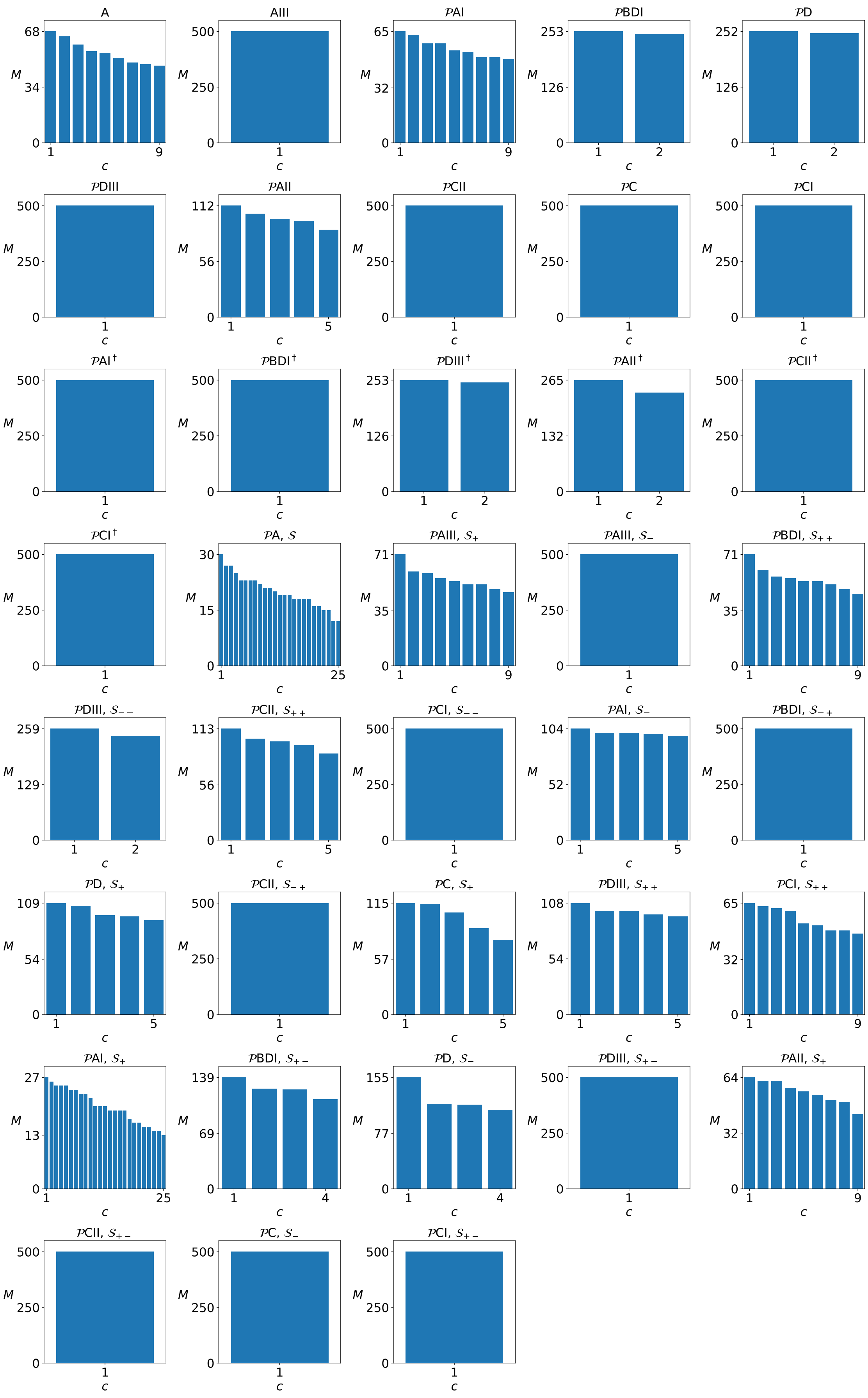

FIG. S54. Topological classifications for non-Hermitian topological systems in $d = 7$ dimension with a point gap after considering the parity transformation.

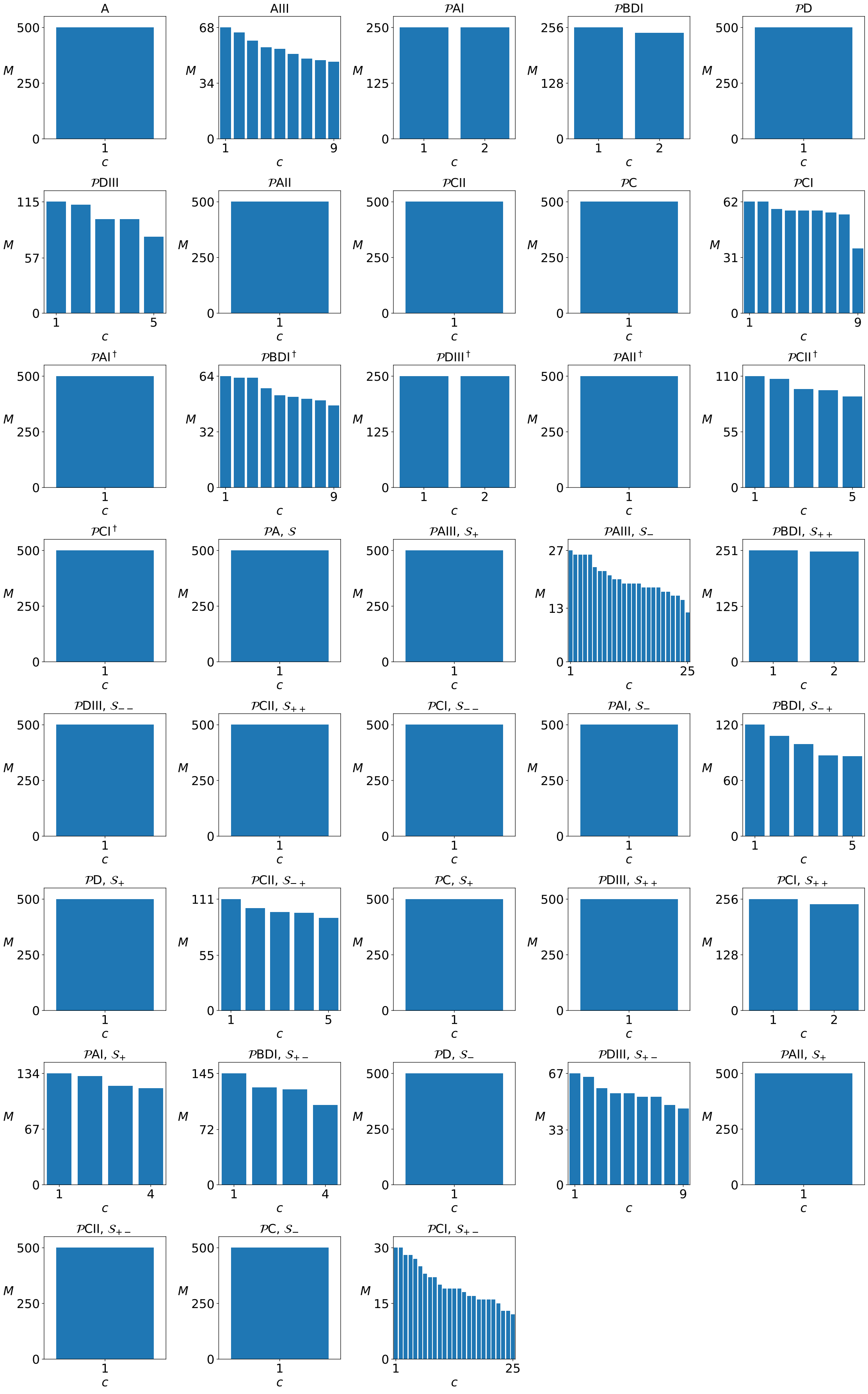

FIG. S55. Topological classifications for non-Hermitian topological systems in $d = 8$ dimension with a point gap after considering the parity transformation.

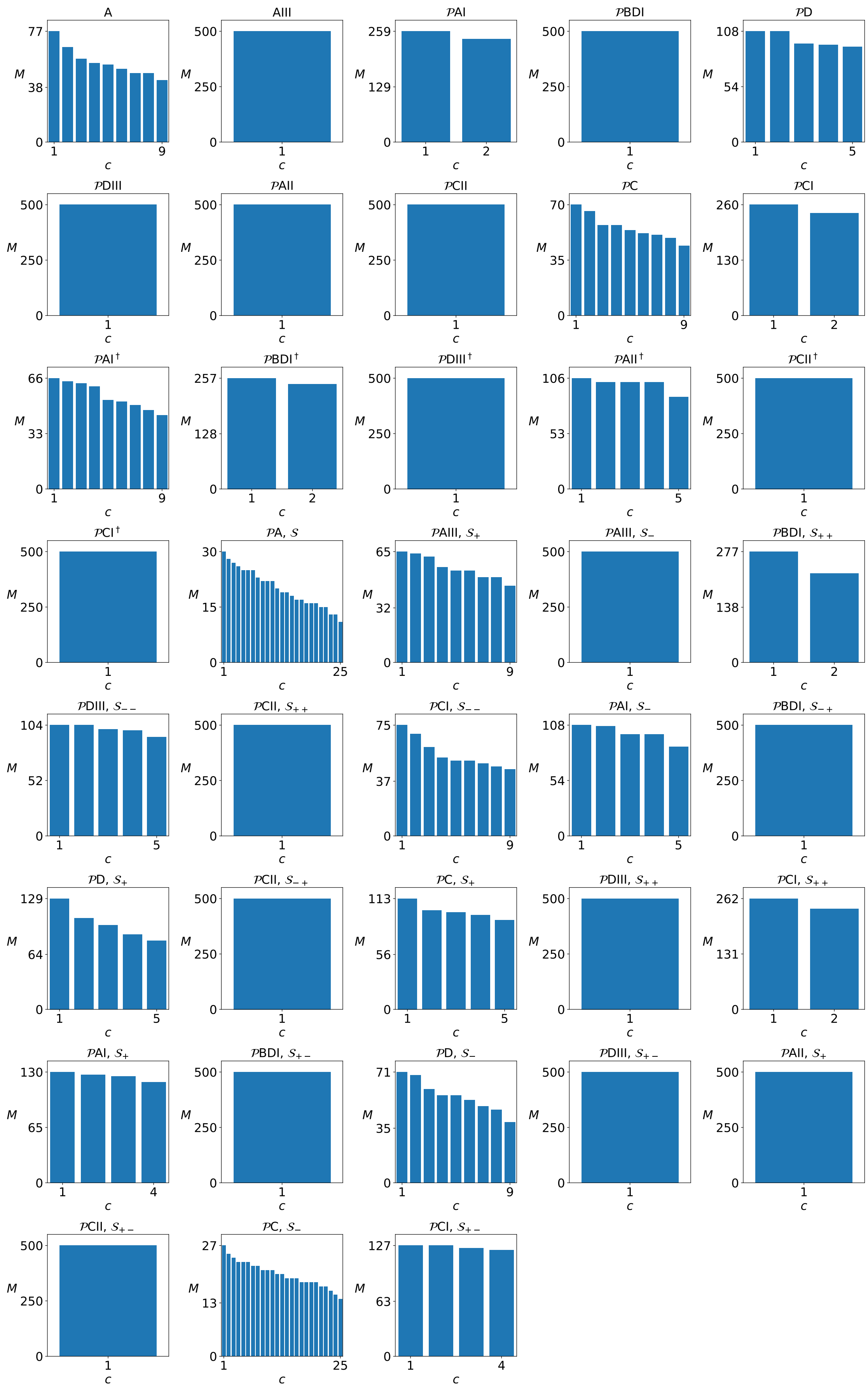

FIG. S56. Topological classifications for non-Hermitian topological systems in $d = 9$ dimension with a point gap after considering the parity transformation.

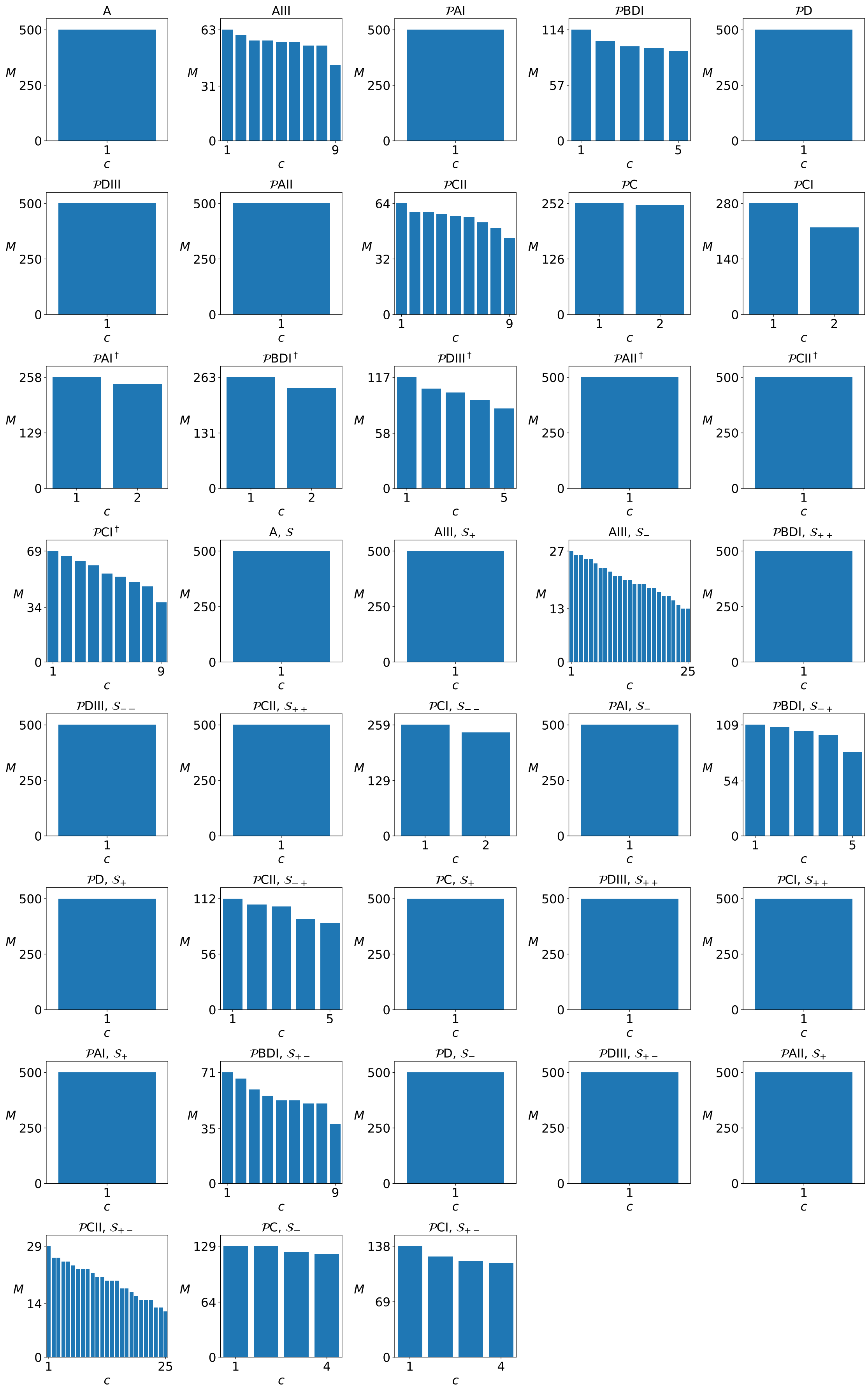

FIG. S57. Topological classifications for non-Hermitian topological systems in $d = 10$ dimension with a point gap after considering the parity transformation.

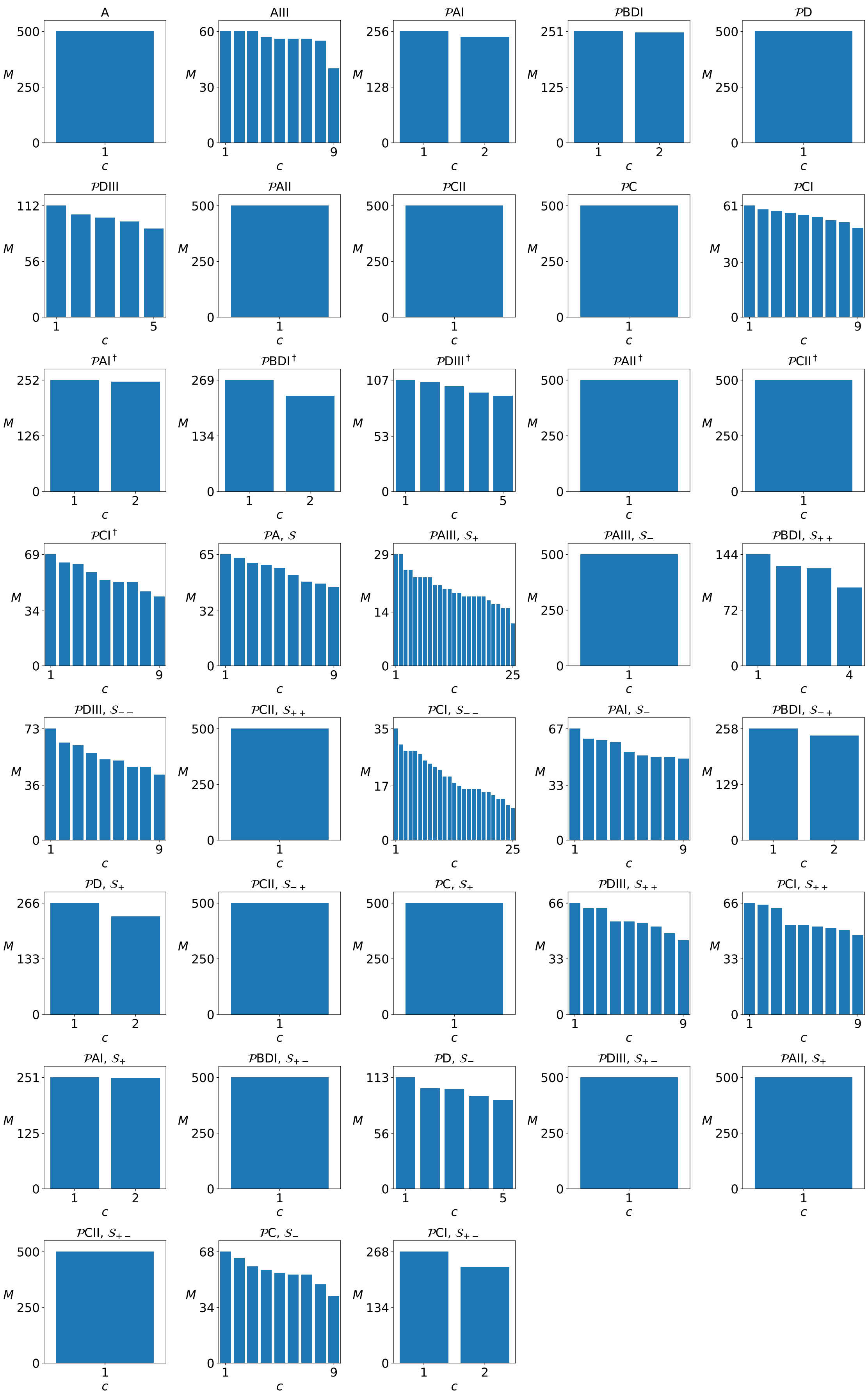

FIG. S58. Topological classifications for non-Hermitian topological systems in $d = 1$ dimension with a real line gap after considering the parity transformation.

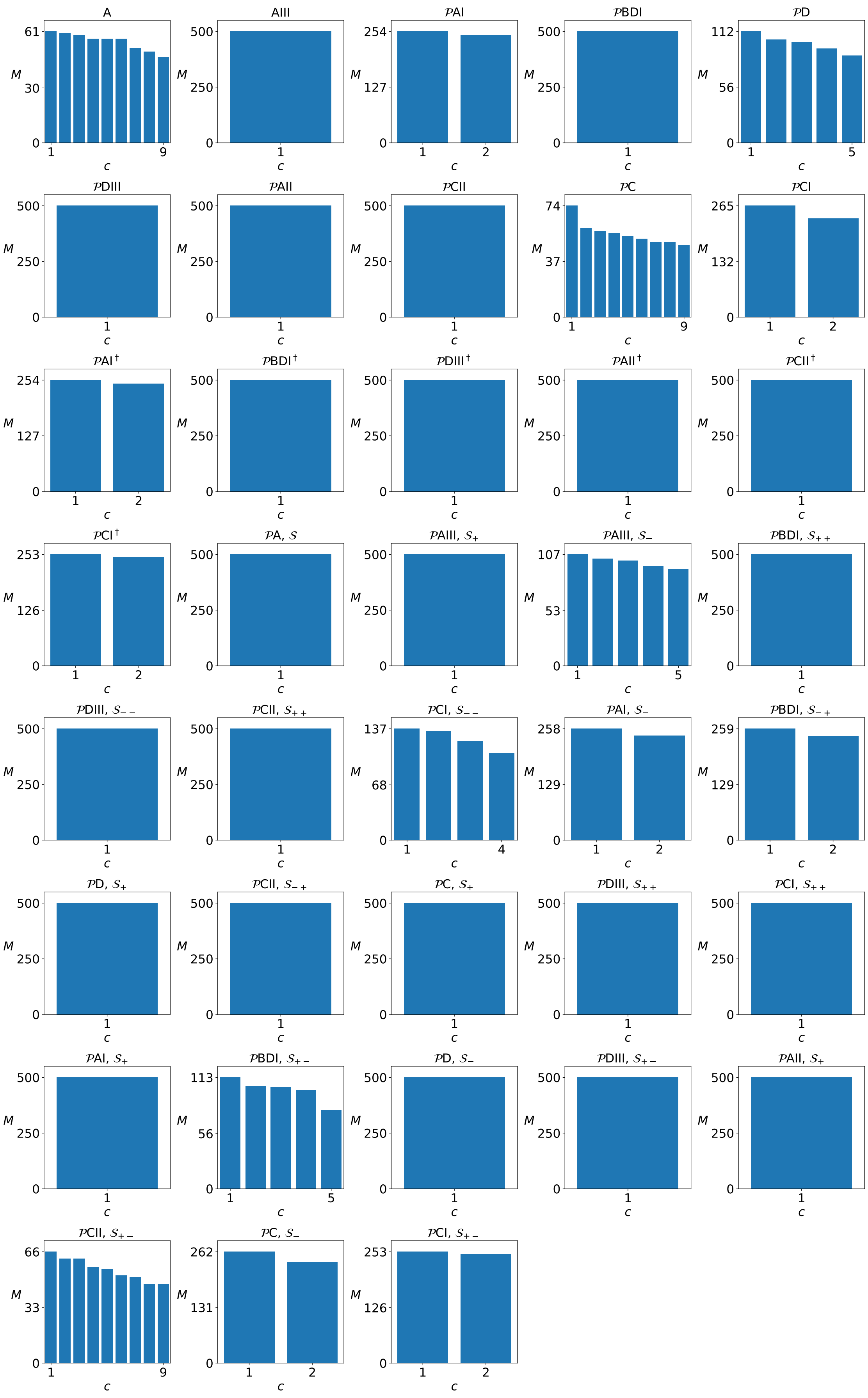

FIG. S59. Topological classifications for non-Hermitian topological systems in $d = 2$ dimension with a real line gap after considering the parity transformation.

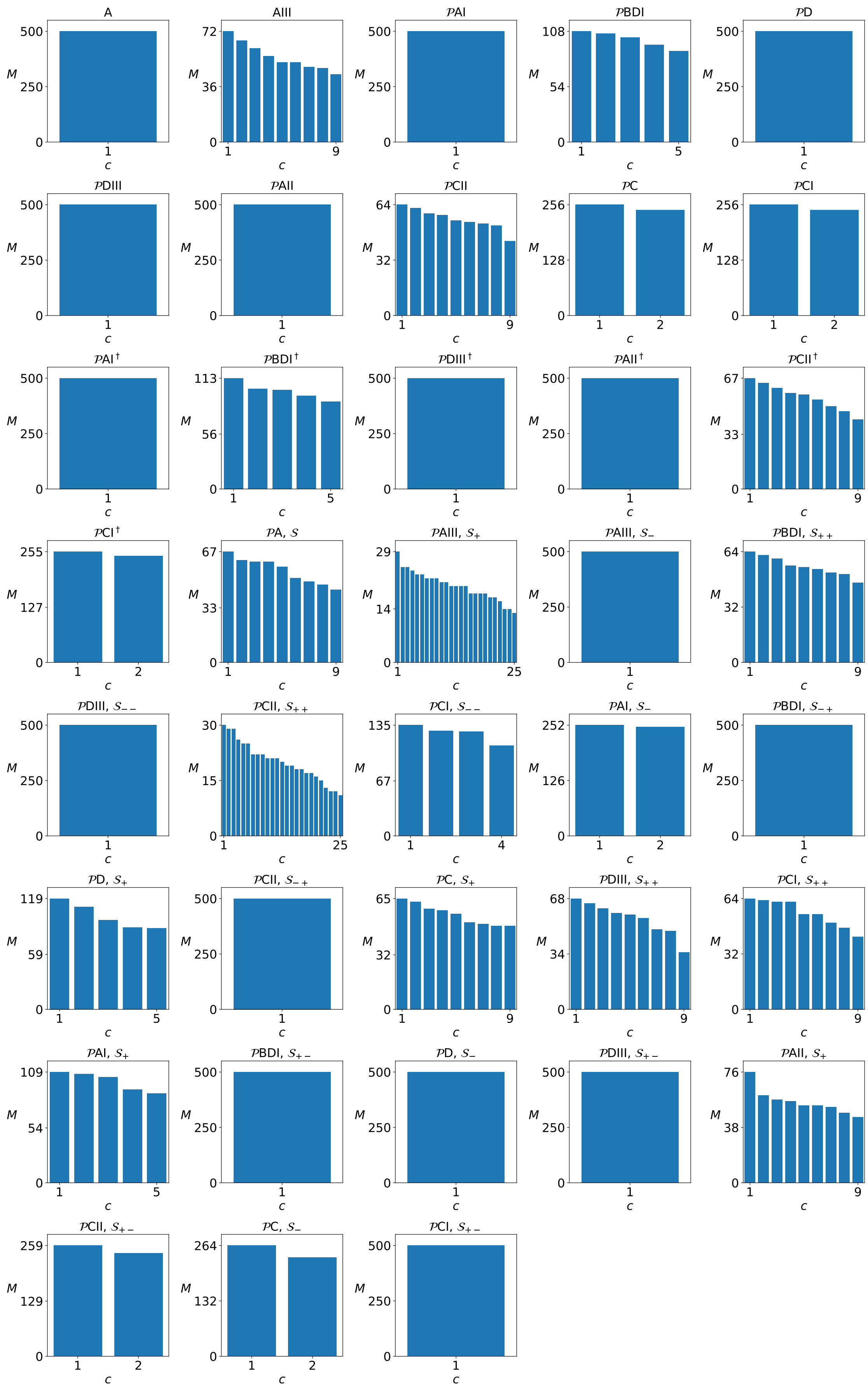

FIG. S60. Topological classifications for non-Hermitian topological systems in $d = 3$ dimension with a real line gap after considering the parity transformation.

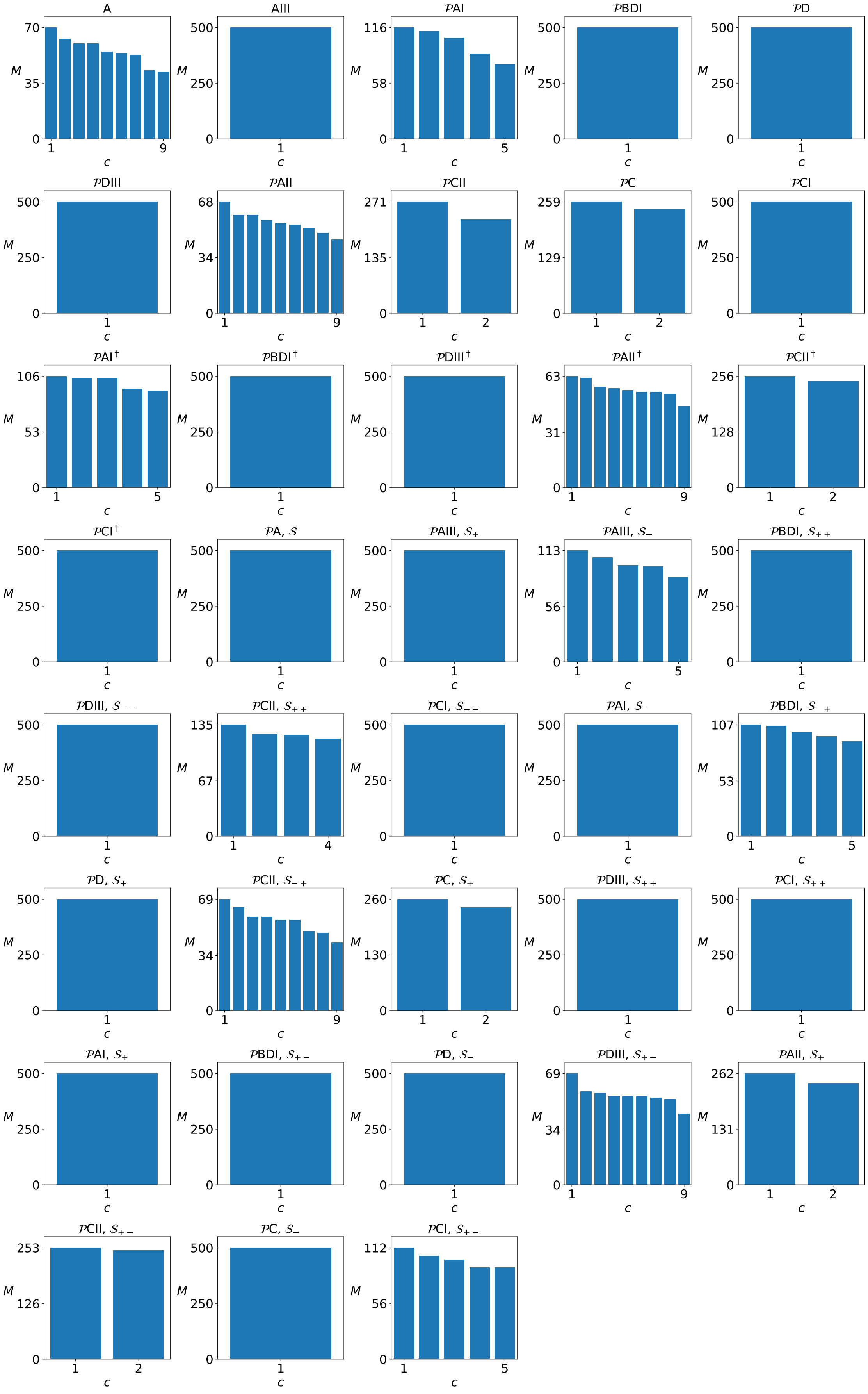

FIG. S61. Topological classifications for non-Hermitian topological systems in $d = 4$ dimension with a real line gap after considering the parity transformation.

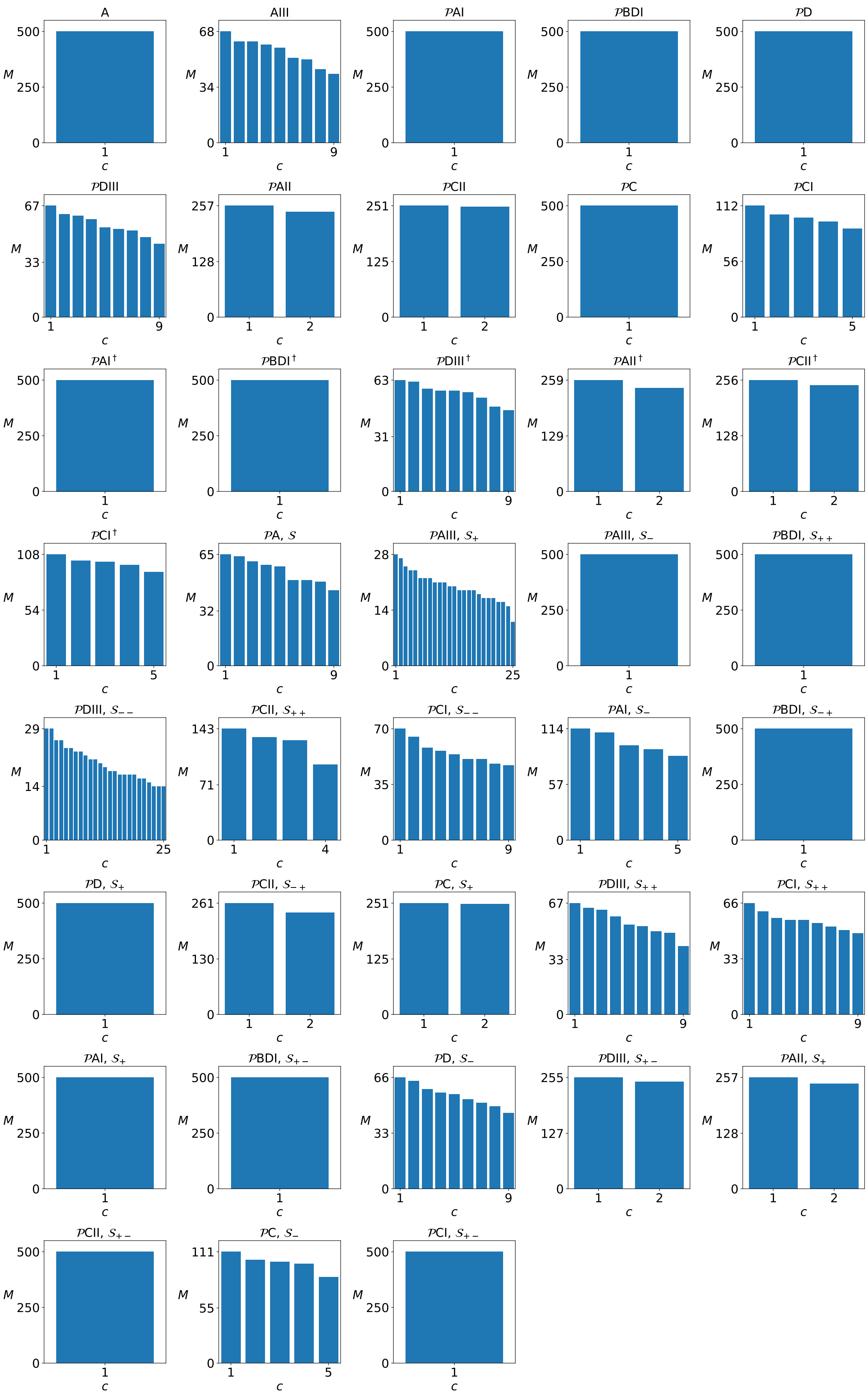

FIG. S62. Topological classifications for non-Hermitian topological systems in $d = 5$ dimension with a real line gap after considering the parity transformation.

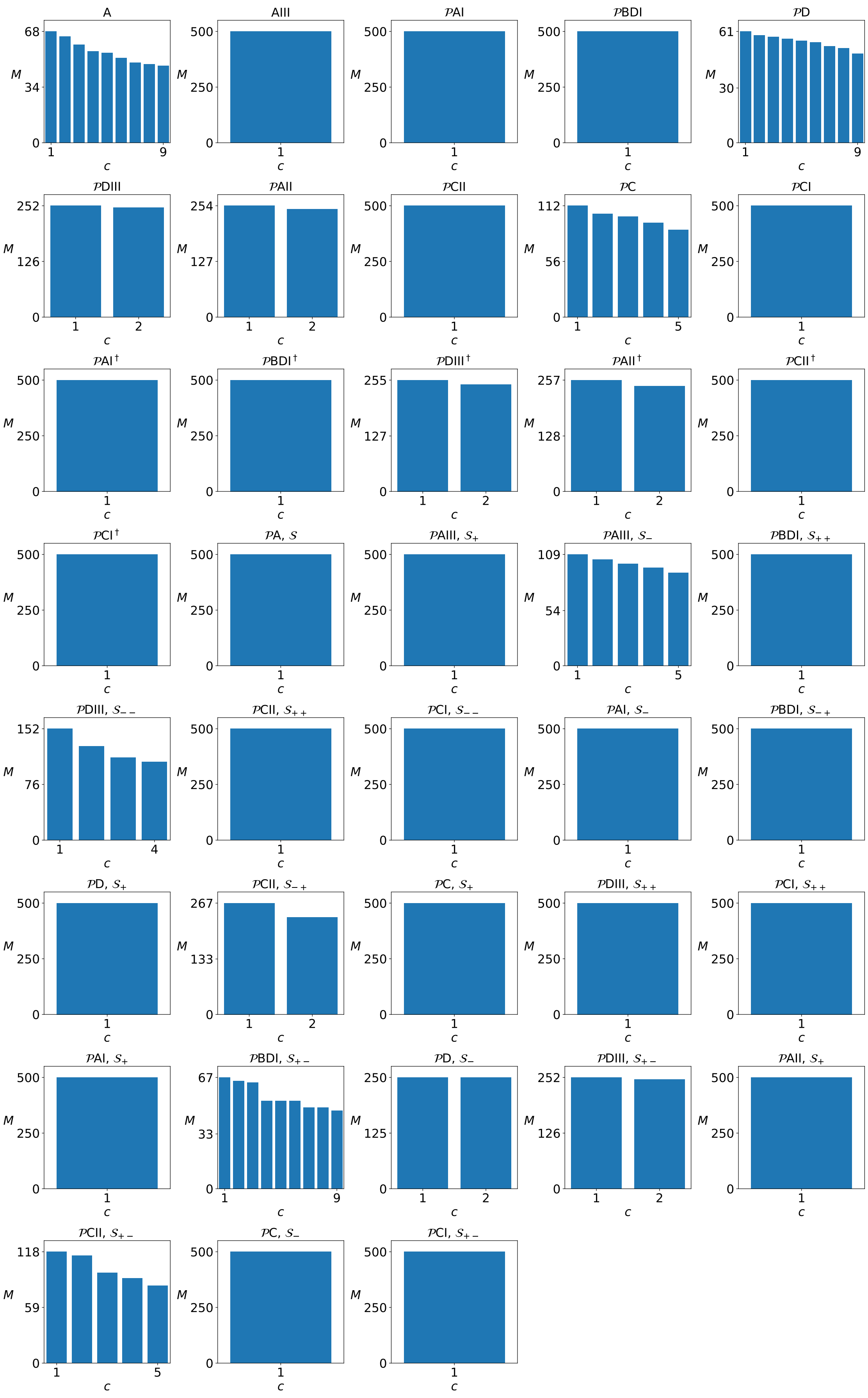

FIG. S63. Topological classifications for non-Hermitian topological systems in $d = 6$ dimension with a real line gap after considering the parity transformation.

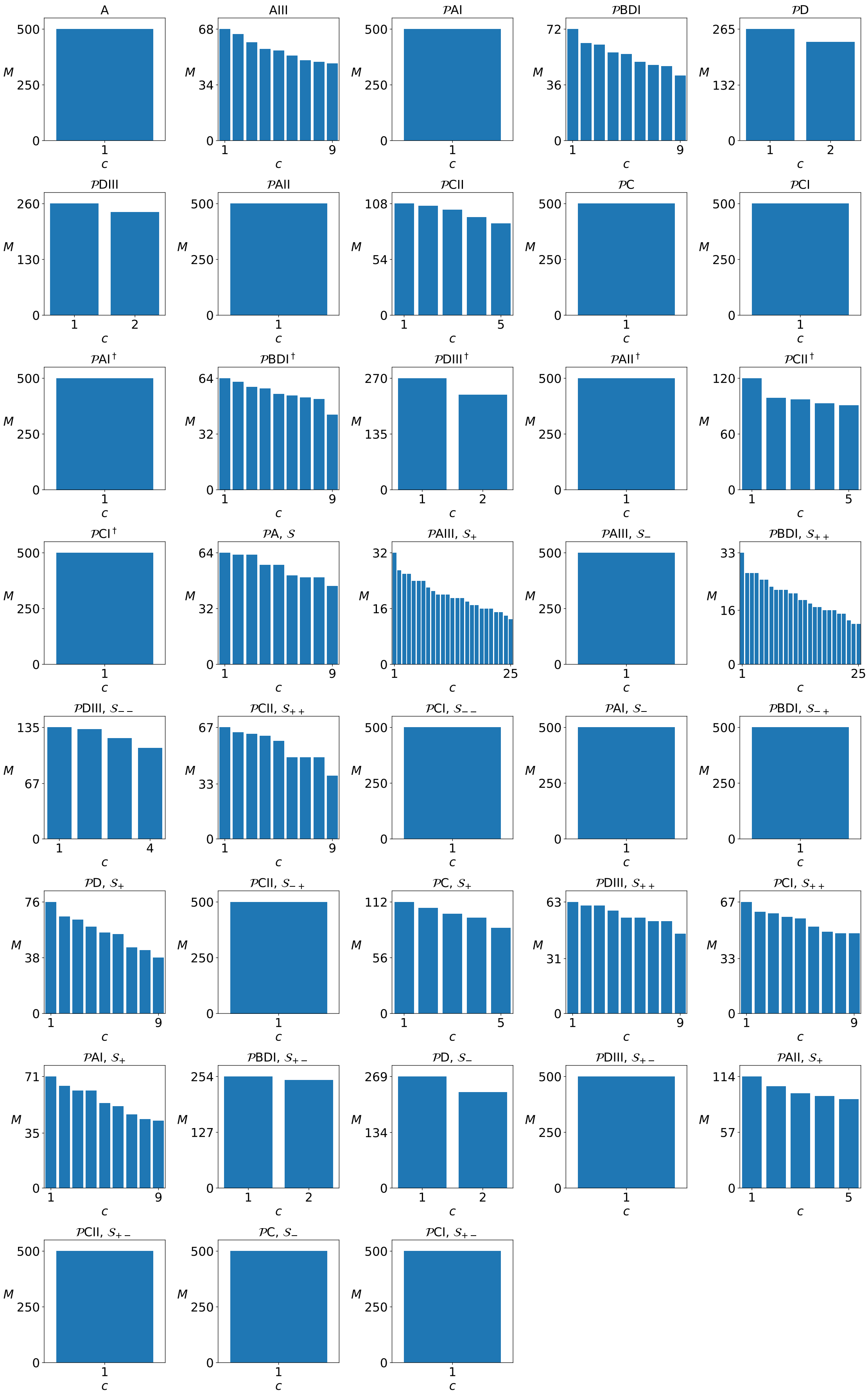

FIG. S64. Topological classifications for non-Hermitian topological systems in $d = 7$ dimension with a real line gap after considering the parity transformation.

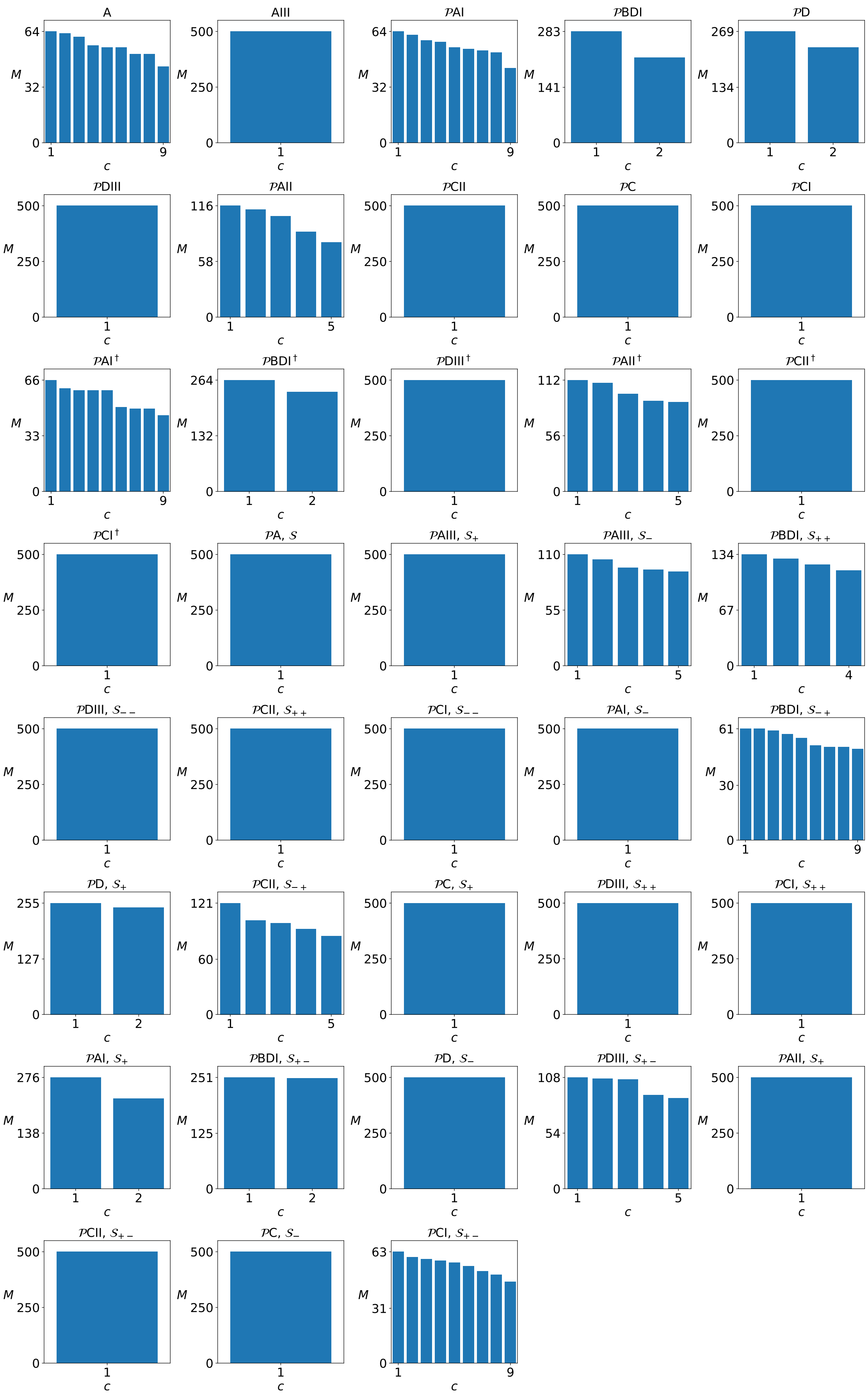

FIG. S65. Topological classifications for non-Hermitian topological systems in $d = 8$ dimension with a real line gap after considering the parity transformation.

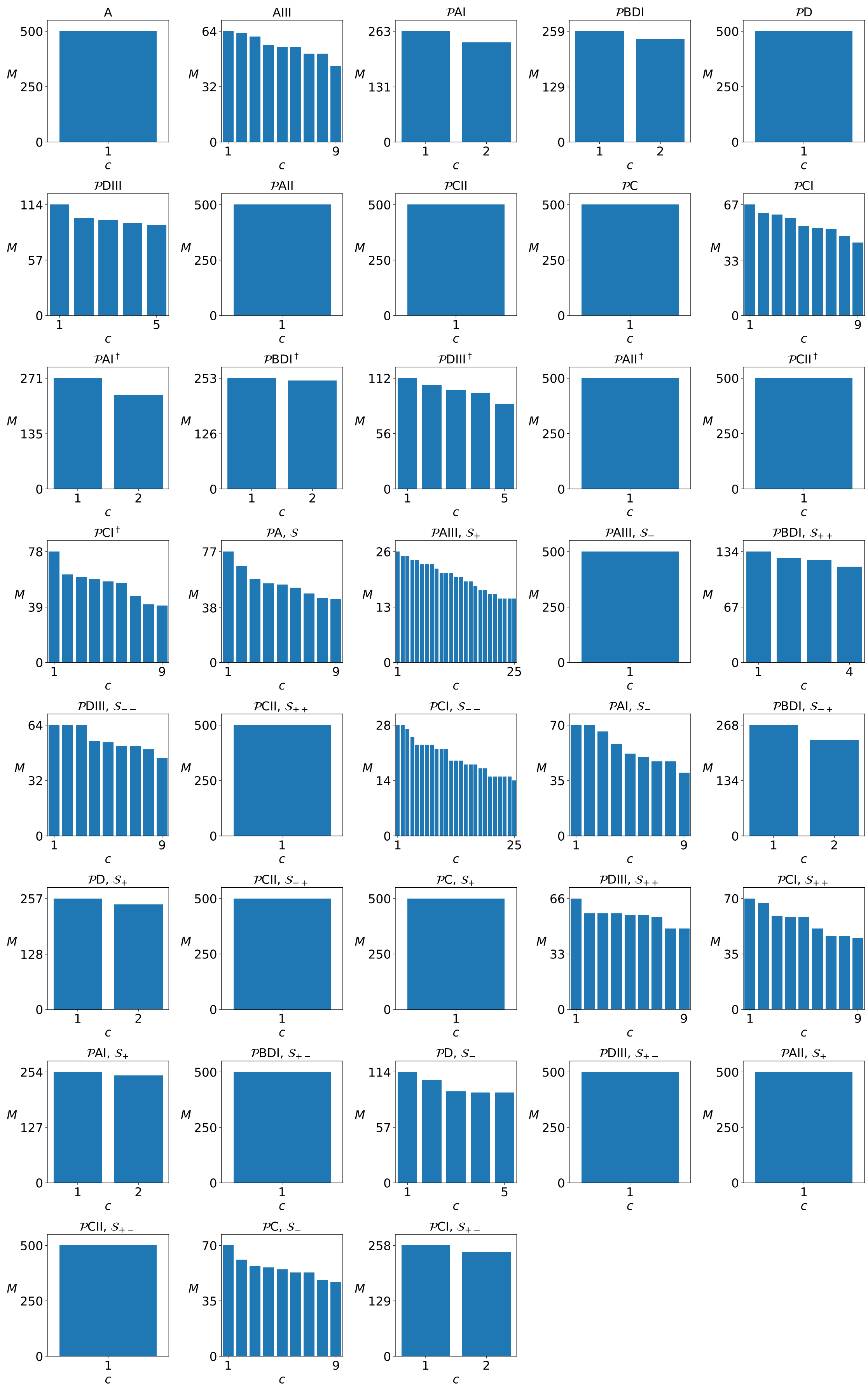

FIG. S66. Topological classifications for non-Hermitian topological systems in $d = 9$ dimension with a real line gap after considering the parity transformation.

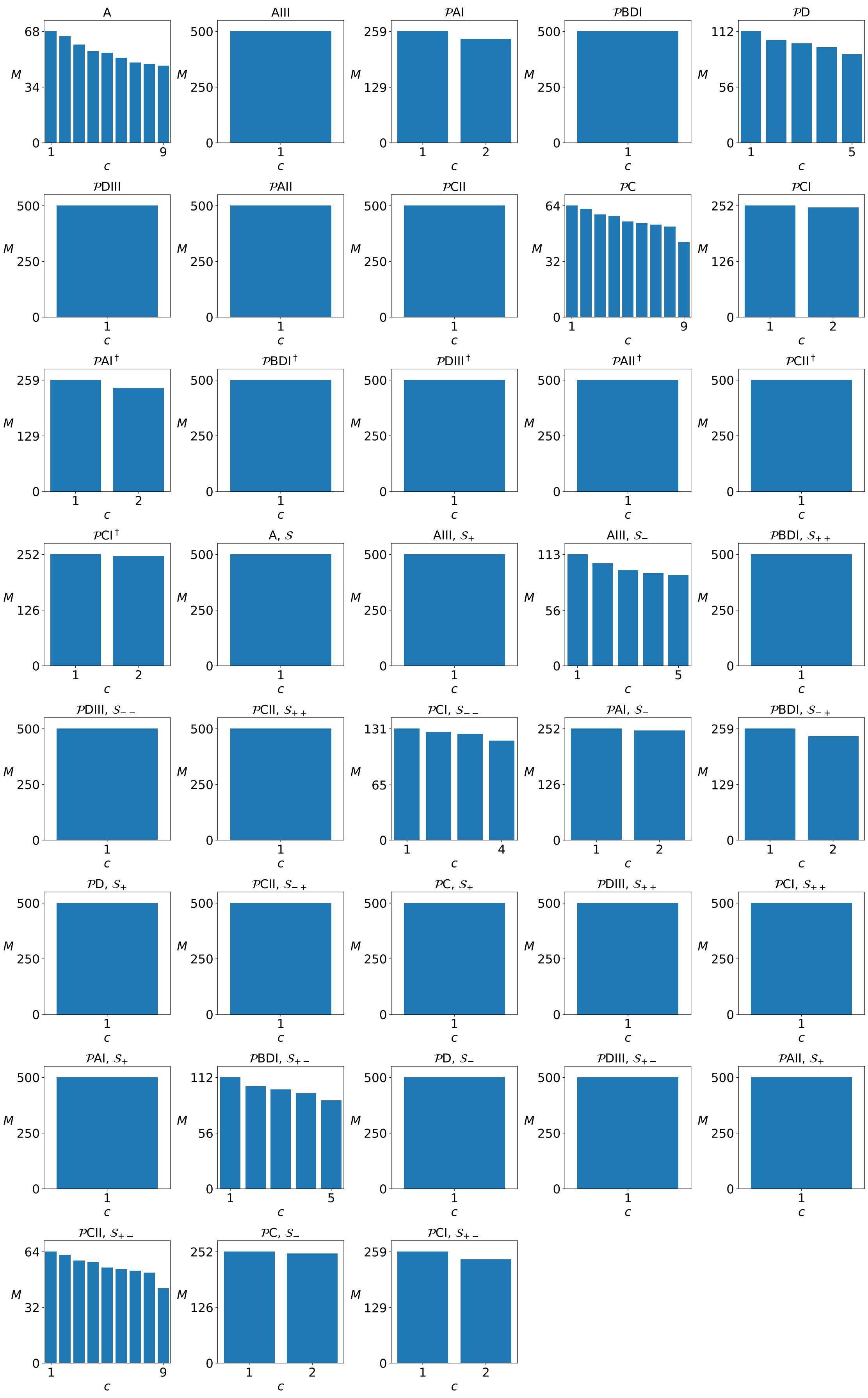

FIG. S67. Topological classifications for non-Hermitian topological systems in $d = 10$ dimension with a real line gap after considering the parity transformation.

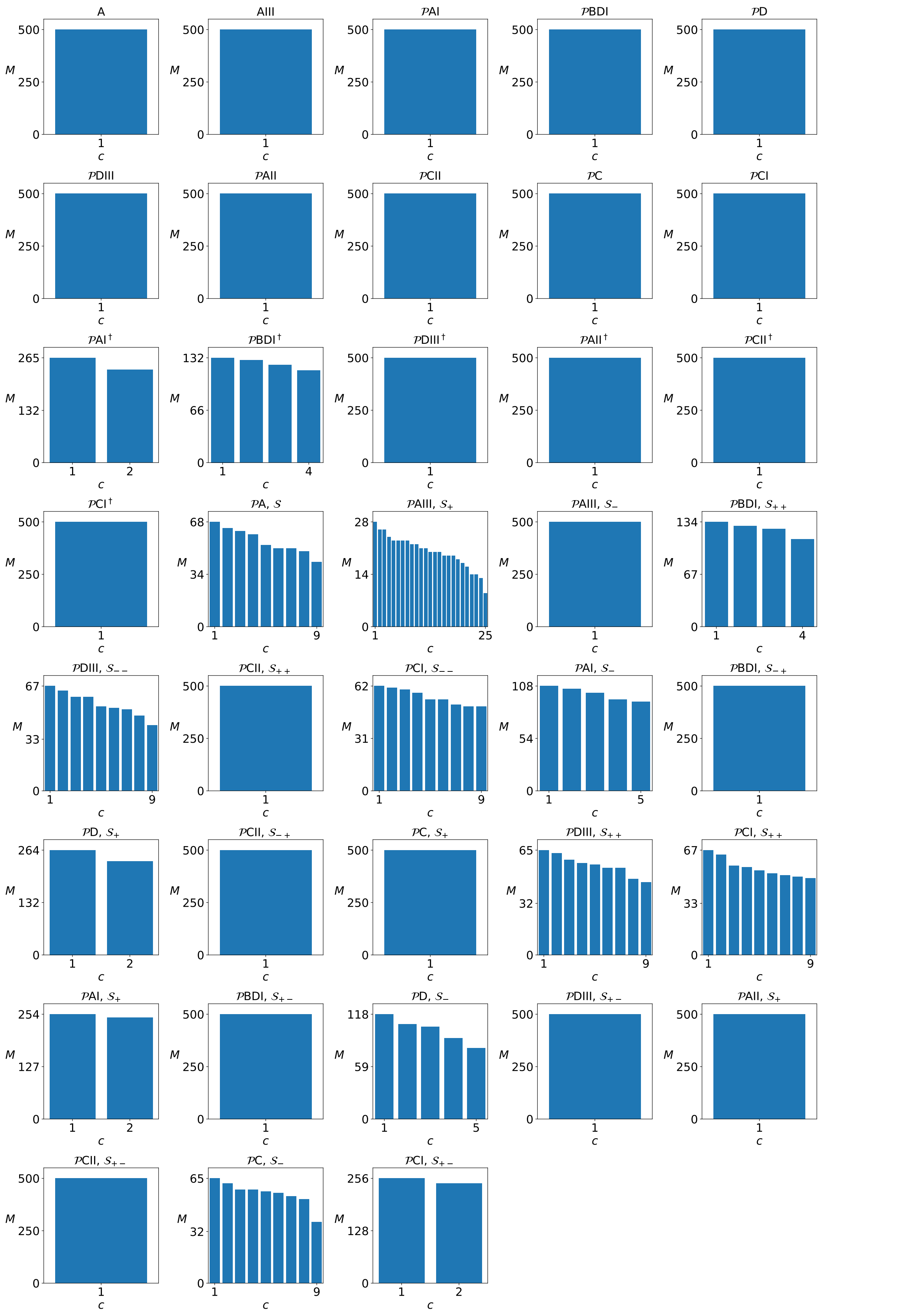

FIG. S68. Topological classifications for non-Hermitian topological systems in $d = 1$ dimension with an imaginary line gap after considering the parity transformation.

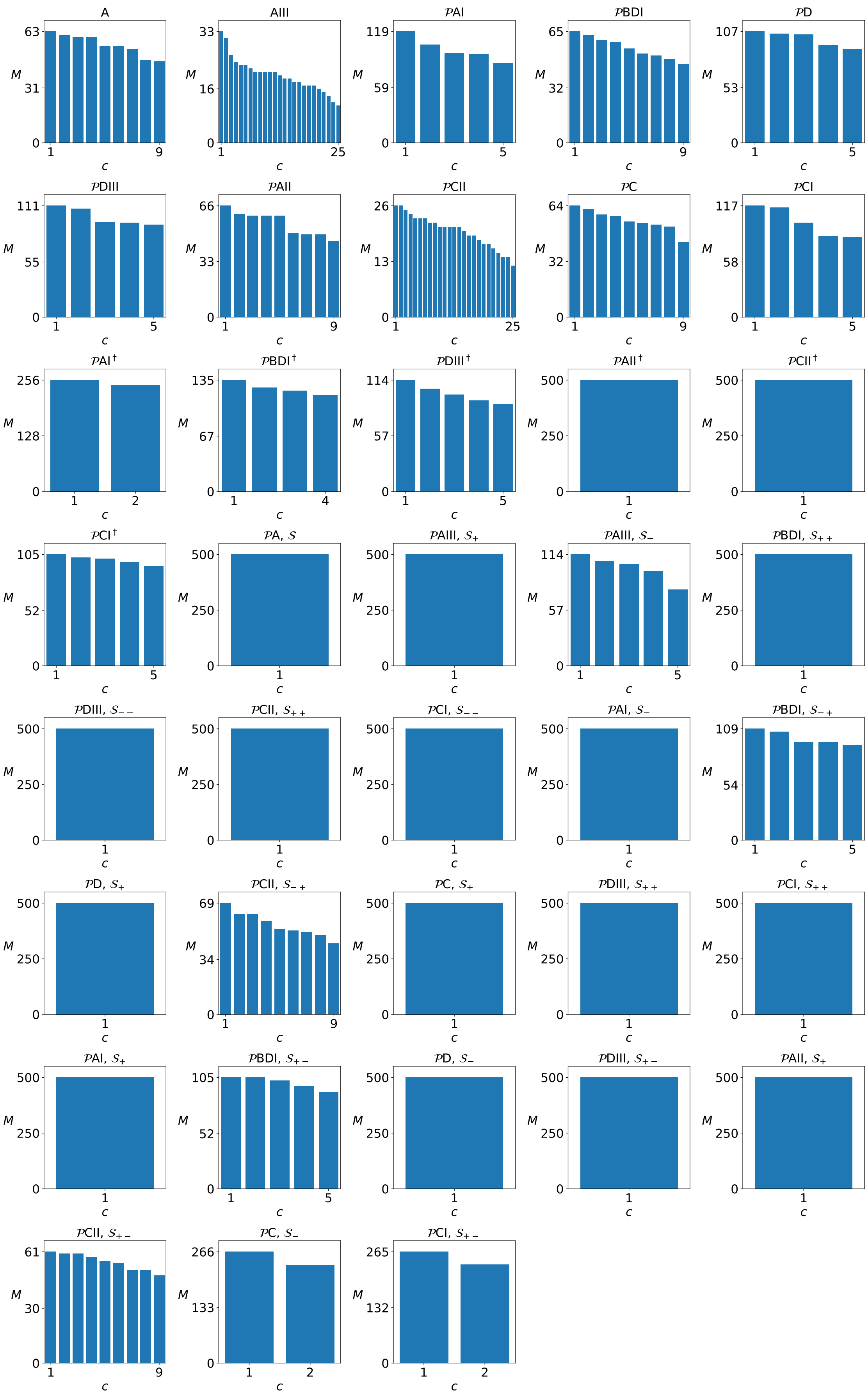

FIG. S69. Topological classifications for non-Hermitian topological systems in $d = 2$ dimension with an imaginary line gap after considering the parity transformation.

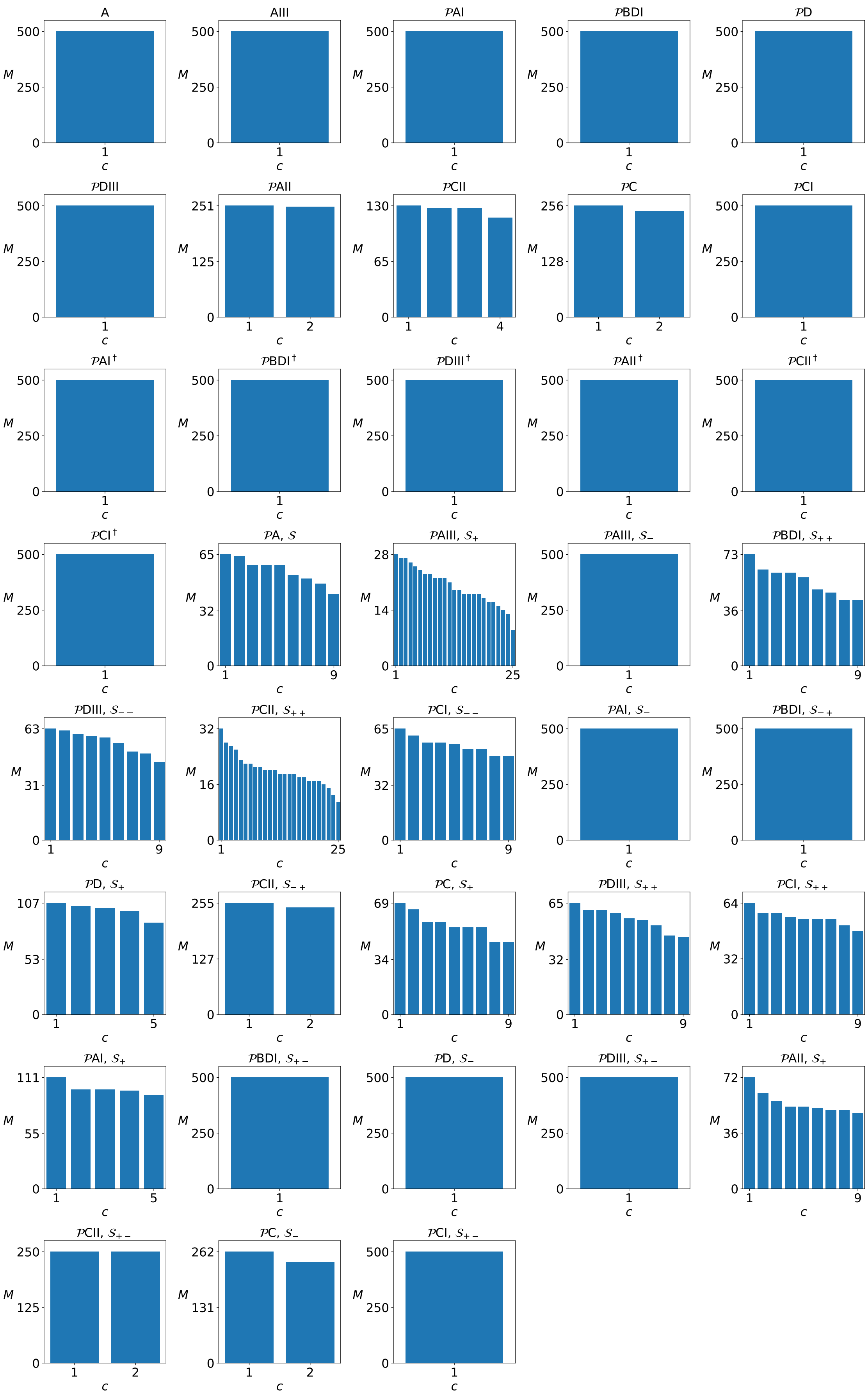

FIG. S70. Topological classifications for non-Hermitian topological systems in $d = 3$ dimension with an imaginary line gap after considering the parity transformation.

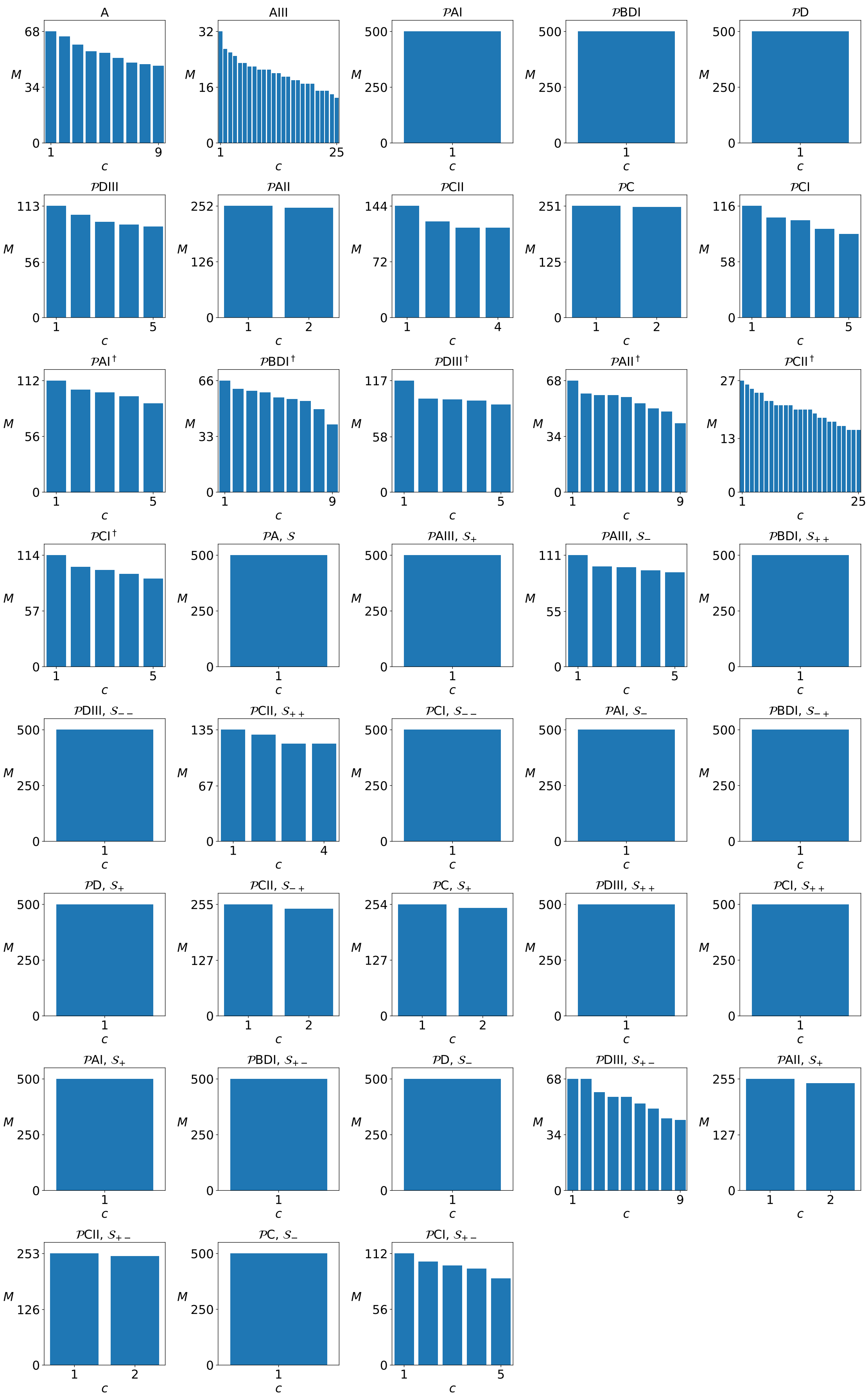

FIG. S71. Topological classifications for non-Hermitian topological systems in d = 4 dimension with an imaginary line gap after considering the parity transformation.

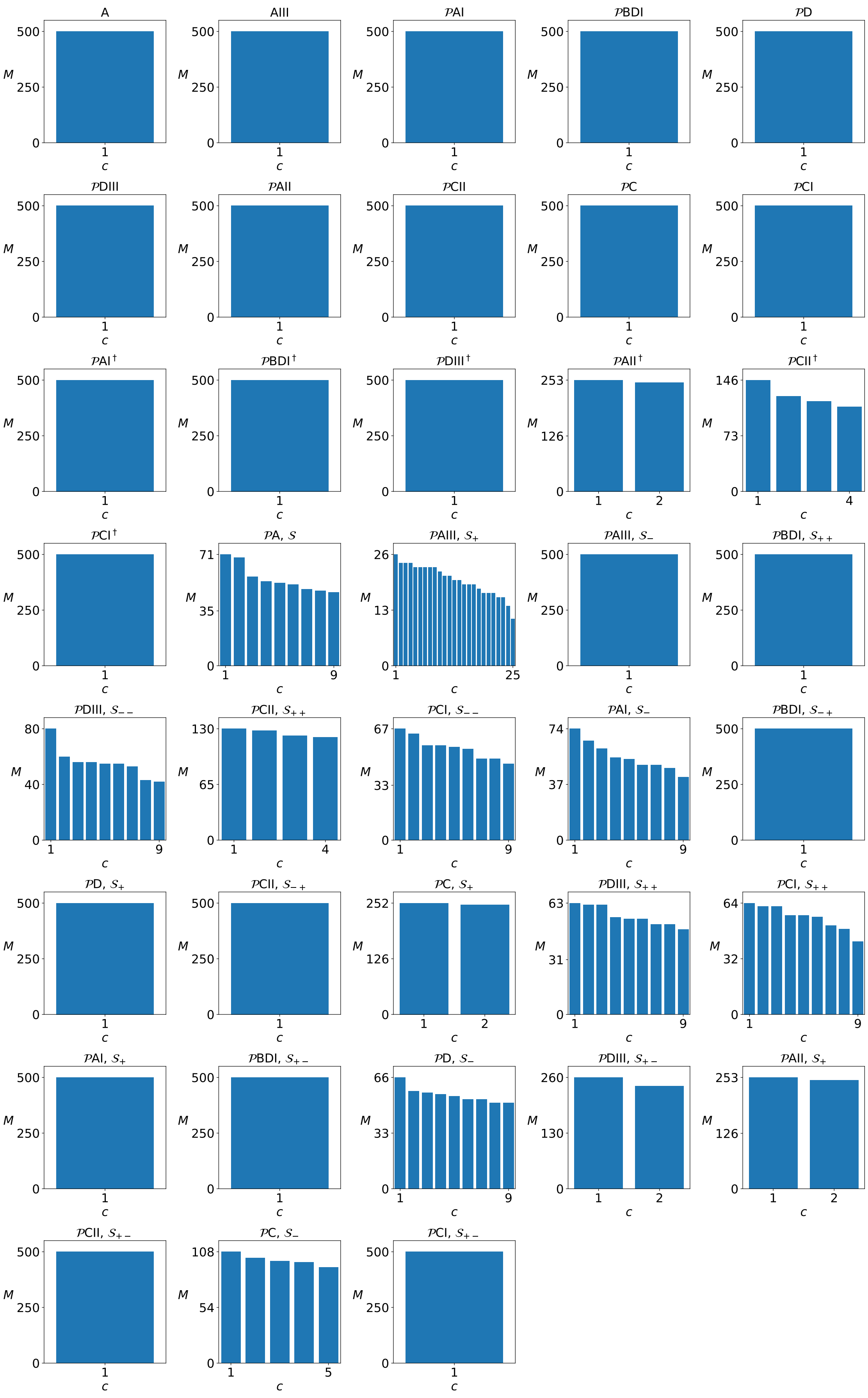

FIG. S72. Topological classifications for non-Hermitian topological systems in $d = 5$ dimension with an imaginary line gap after considering the parity transformation.

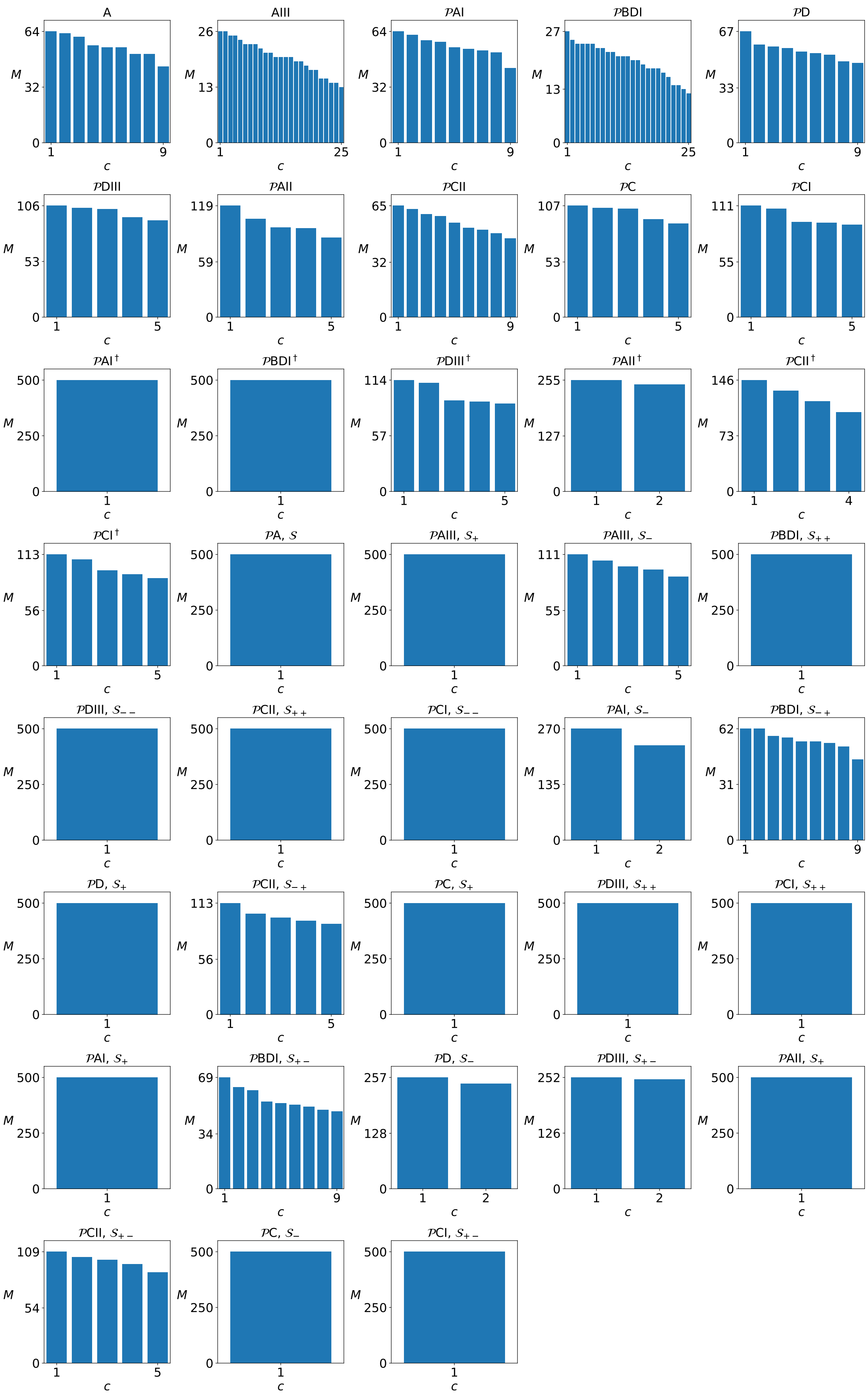

FIG. S73. Topological classifications for non-Hermitian topological systems in $d = 6$ dimension with an imaginary line gap after considering the parity transformation.

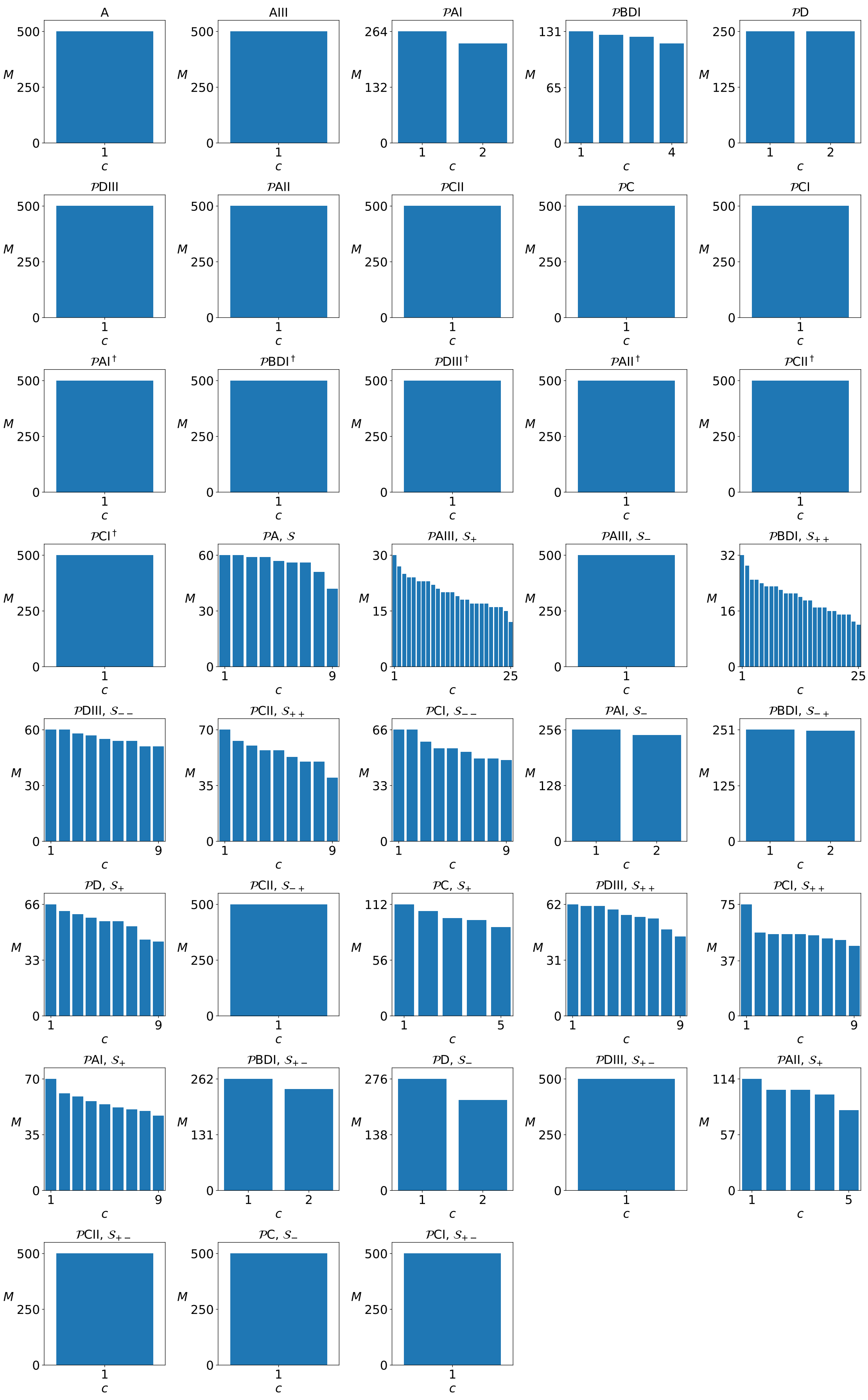

FIG. S74. Topological classifications for non-Hermitian topological systems in $d = 7$ dimension with an imaginary line gap after considering the parity transformation.

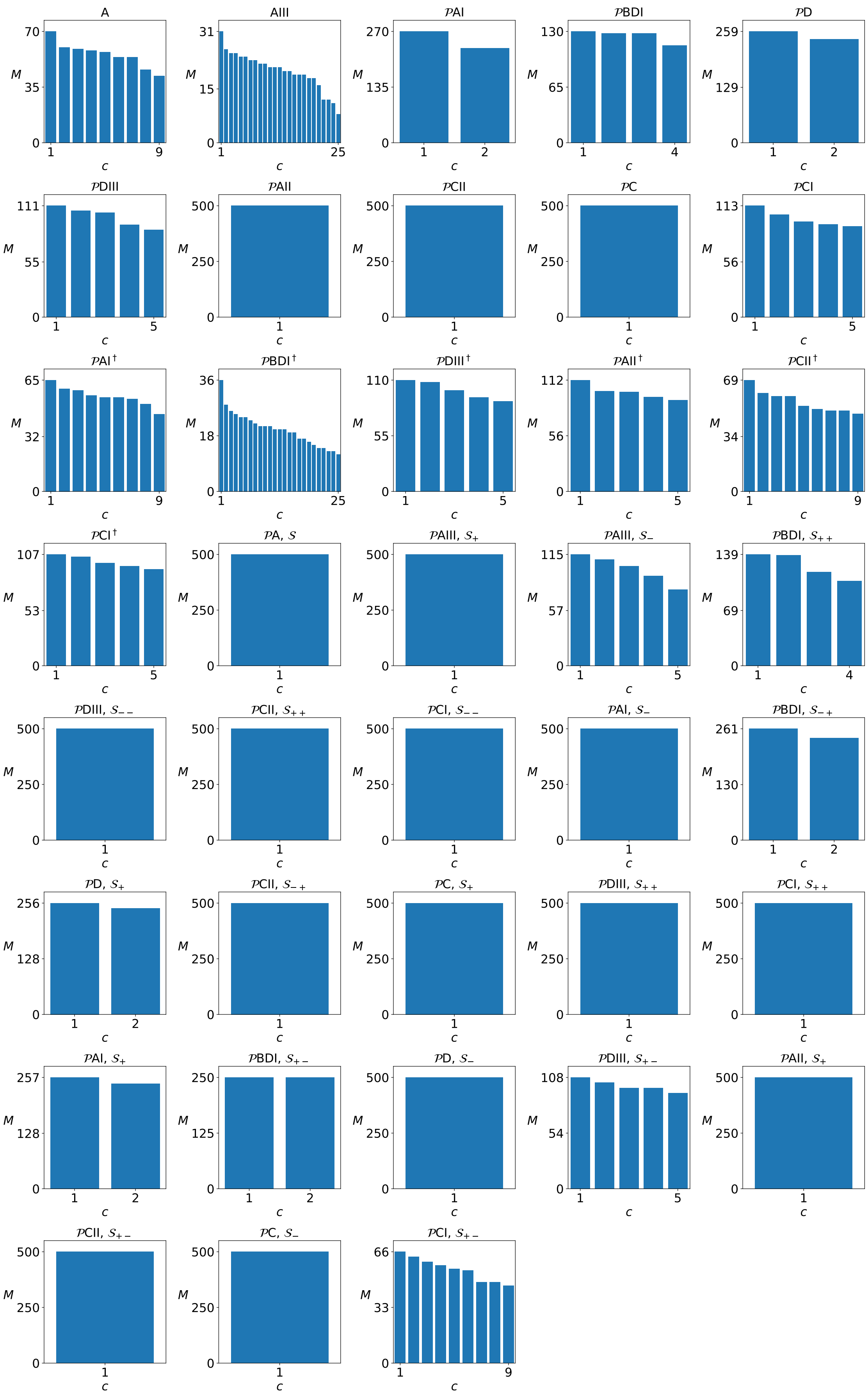

FIG. S75. Topological classifications for non-Hermitian topological systems in $d = 8$ dimension with an imaginary line gap after considering the parity transformation.

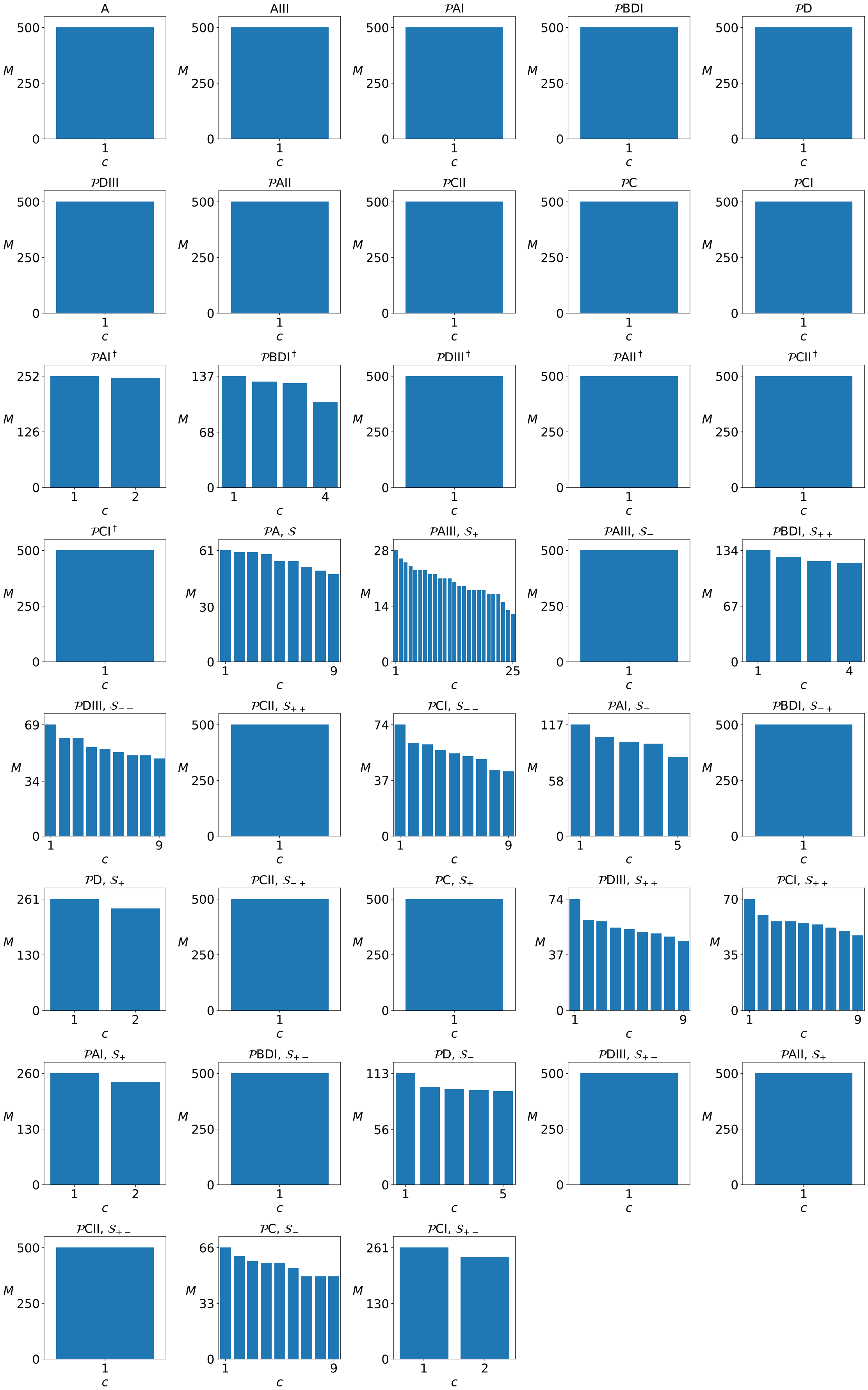

FIG. S76. Topological classifications for non-Hermitian topological systems in $d = 9$ dimension with an imaginary line gap after considering the parity transformation.

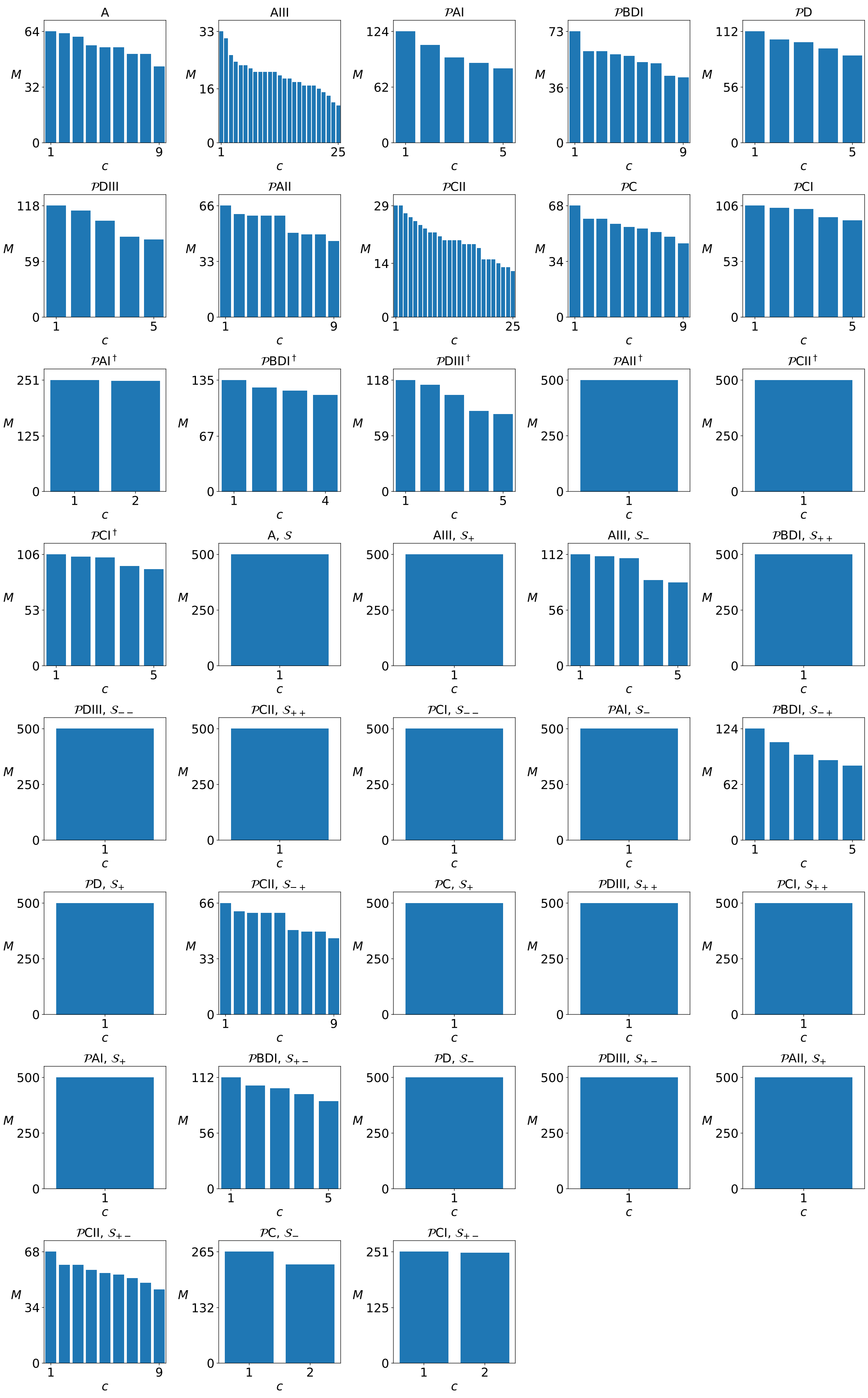

FIG. S77. Topological classifications for non-Hermitian topological systems in $d = 10$ dimension with an imaginary line gap after considering the parity transformation.
